# COP9 signalosome regulates EGFR and Notch signaling during myeloid-type progenitor cell fate decision in *Drosophila*

**DOI:** 10.1038/s44319-026-00862-w

**Published:** 2026-07-07

**Authors:** Gayatri Rai, Deepak Maurya, Debleena Mandal, Bama Charan Mondal

**Affiliations:** 1 Cytogenetics Laboratory, Department of Zoology, Institute of Science, Banaras Hindu University, Varanasi, India; 2 Cell and Developmental Biology Laboratory, School of Life Sciences, Jawaharlal Nehru University, New Delhi, India

**Keywords:** Development, Post-translational Modifications & Proteolysis, Signal Transduction

## Abstract

The COP9 signalosome (CSN) plays crucial roles in various cellular processes, including cell proliferation and DNA repair, by regulating Cullin-RING ubiquitin ligases that influence protein stability. However, the mechanism by which CSN regulates multiple signaling pathways in stem and progenitor cells for different cell fate decisions remains elusive. Here, we examine the role of the CSN in determining the fate of blood progenitors within the developing *Drosophila* larval hematopoietic organ, the lymph gland, using in vivo genetics and cell biological methods. We find that CSN-deneddylated, inactive Cullin-1 regulates the differentiation of intermediate progenitors in the lymph glands to generate a correct ratio of plasmatocytes and crystal cells. CSN also controls Serrate activation, which triggers Notch signaling in neighboring cells, leading them to become crystal cells. Moreover, CSN modulates epidermal growth factor receptor (EGFR) signaling, which regulates Notch signaling in intermediate progenitors. Our study reveals that CSN-mediated regulation of EGFR and Notch signaling is crucial for determining the cell fate of myeloid-type blood progenitors, making the lymph gland a convenient model to understand human disease caused by dysregulated EGFR and Notch signaling.

## Synopsis

In the *Drosophila* lymph gland, the COP9 signalosome (CSN) directs the differentiation of myeloid-type intermediate progenitors by regulating Serrate/Notch and EGFR signaling.

- CSN and Cullin-1 regulate the correct ratio of plasmatocytes and crystal cells.
- EGFR signaling is active during crystal cell specification.
- CSN regulates EGFR signaling and is associated with Notch signaling activity.
- CSN also controls the activation of Notch ligand Serrate.

## Abstract (web summary)

In the *Drosophila* lymph gland, the COP9 signalosome (CSN) directs the differentiation of myeloid-type intermediate progenitors by regulating Serrate/Notch and EGFR signaling.

## Graphical abstract

**Figure:**
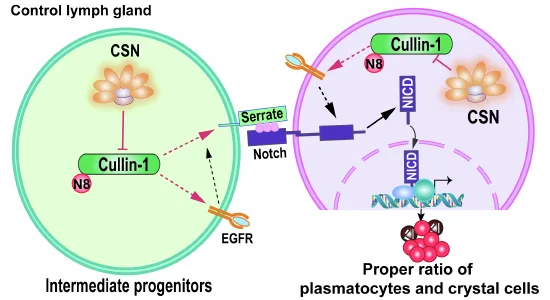

**Figure:**
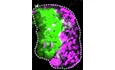

## Introduction

Signals from microenvironments and systemic signaling pathways regulate intrinsic factors that play roles in stem and progenitor cell maintenance and differentiation into specific cell types (Brunet et al, 2023). Intrinsic proteins are crucial for signaling pathway function, and their stability is required to ensure that the pathways correctly determine cell fate (Rape, 2018). Post-translational modifications affect protein stability through ubiquitin ligase-mediated degradation, ultimately regulating activities of various signaling pathways (Wolf and Menssen, 2018; Zhong et al, 2023). However, the mechanisms by which the combined activities of different signaling pathways regulate the ability of stem and progenitor cells to choose a specific cell fate remain elusive.

**Figure 1:**
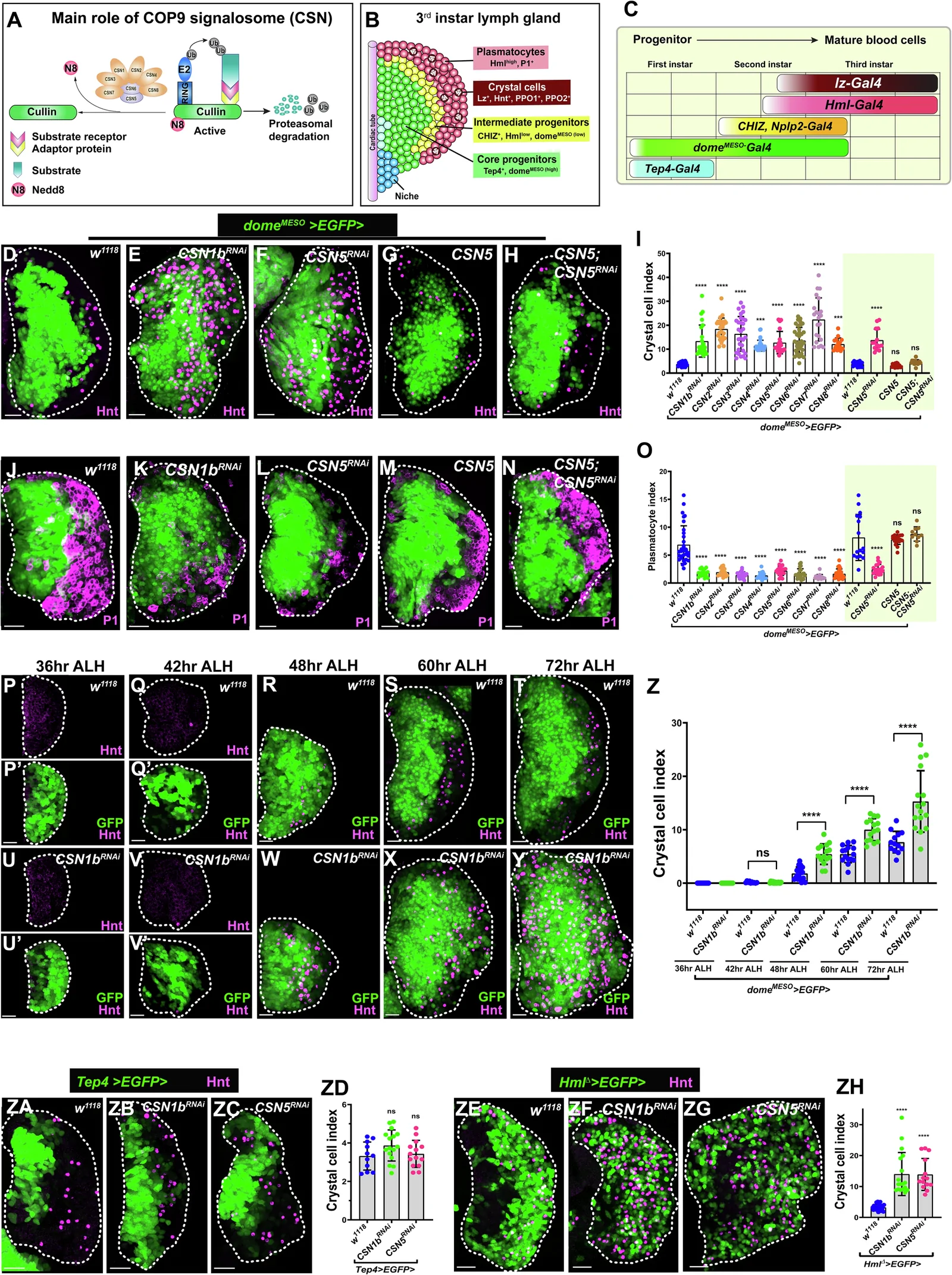
COP9 signalosome (CSN) regulates the lymph gland blood progenitors’ cell fate. (**A**) A schematic illustrating that the primary function of CSN is the deneddylation of Cullin-RING ubiquitin ligases. (**B**) A schematic of the *Drosophila* third instar larval lymph gland lobe showing different types of cells with their known genetic markers. (**C**) A schematic illustrating different Gal4 driver expressions in various cell types during lymph gland development. (**D**–**H**) Knockdown of CSN complex in lymph gland progenitors (green) using *dome*^*MESO*^*-Gal4, UAS-EGFP* driver by expressing *CSN1b*^*RNAi*^ (**E**) and *CSN5*^*RNAi*^ (**F**) increases the Hnt-positive (magenta) crystal cell number compared to control *w*^*1118*^ (**D**). Overexpression of CSN5 in the background of *CSN5*^*RNAi*^ in the progenitor rescued *CSN5*^*RNAi*^-mediated increase in crystal cell number (**H**). Overexpression of CSN5 does not alter crystal cell number (**G**). (**I**) Crystal cell index per lymph gland lobe in (**D**–**H**). *w*^*1118*^ (*n* = 20), *CSN1b*^*RNAi*^ (*n* = 20, *P* < 0.0001), *CSN2*^*RNAi*^ (*n* = 25, *P* < 0.0001), *CSN3*^*RNAi*^ (*n* = 30, *P* < 0.0001), *CSN4*^*RNAi*^ (*n* = 17, *P* = 0.0001), *CSN5*^*RNAi*^ (*n* = 19, *P* < 0.0001), *CSN6*^*RNAi*^ (*n* = 36, *P* < 0.0001), *CSN7*^*RNAi*^ (*n* = 22, *P* < 0.0001), and *CSN8*^*RNAi*^ (*n* = 16, *P* < 0.0001). *w*^*1118*^ (*n* = 18), *CSN5*^*RNAi*^ (*n* = 14, *P* < 0.0001), *CSN5* (*n* = 17, *P* = 0.8730), and *CSN5; CSN5*^*RNAi*^ (*n* = 14, *P* = 0.8086). (**J**–**N**) Using *dome*^*MESO*^*-Gal4, UAS-EGFP* driver by expressing *CSN1b*^*RNAi*^ (**K**) and *CSN5*^*RNAi*^ (**L**) caused reduced plasmatocyte number marked by P1 (magenta) compared to the control lymph gland (*dome*^*MESO*^*-Gal4, UAS-EGFP/+*). Overexpression of CSN5 in the background of *CSN5*^*RNAi*^ in the progenitor rescued *CSN5*^*RNAi*^-mediated decrease in plasmatocyte volume (**N**). Overexpression of CSN5 does not alter the amount of plasmatocytes (*n* = 18, *P* = 0.9571) (**M**). (**O**) Plasmatocyte index per lymph gland lobe in (**J**–**N**). *w*^*1118*^ (*n* = 32), *CSN1b*^*RNAi*^ (*n* = 19, *P* < 0.0001), *CSN2*^*RNAi*^ (*n* = 19, *P* < 0.0001), *CSN3*^*RNAi*^ (*n* = 18, *P* < 0.0001), *CSN4*^*RNAi*^ (*n* = 17, *P* = 0.4598), *CSN5*^*RNAi*^ (*n* = 19, *P* < 0.0001), *CSN6*^*RNAi*^ (*n* = 23, *P* = 0.8429), *CSN7*^*RNAi*^ (*n* = 15, *P* = 0.9329), and *CSN8*^*RNAi*^ (*n* = 23, *P* = 0.4753). *w*^*1118*^ (*n *= 16), *CSN5*^*RNAi*^ (*n* = 23, *P* < 0.0001), *CSN5* (*n* = 18, *P* = 0.9571), and *CSN5; CSN5*^*RNAi*^ (*n* = 10, *P* = 0.9024). (**P**–**Y**) Lymph glands of control (*dome*^*MESO*^*-Gal4/+*) (**P**–**T**) and CSN1b knockdown (*CSN1b*^*RNAi*^) (U-Y) are compared at 36, 42, 48, 60, and 72 h ALH. Progenitors are marked in green, and *CSN1b*^*RNAi*^ shows a notable increase in crystal cell numbers (magenta) starting at 48 h ALH. For better understanding we have shown magenta channel for crystal cells separately for 36 h ALH (**P**, **U**) and 42 h ALH (**Q**, **V**). (**Z**) Crystal cell index in (**P**–**Y**). *CSN1b*^*RNAi*^ (*n* = 12), and *w*^*1118*^ (*n* = 14) at 36 h ALH. *CSN1b*^*RNAi*^ (*n* = 14, *P* = 0.6084), and *w*^*1118*^ (*n* = 10) at 42 h ALH. *CSN1b*^*RNAi*^ (*n* = 15, *P* < 0.0001), and *w*^*1118*^ (*n* = 18) at 48 h ALH. *CSN1b*^*RNAi*^ (*n* = 15, *P* < 0.0001), and *w*^*1118*^ (*n* = 15) at 60 h ALH. *CSN1b*^*RNAi*^ (*n* = 16, *P* < 0.0001), and *w*^*1118*^ (*n *= 13) at 72 h ALH. (**ZA**–**ZC**) Knockdown of CSN complexes in core progenitors (green) using *Tep4-Gal4, UAS-2XEGFP* by expressing *CSN1b*^*RNAi*^ (**ZB**), and *CSN5*^*RNAi*^ (ZC) did not show any significant difference in the crystal cell numbers (magenta) as compared to control (**ZA**). (**ZD**) Crystal cell index in (**ZA**–**ZC**). *CSN1b*^*RNAi*^ (*n* = 16, *P* = 0.313), *CSN5*^*RNAi*^ (*n* = 14, *P* = 0.9852), and *w*^*1118*^ (*n* = 12). (**ZE**–**ZG**) Knockdown of CSN using a differentiated zone driver *Hml*^*Δ*^*-Gal4, UAS-EGFP* by expressing *CSN1b*^*RNAi*^ (**ZF**), and *CSN5*^*RNAi*^ (**ZG**) resulted in a drastic increase in crystal cell (magenta) as compared to control (**ZE**). (**ZH**) Crystal cell index in (**ZE**–**ZG**). *CSN1b*^*RNAi*^ (*n* = 18, *P* < 0.0001), *CSN5*^*RNAi*^ (*n *= 14, *P* < 0.0001), and *w*^*1118*^ (*n* = 23). All confocal images represent maximum-intensity projections of the middle third optical sections of the wandering third-instar lymph gland lobe from at least three independent biological experiments. White dashed lines demarcate one lymph gland lobe. Scale bar, 25 μm. Error bars in graphs: mean ± S.D. n.s.: not significant (*P* ≥ 0.05); **P < 0.01; ***P < 0.001; ****P < 0.0001. One-way ANOVA with Tukey’s post-hoc test was used for analysis involving (**I**,** O**,** ZD**, **ZH**). A two-tailed, unpaired Student’s *t* test was employed to compare the mean value of the two groups in (**Z**). Detailed genotypes are in Appendix Table S1. Source data are available online for this figure.

The COP9 signalosome (CSN) is an eight-subunit (CSN1 to CSN8) multifunctional complex protein found in all eukaryotes. CSN holoenzyme’s primary function is to remove the ubiquitin-like protein Nedd8 (deneddylation) from the Cullin-RING-ubiquitin ligase complex to make it inactive (Fig. 1A) (Schulze-Niemand and Naumann, 2023; Wei et al, 2008). However, some of the CSN subunits play other functions individually (Pan et al, 2014; Suisse et al, 2017). The CSN-mediated deneddylation process is crucial for protein degradation, supporting various cellular functions, including the control of the cell cycle and regulation of the DNA damage response (DDR) (Fuzesi-Levi et al, 2014; Hannss and Dubiel, 2011; Meir et al, 2015; Wang et al, 2021; Wei et al, 2008). CSN regulates signaling pathways in different tissues and model systems, including *Drosophila* (Deng et al, 2011; Harari-Steinberg et al, 2007; Huang et al, 2014). In *Drosophila*, CSN-mediated deneddylation destabilizes Cubitus interruptus (Ci), which regulates low-to-intermediate Hedgehog (Hh) signaling cells in wing discs (Wu et al, 2011); likewise, CSN-Hh signaling is involved in proper oogenesis(Lu et al, 2015) and R cell differentiation in the eye (Suh et al, 2002). CSN also regulates epidermal growth factor receptor (EGFR) signaling in development (Suisse et al, 2017) and diseases, including cancer (Fang et al, 2015; Najor et al, 2017; Su et al, 2020). In a genome-wide RNA interference (RNAi) screening, CSN was identified as a regulator of Notch in determining binary cell fate choices during *Drosophila* sensory organ development (Mummery-Widmer et al, 2009). However, how CSN regulates Notch signaling remains unclear.

Notch signaling affects many cellular functions across species (Bray, 2016; Sachan et al, 2023). EGFR signaling is also involved in various cellular functions, including stem and progenitor cell maintenance, proliferation, and differentiation (Chabu et al, 2017; Cho et al, 2024; Goins et al, 2024; Levantini et al, 2022; Schweitzer and Shilo, 1997). Context-specific EGFR and Notch signaling pathways work together in some cases but antagonistically in others (Doroquez and Rebay, 2006; Flores et al, 2000; Sundaram, 2005). However, the potential role of CSN in cell fate determination by regulating EGFR and Notch signaling pathways remains unexplored.

*Drosophila melanogaster* is widely used to study hematopoiesis due to its excellent in vivo genetic tools for investigating gene regulatory networks (Banerjee et al, 2019; Boulet et al, 2018). Development of the hematopoietic system in mammals and *Drosophila* depends on evolutionarily conserved signaling pathways and transcription factors. *Drosophila* has three terminally differentiated myeloid-type blood cells: plasmatocytes, the most common phagocytic blood cells, which remove pathogens and cellular debris, like mammalian macrophages (Maurya et al, 2024). Crystal cells contribute to wound healing, melanization, blood clotting, and oxygen sensing (Banerjee et al, 2019; Shin et al, 2024). Crystal cell formation requires activation of Serrate/Notch signaling and Runx family transcription factor Lozenge (Lz) (Duvic et al, 2002; Lebestky et al, 2003; Terriente-Felix et al, 2013). Lamellocytes are rare, large, flat cells that encapsulate and neutralize bulky invaders like wasp embryos (Anderl et al, 2016; Csordas et al, 2021; Rizki and Rizki, 1992). Lamellocytes and crystal cells don’t proliferate or differentiate into other cell types, but plasmatocytes can proliferate and transdifferentiate into crystal cells or lamellocytes whenever needed (Anderl et al, 2016; Marcetteau et al, 2025). The *Drosophila* larval hematopoietic organ, called the lymph gland, begins to develop during the late embryonic stage. It matures during the larval stages and releases blood cells called hemocytes to fill the pupa and adult. The third instar larval lymph gland comprises distinct functional zones containing specialized cells. In the medullary zone, signals from the niche, including inputs from posterior signaling center cells, the cardiac tube, and the differentiated zone, safeguard core and distal progenitor cells from premature differentiation (Fig. 1B) (Banerjee et al, 2019; Jung et al, 2005; Kharrat et al, 2022; Morin-Poulard et al, 2016). Studies showed that multiple signaling pathways, such as Hh signaling from the niche, cell-autonomous factors downstream of EGFR, *Drosophila* PDGF/VEGF receptor (Pvr), JAK/STAT, and systemic signals (e.g., insulin-like peptides, neurotransmitters) regulate lymph gland progenitor maintenance (Banerjee et al, 2019; Goins et al, 2024; Jung et al, 2005; Kapoor et al, 2022; Krzemien et al, 2007; Lebestky et al, 2003; Liu et al, 2025; Mondal et al, 2014; Shim et al, 2013). However, the mechanism that determines the correct number of various blood cell types during progenitor differentiation remains unknown.

Here, we focus on the CSN-mediated mechanisms that influence the cell fate decisions of myeloid-type progenitors, leading to the differentiation into plasmatocytes and crystal cells. We find that CSN modulation of EGFR and Notch signals is critical in deciding the fate of myeloid-type progenitor cells in the lymph gland. Notably, we demonstrate that, in addition to active Notch signaling, EGFR signaling also contributes to the specification of crystal cells. We reveal a genetic association between CSN and Serrate-dependent Notch activation during crystal cell development in the lymph gland. Our research findings suggest that the regulation of EGFR and Notch signaling mediated by CSN might contribute to the development of cancers and other diseases, thereby facilitating the development of potential CSN-targeted therapeutic interventions.

## Results

### COP9 signalosome (CSN) regulates the lymph gland blood progenitors’ cell fate

**Figure EV1:**
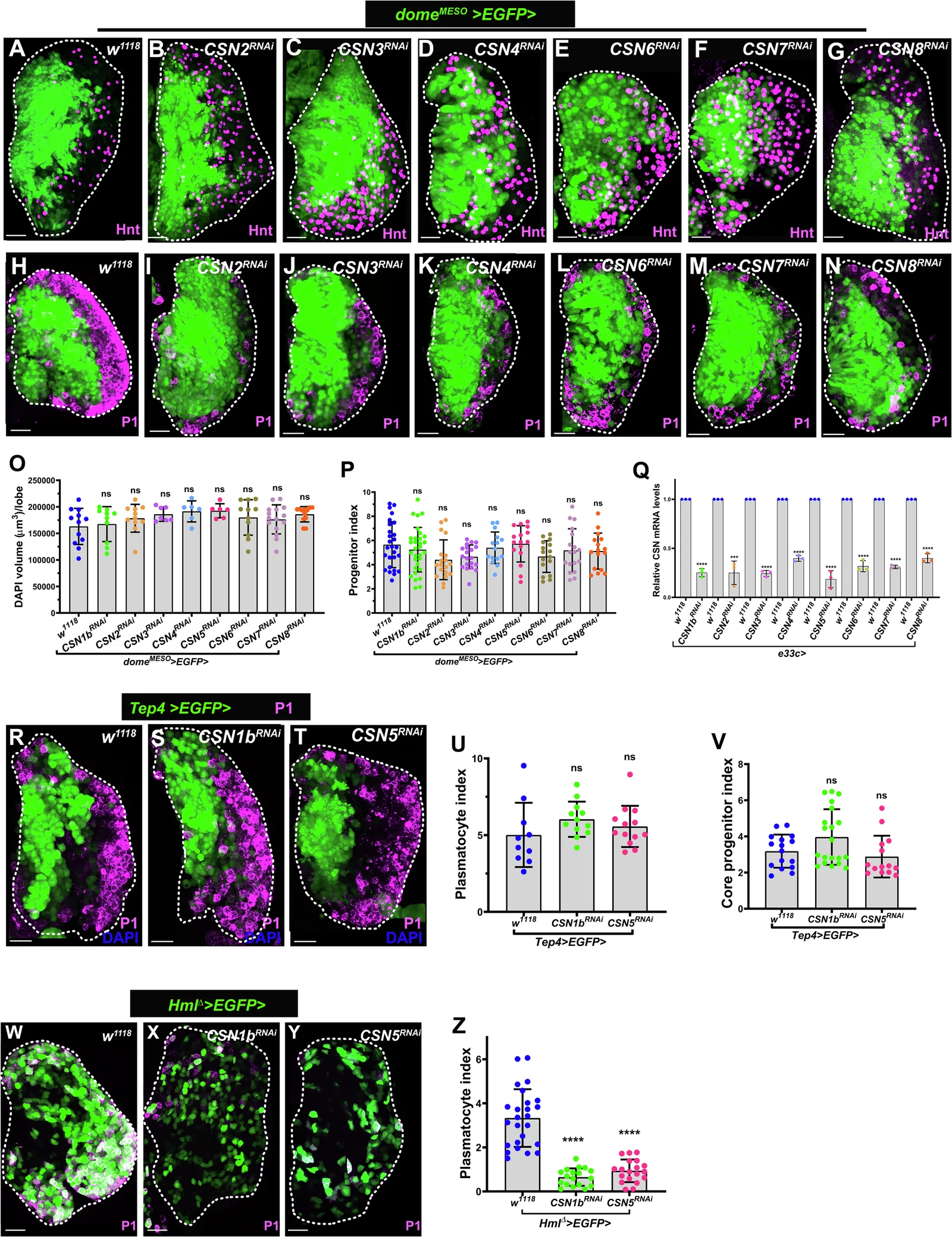
COP9 signalosome (CSN) regulates the lymph gland blood progenitors’ cell fate, related to Fig. 1. (**A**–**G**) Knockdown of CSN complex in lymph gland progenitors (green) using *dome*^*MESO*^*-Gal4, UAS-EGFP* driver by expressing *CSN2*^*RNAi*^ (**B**), *CSN3*^*RNAi*^ (**C**), *CSN4*^*RNAi*^ (**D**), *CSN6*^*RNAi*^ (**E**), *CSN7*^*RNAi*^ (**F**), and *CSN8*^*RNAi*^ (**G**), increases the Hnt positive (magenta) crystal cell number compared to control *w*^*1118*^ (**A**). The quantification of the crystal cell index is shown in Fig. 1I. (**H**–**N**) Using *dome*^*MESO*^*-Gal4, UAS-EGFP* driver by expressing *CSN2*^*RNAi*^ (**I**), *CSN3*^*RNAi*^ (**J**), *CSN4*^*RNAi*^ (**K**), *CSN6*^*RNAi*^ (**L**), *CSN7*^*RNAi*^ (**M**), and *CSN8*^*RNAi*^ (**N**) caused reduced plasmatocyte number marked by P1 (magenta) compared to the control lymph gland (*dome*^*MESO*^*-Gal4, UAS-EGFP/+*) (**H**). The quantification of the Plasmatocyte index is shown in Fig. 1O. (**O**) Graph showing the lymph gland size in terms of DAPI volume (μm^3^) in Figs. 1D–F,J–L, and  EV1A–N. Upon progenitor-specific (*dome*^*MESO*^*-Gal4, UAS-2XEGFP*) knockdown of *CSN* subunits by expressing *CSN1b*^*RNAi*^ (*n* = 13, *P* > 0.9999), *CSN2*^*RNAi*^ (*n* = 18, *P* = 0.8995), *CSN3*^*RNAi*^ (*n* = 16, *P* = 0.6736), *CSN4*^*RNAi*^ (*n* = 16, *P* = 0.4598), *CSN5*^*RNAi*^ (*n* = 20, *P* = 0.4035), *CSN6*^*RNAi*^ (*n* = 19, *P* = 0.8429), *CSN7*^*RNAi*^ (*n* = 18, *P* = 0.9329), and *CSN8*^*RNAi*^ (*n* = 13, *P* = 0.4753) is similar when compared to the wild-type lymph gland *w*^*1118*^ (*n* = 15). (**P**) Progenitor index in (Figs. 1D–F,J–L, and  EV1A–N). *w*^*1118*^ (*n* = 27), *CSN1b*^*RNAi*^ (*n* = 31, *P* = 0.9866), *CSN2*^*RNAi*^ (*n* = 19, *P* = 0.1888), *CSN3*^*RNAi*^ (*n* = 20, *P* = 0.4614), *CSN4*^*RNAi*^ (*n* = 15, *P* > 0.9999), *CSN5*^*RNAi*^ (*n* = 16, *P* > 0.9999), *CSN6*^*RNAi*^ (*n* = 16, *P* = 0.5798), *CSN7*^*RNAi*^ (*n* = 18, *P* = 0.9878) and *CSN8*^*RNAi*^ (*n* = 16, *P* = 0.9793). (**Q**) Quantitative RT-PCR showing significant reduction in the transcript level of CSNs in respective *CSN*^*RNAi*^ background when expressed using pan-lymph gland Gal4 driver *e33c-Gal4* (*e33c-Gal4 UAS-CSN1b*^*RNAi*^) (BL 27303) (*n* = 3, *P* < 0.0001), *e33c-Gal4 UAS-CSN2*^*RNA*i^ (BL 28908) (*n* = 3, *P* = 0.0004), *e33c-Gal4, UAS-CSN3*^*RNA*i^ (BL 33369) (*n* = 3, *P* < 0.0001), *e33c-Gal4 UAS-CSN4*^*RNA*i^ (BL 42798) (*n* = 3, *P* < 0.0001), *e33c-Gal4 UAS-CSN5*^*RNA*i^ (BL 28732) (*n* = 3, *P* < 0.0001), *e33c-Gal4 UAS-CSN6*^*RNA*i^ (BL 36073) (*n *= 3, *P* < 0.0001), *e33c-Gal4 UAS-CSN7*^*RNA*i^ (BL 33663) (*n* = 3, *P *< 0.0001)and *e33c-Gal4 UAS-CSN8*^*RNA*i^(v50565) (*n* = 3, *P* < 0.0001) when compared to control (*e33c-Gal4*). (**R**–**T**) Knockdown of CSN in lymph gland core progenitors (green) using *Tep4-Gal4, UAS-2XEGFP* driver by expressing *CSN1b*^*RNA*i^ (**S**) and *CSN5*^*RNA*i^ (**T**) does not show any significant difference in the plasmatocyte numbers marked by anti-P1 (magenta) compared to control (*Tep4-Gal4, UAS-EGFP/+*) (**R**). (**U**) Plasmatocyte index in (**R**–**T**): *w*^*1118*^ (*n* = 10), *CSN1b*^*RNAi*^ (*n* = 17, *P *= 0.4335), *CSN5*^*RNAi*^ (*n* = 12, *P* = 0.8359). (**V**) Core progenitor index in (Figs. 1IZA–ZC and EV1R–T). *w*^*1118*^ (*n* = 21), *CSN1b*^*RNAi*^ (*n* = 17, *P* = 0.3448), *CSN5*^*RNAi*^ (*n* = 12, *P* = 0.934). (**W**–**Y**) Knockdown of CSN using a differentiated zone driver *Hml*^*Δ*^*-Gal4, UAS-EGFP* by expressing *CSN1b*^*RNAi*^ (**X**), and *CSN5*^*RNAi*^ (**Y**) resulted in a drastic decrease in plasmatocyte numbers (magenta) as compared to control (**W**). (**Z**) Plasmatocyte index in (W-Y): *w*^*1118*^ (*n* = 25), *CSN1b*^*RNAi*^ (*n* = 18, *P* < 0.0001), *CSN5*^*RNAi*^ (*n* = 19, *P* < 0.0001). All confocal images represent maximum-intensity projections of the middle-third optical sections of the wandering third-instar anterior lymph gland lobe from at least three independent biological experiments. White dashed lines demarcate one lymph gland lobe. Scale bar, 25 μm. Error bars in graphs: mean ± S.D. n.s.: not significant (*P* ≥ 0.05); ***P < 0.001; ****P < 0.0001. A one-way ANOVA with Tukey’s post-hoc test was used for analysis involving (**O**, **P**, **U**, **V**, **Z**). A two-tailed, unpaired Student’s *t* test was employed to compare the mean value of the two groups in (**Q**). Source data are available online for this figure.

Genome-wide RNAi screens for genes involved in *Drosophila* hematopoiesis reported that depletion of CSN subunits causes larval blood phenotype (Avet-Rochex et al, 2010; Evans et al, 2021). Multiple studies have established a direct connection between CSN and DDR across several model systems (Fuzesi-Levi et al, 2014; Hannss and Dubiel, 2011; Meir et al, 2015; Wang et al, 2021). Recently, we reported the DDR is associated with plasmatocyte differentiation (Maurya et al, 2024), which led us to examine whether CSN plays a role in *Drosophila* blood cell fate decisions. To investigate this, we depleted each of the eight CSN subunits (CSN1b to CSN8) using multiple RNAi transgenic lines with several Gal4 drivers across different cell types of the developing lymph gland (Fig. 1B,C). We first used the *dome*^*MESO*^*-Gal4* driver, which is strongly expressed in the lymph gland’s core progenitors but at low levels in intermediate progenitors (also known as distal progenitors or differentiating cells) (Cho et al, 2024; Girard et al, 2021; Goins et al, 2024). Knocking down any one of the eight CSN (*CSN1b*^*RNAi*^ to *CSN8*^*RNAi*^) in the lymph gland progenitors (*dome*^*MESO*^ > *EGFP* > *CSN*^*RNAi*^) caused a significant increase in the number of crystal cells marked by the expression of transcription factor Hindsight (Hnt; also called pebbled) in wandering third instar larval lymph glands (Figs. 1D–F,I and EV1A–G), indicating that during normal development, these CSN subunits work together as a holoenzyme to suppress crystal cell formation. We next examined whether CSN depletion also affects the differentiation of plasmatocytes, which comprise the majority of total blood cells. Interestingly, CSN-depletion in the lymph gland progenitors (*CSN1b*^*RNAi*^ to *CSN8*^*RNAi*^) showed a significant decrease in plasmatocyte numbers marked by P1 (also called nimC1) (Kurucz et al, 2007) (Figs. 1J–L,O and EV1H–N) without any substantial change in lymph gland size in terms of DAPI-stained nuclei volume (Fig. EV1O) and dome^MESO^ > GFP+ progenitors index (Fig. EV1P). Lamellocytes are identified by Myospheroid (Mys, a βPS integrin) (Evans et al, 2022; Madhwal et al, 2020) immunostaining; however, CSN-depleted lymph gland progenitors did not cause ectopic lamellocyte differentiation (Appendix Fig. S1A–E).

Our data show that the number of crystal cells in wandering third-instar lymph glands is similar across various *Gal4* drivers and control genotypes used in *Drosophila* hematopoiesis research (Appendix Fig. S1F), consistent with the existing literature (Blanco-Obregon et al, 2020; Cho et al, 2018; Girard et al, 2021; Yoon et al, 2017). To ensure that these phenotypes are indeed due to CSN depletion, we tested multiple CSN RNAi lines available at the Bloomington Stock Center, USA (see Reagents and Tools table for details). Additionally, we measured RNAi efficiency using quantitative RT-PCR to assess the downregulation of target CSN subunits by the *e33c-Gal4* driver in the entire lymph gland (Fig. EV1Q). Finally, the increased crystal cell and decreased plasmatocyte phenotypes, induced by CSN5RNAi expression in lymph gland progenitors, were rescued by coexpression of wild-type CSN5 (Fig. 1D,F–I,J,L–O). We also examined other crystal cell markers in differentiated cells following depletion of the CSN1b and CSN5 subunits. These CSN subunits were selected because the CSN5 subunit is the catalytic domain of the CSN holoenzyme, while CSN1b is one of the subunits of the non-catalytic region. Remarkably, depletion of any of these CSN subunits (*CSN1b*^*RNAi*^ and *CSN5*^*RNAi*^) in lymph gland progenitors also caused a significant increase in Lozenge (Lz) positive cells, an early marker for crystal cell (Appendix Fig. S1G–K), and prophenoloxidase 2 (PPO2) (Shin et al, 2024), a mature crystal cell marker (Appendix Fig. S1L–P). These data suggest that during normal lymph gland development, CSN activity in progenitors decides their appropriate cell fate.

### CSN does not affect the core progenitor properties during lymph gland development

We next investigated when CSN knockdown affects crystal cell numbers. To monitor this, we measured the number of crystal cells of CSN1b-depleted lymph glands taken from synchronized larvae (Maurya et al, 2024; Mondal et al, 2011). The Hnt+ crystal cells are not detectable at 36 h After Larval Hatching (ALH), but by 42 h ALH, they begin to differentiate, and the number of crystal cells is comparable between the control and CSN-depleted lymph glands (Fig. 1P,Q,U,V,Z). However, the number of crystal cells began to increase by 48 h ALH in CSN-depleted lymph glands and remained elevated at 60 h ALH and 72 h ALH compared with controls (Fig. 1R–T,W–Z), implying that CSN’s activity initiates during the differentiation process at the early third instar stage.

Recent studies have reported that third-instar lymph gland progenitors are mainly arrested in the G2 phase of the cell cycle (Cho et al, 2024; Goins et al, 2024; Kapoor et al, 2022). We observed that CSN-depleted lymph gland progenitors did not show any change in cell cycle status using the Fluorescence Ubiquitin Cell Cycle Indicator (FUCCI) transgenic fly line (Zielke et al, 2014) and anti-phospho-Histone H3 (pH3) immunostaining (Appendix Fig. S2A–I), indicating that the cell cycle and proliferation status of the CSN-depleted lymph gland progenitors remain unaltered. We further tested whether other known progenitor cell markers (e.g., E-cadherin) and signaling (e.g., Hh) are active in the CSN-depleted lymph gland (Gao et al, 2013; Mandal et al, 2007; Maurya et al, 2024; Mondal et al, 2011; Sharma et al, 2019). CSN-depleted lymph gland progenitors did not show any significant change in the staining intensity of E-cadherin or Ci (a Hh signaling downstream transcription factor) (Appendix Fig. S2J–R), indicating that the lymph gland progenitor population is unaffected.

**Figure EV2:**
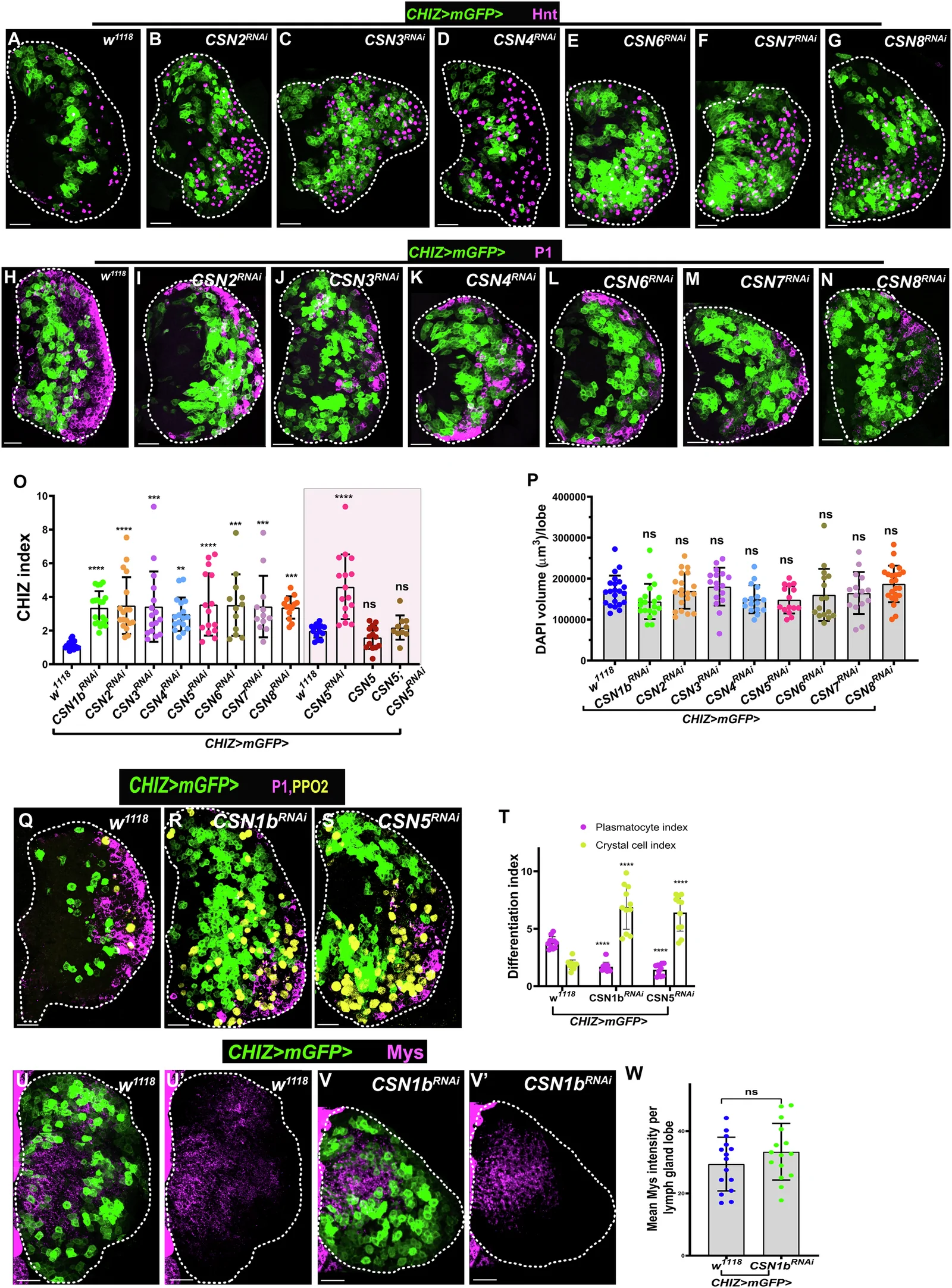
CSN is required during the myeloid-type intermediate progenitor cell fate decision, related to Fig. 2. (**A**–**G**) Knockdown of CSN complex in lymph gland progenitors (green) using *CHIZ-Gal4, UAS-mGFP* driver by expressing *CSN2*^*RNAi*^ (**B**), *CSN3*^*RNAi*^ (**C**), *CSN4*^*RNAi*^ (**D**), *CSN6*^*RNAi*^ (**E**), *CSN7*^*RNAi*^ (**F**) and *CSN8*^*RNAi*^ (**G**), increases the Hnt positive (magenta) crystal cell number compared to control *w*^*1118*^ (**A**). (**H**–**N**) Using *CHIZ-Gal4, UAS-mGFP* driver by expressing *CSN2*^*RNAi*^ (*n* = 18, *P* < 0.0001) (**I**), *CSN3*^*RNAi*^ (**J**) *CSN4*^*RNAi*^ (**K**), *CSN6*^*RNAi*^ (**L**), *CSN7*^*RNAi*^ (**M**) and *CSN8*^*RNAi*^ (**N**) caused reduced plasmatocytes number marked by P1 (magenta) compared to control lymph gland (*CHIZ-Gal4, UAS-mGFP/+*) (**H**). (**O**) Intermediate progenitors index in (**B**–**J**): *w*^*1118*^ (*n* = 21), *CSN1b*^*RNAi*^ (*n* = 19, *P* < 0.0001), *CSN2*^*RNAi*^ (*n* = 18, *P* < 0.0001), *CSN3*^*RNAi*^ (*n* = 16, *P* = 0.0001), *CSN4*^*RNAi*^ (*n* = 17, *P* = 0.0039), *CSN5*^*RNAi*^ (*n* = 15, *P* < 0.0001), *CSN6*^*RNAi*^ (*n* = 12, *P* = 0.0003), *CSN7*^*RNAi*^ (*n* = 12, *P* = 0.0006) and *CSN8*^*RNAi*^ (*n* = 13, *P* = 0.0006). *w*^*1118*^ (*n* = 14), *CSN5*^*RNAi*^ (*n* = 16, *P* < 0.0001), *CSN5* (*n* = 14, *P* > 0.812), and *CSN5*;*CSN5*^*RNAi*^ (*n* = 10, *P* = 0.9758). (**P**) Graph showing the lymph gland size in terms of DAPI volume (μm^3^) upon intermediate progenitor-specific knockdown of CSN, shown in Figs. 2A–C,G–I and  EV2A–N. The lymph gland size upon expression of *CSN1b*^*RNAi*^ (*n* = 19, *P* = 0.6699), *CSN2*^*RNAi*^ (*n* = 20, *P* > 0.9999), *CSN3*^*RNAi*^ (*n *= 17, *P* = 0.998); *CSN4*^*RNAi*^ (*n* = 16, *P* = 0.9078), *CSN5*^*RNAi*^ (*n* = 15, *P* = 0.8868), *CSN6*^*RNAi*^ (*n* = 16, *P* = 0.9994), *CSN7*^*RNAi*^ (*n* = 15, *P* > 0.9999), and *CSN8*^*RNAi*^ (*n* = 23, *P* = 0.9263) is nearly similar when compared to the wild-type lymph gland *w*^*1118*^ (*n* = 22). (**Q**–**S**) Knockdown of CSN complex in lymph gland progenitors (green) using *CHIZ-Gal4, UAS-mGFP* driver by expressing *CSN1b*^*RNAi*^ (**R**), and *CSN5*^*RNAi*^ (**S**), increases the PPO2 positive (yellow) crystal cells and decreases P1 positive (magenta) plasmatocytes compared to control *w*^*1118*^ (**Q**). (**T**) Plasmatocyte and crystal cell index in (**Q**–**S**). *CSN1b*^*RNAi*^ (*n* = 11, *P* < 0.0001), *CSN5*^*RNAi*^ (*n* = 11, *P* < 0.0001) and *w*^*1118*^ (*n* = 11). (**U**–**V’**) Intermediate progenitor-specific knockdown of *CSN1b* (*CHIZ-Gal4 UAS-mGFP; CSN1b*^*RNAi*^) (**V**–**V’**) does not induce any lamellocyte differentiation and looks similar to the wild-type lymph gland (*CHIZ-Gal4 UAS-mGFP*) (**U**–**U’**). (**W**) Mean Mys (lamellocyte marker) fluorescent intensity per lymph gland lobe in (**U**–**V’**). *CSN1b*^*RNAi*^ (*n* = 14, *P* = 0.2305) and *w*^*1118*^ (*n* = 15). All confocal images represent maximum-intensity projections of the middle-third optical sections of the wandering third-instar anterior lymph gland lobe from at least three independent biological experiments. White dashed lines demarcate one lymph gland lobe. Scale bar, 25 μm. Each data point represents the number of lymph gland lobes analyzed. Error bars in graphs: mean ± S.D. n.s.: not significant (*P* ≥ 0.05); ****P < 0.0001. A one-way ANOVA with Tukey’s post-hoc test was used for analysis involving (**O**, **P**). A two-way ANOVA with Tukey’s post-hoc test was used for analysis involving (**T**). A two-tailed, unpaired Student’s *t* test was employed to compare the mean value of the two groups in (**W**). Source data are available online for this figure.

During the early larval stages, the blood progenitors in the inner core of the lymph gland undergo rapid proliferation. At the mid-second instar stage, cells cease proliferating and differentiate into mature blood cells through the intermediate progenitor stage at the outer boundary’s distal borders of the lymph gland (Cho et al, 2024; Girard et al, 2021; Goins et al, 2024). Several studies reported that the *Tep4-Gal4 UAS-EGFP* driver is expressed in the lymph gland core progenitors (Girard et al, 2021; Goins et al, 2024). Thus, to test whether loss of CSN in the core progenitor caused a similar phenotype as in *dome*^*MESO*^*-Gal4* driven *CSN*^*RNAi*^, we depleted CSN (*CSN1b*^*RNAi*^ *and* *CSN5*^*RNAi*^) in the core progenitor (*Tep4* > *EGFP* > *CSN*^*RNAi*^) during lymph gland development. Remarkably, loss of CSN in the lymph gland core progenitors did not cause a significant change in crystal cells (Fig. 1ZA–ZD), plasmatocytes (Fig. EV2R–U), and core progenitor population in the lymph gland (Fig. EV2V). However, CSN-depletion (*CSN1b*^*RNAi*^ and *CSN5*^*RNAi*^) using the *Hml*^*Δ*^*-Gal4* driver, which began to express in intermediate progenitors and also in differentiated plasmatocytes of the lymph gland (Girard et al, 2021; Spratford et al, 2021), caused a significant increase in crystal cells (Fig. 1ZE–ZH) and a significant decrease in plasmatocytes (Fig. EV1W–Z). These results suggest that CSN does not alter the properties of core progenitors but rather prevents intermediate progenitors from differentiating into crystal cells. Consequently, the absence of CSN causes a rise in the number of crystal cells.

### CSN affects myeloid-type intermediate progenitor cell fate decision

**Figure 2:**
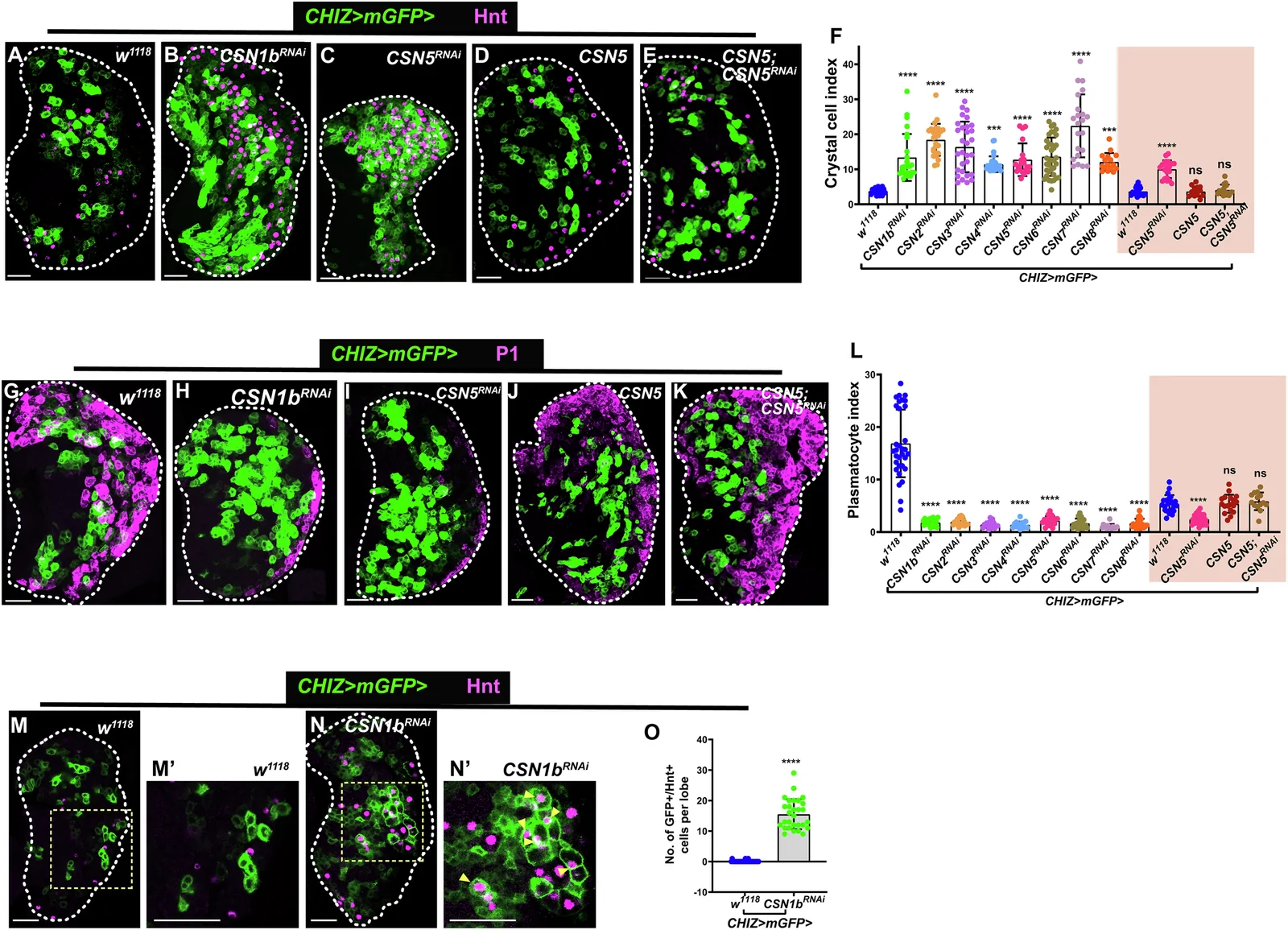
CSN is required during the myeloid-type intermediate progenitor cell fate decision. (**A**–**E**) Knockdown of CSN in Intermediate progenitors (green) using *CHIZ-Gal4, UAS-mCD8::GFP* by expressing *CSN1b*^*RNAi*^ (**B**), and *CSN5*^*RNAi*^ (**C**) increases the crystal cell number (magenta) and CHIZ-positive cells (green) as compared to control *w*^*1118*^ (**A**). The increased crystal cell phenotype in *CSN5*^*RNAi*^ is rescued by overexpression of *CSN5* in *CSN5*^*RNAi*^ background (**E**). Overexpression of CSN5 does not alter crystal cell number (**D**). (**F**) Crystal cell index in (**A**–**E**). *w*^*1118*^ (*n* = 20), *CSN1b*^*RNAi*^ (*n* = 25, *P* < 0.0001), *CSN2*^*RNAi*^ (*n* = 26, *P* < 0.0001), *CSN3*^*RNAi*^ (*n* = 30, *P* < 0.0001), *CSN4*^*RNAi*^ (*n* = 28, *P* = 0.0001), *CSN5*^*RNAi*^ (*n* = 20, *P* < 0.0001), *CSN6*^*RNAi*^ (*n* = 36, *P* < 0.0001) (**E**), *CSN7*^*RNAi*^ (*n* = 21, *P* < 0.0001), and *CSN8*^*RNAi*^ (*n* = 16, *P* = 0.0004). *w*^*1118*^ (*n* = 17), *CSN5*^*RNAi*^ (*n* = 20, *P* < 0.0001), *CSN5* (*n *= 15, *P* > 0.9999), and *CSN5*;*CSN5*^*RNAi*^ (*n* = 17, *P* = 0.8993). (**G**–**K**) Knockdown of CSN in lymph gland Intermediate progenitors (green) using *CHIZ-Gal4, UAS-mGFP* driver by expressing *CSN1b*^*RNAi*^ (**H**), and *CSN5*^*RNAi*^ (**I**), reduces the plasmatocyte number marked by P1 (magenta) compared to control (*CHIZ-Gal4, UAS-mGFP/+*) (**G**). Overexpression of CSN5 in the background of *CSN5*^*RNAi*^ in the progenitor rescued the *CSN5*^*RNAi*^-mediated decrease in plasmatocyte number (**K**); only overexpression of CSN5 does not alter the amount of (**J**). (**L**) Plasmatocyte index in (**G**–**K**). *w*^*1118*^ (*n* = 32), *CSN1b*^*RNAi*^ (*n* = 19, *P *< 0.0001), *CSN2*^*RNAi*^ (*n* = 19, *P* < 0.0001), *CSN3*^*RNAi*^ (*n* = 18, *P* < 0.0001), *CSN4*^*RNAi*^ (*n* = 17, *P *< 0.0001), *CSN5*^*RNAi*^ (*n* = 19, *P* < 0.0001), *CSN6*^*RNAi*^ (*n* = 17, *P* < 0.0001), *CSN7*^*RNAi*^ (*n* = 15, *P* < 0.0001), and *CSN8*^*RNAi*^ (*n* = 23, *P* < 0.0001). *w*^*1118*^ (*n* = 22), *CSN5*^*RNAi*^ (*n* = 25, *P* < 0.0001), *CSN5* (*n* = 18, *P* = 0.812), and *CSN5*;*CSN5*^*RNAi*^ (*n* = 14, *P* = 0.9758). (**M**–**N’**) Intermediate progenitor-specific knockdown of *CSN1b* (**N**–**N’**) caused many Hnt+ cells to colocalize with the *CHIZ*+ cells (green) when compared to the control lymph gland (**M**–**M’**). (**O**) Quantification of Hnt+ cells colocalizing with CHIZ+ cells per lymph gland lobe in (**M**–**N’**). *CSN1b*^*RNAi*^ (*n* = 29, *P* < 0.0001) and *w*^*1118*^ (*n* = 30). All confocal images represent maximum intensity projections of the middle third optical sections, except images (**M**–**N’**), the single optical section of the wandering third instar lymph gland lobe from at least three independent biological experiments. White dashed lines demarcate one lymph gland lobe. Scale bar, 25 μm. Each data point represents the number of lymph gland lobes analyzed. Error bars in graphs: mean ± S.D. n.s.: not significant (*P* ≥ 0.05); **P < 0.01; ***P < 0.001; ****P < 0.0001. One-way ANOVA with Tukey’s post-hoc test was used for analysis involving (**F**, **L**). A two-tailed, unpaired Student’s *t* test was employed to compare the mean value of the two groups in (**O**). Detailed genotypes are in Appendix Table S1. Source data are available online for this figure.

**Figure 3:**
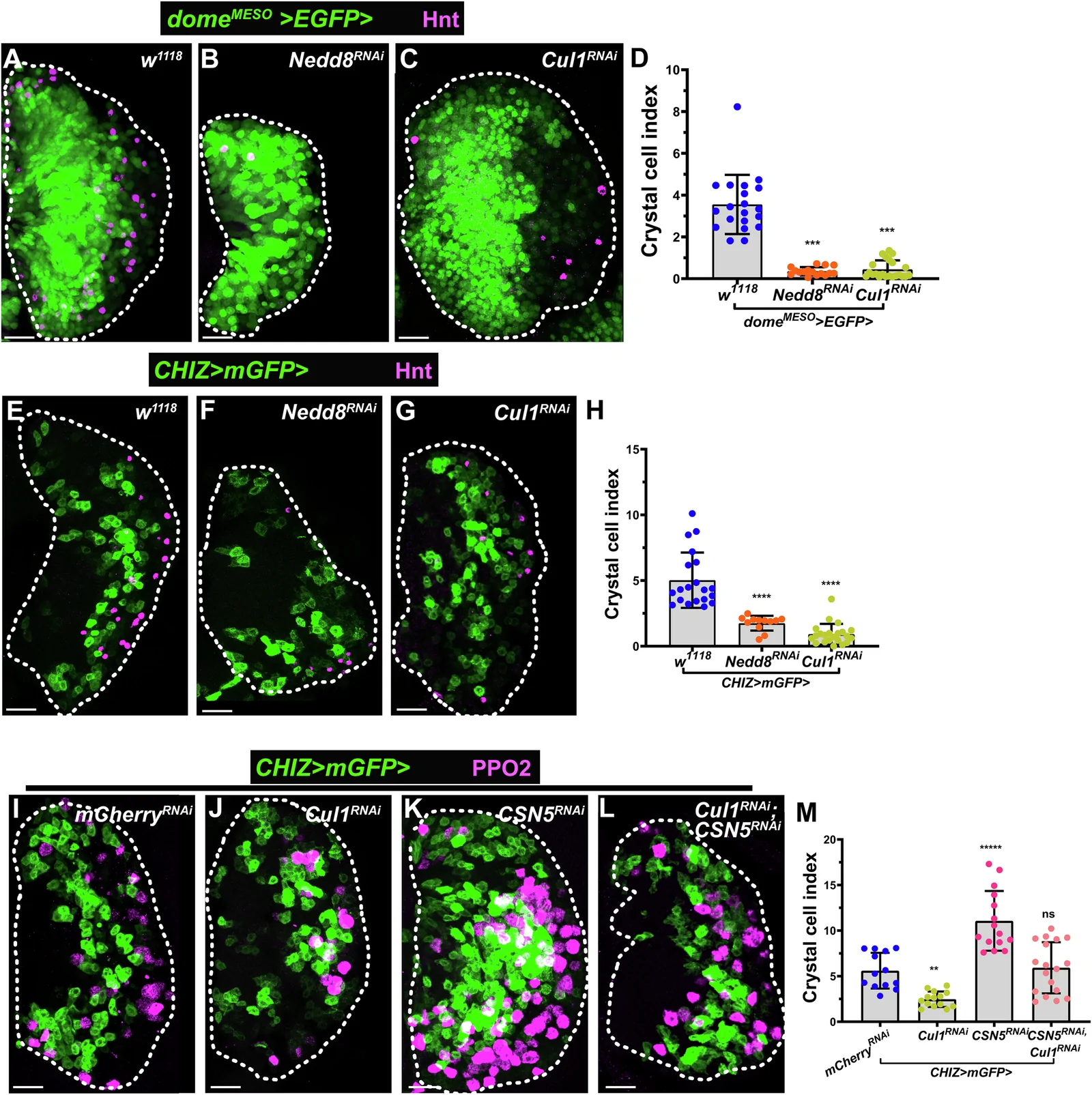
CSN-mediated Cullin-1 is involved in progenitor cell fate decision. (**A**–**C**) Knockdown of *Nedd8* and *Cul1* in progenitors (green) using *dome*^*MESO*^*-Gal4, UAS-EGFP* driver expressing *Nedd8*^*RNAi*^ (**B**) and *Cul1*^*RNAi*^ (**C**) leads to a decrease in crystal cells (magenta) compared to the control (**A**). (**D**) Crystal cell index in (**A**–**C**). *Nedd8*^*RNAi*^ (*n* = 16, *P* = 0.0005), *Cul1*^*RNAi*^ (*n* = 22, *P* = 0.0002), and *w*^*1118*^ (*n* = 20). (**E**–**G**) Knockdown of *Nedd8* and *Cul1* in intermediate progenitor (green) using *CHIZ-Gal4, UAS-mCD8GFP* by expressing *Nedd8*^*RNAi*^ (**F**) and *Cul1*^*RNAi*^ (**G**) decreases the number of crystal cells (magenta) compared to the control (**E**). (**H**) Crystal cell index in (**E**–**G**). *Nedd8*^*RNAi*^ (*n* = 12, *P* < 0.0001), *Cul1*^*RNAi*^ (*n* = 22, *P* < 0.0001), and *w*^*1118*^ (*n* = 20). (**I**–**L**) Depletion of Cul1 in the intermediate progenitor (green) decreased the crystal cells (magenta) (**I**) compared to control (**J**). Knockdown of CSN5 (**K**) resulted in increased crystal cells (magenta). Depletion of CSN5 in the background of *Cul1*^*RNAi*^ in the intermediate progenitor rescued *CSN5*^*RNAi*^-mediated increase in crystal cell number (**L**). (**M**) Crystal cell index in (**I**–**L**). *Cul1*^*RNAi*^ (*n* = 14, *P* = 0.009), *CSN5*^*RNAi*^ (*n* = 15, *P* < 0.0001), *Cul1*^*RNAi*^*; CSN5*^*RNAi*^ (*n* = 18, *P* = 0.9841) and *mCherry*^*RNAi*^ (*n* = 13). All confocal images represent maximum-intensity projections of the middle third optical sections of the wandering third-instar lymph gland lobe from at least three independent biological experiments. Scale bar, 25 μm. Each data point represents the number of lymph gland lobes analyzed. Error bars in graphs: mean  ± S.D. n.s.: not significant (*P* ≥ 0.05); **P < 0.01; ***P < 0.001; ****P < 0.0001. One-way ANOVA with Tukey’s post-hoc test was used for analysis involving (**D**, **H**, **M**). Detailed genotypes are in Appendix Table S1. Source data are available online for this figure.

We found that depleting CSN enhances crystal cell differentiation only when it occurs in progenitors (*dome*^*MESO*^*-Gal4*) or differentiating cells (*Hml*^*Δ*^*-Gal4*), but not in core progenitors (*Tep4-Gal4*). Consequently, we examined the lymph gland phenotype upon CSN depletion specifically in the intermediate progenitor zone, using the *CHIZ-Gal4* driver (Maurya et al, 2024; Spratford et al, 2021). This driver is expressed solely in intermediate progenitors, not in differentiated plasmatocytes or crystal cells (Fig. 1B,C) (Spratford et al, 2021). Thus, the *CHIZ-Gal4* driver helps us determine when plasmatocytes or crystal cells differentiate from the intermediate progenitor better than the *Hml*^*Δ*^*-Gal4* driver. The lymph glands in each CSN subunit-depleted in intermediate progenitor backgrounds showed a significantly higher number of crystal cells (Figs. 2A–C,F and EV2A–G,Q–T) and also remarkably higher numbers of CHIZ>mGFP+ cells (Fig. EV2O). However, the number of plasmatocytes decreased significantly (Figs. 2G–I,L and EV2H–N,Q–T), and the size of the lymph glands was unaffected (Fig. EV2P). The enhanced numbers of crystal cells and low numbers of plasmatocytes in the lymph gland with CSN5-depleted intermediate progenitors (*CHIZ* > *mGFP* > *CSN5*^*RNAi*^) were rescued by expressing CSN5 (*CHIZ* > *mGFP* > *CSN5; CSN5*^*RNAi*^) (Fig. 3A,C–F,G,I–L). The high number of crystal cell phenotype results was further confirmed using a mature crystal cell reporter line, BcF6-RedStinger, which labeled PPO1 [also known as Black cells (Bc)] (Tokusumi et al, 2009). We found that BcF6-RedStinger+ cells were significantly higher in the lymph gland with CSN-depleted (*CSN1b*^*RNAi*^, *CSN5*^*RNAi*^, and *CSN6*^*RNAi*^) intermediate progenitors (Appendix Fig. S3A–E). Additionally, CSN depletion using *Nplp2-Gal4* (another intermediate progenitor Gal4 driver) (Cho et al, 2024) causes a dramatic increase in crystal cell numbers in the lymph gland, similar to *CHIZ-Gal4* (Appendix Fig. S3F–J). We also observed that crystal cells (both early markers Hnt or Lz and mature marker PPO2) usually do not colocalize with CHIZ>mGFP+ cells in controls (Fig. 2M–M’,O; Appendix Fig. S3K,M,N,P). Interestingly, we observed that 20% of Hnt + , 18% of Lz + , and 10% of PPO2+ crystal cells were CHIZ>mGFP+ in *CHIZ* > *mGFP* driver-mediated CSN-depleted lymph glands (Fig. 2N–N’,O; Appendix Fig. S3L,M,O,P), indicating a bias towards crystal cell fate in CSN-depleted CHIZ>mGFP+ cells. However, no lamellocyte differentiation was found in the lymph glands with CSN-depleted intermediate progenitors (Fig. EV2U–W). These data suggest that CSN functions primarily during cell fate decisions in lymph gland intermediate progenitors.

### CSN modulates Cullin-1 complex activity crucial for blood progenitor cell fate decisions

**Figure EV3:**
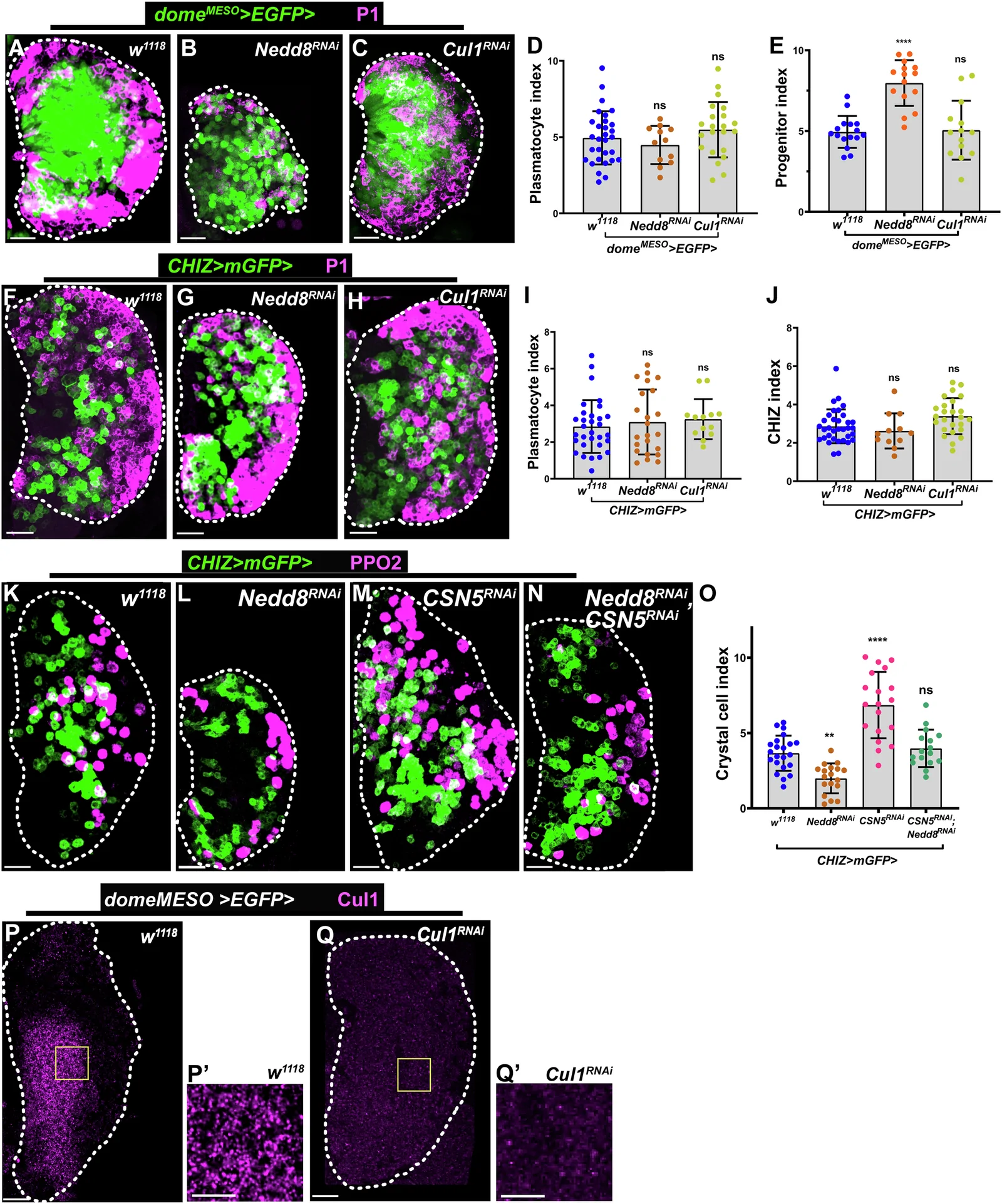
Involvement of Cullin-1 in CSN-mediated cell fate decision in the lymph gland, related to Fig. 3. (**A**–**C**) Knockdown of Nedd8 and Cullin-1 in lymph gland progenitors (green) using *dome*^*MESO*^*-Gal4, UAS-EGFP* driver by expressing *Nedd8*^*RNAi*^ (**B**) and *Cul1*^*RNAi*^ (**C**) shows plasmatocytes marked by P1(magenta) compared to control (*dome*^*MESO*^*-Gal4 UAS-EGFP/+*) (**A**). (**D**) Plasmatocyte index in (**A**–**C**). *w*^*1118*^ (*n* = 31), *Nedd8*^*RNAi*^ (*n* = 12, *P* = 0.6998), and *Cul1*^*RNAi*^ (*n* = 22, *P* = 0.4967). (**E**) Progenitor index in (**A**–**C**). *w*^*1118*^ (*n* = 16), *Nedd8*^*RNAi*^ (*n* = 15, *P* < 0.0001), and *Cul1*^*RNAi*^ (*n* = 14, *P* = 0.9766). (**F**–**H**) Knockdown of Nedd8 and Cullin-1 in lymph gland intermediate progenitors (green) using *CHIZ-Gal4, UAS-mGFP* driver by expressing *Nedd8*^*RNAi*^ (**G**), and *Cul1*^*RNAi*^ (**H**) resulted in a similar number of plasmatocytes marked by P1 (magenta) compared to control (*CHIZ-Gal4, UAS-mGFP/+*) (**F**). (**I**) Plasmatocyte index in (**F**–**H**). *w*^*1118*^ (*n* = 31), *Nedd8*^*RNAi*^ (*n* = 23, *P* = 0.8285), and *Cul1*^*RNAi*^ (*n* = 13, *P* = 0.703). (**J**) CHIZ index in (**F**–**H**): *w*^*1118*^ (*n* = 35), *Nedd8*^*RNAi*^ (*n* = 15, *P* = 0.7161), and *Cul1*^*RNAi*^ (*n* = 25, *P* = 0.0751). (**K**–**N**) Depletion of Nedd8 in the *CHIZ*^*+*^ progenitor (green) decreased the number of crystal cells (magenta) (**L**) compared to control (**K**). Depletion of CSN5 (**M**) in the background of *Nedd8*^*RNAi*^ in the intermediate progenitor rescued *CSN5*^*RNAi*^-mediated increase in crystal cell number (**N**). (**O**) Crystal cell index in (**K**–**N**). *w*^*1118*^ (*n* = 22), *Nedd8*^*RNAi*^ (*n* = 19, *P* = 0.0037), *CSN5*^*RNAi*^ (*n* = 19, *P* < 0.0001), and *Nedd8*^*RNAi*^*; CSN5*^*RNAi*^ (*n* = 16, *P* = 0.9165). (**P**–**Q’**) Decreased Cul1 staining in the lymph gland (*dome*^*MESO*^*>Cul1*^*RNAi*^) (**Q**–**Q’**) compared to control (*dome*^*MESO*^*-Gal4 UAS-EGFP/+*) (**P**–**P’**) using anti-Cul1 immunostaining. All confocal images represent maximum-intensity projections of the middle-third optical sections of the wandering third-instar lymph gland lobe from at least three independent biological experiments. Scale bar, 25 μm, except (**P’**, **Q’**), where the scale bar is 10 μm and is a single optical section. Each data point represents the number of lymph gland lobes analyzed. Error bars in graphs: mean ± S.D. n.s.: not significant (*P* ≥ 0.05); **P < 0.01; and ****P < 0.0001. A one-way ANOVA with Tukey’s post-hoc test was used for analysis involving (**D**, **E**, **I**, **J**, **O**). Source data are available online for this figure.

The CSN is crucial for cellular regulation as it removes Nedd8 (deneddylate) from the Cullin RING ligase (CRL) complex, inactivating it and halting substrate degradation (Schulze-Niemand and Naumann, 2023; Wei et al, 2008). While Nedd8 attachment activates the CRL complex, CSN’s role in deneddylation is essential for regulating CRL activity and enabling the exchange of substrate receptors. In our study of Nedd8-depleted progenitors (*dome*^*MESO*^ > *EGFP>Nedd8*^*RNAi*^) and intermediate progenitors (*CHIZ* > *mGFP> Nedd8*^*RNAi*^*)*, we found a significant decrease in crystal cells (Fig. 3A,B,D–F,H). Plasmatocytes index (Fig. EV3A,B,D,F,G,I) and CHIZ index remain unchanged (Fig. EV3J); however, the progenitor index increased (Fig. EV3E) because the lymph gland was smaller than control (Figs. 3A,B and EV3A,B). A rescue experiment with simultaneous downregulation of Nedd8 and CSN5 (*CHIZ* > *mGFP>Nedd8*^*RNAi*^; *CSN5*^*RNAi*^) showed that crystal cell numbers became similar to controls (Fig. EV3K–O). These findings highlight the critical role of Nedd8 in the differentiation of plasmatocytes and crystal cells, indicating that Nedd8 acts in an antagonistic manner to CSN.

Our RNAi screen targeting *Drosophila* cullins identified Cul1 as the most potent regulator of CSN-mediated blood cell differentiation. Knockdown of Cul1 (tested with two *Cul1*^*RNAi*^ lines) caused a significant reduction in crystal cells (Fig. 3A,C,D), a phenotype opposite to that observed upon CSN depletion. We also observed significantly decreased Cul1 immunostaining in the lymph gland (*dome*^*MESO*^*>Cul1*^*RNAi*^) using a prevalidated anti-Cul1 antibody (Fig. EV3P–Q’) (Sato et al, 2019). However, Cul1 depletion in progenitors did not significantly alter the progenitor and plasmatocyte indices (Fig. EV3A,C–E). We then tested the function of Cul1 in intermediate progenitors by knocking down Cul1 with *CHIZ-Gal4*, which reduced the number of crystal cells (Fig. 3E,G,H), but the plasmatocyte and CHIZ indices remained similar to control (Fig. EV3F,H–J). Remarkably, the high number of crystal cells in intermediate progenitors with depleted CSN5 was rescued by *Cul1*^*RNAi*^ (Fig. 3I–M), indicating that CSN-mediated deneddylation of Cul1 regulates the lymph gland’s cell fate decision. We also found no significant change in the index of TUNEL-positive cells and pH3+ cells in the lymph glands of *Nedd8*^*RNAi*^ and *Cul*^*RNAi*^ backgrounds (Appendix Fig. S4A–P), indicating no alteration in cell proliferation or cell death in these backgrounds. These data indicate that CSN modulates the activity of the Cul1 complex, impacting cell fate during lymph gland development.

### CSN modulates Neuralized/Serrate-mediated Notch activation in developing lymph gland cells

**Figure 4:**
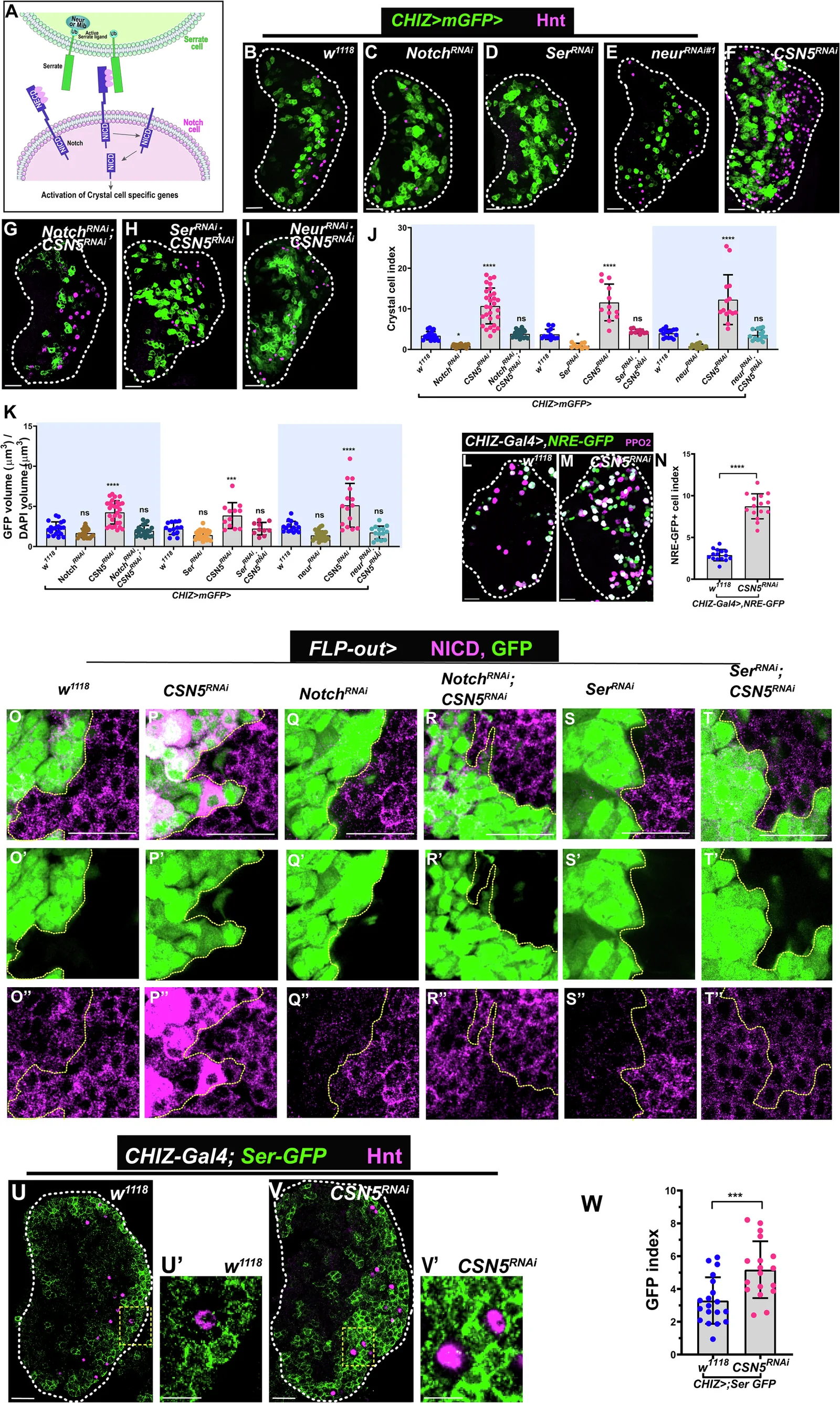
CSN modulates Neuralized/Serrate-mediated Notch activity in developing lymph gland cells. (**A**) Schematic of the Notch signaling pathway and its components. (**B**–**I**) Knockdown of Serrate-Notch signaling components in Intermediate progenitor (green) using *CHIZ-Gal4, UAS-mGFP* by expressing *Notch*^*RNAi*^ (**C**), *Ser*^*RNAi*^ (**D**), and *neur*^*RNAi*^ (**E**) resulted in drastically reduced crystal cells (magenta) in the lymph gland. The increase in the number of crystal cells phenotype in *CSN5*^*RNAi*^ (**F**) is rescued by downregulation of Serrate-Notch signaling components in the *CSN5*^*RNAi*^ background in the intermediate progenitors by expressing *Notch*^*RNAi*^ (**G**), *Ser*^*RNAi*^ (**H**), and *Neur*^*RNAi*^ (**I**) along with *CSN5*^*RNAi*^. (**J**) Crystal cell index in (**B**–**I**). *w*^*1118*^ (*n* = 18), *Notch*^*RNAi*^ (*n* = 21, *P* = 0.0251), *CSN5*^*RNAi*^ (*n* = 29, *P* < 0.0001), *Notch*^*RNAi*^*;CSN5*^*RNAi*^ (*n* = 20, *P* = 0.9487), *w*^*1118*^ (*n* = 13), *Ser*^*RNAi*^ (*n* = 10, *P* = 0.0491), *CSN5*^*RNAi*^ (*n* = 12, *P* < 0.0001), *Ser*^*RNAi*^*;CSN5*^*RNAi*^ (*n* = 10, *P* = 0.8898), *w*^*1118*^ (*n* = 15), *neur*^*RNAi*^ (*n* = 14, *P* = 0.0449), *CSN5*^*RNAi*^ (*n* = 14, *P* < 0.0001), *neur*^*RNAi*^*;CSN5*^*RNAi*^ (*n* = 14, *P* = 0.9866). (**K**) CHIZ index in (**B**–**I**). *w*^*1118*^ (*n* = 18), *Notch*^*RNAi*^ (*n* = 21, *P* = 0.4429), *CSN5*^*RNAi*^ (*n* = 29, *P* < 0.0001), *Notch*^*RNAi*^*;CSN5*^*RNAi*^ (*n* = 20, *P* = 0.8932), *w*^*1118*^ (*n* = 13), *Ser*^*RNAi*^ (*n* = 25, *P* = 0.0627), *CSN5*^*RNAi*^ (*n* = 12, *P* = 0.0003), *Ser*^*RNAi*^*;CSN5*^*RNAi*^ (*n* = 10, *P* > 0.9999), *w*^*1118*^ (*n* = 15), *neur*^*RNAi*^ (*n* = 29, *P* = 0.0708), *CSN5*^*RNAi*^ (*n* = 15, *P* < 0.0001), *neur*^*RNAi*^*;CSN5*^*RNAi*^ (*n* = 14, *P* = 0.4922). (**L**, **M**) Knockdown of CSN5 in lymph gland intermediate progenitors using *CHIZ-Gal4* driver by expressing *CSN5*^*RNAi*^ resulted in increased expression of Notch activity reporters marked by *NRE-GFP* as compared to control (**L**). (**N**) NRE-GFP+ cell index in (**L**, **M**). *CSN5*^*RNAi*^ (*n* = 15, *P *< 0.0001), and *w*^*1118*^ (*n* = 15). (**O**–**T”**) Mock FLP-out Gal4-expressing clones (GFP-positive cells, green) show no reduction in NICD expression (magenta, **O**–**O’′**), whereas similar clones expressing *Notch*^*RNAi*^ and *Ser*^*RNAi*^ show a very strong reduction in NICD protein expression (**Q**–**Q’′**, **S**–**S”**). FLP-out Gal4-expressing clones expressing *CSN5*^*RNAi*^ (**P**–**P’′**) showed increased NICD levels. Coexpression of *CSN5*^*RNAi*^ with either *Notch*^*RNAi*^ (**R**–**R’′**) or *Ser*^*RNAi*^ (**T**–**T’′**) restored NICD expression to levels comparable to the control (**O**–**O′**), showing no reduction. Mock FLP-out Gal4-expressing clones (GFP-positive cells, green) is demarcated with yellow dashed line. FLP-out clones were made using *Hand-Gal4 Hml*^*Δ*^*-Gal4 UAS-FLP Ay-Gal4 UAS-GFP* at 29 °C. (**U**, **V**) Knockdown of CSN5 in lymph gland intermediate progenitors using *CHIZ-Gal4* driver by expressing *CSN5*^*RNAi*^ (**V**–**V’**) resulted in increased expression of *Ser-GFP* as compared to control (**U**–**U’**). (**W**) Ser-GFP index in (**U**,** V**): *w*^*1118*^ (*n* = 19), *CSN5*^*RNAi*^ (*n* = 18, *P* = 0.0009). All confocal images represent maximum intensity projections of the middle third optical sections (except **O**–**V’**, the single optical section) of the wandering third instar lymph gland lobe from at least three independent biological experiments. White dashed lines demarcate one lymph gland lobe. Scale bar, 25 μm. (**U’**, **V’**: 10 μm). Error bars in graphs: mean ± S.D. n.s.: not significant (*P* ≥ 0.05); **P < 0.01; ***P < 0.001; ****P < 0.0001. One-way ANOVA with Tukey’s post-hoc test was used for analysis involving (**J**, **K**). A two-tailed, unpaired Student’s *t* test was employed to compare the mean value of the two groups in (**N**, **W**). Detailed genotypes are in Appendix Table S1. Source data are available online for this figure.

**Figure EV4:**
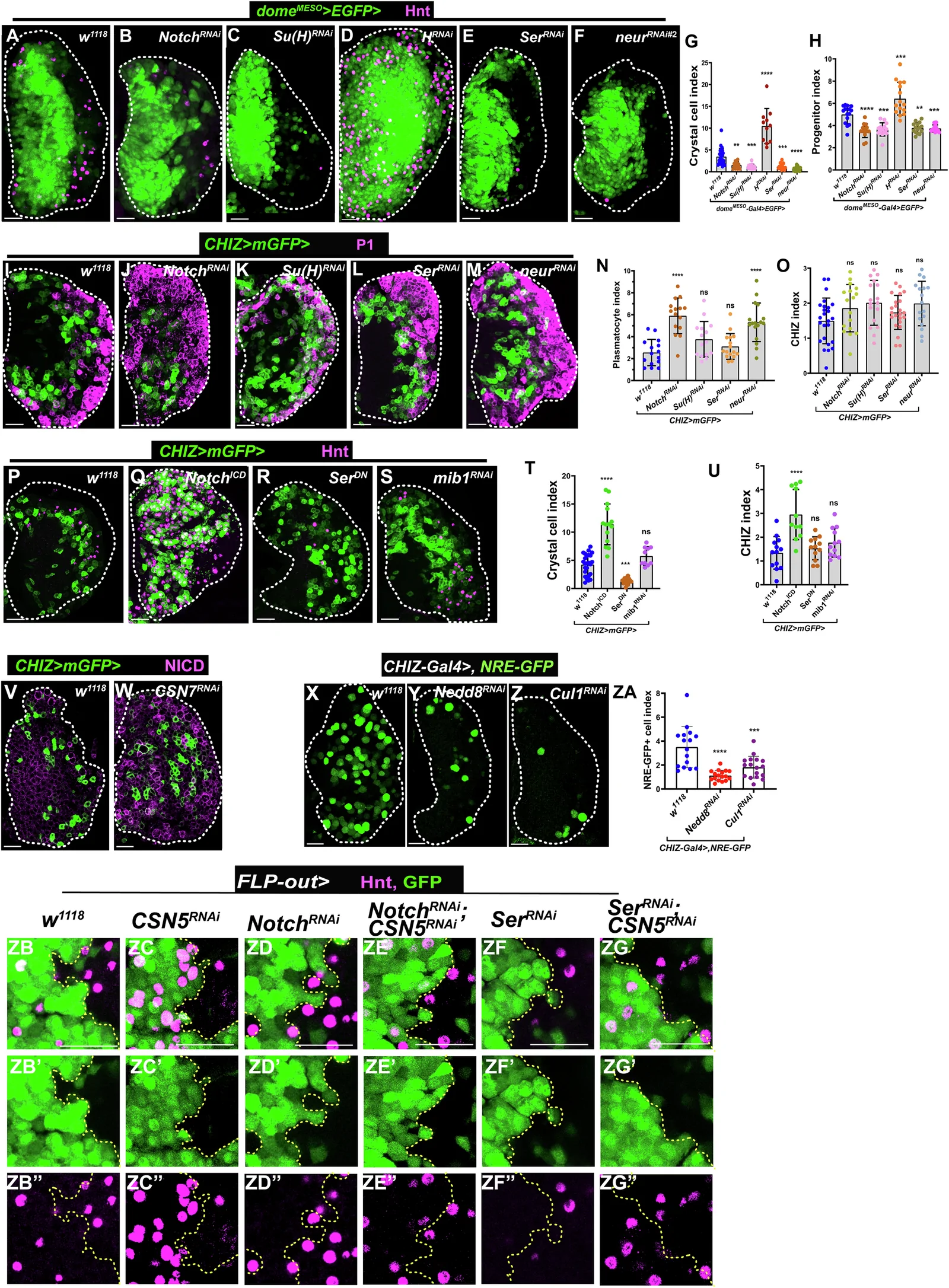
CSN modulates Neuralized/Serrate-mediated Notch activity in developing lymph gland cells, related to Fig. 4. (**A**–**F**) Knockdown of Notch-Serrate signaling pathway components in lymph gland progenitors (green) using *dome*^*MESO*^*-Gal4, UAS-EGFP* driver by expressing *Notch*^*RNAi*^ (**B**), *Su(H)*^*RNAi*^ (**C**), *Ser*^*RNAi*^ (**E**), and *neur*^*RNAi#2*^ (V108239) (**F**) resulted in very few crystal cells, while knockdown of Notch negative regulator Hairless by expressing *H*^*RNAi*^ (**D**) caused a dramatic increase in crystal cells marked by Hnt positive cells (magenta) compared to the control (*dome*^*MESO*^*-Gal4, UAS-EGFP/+*) (**A**). (**G**) Crystal cell index in (**A**–**F**). *Notch*^*RNAi*^(*n* = 15, *P* = 0.0057), *Su(H)*^*RNAi*^ (*n* = 15, *P* = 0.0007), *H*^*RNAi*^ (*n* = 11, *P* < 0.0001), *Ser*^*RNAi*^ (*n* = 16, *P* = 0.0005), *neur*^*RNAi#2*^(*n* = 24, *P* < 0.0001) and *w*^*1118*^ (*n* = 25). (**H**) Progenitor index in (**A**–**F**). *Notch*^*RNAi*^ (*n* = 14, *P* < 0.0001), *Su(H)*^*RNAi*^ (*n* = 14, *P* = 0.0004), *H*^*RNAi*^ (*n* = 15, *P* = 0.0001), *Ser*^*RNAi*^ (*n* = 15, P = 0.0013), *neur*^*RNAi#2*^ (*n* = 15, *P* = 0.0008) and *w*^*1118*^ (*n* = 15). (**I**–**M**) Knockdown of Notch-Serrate signaling pathway components in lymph gland intermediate progenitors (green) using *CHIZ-Gal4, UAS-mGFP* driver by expressing *Notch*^*RNAi*^ (**J**) and *neur*^*RNAi*^ (**M**) resulted in higher plasmatocyte number, while *Su(H)*^*RNAi*^ (**K**), *Ser*^*RNAi*^ (**L**) caused no significant change in plasmatocyte number compared to the control (*CHIZ-Gal4, UAS-mGFP /+*) (**I**). (**N**) Plasmatocyte index in (**I**–**M**). *w*^*1118*^ (*n* = 15), *Notch*^*RNAi*^ (*n* = 15, *P* < 0.0001), *Su(H)*^*RNAi*^ (*n* = 15, *P* = 0.1898), *Ser*^*RNAi*^ (*n* = 16, *P* = 0.8407), and *neur*^*RNAi*^ (*n* = 17, *P* < 0.0001). (**O**) CHIZ index in (**I**–**M**). *w*^*1118*^ (*n* = 25), *Notch*^*RNAi*^ (*n* = 17, *P* = 0.3425), *Su(H)*^*RNAi*^ (*n* = 17, *P* = 0.0689), *Ser*^*RNAi*^ (*n* = 23, *P* = 0.661) and *neur*^*RNAi*^ (*n* = 17, *P* = 0.0869). (**P**–**S**) Alteration of Notch-Serrate signaling pathway components in lymph gland intermediate progenitors (green) using *CHIZ-Gal4, UAS-mGFP* driver by expressing *Notch*^*ICD*^ (**Q**) leads to increased crystal cells marked by Hnt (magenta), reduced by expressing *Ser*^*DN*^ (**R**), and no change by expressing *mib1*^*RNAi*^ (**S**) as compared to wild-type lymph gland (*CHIZ-Gal4, UAS-mGFP*) (**P**). (**T**) Crystal cell index in (**P**–**S**). *w*^*1118*^ (*n* = 25), *Notch*^*ICD*^ (*n* = 14, *P* < 0.0001), *Ser*^*DN*^ (*n* = 17, *P* = 0.0003), and *mib1*^*RNAi*^ (*n* = 10, *P* = 0.2454). (**U**) CHIZ index in (**P**–**S**): *w*^*1118*^ (*n* = 13), *Notch*^*ICD*^ (*n* = 11, *P* < 0.0001), *Ser*^*DN*^ (*n* = 12, *P* = 0.9109), and *mib1*^*RNAi*^ (*n* = 12, *P* = 0.4629). (**V**–**W**) Knockdown of CSN5 in lymph gland intermediate progenitors using the *CHIZGal4* driver by expressing *CSN7*^*RNAi*^ (**W**) resulted in increased NICD expression compared to control (**V**). (**X**–**Z**) Knockdown of *Nedd8* and *Cullin1* by expressing *Nedd8*^*RNAi*^ (**Y**) and *Cul1*^*RNAi*^ (**Z**) using the *CHIZ-Gal4* driver resulted in decreased expression of Notch activity reporters marked by *NRE-GFP* as compared to control (**X**). (**ZA**) NRE-GFP+ cell index in (**X**–**Z**). *w*^*1118*^ (*n* = 16), *Nedd8*^*RNAi*^ (*n *= 17, *P* < 0.0001), and *Cul1*^*RNAi*^ (*n* = 17, *P* = 0.0003). (**ZB**–**ZG”**) Mock FLP-out Gal4-expressing clones (GFP-positive cells, green) show no reduction in the crystal cell number marker by Hnt (magenta, **ZB**–**ZB’′**), whereas similar clones expressing *Notch*^*RNAi*^ and *Ser*^*RNAi*^ show a very strong reduction in crystal cell number (**ZD**–**ZD’′**, **ZF**–**ZF”**). FLP-out Gal4-expressing clones expressing *CSN5*^*RNAi*^ (**ZC**–**ZC’′**) showed increased crystal cell number. Coexpression of *CSN5*^*RNAi*^ with either *Notch*^*RNAi*^ (**ZE**–**ZE’′**) or *Ser*^*RNAi*^ (**ZG**–**ZG’′**) restored crystal cell number to levels comparable to the control (**ZB**–**ZB”**), showing no reduction. Mock FLP-out Gal4-expressing clones (GFP-positive cells, green) are demarcated with a yellow dashed line. FLP-out clones were made using *Hand-Gal4 Hml*^*Δ*^*-Gal4UAS-FLP Ay-gal4 UAS-GFP* at 29 °C. All confocal images represent maximum intensity projections of the middle third optical sections of the primary lobe of the lymph gland, which has been outlined in white dashed lines and represented with maximum intensity projections of the middle third optical sections, except (**ZB**–**ZG”**) images of lymph glands are the single optical section. “*n*” represents the number of lymph gland lobes analyzed. Scale bar, 25 μm. n.s.: not significant (*P* ≥ 0.05); **P < 0.01; ***P < 0.001; ****P < 0.0001. Bars in graphs: mean with S.D. A one-way ANOVA with Tukey’s post-hoc test was used for analysis involving (**G**, **H**, **N**, **O**, **T**, **U**, **ZA**). Source data are available online for this figure.

Notch signaling is crucial for development in all metazoans, influencing cell fate decisions, proliferation, and survival (Siebel and Lendahl, 2017; Zhou et al, 2022). Delta (Dl) or Serrate (Ser)/Jagged ligands trigger the proteolytic cleavage of the Notch receptor in neighboring cells, releasing the Notch Intracellular Domain (NICD) (Fig. 4A) (Schweisguth, 2004). The NICD subsequently enters the nucleus, where it interacts with co-transcription factors and co-activators to induce the expression of target genes. This pathway specifies the crystal cell fate in Hml-positive cells, with Ser expressed in lineage specifying cells in the intermediate progenitor zone (Ferguson and Martinez-Agosto, 2014; Marcetteau et al, 2025). Here, we investigated the Ser/Notch signaling in the context of CSN-mediated lymph gland progenitor cell fate decision. Notch-depleted intermediate progenitors (*CHIZ* > *mGFP>Notch*^*RNAi*^) and progenitors (*dome*^*MESO*^ > *EGFP>Notch*^*RNAi*^) caused a significantly low number of crystal cells (Figs. 4B,C,J and EV4A,B,G) and an increased number of plasmatocytes (Fig. EV4I,J,N), but no change in CHIZ+ cells (Figs. 4K and EV4O), though altering the progenitor population (Fig. EV4H). Also, we did not detect significant change in the index of TUNEL-positive cells and pH3+ cells in the lymph glands of *Notch*^*RNAi*^ background (Appendix Fig. S6F–U), indicating that the loss of Notch activity has no alteration in cell proliferation or cell death in the lymph gland. Expressing an active form of Notch in the intermediate progenitors (*CHIZ* > *mGFP>Notch*^*ICD*^) caused a significantly large number of crystal cells (Fig. EV4P,Q,T) and CHIZ+ cells (Fig. EV4U). The transcription regulators Hairless and Suppressor of Hairless [Su(H)] play a central role in Notch signaling that regulates a broad spectrum of cell-fate determinations (Bray and Furriols, 2001; Fortini and Artavanis-Tsakonas, 1994). Hairless (H) interacts with Su(H) to recruit co-repressors, inhibiting transcriptional activation of Notch-responsive genes without activating the Notch pathway (DeHaro-Arbona et al, 2024; Morel et al, 2001). Su(H)-depletion resulted in significantly fewer crystal cells (Fig. EV4A,C,G) and no change in plasmatocyte numbers (Fig. EV4I,K,N), consistent with published works (Blanco-Obregon et al, 2020; Tokusumi et al, 2010). In contrast, H depletion resulted in a significant increase in the number of crystal cells (Fig. EV4A,D,G) and progenitor populations compared with controls (Fig. EV4H).

Furthermore, altering Serrate activity in the intermediate progenitors (*CHIZ* > *mGFP>Ser*^*RNAi*^*or Ser*^*DN*^) and in progenitors (*dome*^*MESO*^ > *EGFP>Ser*^*RNAi*^) caused a significantly low number of crystal cells (Figs. 4B,D,J and EV4A,E,G,P,R,T); with no significant change in CHIZ+ cells (Figs. 4K and EV4O,U) and plasmatocyte numbers in the lymph gland (Fig. EV4I,L,N), consistent with earlier works (Mukherjee et al, 2011; Spratford et al, 2021). The E3 ubiquitin ligases Neuralized (Neur) and Mindbomb 1 (Mib1) are necessary for endocytosis-dependent activation of Ser and subsequent Notch activation in neighboring cells (Berndt et al, 2017; Daskalaki et al, 2011; Le Borgne et al, 2005). To test which one is involved in this process, we depleted Neur in the lymph gland (*CHIZ* > *mGFP>neur*^*RNAi*^ or *dome*^*MESO*^ > *EGFP>neur*^*RNAi*^) using two different *neur*^*RNAi*^ lines (BL26023 and v108239). In both cases, we observed a significant decrease in crystal cell numbers (Figs. 4B,E,J and EV4A,F,G), no significant change in CHIZ+ cells (Figs. 4K and EV4O), but an increase in the plasmatocytes population (Fig. EV4I,M,N). However, Mib1-depletion in intermediate progenitors caused no significant change in the number of these populations (Fig. EV4P,S,T), though the same *mib1*^*RNAi*^ caused a phenotype similar to *neur*^*RNAi*^ in the wing (Bardin and Schweisguth, 2006; Wang and Struhl, 2005) (Appendix Fig. S5A–C). Thus, our data indicate that Neur is mainly involved in the Ser/Notch signaling-mediated crystal cell differentiation. Next, we rescued the high numbers of crystal cells and CHIZ cells in CSN5-depleted lymph gland by expressing *Notch*^*RNAi*^, *Ser*^*RNAi*^, and *neur*^*RNAi*^ (Fig. 4B–K), indicating that Notch signaling is downstream of CSN. During lymph gland development, an active Notch signaling reporter, NRE-GFP, is expressed from 36 h ALH, and the number of NRE-GFP-positive cells increase through the third larval instar (Appendix Fig. S5D–G), consistent with the requirement of Notch for crystal cell differentiation and its maintenance (Mukherjee et al, 2011). We found that CSN-depleted intermediate progenitors exhibited a significant increase in cells positive for widely used Notch reporters, as indicated by NRE-GFP (Saj et al, 2010) (Fig. 4L–N), Enhancer of Split transcriptional reporter (E(spl)mβHLH-GFP) (Tian et al, 2023), Su(H)-lacZ (Appendix Fig. S5H–M) (Blanco-Obregon et al, 2020), and high-intensity NICD immunostaining (Fig. EV4V–W) in the differentiated zone of the lymph gland. Consistently, depletion of Nedd8 and Cul1 in intermediate progenitors significantly reduced the number of NRE-GFP+ cells in the lymph glands compared to controls (Fig. EV4X–ZA).

Considering that Notch/Serrate signaling functions as a mechanism for transmitting signals between neighboring cells, often within the same tissue, we tested it by generating a somatic clone in the developing lymph gland that spans the progenitor-to-differentiated zone using *HHLT* > *GFP* [*Hand-Gal4, Hml*^*Δ*^*-Gal4, UAS-FLP, UAS-EGFP; Gal4-Act5C(FRT.CD2).P*], a lineage trace driver for the lymph gland. Due to inefficient Flippase activity, lineage patches of Gal4 expression are observed throughout the lymph gland with patches (Mondal et al, 2014). We observed a high number of crystal cells (Hnt + ) and high-intensity NICD immunostaining in *HHLT* > *GFP* > *CSN5*^*RNAi*^ lymph gland cells compared to neighboring cells (Figs. EV4ZB–ZC” and 4O–P”). Notably, both coexpression of *HHLT* > *GFP* > *CSN5*^*RNAi*^*;Ser*^*RNAi*^ and *HHLT* > *GFP* > *CSN5*^*RNAi*^*;Notch*^*RNAi*^ rescued crystal cell numbers and high-intensity NICD immunostaining in the *CSN5*^*RNA*i^-expressing cells compared to neighboring cells in the lymph gland (Figs. EV4ZB–ZG” and 4O–T”).

A protein trap line (*Ser-GFP*; BL59824) showed that Ser-GFP recapitulates Ser immunostaining in the larval wing disc and brain (Sood et al, 2024) (Appendix Fig. S5N–O), and also in early third instar lymph gland (Marcetteau et al, 2025), including posterior signaling center (Appendix Fig. S5P) (Lebestky et al, 2003). We observed that Ser-GFP+ cells overlap with CHIZ>mRFP+ cells during the mid-second instar. However, by the mid-third instar, Ser-GFP+ cells no longer colocalize with CHIZ>mRFP + , and Ser-GFP expression markedly decreased by the wandering third instar, and this pattern aligns with previously reported expression dynamics (Spratford et al, 2021) (Appendix Fig. S5Q–U). We further examined its expression in the lymph gland by expressing *Ser*^*RNAi*^ (*CHIZ>Ser*^*RNAi*^*; Ser-GFP*) backgrounds, which drastically reduced Ser-GFP expression in the lymph glands (Appendix Fig. S5V–X). This dynamic Ser expression pattern reflects a tight temporal regulation of its expression during lymph gland development, as reported earlier (Spratford et al, 2021). This suggests that all the Ser-expressing cells in the lymph gland should undergo a CHIZ+ state at some point during development. Interestingly, CSN5-depletion in the CHIZ+ cells dramatically increased Ser-GFP expression, which persisted until wandering third-instar lymph glands (Fig. 4U–W). Based on earlier studies and our data, we conclude that during lymph gland development, CSN suppresses Ser to prevent activating Notch signaling in adjacent cells, which is essential for crystal cell differentiation.

### CSN is genetically associated with EGFR signaling during lymph gland cell fate decision

**Figure EV5:**
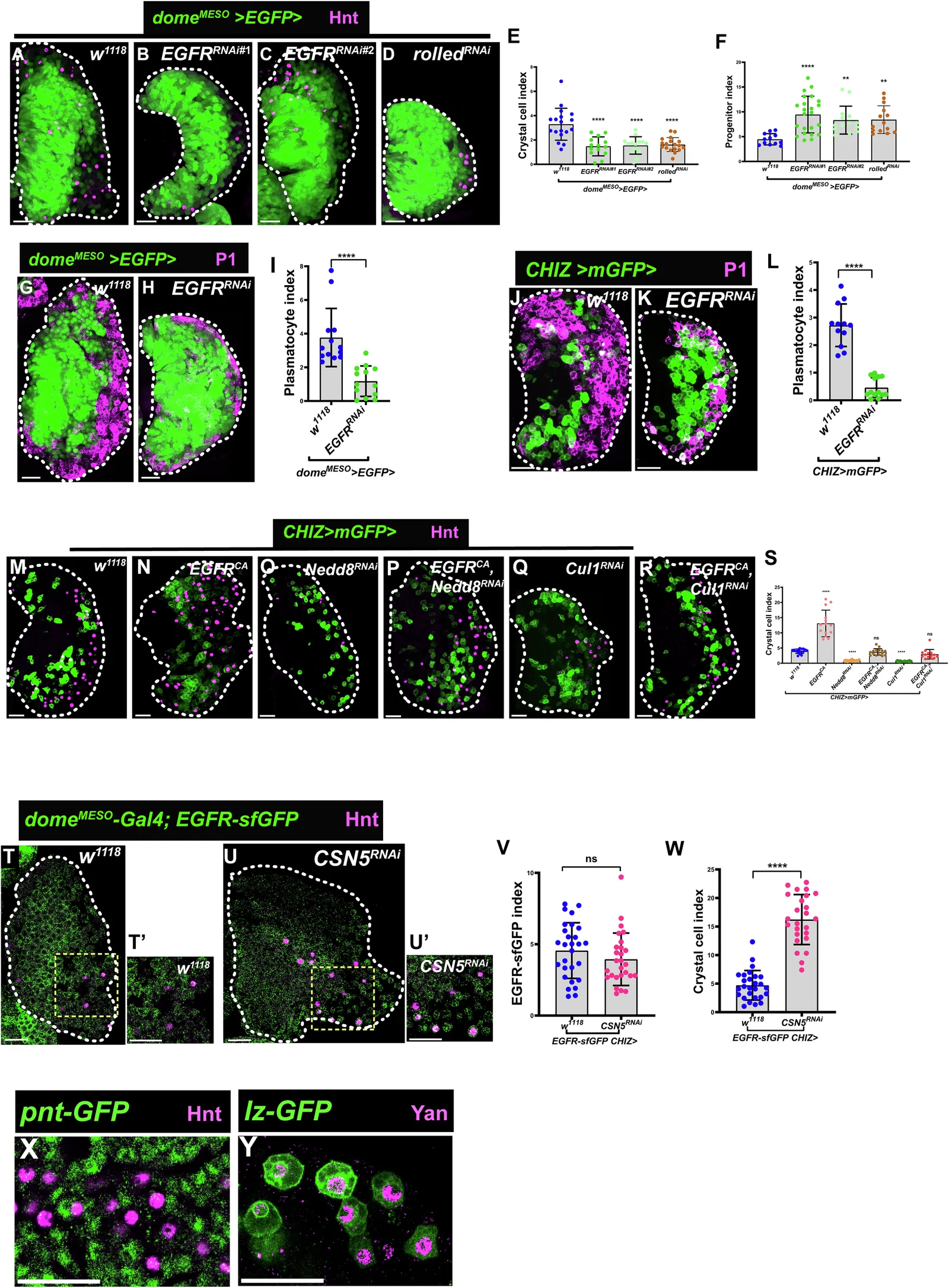
CSN is genetically associated with EGFR signaling during lymph gland cell fate decision, related to Fig. 5. (**A**–**D**) Knockdown of the EGFR signaling pathway in lymph gland progenitors (green) using *dome*^*MESO*^*-Gal4, UAS-2XEGFP* driver by expressing *EGFR*^*RNAi#1*^(BL31525) (**B**), *EGFR*^*RNAi#2*^(BL60012) (**C**), and *rolled*^*RNAi*^ (**D**) leads to decreased crystal cells number marked by Hnt (magenta) compared to wild-type lymph gland (*dome*^*MESO*^*-Gal4, UAS-2XEGFP/+*) (**A**). (**E**) Crystal cell index in (**A**–**D**). *w*^*1118*^ (*n* = 18), *EGFR*^*RNAi#1*^ (*n* = 14, *P* < 0.0001), *EGFR*^*RNAi#2*^ (*n* = 14, *P* < 0.0001), and *rolled*^*RNAi*^ (*n* = 18, *P* < 0.0001). (**F**) Progenitor index in (**A**–**D**). *w*^*1118*^ (*n* = 12), *EGFR*^*RNAi#1*^ (*n* = 23, *P* < 0.0001), *EGFR*^*RNAi#2*^ (*n* = 14, *P* = 0.0082), and *rolled*^*RNAi*^ (*n* = 14, *P* = 0.0065). (**G**, **H**) Knockdown of the EGFR signaling pathway in lymph gland progenitors (green) using *dome*^*MESO*^*-Gal4, UAS-2XEGFP* driver by expressing *EGFR*^*RNAi#*1^ (**H**) leads to decreased plasmatocytes marked by P1 (magenta) as compared to wild-type lymph gland (*dome*^*MESO*^*-Gal4, UAS-2XEGFP/+*) (**G**). (**I**) Plasmatocyte index in (**G**, **H**). *w*^*1118*^ (*n* = 13), and *EGFR*^*RNAi#*1^ (*n* = 13, *P* < 0.0001). (**J**, **K**) Knockdown of the EGFR signaling pathway in lymph gland core progenitors (green) using *Tep4-Gal4, UAS-EGFP* driver by expressing *EGFR*^*RNAi#1*^ (**K**) shows no significant change in crystal cell (yellow) and plasmatocytes (magenta) number as compared to wild-type lymph gland (*Tep4-Gal4, UAS-EGFP /+*) (**J**). (**L**) Plasmatocyte index in (**J**, **K**). *w*^*1118*^ (*n* = 12), and *EGFR*^*RNAi*^ (*n* = 13, *P* < 0.0001). (**M**–**R**) Knockdown of *Nedd8* and *Cullin1* by expressing *Nedd8*^*RNAi*^ (**O**) and *Cul1*^*RNAi*^ (**Q**) using the *CHIZ-Gal4* driver resulted in decreased crystal cells marked by Hnt (magenta) compared to control (**M**). The increase in the number of crystal cells phenotype in *EGFR*^*CA*^ (**N**) is rescued by downregulation of *Nedd8* and *Cullin1* in the *EGFR*^*CA*^ background in the intermediate progenitors by expressing (**P**), and *Cul1*^*RNAi*^ (**R**). (**S**) Crystal cell index in (**M**–**R**). *w*^*1118*^ (*n* = 13), *EGFR*^*CA*^ (*n* = 14, *P* < 0.0001), *Nedd8*^*RNAi*^ (*n* = 15, P < 0.0001), *EGFR*^*CA*^*; Nedd8*^*RNAi*^ (*n* = 15, *P* = 0.9988), *Cul1*^*RNAi*^ (*n* = 14, *P* < 0.0001), and *EGFR*^*CA*^*; Cul1*^*RNAi*^ (*n* = 15, *P* = 0.6675). (**T**–**U’**) Knockdown of CSN5 in lymph gland progenitors using *dome*^*MESO*^*-Gal4* driver by expressing *CSN5*^*RNA*i^ (**U**–**U’**) leads to expression of EGFR protein (green) marked by EGFR activity reporter (*EGFR-sfGFP*) in the periphery crystal cells marked by Hnt (magenta), in comparison to control (*dome*^*MESO*^*-Gal4; EGFR-sfGFP)* (**T**–**T’**). (**V**) EGFR-sfGFP index in (Fig. 6J,K). *CSN5*^*RNAi*^ (*n* = 28, *P* = 0.2401) with control *w*^*1118*^ (*n* = 28). (**W**) Crystal cell index in (Fig. 6J,K). *CSN5*^*RNAi*^ (*n* = 25, *P* < 0.0001) with control *w*^*1118*^ (*n* = 27). (**X**) Pnt expression marked by pnt-GFP is observed throughout the lymph gland except Hnt+ crystal cells. (**Y**) Anti-Yan immunostaining shows Yan expression in the lz-GFP+ crystal cells. All confocal images represent maximum intensity projections of the middle third optical sections, except for image (**T**–**U’**, **X**, **Y**), where the images are single optical sections of the wandering third instar lymph gland lobe from at least three independent biological experiments. White dashed lines demarcate one lymph gland lobe. Scale bar, 25 μm. “*n*” represents the number of lymph gland lobes analyzed. Error bars in graphs: mean ± S.D. n.s.: not significant (*P* ≥ 0.05); **P < 0.01; ***P < 0.001; ****P < 0.0001. A one-way ANOVA with Tukey’s post-hoc test was used for analysis involving (**E**, **F**, **S**). A two-tailed, unpaired Student’s *t* test was employed to compare the mean value of the two groups in (**I**, **L**, **V**, **W**). Source data are available online for this figure.

**Figure 5:**
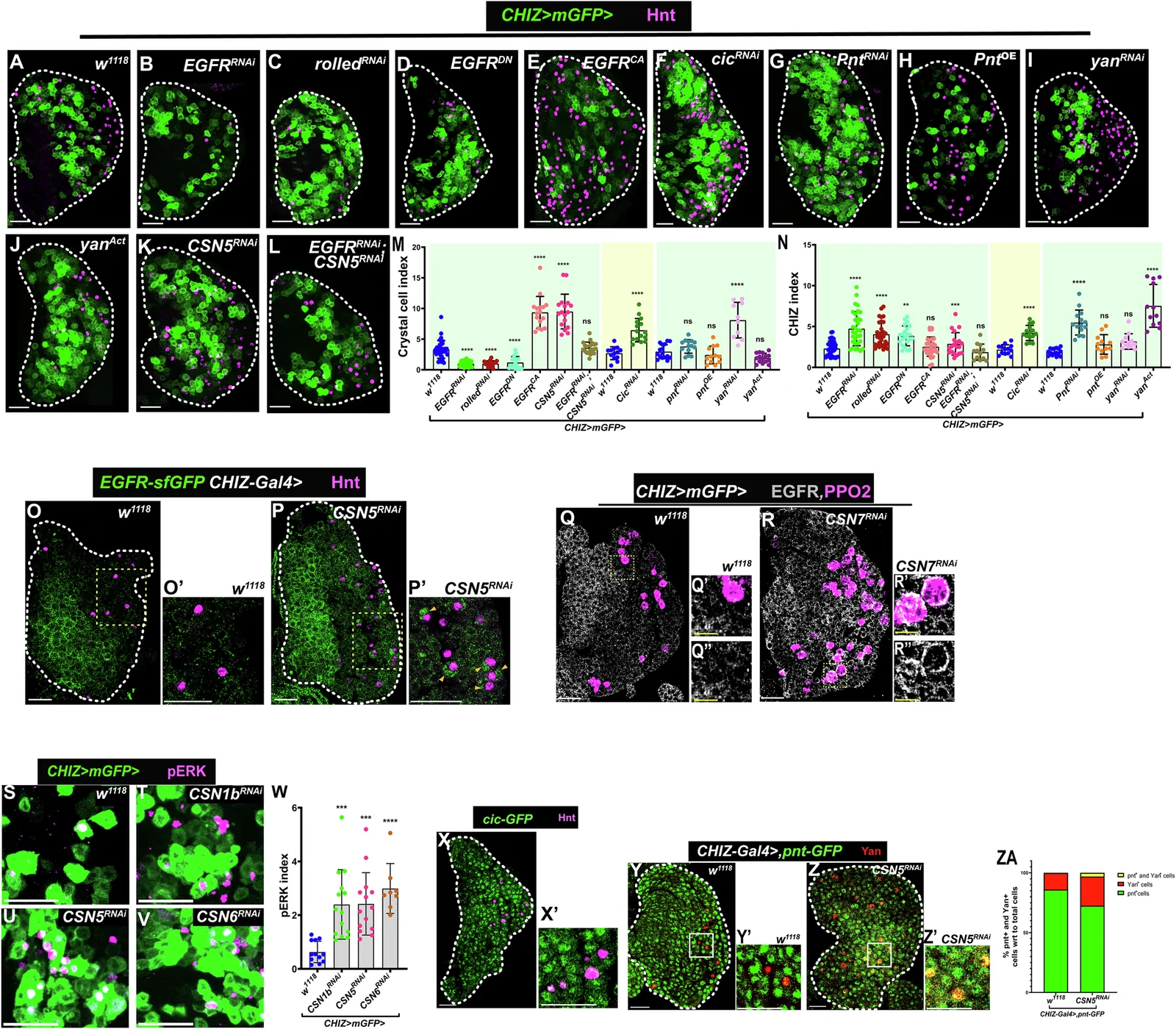
CSN is genetically associated with EGFR signaling during lymph gland cell fate decision. (**A**–**L**) Downregulating EGFR in intermediate progenitors with *EGFR*^*RNA*i^ (**B**), expressing dominant negative *EGFR* (*EGFR*^*DN*^) (*n* = 18) (**D**), and using *rolled*^*RNAi*^ (*n* = 18) (**C**) results in fewer crystal cells. Conversely, expressing the constitutive active *EGFR* (*EGFR*^*CA*^) (*n* = 16) (**E**) causes more crystal cells compared to the control (*n* = 35). Additionally, the increased number of crystal cells with the *CSN5*^*RNAi*^ (*n* = 17) (**K**) phenotype was rescued by expressing *EGFR*^*RNAi*^ in the *CSN5*^*RNAi*^ background (*n* = 20) (**L**) within intermediate progenitors. Knockdown of Capicua in intermediate progenitors by expressing *cic*^*RNA*i^ (*n* = 16) (**F**) leads to an increase in Hnt+ crystal cells compared to the control (**A**) lymph gland. Alterations of pnt and yan in intermediate progenitors by expressing *yan*^*RNA*i^ lead to more crystal cells (**I**), while *pnt*^*OE*^ (**H**), *pnt*^*RNA*i^ (**G**), and *yan*^*Act*^ (**J**), lead to no significant change in crystal cell number as compared to the control (**A**). (**M**) Crystal cell index in (**A**–**L**). *EGFR*^*RNA*i^ (*n* = 25, *P* < 0.0001), *rolled*^*RNAi*^ (*P* < 0.0001), *EGFR*^*DN*^ (*n* = 18, *P* < 0.0001), *EGFR*^*CA*^ (*n* = 16, *P* < 0.0001), *CSN5*^*RNAi*^ (*n* = 17, *P* < 0.0001), *EGFR*^*RNA*i^*; CSN5*^*RNAi*^ (*n* = 20, *P* = 0.9985), and *w*^*1118*^ (*n* = 35)*. cic*^*RNA*i^ (*n* = 16, *P* < 0.0001) and *w*^*1118*^ (*n* = 14)*. pnt*^*RNA*i^ (*n* = 14, *P* = 0.5542); *pnt*^*OE*^ (*n* = 12, *P* = 0. 8822); *yan*^*RNA*i^ (*n* = 10, *P* < 0.0001); *yan*^*Act*^ (*n* = 15, *P* = 0. 3189), and *w*^*1118*^ (*n* = 16). (**N**) CHIZ index in (**A**–**L**). *EGFR*^*RNA*i^ (*n* = 32, *P* < 0.0001), *rolled*^*RNAi*^ (*n* = 30, *P* < 0.0001), *EGFR*^*DN*^ (*n* = 22, *P* = 0.0016), *EGFR*^*CA*^ (*n* = 31, *P* = 0.9953), *CSN5*^*RNAi*^ (*n* = 26, *P* = 0.0006), *EGFR*^*RNA*i^*; CSN5*^*RNAi*^ (*n* = 12, *P* = 0.9673) and *w*^*1118*^ (*n* = 13)*. cic*^*RNA*i^ (*n* = 15, *P* < 0.0001) and *w*^*1118*^ (*n* = 13)*. pnt*^*RNA*i^ (*n* = 16, *P* < 0.0001), *pnt*^*OE*^ (*n* = 12, *P *= 0.4874), *yan*^*RNA*i^ (*n* = 12, *P* = 0.173), *yan*^*Act*^ (*n* = 12, *P* < 0.0001), and *w*^*1118*^ (*n* = 16). (**O**–**P’**) CSN5 depletion in intermediate progenitors (**P**–**P’**) causes accumulation of *EGFR::sfGFP* (green) in Hnt+ cells compared to control (**O**–**O’**) lymph gland (shows a single optical section). Quantification is shown in Fig. EV5V-W. (**Q**–**R”**) CSN7 depletion in intermediate progenitors (**R**–**R”**) leads to accumulation of anti-EGFR immunostaining in PPO2+ cells compared to control (**Q**–**Q”**) lymph gland (shown is a single optical section). (**S**–**V**) Knockdown of CSN in intermediate progenitors using *CHIZ-Gal4, UAS-mGFP* driver by expressing *CSN1b*^*RNA*i^ (**T**), *CSN5*^*RNA*i^ (**U**), and *CSN6*^*RNA*i^ (**V**) leads to increased pERK+ cells in the intermediate progenitor zone compared to the control (**S**). (**W**) pERK index in (**S**–**V**). *CSN1b*^*RNA*i^ (*n* = 14, *P* = 0.0005), *CSN5*^*RNA*i^ (*n* = 14, *P* = 0.0004), *CSN6*^*RNA*i^ (**V**) (*n* = 8, *P* < 0.0001) and *w*^*1118*^ (*n* = 12). (**X**–**X’**) Capicua expression marked by *cic-GFP* is observed throughout the lymph gland except Hnt+ crystal cells. (**Y**–**Z’**) CSN5 depletion in intermediate progenitors, achieved by expressing *CSN5*^*RNAi*^ (*n* = 11) (**Z**–**Z’**), resulted in more Yan+ cells colocalizing with *pnt-GFP* cells compared to the control (*n* = 11) (**Y**–**Y’**). (**ZA**) Percentage of pnt + , yan + , and double positive cells to total cells in (**Y**–**Z’**). *CSN5*^*RNAi*^ (*n* = 11) and *w*^*1118*^ (*n* = 11). All confocal images represent maximum intensity projections of the middle third optical sections, except images (**O**–**R”**, **X**–**X’**, **Y**–**Z’**), which are the single optical sections of the wandering third instar lymph gland lobe from at least three independent biological experiments. White dashed lines demarcate one lymph gland lobe. Scale bar, 25 μm. Each data point represents the number of lymph gland lobes analyzed. Error bars in graphs: mean ± S.D. n.s.: not significant (*P* ≥ 0.05); **P < 0.01; ***P < 0.001; ****P < 0.0001. One-way ANOVA with Tukey’s post-hoc test was used to analyze (**M**, **N**, **W**). Detailed genotypes are in Appendix Table S1. Source data are available online for this figure.

Receptor tyrosine kinase EGFR signaling plays a vital role in regulating and maintaining progenitor cells in the *Drosophila* lymph glands, and it may influence cell fate determination by activating developmental programs (Cho et al, 2024; Goins et al, 2024). We hypothesized that CSN and EGFR might collaborate to influence cell fate determination in the lymph gland. Remarkably, the number of crystal cells decreased significantly when we downregulated EGFR signaling using two separate *EGFR*^*RNAi*^ lines (Fig. EV5A–C,E). The lymph gland with EGFR-depleted progenitors also resulted in significantly fewer plasmatocytes (Fig. EV5G–I) and a higher progenitor index (Fig. EV5F). However, no significant change in the index of TUNEL-positive cells or pH3+ cells was observed in the lymph glands of *EGFR*^*RNAi*^ background (Appendix Fig. S6F–U), indicating no alteration in cell proliferation or cell death in the lymph gland upon EGFR depletion. Using the *Tep4-Gal4* driver to deplete EGFR in core progenitors did not significantly change crystal cell and plasmatocyte populations (Appendix Fig. S6A–D). We next employed a *CHIZ* > *mGFP* driver to perturb EGFR signaling using RNAi or a dominant negative form of EGFR (*EGFR*^*DN*^). This perturbation caused a significant decrease in crystal cells (Fig. 5A,B,D,M) and plasmatocytes (Fig. EV5J–L), while the CHIZ index increased (Fig. 5N). *EGFR*^*CA*^ expression in intermediate progenitors dramatically increased the number of crystal cells (Fig. 5A,E,M), with the CHIZ index remaining unchanged (Fig. 5N). Remarkably, knockdown of CSN5 and EGFR together (*CHIZ* > *mGFP* > *CSN5*^*RNAi*^*; EGFR*^*RNAi*^) restored the number of crystal cells and CHIZ index to control levels (Fig. 5A,B,K–N). Additionally, both *Cul1*^*RNAi*^ and *Nedd8*^*RNAi*^ rescued a large number of crystal cell phenotypes caused by *EGFR*^*CA*^ (Fig. EV5M–S), indicating that EGFR is possibly downstream of CSN, Nedd8, and Cul1.

*EGFR-sfGFP* (BL92329) recapitulates endogenous EGFR protein expression (Revaitis et al, 2020). EGFR expression is reported to be high in the lymph gland posterior signaling center, core progenitors, mid-level in intermediate progenitors, but absent in the differentiated zone (Cho et al, 2024; Goins et al, 2024). Surprisingly, CSN-depletion using *CHIZ-Gal4* and *dome*^*MESO*^*-Gal4* drivers was associated with strong EGFR-sfGFP expression in crystal cells (Figs. 5O–P’ and EV5T–W), and similar results were observed using an anti-EGFR immunostaining (Fig. 5Q–R”).

Activation of EGFR signaling leads to the activation of a kinase cascade to phosphorylate the transcription factor Extracellular Signal-Related Kinase (ERK). To explore downstream of EGFR signaling, we used pERK (active ERK) immunostaining in the lymph gland (anti-pERK CST#4370) (Cho et al, 2024; Ewen-Campen and Perrimon, 2024). We also checked whether the antibody was working in the larval wing disc (Appendix Fig. S6E), as a control, and observed that our immunostaining of pERK appeared similar to that in the published literature (Ewen-Campen and Perrimon, 2024). Remarkably, *CHIZ-Gal4*-driven *CSN*^*RNAi*^ in the lymph gland showed more pERK-positive cells in the intermediate progenitor zone (Fig. 5S–W). A subset of CHIZ cells displayed nuclear pERK, although staining was also sporadically observed throughout the lymph gland, as reported earlier (Spratford et al, 2021). Additionally, when *Drosophila* ERK (also known as *rolled*) was depleted using *rolled*^*RNAi*^ (*dome*^*MESO*^ > *EGFP>rolled*^*RNAi*^ and *CHIZ* > *mGFP>rolled*^*RNAi*^), we observed a significant decrease in crystal cells (Figs. 5A,C,M and EV5A,D,E) along with an increase in CHIZ index (Fig. 5N), and progenitor index (Fig. EV5F).

A study reported that CSN subunits protect Capicua (Cic) from ERK-dependent and -independent degradation mechanisms (Suisse et al, 2017). To explore Cic’s role as a negative regulator of EGFR signaling in lymph gland cell fate, we depleted Cic using a prevalidated RNAi line (BL25995) (Sgammeglia et al, 2023) with *CHIZ-Gal4*, which significantly increased the number of crystal cells (Fig. 5A,F,M) and CHIZ+ cells in the lymph gland (Fig. 5N). Notably, high Cic expression (*Cic-GFP*, BL42267) (Suisse et al, 2017) was observed in lymph gland cells, except in Hnt+ crystal cells (Fig. 5X–X’). These findings may suggest that EGFR signaling is active in the crystal cells. Furthermore, studies reported that the expression of EGFR target genes in *Drosophila* eye development depends on the ratio of transcription activator Pointed (Pnt) and repressor Yan (also known as aop) (Astigarraga et al, 2007; Bernasek et al, 2023; Shwartz et al, 2013). Single-cell transcriptomic data showed variable Pnt expression in most lymph gland cells, with mature crystal cells expressing the least (Girard et al, 2021). We found that Pnt-GFP was expressed in the lymph gland but not in Hnt+ crystal cells (Fig. EV5X). Interestingly, nuclear Yan immunostaining was identified in crystal cells (Spratford et al, 2021), which we also observed (Fig. EV5Y). However, in the CSN5-depleted intermediate progenitor background, some cells showed colocalization of Pnt-GFP and Yan (Fig. 5Y–ZA). We further assessed crystal cell numbers driven by *CHIZ-Gal4* with *pnt*^*RNAi*^, pnt overexpression, *yan*^*RNAi*^, and activated Yan (*yan*^*Act*^). Results showed that crystal cell numbers increased with *yan*^*RNAi*^ and decreased with *yan*^*Act*^, while Pnt manipulation had no significant effect (Fig. 5A,G–J,M), indicating their dynamic participation in cell fate decisions as shown during *Drosophila* eye development (Girard et al, 2021; Spratford et al, 2021). These data suggest that genetic interaction between CSN and EGFR signaling occurs during the lymph gland’s cell fate determination. However, we cannot exclude the possibility that any CSN subunit may have redundant functions, as previously reported (Pan et al, 2014; Suisse et al, 2017).

### The combination of CSN, EGFR, and Notch signalings regulate crystal cell specification in the lymph gland

**Figure 6:**
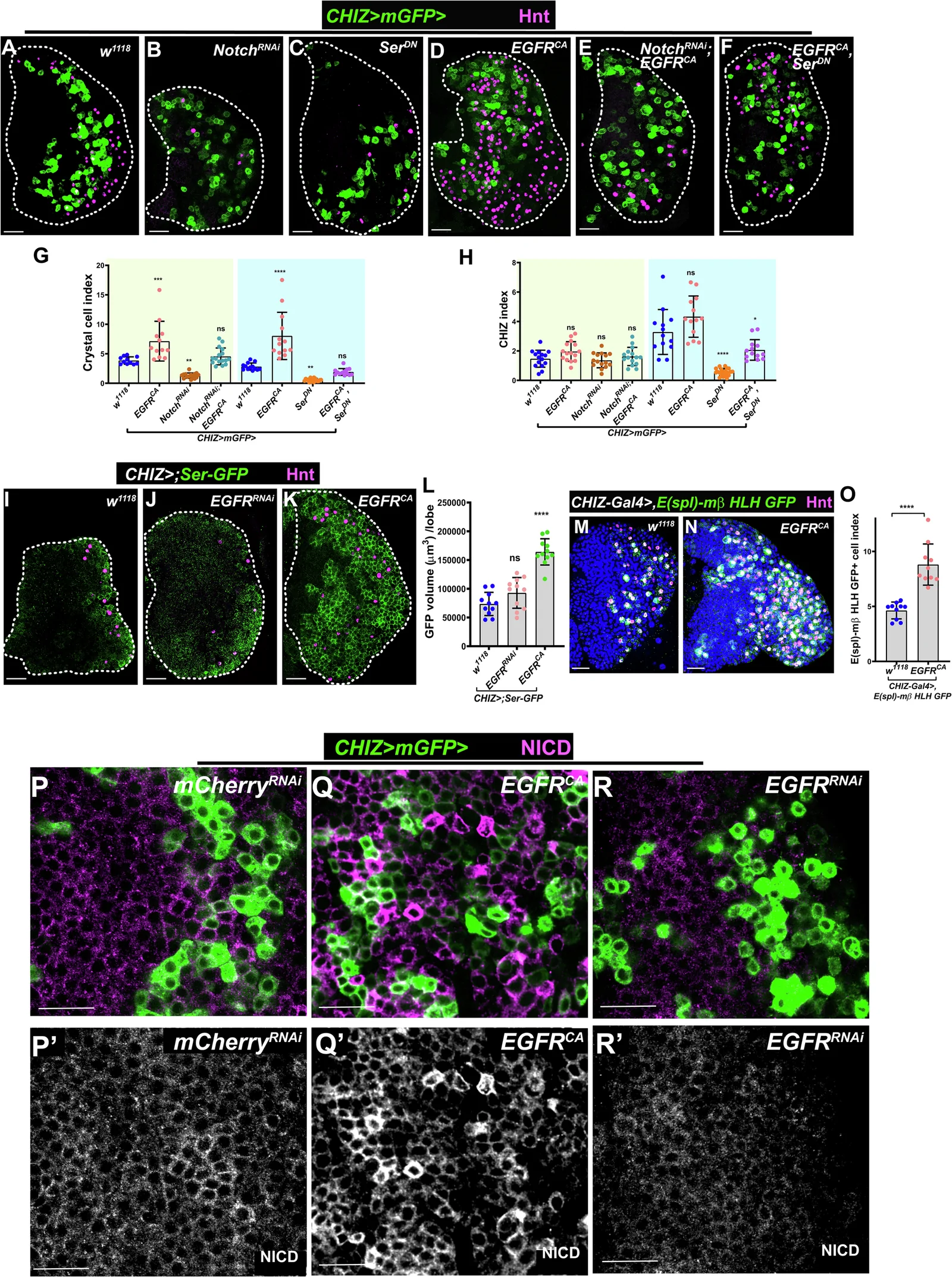
The combination of CSN, EGFR, and Notch signaling regulates crystal cell specification in the lymph gland. (**A**–**F**) A high number of crystal cells in constitutive active EGFR overexpression *EGFR*^*CA*^ (**D**), when compared to control (**A**) in intermediate progenitors are rescued (**E**) by depleting Notch using *Notch*^*RNAi*^ (**E**) and Serrate using *Ser*^*DN*^ (**F**). (**G**) Crystal cell index in (**A**–**F**). *EGFR*^*CA*^ (*n* = 12, *P* = 0.0004), *Notch*^*RNAi*^ (*n* = 15, *P* = 0.004), *Notch*^*RNAi*^*; EGFR*^*CA*^ (*n* = 16, *P* = 0.7604), *w*^*1118*^ (*n* = 11) and *EGFR*^*CA*^ (*n* = 13, *P* < 0.0001), *Ser*^*DN*^ (*n* = 20, *P* = 0.0089), *Ser*^*DN*^*; EGFR*^*CA*^ (*n* = 13, *P* = 0.6232), *w*^*1118*^ (*n* = 11). (**H**) CHIZ index in (**A**–**F**). *EGFR*^*CA*^ (*n* = 15, *P* = 0.1182), *Notch*^*RNAi*^ (*n* = 15, *P *= 0.9571), *Notch*^*RNAi*^*; EGFR*^*CA*^ (*n* = 15, *P* = 0.8978), *w*^*1118*^ (*n* = *15*) and *EGFR*^*CA*^ (*n* = 11, *P* = 0.1027), *Ser*^*DN*^ (*n* = 17, *P* = 0.0008), *Ser*^*DN*^*; EGFR*^*CA*^ (*n* = 17, *P* = 0.0107), *w*^*1118*^ (*n* = 13). (**I**–**K**) Downregulation of EGFR in intermediate progenitors using *CHIZ-Gal4, UAS-mCD8GFP* driver by expressing *EGFR*^*RNA*i^ (**J**), unaffected Ser-GFP staining pattern, but *EGFR*^*CA*^ overexpression in intermediate progenitors causes high Ser-GFP staining in the lymph gland (**K**) compared to control (**I**). (**L**) Quantification of the Ser-GFP volume in (**I**–**K**). *EGFR*^*RNA*i^ (*n* = 14, *P* = 0.9034), *EGFR*^*CA*^ (*n* = 13, *P* < 0.0001), and *w*^*1118*^ (*n* = 17). (**M**, **N**) Activation of EGFR in intermediate progenitors by expressing *EGFR*^*CA*^ (**N**) led to increased Hnt+ cells (magenta) and Notch reporter activity, marked by E(spl)-mβ HLH GFP (green), compared to the control. (**O**) E(spl)-mβ HLH GFP+ cell index in (**M**, **N**). *EGFR*^*CA*^ (*n* = 10, *P* < 0.0001) and *w*^*1118*^ (*n* = 10). (**P**–**R’**) Constitutive active EGFR overexpression in intermediate progenitors showed a significant increase in NICD staining (**Q**–**Q’**) in the intermediate progenitor zone compared to control (*CHIZ* > *mGFP>mCherry*^*RNAi*^) (**P**–**P’**), whereas EGFR-depleted cells show lower NICD staining (**R**–**R’**). See Appendix Fig. S6K–M” for the whole lymph gland, GFP only channel of Fig. 1P–R’, and their consecutive relative fluorescent intensity of NICD and CHIZ+ cells. All confocal images represent maximum intensity projections of the middle third optical sections, except images (**I**–**K**, **P**–**R**’), the single optical section of the wandering third instar lymph gland lobe from at least three independent biological experiments. White dashed lines demarcate one lymph gland lobe. Scale bar, 25 μm. Each data point represents the number of lymph gland lobes analyzed (*n*). Error bars in graphs: mean ± S.D n.s.: not significant (*P* ≥ 0.05); **P < 0.01; ***P < 0.001; ****P < 0.0001, One-way ANOVA with Tukey’s post-hoc test was used for analysis involving (**G**, **H**,**L**). A two-tailed, unpaired Student’s *t* test was employed to compare the mean value of the two groups in (**O**). Detailed genotypes are in Appendix Table S1. Source data are available online for this figure.

The relationship between EGFR and Notch signaling has been reported as context-specific (Doroquez and Rebay, 2006; Sundaram, 2005); for example, they work combinatorially during *Drosophila* eye development (Doroquez and Rebay, 2006; Flores et al, 2000; Nagaraj and Banerjee, 2007; Yasugi et al, 2010). In our observations, CSN genetically interacts with both the EGFR and Notch signaling pathways in lymph gland cell fate determination. We aimed to investigate whether CSN, EGFR, and Notch act in a combinatorial manner in the lymph gland to maintain progenitors and promote proper differentiation. We performed rescue experiments by overexpressing *EGFR*^*CA*^ in the background of Ser-perturbed intermediate progenitors to check if EGFR and Ser genetically interact during crystal cell differentiation. We observed that the dominant-negative form of Ser (*Ser*^*DN*^) rescues the *EGFR*^*CA*^-mediated high number of crystal cell and CHIZ+ cell numbers (Fig. 6A,C,F,G,H), indicating that EGFR and Ser are genetically associated during the crystal cell differentiation. The Ser-GFP expression pattern was unchanged in the wandering third instar lymph gland with EGFR-depleted intermediate progenitors compared to control, but remained higher in the EGFR^CA^ background (Fig. 6I–L), as seen in the CSN5-depleted lymph gland (Fig. 4U–W). These results indicate that EGFR influences Ser activity in the lymph gland.

We next performed a rescue experiment to further validate EGFR-mediated regulation of Notch signaling during crystal cell differentiation by overexpressing EGFR^CA^ in the Notch-depleted background in the intermediate progenitor cells (*CHIZ* > *mGFP>Notch*^*RNAi*^*; EGFR*^*CA*^). We found a significant rescue of a high number of crystal cells in the EGFR^CA^-background (Fig. 6A,B,D,E,G). The same genetic combination also restored plasmatocyte numbers (Appendix Fig. S7A–E). However, we found that EGFR^RNAi^ could not rescue the high number of crystal cells observed upon overexpression of NICD (Appendix Fig. S7F–J), possibly because Notch signaling is downstream of EGFR signaling during crystal cell differentiation in the lymph gland. Notably, EGFR^CA^ overexpression in intermediate progenitors showed a significant increase in Notch reporter activity (Fig. 6M–O). NICD puncta as readout of active Notch stabilization also observed in EGFR^CA^ overexpressed intermediate progenitors (Fig. 6P–Q’; Appendix Fig. S7K–L”); however, EGFR-depleted intermediate progenitors showed lower NICD staining (Fig. 6P–P’,R–R’; Appendix Fig. S7K–K”,M–M”). This suggests that EGFR is genetically associated with Notch activation for lymph gland crystal cell differentiation.

**Figure 7:**
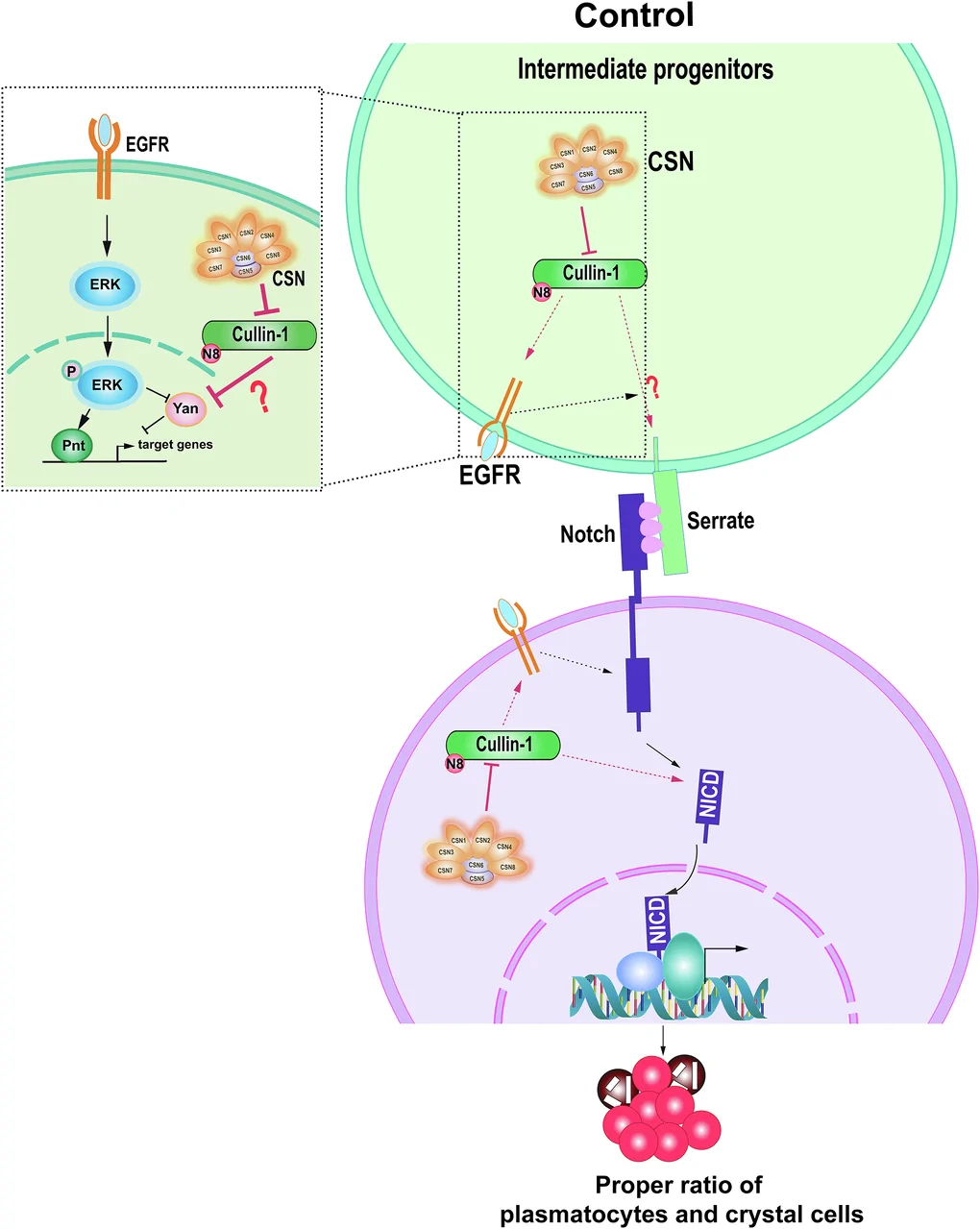
Genetic model of CSN’s regulation of EGFR and Notch pathways. A schematic shows a mechanism of *Drosophila* myeloid-type progenitor-to-crystal cell differentiation through the intermediate progenitor cell, which requires CSN-mediated modulation of the EGFR and Notch signaling pathway.

Therefore, EGFR regulates Notch signaling to maintain the proper ratio of several types of myeloid-type blood cell differentiation, and CSN modulates both EGFR and Notch signaling in lymph gland intermediate progenitors. Taken together, our study revealed that the combinatorial action of CSN-mediated Cul1, EGFR, and Notch signaling is necessary for the proper determination of cell fate in the lymph glands (Fig. 7).

## Discussion

Here, we describe how CSN controls cell fate decisions of myeloid-type progenitors during *Drosophila* lymph gland development by regulating the EGFR and Notch signaling pathways. Knocking down any of the eight CSN components in intermediate progenitor cells of the lymph gland significantly increases the number of crystal cells and decreases plasmatocyte numbers, indicating CSN inhibits crystal cell differentiation and supports plasmatocyte formation during normal lymph gland development. Our results suggest that the CSN holoenzyme functions in conjunction with the Cul1-mediated CRL complex to promote a proper ratio of mature, differentiated cells from intermediate progenitors. We show genetically that CSN regulates Serrate-dependent Notch activation during crystal cell differentiation. We also find that EGFR signaling is genetically associated with the modulation of Notch activity during lymph gland development. Overall, this study reveals that during the normal development of myeloid-type progenitors, a combination of EGFR and Notch signaling is modulated by CSN to make cell fate decisions for a proper number of plasmatocytes and crystal cells in the lymph gland.

Various signaling pathways, including cell-autonomous (e.g., JAK/STAT), microenvironmental (e.g., Hh), and systemic signaling (e.g., insulin, GABA, dopamine, serotonin), regulate the maintenance of lymph gland progenitors (Banerjee et al, 2019; Goins et al, 2024; Kapoor et al, 2022; Krzemien et al, 2007; Liu et al, 2025; Mondal et al, 2014; Shim et al, 2013). However, CSN is dispensable for maintaining lymph gland progenitors, as their markers, signaling activity, cell cycle status, and size are similar to those of the controls.

Our genetic analysis reveals that CSN influences cell fate by regulating the Ser/Notch signaling pathway. Depleting the Notch-negative regulator Hairless (H) in progenitors raises crystal cell numbers, akin to CSN knockdown. This supports a model in which CSN loss activates cullins, degrades H, increases Notch activity (Bang et al, 1995) and causes excessive differentiation of crystal cells. H appears crucial for crystal cells, though more research is needed to understand H’s mechanistic role. Overall, our data extend previous studies on CSN regulating cullins (Wu et al, 2005), which may later control the ubiquitylation of Notch or essential Notch pathway elements (Mummery-Widmer et al, 2009). This comprehensive genetic study extended the earlier reports that the Ser/Notch signaling pathway is crucial for crystal cell specification (Blanco-Obregon et al, 2020; Duvic et al, 2002; Mukherjee et al, 2011; Spratford et al, 2021).

EGFR signaling in *Drosophila* eye development depends on the ratio of transcription activator Pnt and repressor Yan, with Cic also negatively regulating EGFR transcriptional target (Ajuria et al, 2011; Bernasek et al, 2023; Rebay and Rubin, 1995; Shwartz et al, 2013). Single-cell transcriptomic data indicate that Pnt expression levels vary among lymph gland cells, with the lowest levels in mature crystal cells, which, surprisingly, show nuclear Yan (Girard et al, 2021; Spratford et al, 2021). We observed Pnt-GFP in the lymph gland but not in crystal cells. Normally, nuclear Yan did not colocalize with Pnt-GFP, except in CSN5-depleted intermediate progenitors, where there is some colocalization. High Cic-GFP expression is observed in lymph gland cells, except in crystal cells. Interestingly, depletion of Cic or Yan in intermediate progenitors increases the number of crystal cells, a phenomenon similar to that observed with CSN depletion. This supports a model in which the loss of CSN activates Cul1, promoting the degradation of negative regulators, such as Cic, which results in enhanced EGFR signaling and drives excessive differentiation of crystal cells. A similar mechanism was reported, where CSN subunits protect Cic from ERK-dependent and -independent mechanisms of degradation in the *Drosophila* wing disc and in embryonic D2F blood cell line (Suisse et al, 2017), suggesting conserved regulation across developmental contexts. Moreover, recent reports have shown that EGFR signaling is critical for progenitor maintenance and is responsible for the G2-arrested progenitor cells in the third-instar larval lymph gland (Cho et al, 2024; Goins et al, 2024). Overall, EGFR signaling is also crucial for the differentiation of crystal cells.

The EGFR and Notch pathways are sequentially integrated at multiple stages throughout *Drosophila* development, a process similar to that described here. During the development of the *Drosophila* eye, EGFR signaling induces expression of the Notch ligand Delta in photoreceptor cells, which then activates Notch signaling in adjacent cells, leading to their differentiation into cone cells and pigment cells (Flores et al, 2000; Nagaraj and Banerjee, 2007; Tsuda et al, 2002). This sequential integration of the EGFR and Notch pathways ensures proper gene regulation, facilitating the development of the R1, R6, and R7 photoreceptors (Flores et al, 2000; Tomlinson and Struhl, 2001). Similarly, these pathways are sequentially integrated during neuroblast, muscle, and wing vein development (Artero et al, 2003; Shilo, 2005; Yasugi et al, 2010). Likewise, in *C. elegans*, during vulval fate specification, anchor cells release EGF signals that specify the nearest vulval precursor cell (VPC) P6.p to a primary cell fate. This high EGFR activation triggers Notch ligand expression in primary cells, where Notch signaling inhibits EGFR activity in neighboring VPCs and promotes secondary cell fate (Schmid and Hajnal, 2015). Since both the EGFR and Notch pathways are crucial for maintaining blood cell homeostasis, integrating them through CSN-mediated modulation of multiple cross-interactions at different levels is essential for determining blood cell fate and ensuring appropriate numbers of plasmatocytes and crystal cells.

CSN regulates multiple signaling pathways in various cellular functions, including *Drosophila* and mouse immunity (Deng et al, 2011; Fang et al, 2015; Harari-Steinberg et al, 2007; Huang et al, 2014; Lu et al, 2015; Mummery-Widmer et al, 2009; Najor et al, 2017; Su et al, 2020; Suh et al, 2002; Wu et al, 2011). Crystal cells support innate immunity and respiratory function(Shin et al, 2024), highlighting their evolutionary importance. Hematopoietically, they share traits with platelets and erythrocytes from the myeloid lineage (Akashi et al, 2000). Since depleting CSN promotes crystal cell differentiation, CSN could be targeted to enhance immune responses and respiratory health. However, given that CSN regulates multiple signaling pathways, further studies are required to determine whether specific downstream components can be targeted without broader cellular effects. Furthermore, interactions between CSN and EGFR signaling occur in various cancers (Fang et al, 2015; Najor et al, 2017; Su et al, 2020) and many studies have reported that the dysregulation of EGFR and Notch signaling is a significant factor in the development of numerous cancers and diseases (Levantini et al, 2022; Ni et al, 2021; Pancewicz-Wojtkiewicz, 2016). Our work identifies a biological setting in which these interactions might be further analyzed, with a potential to explore potential therapeutic interventions targeting CSN (Schlierf et al, 2016).

## Methods

**Table:** Reagents and tools table

Reagent/resource	Reference or source	Identifier or catalog number
**Experimental models**		
***Drosophila melanogaster***	**Bloomington Drosophila Stock Center (BDSC), Vienna Drosophila Resource Center (VDRC)**	N/A
*dome* ^*MESO*^ *-Gal4, UAS-EGFP*	Utpal Banerjee’s lab	N/A
*Hml* ^*∆*^ *-Gal4, UAS-EGFP*	Utpal Banerjee’s lab	N/A
*CHIZ-Gal4, UAS-mGFP*	Utpal Banerjee’s lab	N/A
*e33c-Gal4*	Maneesha Inamdar’s lab	N/A
*Tep4-Gal4, UAS-EGFP*	Lolitika Mandal’s lab	N/A
*Nplp2-Gal4*	Jiwon Shim’s lab	NA
*Hand-Gal4, Hml* ^*Δ*^ *-Gal4, UAS-FLP.JD1, UAS-2EGFP; P[Gal4-Act5C(FRT.CD2).P] a.k.a. HHLT*	Utpal Banerjee’s lab	NA
*w* ^*1118*^	Utpal Banerjee’s lab	N/A
*UAS-CSN5*	Cheng-Ting Chien’s lab	PMID: 21304511
*UAS-CSN5* ^*D148N*^	Cheng-Ting Chien’s lab	PMID: 21304511
*BcF6-mCherry*	Robert Schulz lab	PMID:27913635
*UAS-mCherry* ^*RNAi*^	BDSC	RRID:BDSC_35785
*UAS-CSN1b* ^*RNAi*^	BDSC	RRID:BDSC_27303
*UAS- CSN2/Alien* ^*RNAi*^	BDSC	RRID:BDSC_28908
*UAS-CSN3* ^*RNAi*^	BDSC	RRID:BDSC_33369
*UAS-CSN4* ^*RNAi*^	BDSC	RRID:BDSC_42798
*UAS-CSN5* ^*RNAi*^	BDSC	RRID:BDSC_28732
*UAS-CSN5* ^*RNAi*^	BDSC	RRID:BDSC_42781
*UAS-CSN5* ^*RNAi*^	BDSC	RRID:BDSC_62970
*UAS-CSN6* ^*RNAi*^	BDSC	RRID:BDSC_36073
*UAS-CSN6* ^*RNAi*^	BDSC	RRID:BDSC_41991
*UAS-CSN7* ^*RNAi*^	BDSC	RRID:BDSC_33663
*UAS-CSN8* ^*RNAi*^	VDRC	v50565/GD
*UAS-CSN6* ^*RNAi*^	VDRC	v100377/KK
*UAS-Ser*	BDSC	RRID:BDSC_5815
*UAS-Ser* ^*DN*^	BDSC	RRID:BDSC_52009
*UAS-neur* ^*RNAi#1*^	BDSC	RRID:BDSC_26023
*UAS-mib1* ^*RNAi*^	BDSC	RRID:BDSC_27320
*UAS-Ser* ^*RNAi*^	VDRC	v27172
*UAS-neur* ^*RNAi#2*^	VDRC	v108239
*UAS-Notch* ^*RNAi*^	Spyros Artavanis-Tsakonas lab	PMID: 26222204
*UAS-Notch* ^*ICD*^	Spyros Artavanis-Tsakonas lab	PMID: 9570768
*UAS-Su(H)* ^*RNAi*^	BDSC	RRID:BDSC_28900
*UAS-H* ^*RNAi*^	BDSC	RRID:BDSC_27315
*Su(H)-lacZ*	BDSC	RRID:BDSC_10689
*NRE-GFP*	BDSC	RRID:BDSC_30727
*E(spl)mß-HLH-GFP*	BDSC	RRID:BDSC_65294
*UAS-Nedd8* ^*RNAi*^	BDSC	RRID:BDSC_33881
*UAS-Nedd8* ^*RNAi*^	VDRC	v28445
*UAS-Cul1* ^*RNAi*^	BDSC	RRID:BDSC_36601
*UAS-Cul1* ^*RNAi*^	BDSC	RRID:BDSC_29520
*Ser-EGFP*	BDSC	RRID:BDSC_59824
*UAS-EGFR* ^*RNAi#1*^	BDSC	RRID:BDSC_31525
*UAS-EGFR* ^*RNAi#2*^	BDSC	RRID:BDSC_60012
*UAS-EGFR* ^*DN*^	BDSC	RRID:BDSC_5364
*UAS-EGFR* ^*CA*^	BDSC	RRID:BDSC_9533
*EGFR-sfGFP*	BDSC	RRID:BDSC_92329
*UAS-rolled* ^*RNAi*^	BDSC	RRID:BDSC_34855
*cic-GFP*	BDSC	RRID:BDSC_42267
*pnt-GFP*	BDSC	RRID:BDSC_42680
*UAS-cic* ^*RNAi*^	BDSC	RRID:BDSC_25995
*UAS-pnt* ^*RNAi*^	BDSC	RRID:BDSC_31936
*UAS-pnt* ^*OE*^	BDSC	RRID:BDSC_869
*UAS-yan* ^*RNAi*^	BDSC	RRID:BDSC_35404
*UAS-yan* ^*Act*^	BDSC	RRID:BDSC_5789
**Antibodies**		
Mouse anti-hindsight	Developmental Studies Hybridoma Bank(DSHB)	Cat# 1G9-s; RRID: AB_528278
Mouse anti-P1 (NimC1)	Istvan Ando’s lab	N/A
Mouse anti-Lz	DSHB	Cat# anti-Lozenge, RRID:AB_528346
Rabbit anti-PPO2	Asano T, 2009	Cat# PPO2, RRID:AB_2566912
Mouse anti-Mys	DSHB	Cat# cf.6g11, RRID:AB_528310
Chicken anti-GFP	Abcam	Cat# ab13970, RRID:AB_300798
Rabbit anti-GFP	Invitrogen	Cat# A11122, RRID: AB_221569
Rabbit anti-Phospho-Histone H3	Cell Signaling Technology (CST)	Cat# 9701, RRID:AB_331535
Mouse anti-β-Galactosidase	Promega	Cat# Z3781, RRID:AB_430877
Chicken anti-β-Galactosidase	Abcam	Cat# ab134435, RRID:AB_2737437
Rat anti-DE-cadherin extracellular domain	DSHB	Cat# DCAD2, RRID:AB_528120
Mouse anti-NICD	DSHB	Cat# c17.9c6, RRID:AB_528410
Rat anti-Cubitus interruptus (Ci) full length	DSHB	Cat# 2A1, RRID:AB_2109711
Mouse anti-Yan	DSHB	Cat# anti-Yan 8B12H9 ; RRID: AB_531807
Mouse anti-EGFR	Sigma	Cat# E2906; RRID:AB_609900
Rabbit anti-Cul1	Thermo Fisher Scientific	Cat# 71-8700, RRID: AB_2534002
Rabbit anti-pERK	CST	Cat#4370; RRID:AB_2315112
Goat anti-mouse Cy3	Jackson Immuno Research	Cat# 115-165-003 RRID: AB_2338680
Donkey anti-mouse AlexaFluor555	Invitrogen	Cat# A31570 RRID: AB_2536180
Goat anti-mouse Alexa-Fluor 647	Invitrogen	Cat# A21050 RRID: AB_2535718
Donkey anti-rabbit AlexaFluor555	Invitrogen	Cat# A31572 RRID: AB_162543
Goat anti-rabbit AlexaFluor647	Invitrogen	Cat# A32733 RRID: AB_2866492
Donkey anti-rabbit AlexaFluor488	Invitrogen	Cat# A21206, RRID:AB_2535792
Goat anti-chicken AlexaFluor488	Invitrogen	Cat# A11039, RRID:AB_2534096
Goat anti-chicken AlexaFluor546	Invitrogen	Cat# A-11040, RRID:AB_2534097
Goat anti-rat Alexa-Fluor 546	Invitrogen	Cat# A-11081, RRID:AB_2534125
**Oligonucleotides and other sequence-based reagents**		
RT-PCR primers for CSN1b	F: 5’-CCAATTCGATCAAGGAGAGC-3’ *R:* 5’-AATAGCACTTCAGGGCATTG-3’	This study
RT-PCR primers for CSN2	*F:* 5’-GAGCATCAATTCGATTCTGGACT-3’ *R:* 5’-GGTGTTGGTCTTAAACCACAGC-3’	This study
RT-PCR primers for CSN3	*F:* 5’-TCTGTTTACCGAGTTTGTGG-3’ *R:* 5’-GGGTTTCCAACTGTCGAATC-3’	This study
RT-PCR primers for CSN4	*F:* 5’-ATCTGGAGGACAACGATTCG-3’ *R:* 5’-CTGCAACTCCTCGGAATTAG-3’	This study
RT-PCR primers for CSN5	*F:* 5’-GGTGATGGGTCTAATGCTTGG-3’ *R:* 5’-CCATGTAGGCGGTCATGTACT-3’	This study
RT-PCR primers for CSN6	*F:* 5’-TGAAACAGTGATCAACAAGGACTAC-3’ *R:* 5’-ACCAGCCTATGAAGTCGAGATC-3’	This study
RT-PCR primers for CSN7	*F:* 5’-TCCAAGCACTTCGAGACC-3’ *R:* 5’-CAGCTCGATGAACTTTTCCG-3’	This study
RT-PCR primers for CSN8	*F:* 5’-CCGGAATTCATGCATTTAAATAAATATAGTG-3’ *R:* 5’-CCCGCTCGAGTTAATTCTCTAGGAAGGTTAC-3’	This study
RT-PCR primers for RP49	*F:* 5´-TTGAGAACGCAGGCGACCGT-3´ *R:* 5´-CGTCTCCTCCAAGAAGCGCAAG-3´	This study
**Chemicals, enzymes and other reagents**		
4% PFA (paraformaldehyde)	Thermo Fisher Scientific	Cat# 28908
DABCO (1,4-diazabicyclo [2.2. 2]octane)	Sigma	Cat# D27802
Triton X-100	Sigma	Cat# T8787
Bovine Serum Albumin	SRL	Cat# 83803
Thiomersal	SRL	Cat# 85090
TRI Reagent	Sigma	Cat#T9424
DNase I Solution	Thermo Fisher Scientific	Cat# 89836
Reaction Buffer with MgCl2 for DNase I (10X)	Thermo Fisher Scientific	Cat# B43
Random Hexamer Primer	Thermo Fisher Scientific	Cat# SO142
Ethylenediaminetetraacetic acid (EDTA) (0.5 M), pH 8.0	Thermo Fisher Scientific	Cat# R1021
SYBR Green qPCR Master Mix	Genetix	Cat# PKG025-A
RevertAid Reverse transcriptase	Thermo Fisher Scientific	Cat# EP0442
Phosphatase inhibitor cocktail 2	Sigma	Cat# P5726
**Software**		
ImageJ	NIH	RRID:SCR_002285
Prism 9	GraphPad	RRID:SCR_002798
Zen Software Version 3.4	Zeiss	RRID:SCR_013672
Adobe Illustrator cc 2018	Adobe	RRID:SCR_010279
Microsoft Word, Excel, PowerPoint	Microsoft 2019	Microsoft 2019

### *Drosophila* culture media and fly stocks

*Drosophila* stocks were cultured using standard fly medium comprising 46 g/L cornmeal, 45 g/L sucrose, 18 g/L yeast extract, 7 g/L agar, supplemented with 3 mL/L propionic acid, and 3 g/L p-hydroxybenzoic acid methyl ester. The following *Drosophila* stocks used for this study were *dome*^*MESO*^*-Gal4 UAS-EGFP*, *Hml*^*Δ*^*-Gal4 UAS-EGFP*, *CHIZ-Gal4 UAS-mGFP*, *dome*^*MESO*^*-Gal4*, and *HHLT* from Utpal Banerjee’s lab, *Nplp2-Gal4* from Jiwon Shim’s lab, and *e33c-Gal4* from Maneesha Inamdar’s lab. The following fly lines were obtained from Bloomington *Drosophila* Stock Center (BDSC): *UAS-mCherry*^*RNAi*^ (BL35785), *UAS-CSN1b*^*RNAi*^ (BL27303), *UAS-CSN2/Alien*^*RNAi*^ (BL28908), *UAS-CSN3*^*RNAi*^ (BL33369), *UAS-CSN4*^*RNAi*^ (BL42798), *UAS-CSN5*^*RNAi*^ (BL28732, BL42781, BL62970), *UAS-CSN6*^*RNAi*^ (BL36073, BL41991), *UAS-CSN7*^*RNAi*^ (BL33663), *UAS-Ser* (BL5815), *UAS-Ser*^*DN*^ (BL52009), *UAS-Su(H)*^*RNAi*^ (BL28900), *UAS-H*^*RNAi*^ (BL27315), *UAS-mib1*^*RNAi*^ (BL27320), *UAS-neur*^*RNAi#1*^ (BL26023), *Su(H)-lacZ* (BL10689)*, UAS-Nedd8*^*RNAi*^ (BL33881), *UAS-Cul1*^*RNAi*^ (BL36601, BL29520), *Ser-EGFP* (BL59824), *UAS-EGFR*^*RNAi*^ (BL31525, BL60012), *UAS-EGFR*^*DN*^ (BL5364), *UAS-EGFR*^*CA*^ (BL9533), *EGFR-sfGFP* (BL92329), *UAS-rolled*^*RNAi*^ (BL34855), *UAS-cic*^*RNAi*^ (BL25995), *UAS-pnt*^*RNAi*^ (BL31936), *UAS-pnt*^*OE*^ (BL869), *UAS-yan*^*RNAi*^ (BL35404), *UAS-yan*^*Act*^ (BL5789), *UAS-GC3Ai* (BL84346), *E(spl)mß-HLH-GFP* (BL65294), *NRE-GFP* (BL30727), *cic-GFP* (BL42267), and *pnt-GFP* (BL42680). The following stocks were obtained from the Vienna *Drosophila* Research Center (VDRC): *UAS-CSN6*^*RNAi*^ (v100377), *UAS-CSN8*^*RNAi*^ (v50565), *UAS-Ser*^*RNAi*^ (v27172), *UAS-neur*^*RNAi*^ (v108239), and *Nedd8*^*RNAi*^ (v28445). The following stocks were a kind gift from Spyros Artavanis-Tsakonas, USA: *UAS-Notch*^*RNAi*^*, UAS-Notch*^*ICD*^, Cheng-Ting Chien, Taiwan: *UAS-CSN5*, *UAS-CSN5*^*D148N*^, and Robert Schulez, USA: *BcF6-mCherry*.

All stocks and crosses were reared at 25 °C except those used in Gal4-UAS-based expression experiments. The crosses involving RNAi lines were maintained at 29 °C to increase the efficacy of the Gal4-UAS system. In most experiments, lymph glands from wandering third-instar larvae were used; however, in some specific experiments, lymph glands from early stages were used, and their exact ages were mentioned. Genetic crosses were set using one-day-old virgin female and male after eclosion. The lymph glands of both sexes were used, and our study could not differentiate between the two.

### Methods details

#### *Drosophila* larval lymph gland dissection and Immunohistochemistry

Wandering third instar larval lymph glands were used for this study. The lymph glands were dissected in cold 1xPBS (phosphate-buffered saline). After dissection, the tissues were fixed in 4% paraformaldehyde (PFA, Thermo Fisher Scientific, Cat# 28908) for 30 min before being transferred into microtubes for further processing. All the following steps were performed with gentle oscillation in a tube rotator. After fixation the tissues were washed thrice with 0.3% PBST (0.3% Triton X-100 in 1X PBS) for 15 min each, blocked in blocking solution (0.1% Triton X-100, 0.1% BSA, 10% FCS, 0.1% sodium deoxycholate and 0.02% Thiomersal, in 1XPBS) for 30 min and then incubated in primary antibody (diluted in blocking solution) at 4 °C overnight. To label *Drosophila* pERK, the lymph glands were dissected in Schneider’s media, followed by fixation in 4% PFA for 30 min, washed thrice in 0.3% PBST, blocked and incubated with primary antibody at 4 °C overnight; each step supplemented with phosphatase inhibitor cocktail 2 (1:100, Sigma, cat#P5726). The next day, tissues were washed three times with 0.3% PBST, followed by reblocking with the blocking solution for 30 min, then the tissues were incubated with secondary antibody for 2 h at room temperature. After secondary antibody incubation, the tissues were again washed in 0.3% PBST three times, followed by counterstaining with DAPI (4’,6-Diamidino-2-Phenylindole, Dihydrochloride, Thermo Fisher Scientific, Cat# D1306) (1 μg/ml). Slides were mounted in DABCO (1,4-diazabicyclo [2.2.2] octane, Sigma, Cat# D27802, 2.5% DABCO in 70% glycerol made in 1× PBS)(Maurya et al, 2024) anti-fade medium prior imaging.

The following primary antibodies were used for immunohistochemistry: mouse anti-Hnt (1:10, 1G9, DSHB), mouse anti-P1 (1:200, I. Ando), rabbit anti-PPO2 (1:2500, T. Asano), mouse anti-Mys (1:10, CF.6G11, DSHB), chicken anti-GFP (1:200, ab13970, Abcam), rabbit anti-GFP (1:200, A11122, Invitrogen), chicken anti- β-Galactosidase (1:500, ab134435, Abcam), mouse anti- β-Galactosidase (1:1000, Z3781, Promega), rabbit anti-Phospho-Histone H3 (1:400, 9701, CST), rat anti-DE-cadherin extracellular domain (1:10, DCAD2, DSHB), mouse anti-NICD (1:10, C17.9C6, DSHB), mouse anti-Lz (1:10 pre-absorbed, anti-lozenge, DSHB), rat anti-Ci (1:10, 2A1, DSHB) mouse anti-EGFR (1:100, E2906, Sigma), rabbit anti-pERK (1:100, 4370, CST), mouse anti-Yan (1:10 anti-Yan 8B129, DSHB), and anti-Cul1 (1:100, Cat# 71-8700, Thermo Fisher Scientific).

The secondary antibodies used for the immunohistochemistry are as follows: donkey anti-mouse AlexaFluor555, goat anti-mouse Alexa-Fluor 647, donkey anti-rabbit AlexaFluor555, goat anti-rabbit AlexaFluor647, donkey anti-rabbit AlexaFluor488, goat anti-chicken AlexaFluor488, goat anti-chicken AlexaFluor546, goat anti-Rat Alexa-Fluor 546 and Goat anti-and goat anti-mouse Cy3 from Jackson Immuno Research. All secondary antibodies were used at a 1:200 dilutions.

#### Microscopy and image processing

All confocal images were acquired using a Zeiss LSM-900 confocal microscope using Zen software (version 3.4) under a ×20 objective with a zoom of 1.0 and a ×40 objective with a zoom of 0.5 and used a 2.0 μm optical section interval in all images otherwise specified in the figure legend. The imaging settings were kept identical for both control and experimental samples related to an experiment. Image processing was done using ImageJ software (NIH, USA) (available at ImageJ.nih.gov/ij). For better representation of the lymph gland, most of the images are maximum intensity projections of the middle third stack of lymph gland lobes that were used to allow visibility of the inside of the lymph gland. However, some of the images are single optical sections, which is mentioned in the figure legend. The lymph glands boundary is demarcated by a white dotted line for clarity(Maurya et al, 2024). Adobe Illustrator cc 2018 (version 22.1) was used to make the figure panel and for model preparation. A Sony Digital Camera (DSC-75) attached to a Zeiss Stemi SV6 stereo binocular microscope was used to image *Drosophila* wings.

#### Larval staging

Synchronized first-instar larvae were collected right after they hatched. The flies were allowed to lay eggs for 12 h on egg-laying plates to ensure synchronization. After 12 h of egg collection, the embryos were incubated at 25 °C for another 12 h. Following this incubation, the hatched larvae were removed from the plate using a paintbrush, leaving behind unhatched embryos. The remaining unhatched embryos were incubated at 25 °C for 30 min. Newly hatched larvae were carefully transferred to fresh food vials and placed in a 29 °C incubator. Different stages of larvae at 36 h after larval hatching (ALH), 42 h ALH, 48 h ALH, 60 h ALH, and 72 h ALH were collected for dissection (Mondal et al, 2011) and Hnt staining. Lymph glands were isolated, and immunostaining was performed as described in the “Immunostaining” section.

#### Quantification of lymph gland phenotypes

ImageJ software was used to quantify all the images. The methodology for the quantification of various parameters is given below:

#### Analysis of nuclear/cellular markers-positive cells

The numbers of crystal cells (Hnt, Lz, PPO2), Notch reporters (NRE-GFP, Su(H)-lacZ and mβ-GFP), pERK, and pH3-positive cells were counted manually across the confocal Z stack for both lobes of the primary lymph gland and analyzed separately. For volume analysis of the lymph gland lobe, a specific threshold was chosen tailored to the actual staining and remained constant throughout the analysis. Next, the lymph gland zone was outlined using the free hand selection tool, followed by employing the “measure stack” plugin to find the fluorescent area (DAPI for the whole lymph gland and GFP for the intermediate progenitors or progenitors) of each lymph gland lobe via the “analyze → measure” option, where area, mean grey value, stack position, and limit to threshold checkboxes were selected (Maurya et al, 2024). The area obtained of each optical section were added and multiplied by the number of stack intervals ( = 2 μm) to determine the volume of respective volume staining of the lymph gland. Crystal cell index, plasmatocyte, and progenitor index were calculated as described below(Destalminil-Letourneau et al, 2021; Morin-Poulard et al, 2016):

Notch reporter/pH3/crystal cell or pERK indexes correspond to (Number of marked cells/anterior lobe volume) ×10,000.

Plasmatocyte or progenitor indexes correspond to [(plasmatocyte or progenitor volume/anterior lobe volume)] ×10. To quantify the plasmatocyte or progenitor index, their total volume was measured instead of cell number, since their cytoplasmic and membranous expression patterns make individual counting challenging.

#### FUCCI positive cells analysis

G1, S and G2/M positive cells in the progenitor population were estimated as shown in Kapoor et al, 2022 (Kapoor et al, 2022). Briefly, middle two Z stacks were merged, which best represented the medullary zone of the lymph gland. Following this, the channels of the merged images were separated to analyze the green and red cells separately. For this, Fluorescence Ubiquitin Cell Cycle Indicator (FUCCI) reporter-positive cells (green/magenta) were thresholded, followed by “watershed” and “analyze particles” commands to obtain the number of green and magenta-positive cells, individually. To estimate the white cells, the ROI of the green cells was overlaid on the magenta cells, and the “green only” cells were manually counted. This “green only” count represents the number of cells in the G1 phase. To obtain the number of white cells which depicts the number of cells in G2/M phase green positive cells were subtracted from Green positive cells. The “magenta only” cells which represent the cells in S phase was acquired by eliminating white cells from total red cells counted. These values were the normalized to the total FUCCI positive cells of the merged stacks and represented as %G1, %S or %G2/M population.

#### Intensity analysis

Mean fluorescence intensities of E-Cad, Ci, and Mys in lymph gland tissues were calculated as described in literature (Kapoor et al, 2022; Louradour et al, 2017). In brief, two middle z-stacks from each lymph gland image were selected, and a fixed square region of 30 × 30 μm was used to measure signal intensity using the “Measure” tool. To account for background fluorescence, a 30 × 30 μm square was selected outside the lymph gland area, and the mean background intensity was subtracted from the lymph gland mean intensity. All genotypes were imaged and analyzed in parallel under identical conditions. Laser settings were kept constant within each experimental batch to ensure consistency across measurements.

#### Quantitative real-time PCR analysis

Total RNA was isolated from fifty wandering 3rd instar larval lymph glands using Trizol reagent following the manufacturer’s recommended protocol (Sigma-Aldrich, Cat#T9424). The RNA pellets were resuspended in 15 μl of DEPC-MQ, and their quantitative estimation was performed using spectrophotometric analysis. Subsequently, 1 μg of each RNA sample was incubated with 1U of RNase-free DNase I (Thermo Fisher Scientific, Cat# 89836) for 30 min at 37 °C to eliminate residual DNA. First-strand cDNA was synthesized using the standard cDNA preparation protocol from these incubated samples. The prepared cDNA was then subjected to Real-Time PCR using 5 μl of qPCR master mix (SYBR Green, Genetix, Cat# PKG025-A), 2 pmol/μl of each primer per reaction in 10 μl of the final volume in an ABI 7500 Real-Time PCR machine. To determine changes in gene expression, the relative fold change in mRNA expression for different genes was calculated using the comparative C_T_ method. Data normalization was performed using Rp49 as an internal control. The following primers are used for this study:

*CSN1b* (forward): 5’-CCAATTCGATCAAGGAGAGC-3’

*CSN1b* (reverse): 5’-AATAGCACTTCAGGGCATTG-3’

*CSN2* (forward): 5’-GAGCATCAATTCGATTCTGGACT-3’

*CSN2* (reverse): 5’-GGTGTTGGTCTTAAACCACAGC-3’

*CSN3* (forward): 5’-TCTGTTTACCGAGTTTGTGG-3’

*CSN3* (reverse): 5’-GGGTTTCCAACTGTCGAATC-3’

*CSN4* (forward): 5’-ATCTGGAGGACAACGATTCG-3’

*CSN4* (reverse): 5’-CTGCAACTCCTCGGAATTAG-3’

*CSN5* (forward): 5’-GGTGATGGGTCTAATGCTTGG-3’

*CSN5* (reverse): 5’-CCATGTAGGCGGTCATGTACT-3’

*CSN6* (forward): 5’-TGAAACAGTGATCAACAAGGACTAC-3’

*CSN6* (reverse): 5’-ACCAGCCTATGAAGTCGAGATC-3’

*CSN7* (forward): 5’-TCCAAGCACTTCGAGACC-3’

*CSN7* (reverse): 5’-CAGCTCGATGAACTTTTCCG-3’

*CSN8* (forward): 5’-CCGGAATTCATGCATTTAAATAAATATAGTG-3’

*CSN8* (reverse): 5’-CCCGCTCGAGTTAATTCTCTAGGAAGGTTAC-3’

*Rp49* (forward) 5´-TTGAGAACGCAGGCGACCGT-3´

*Rp49* (reverse) 5´-CGTCTCCTCCAAGAAGCGCAAG-3´.

### Statistical analysis

A minimum of three independent biological replicates were analyzed with at least three experimental repeats, one of which is shown as a representative image. The quantifications shown are for all the sets analyzed. All the statistical analyses were done using GraphPad Prism 9 and Microsoft Excel 2019 software. A two-tailed, unpaired Student’s *t* test was employed to compare the mean value of the two groups. One-way or two-way ANOVA with Tukey’s post-hoc test was used for analysis involving multiple comparisons. *P* values presented represent to determine statistical significance (**** if *P* < 0.0001, *** if *P* < 0.001, ** if *P* < 0.01 and * if *P* < 0.05), and by ns for not significant, *P* ≥ 0.05. We have provided details of the statistical analysis for each set of experimental data, along with the figure number, in the XLSX file in the source data.

### Statement on the experimental design

All experiments represent pooled data conducted using at least three independent crosses. No blinding procedure was applied in any of our experiments. Both male and female flies (adult/larvae) were included in each experiment.

## Supplementary information

Appendix

Peer Review File

Source data Fig. 1

Source data Fig. 2

Source data Fig. 3

Source data Fig. 4

Source data Fig. 5

Source data Fig. 6

Figure EV1 Source Data

Figure EV2 Source Data

Figure EV3 Source Data

Figure EV4 Source Data

Figure EV5 Source Data

Expanded View Figures

## Data Availability

All imaging data generated in this study has been deposited in the BioImage Archive under the accession number S-BIAD3342. The source data of this paper are collected in the following database record: biostudies:S-SCDT-10_1038-S44319-026-00862-w.
