# Supplementary material for: COP9 signalosome regulates EGFR and Notch signaling during myeloid-type progenitor cell fate decision in Drosophila
Source: EMBO Rep. 2026 Jul 7;27(16):4751–81. doi: 10.1038/s44319-026-00862-w (PMC13503907; doi:10.1038/s44319-026-00862-w)
Supplement: Supplementary file 1 [file 44319_2026_862_MOESM14_ESM.pdf]

Expanded View Figures

Figure EV1. COP9 signalosome (CSN) regulates the lymph gland blood progenitors' cell fate, related to Fig. 1.

(**A–G**) Knockdown of CSN complex in lymph gland progenitors (green) using *dome^{MESO}-Gal4, UAS-EGFP* driver by expressing *CSN2^{RNAi}* (**B**), *CSN3^{RNAi}* (**C**), *CSN4^{RNAi}* (**D**), *CSN6^{RNAi}* (**E**), *CSN7^{RNAi}* (**F**), and *CSN8^{RNAi}* (**G**), increases the Hnt positive (magenta) crystal cell number compared to control *w^{1118}* (**A**). The quantification of the crystal cell index is shown in Fig. 1I. (**H–N**) Using *dome^{MESO}-Gal4, UAS-EGFP* driver by expressing *CSN2^{RNAi}* (**I**), *CSN3^{RNAi}* (**J**), *CSN4^{RNAi}* (**K**), *CSN6^{RNAi}* (**L**), *CSN7^{RNAi}* (**M**), and *CSN8^{RNAi}* (**N**) caused reduced plasmatocyte number marked by P1 (magenta) compared to the control lymph gland (*dome^{MESO}-Gal4, UAS-EGFP/+*) (**H**). The quantification of the Plasmatocyte index is shown in Fig. 1O. (**O**) Graph showing the lymph gland size in terms of DAPI volume (μm^3) in Figs. 1D-F,J-L, and EV1A–N. Upon progenitor-specific (*dome^{MESO}-Gal4, UAS-2XEGFP*) knockdown of *CSN* subunits by expressing *CSN1b^{RNAi}* ($n = 13$, $P > 0.9999$), *CSN2^{RNAi}* ($n = 18$, $P = 0.8995$), *CSN3^{RNAi}* ($n = 16$, $P = 0.6736$), *CSN4^{RNAi}* ($n = 16$, $P = 0.4598$), *CSN5^{RNAi}* ($n = 20$, $P = 0.4035$), *CSN6^{RNAi}* ($n = 19$, $P = 0.8429$), *CSN7^{RNAi}* ($n = 18$, $P = 0.9329$), and *CSN8^{RNAi}* ($n = 13$, $P = 0.4753$) is similar when compared to the wild-type lymph gland *w^{1118}* ($n = 15$). (**P**) Progenitor index in (Figs. 1D–F,J–L, and EV1A–N). *w^{1118}* ($n = 27$), *CSN1b^{RNAi}* ($n = 31$, $P = 0.9866$), *CSN2^{RNAi}* ($n = 19$, $P = 0.1888$), *CSN3^{RNAi}* ($n = 20$, $P = 0.4614$), *CSN4^{RNAi}* ($n = 15$, $P > 0.9999$), *CSN5^{RNAi}* ($n = 16$, $P > 0.9999$), *CSN6^{RNAi}* ($n = 16$, $P = 0.5798$), *CSN7^{RNAi}* ($n = 18$, $P = 0.9878$) and *CSN8^{RNAi}* ($n = 16$, $P = 0.9793$). (**Q**) Quantitative RT-PCR showing significant reduction in the transcript level of CSNs in respective *CSN^{RNAi}* background when expressed using pan-lymph gland Gal4 driver *e33c-Gal4* (*e33c-Gal4 UAS-CSN1b^{RNAi}*) (BL 27303) ($n = 3$, $P < 0.0001$), *e33c-Gal4 UAS-CSN2^{RNAi}* (BL 28908) ($n = 3$, $P = 0.0004$), *e33c-Gal4, UAS-CSN3^{RNAi}* (BL 33369) ($n = 3$, $P < 0.0001$), *e33c-Gal4 UAS-CSN4^{RNAi}* (BL 42798) ($n = 3$, $P < 0.0001$), *e33c-Gal4 UAS-CSN5^{RNAi}* (BL 28732) ($n = 3$, $P < 0.0001$), *e33c-Gal4 UAS-CSN6^{RNAi}* (BL 36073) ($n = 3$, $P < 0.0001$), *e33c-Gal4 UAS-CSN7^{RNAi}* (BL 33663) ($n = 3$, $P < 0.0001$)and *e33c-Gal4 UAS-CSN8^{RNAi}*(v50565) ($n = 3$, $P < 0.0001$) when compared to control (*e33c-Gal4*). (**R–T**) Knockdown of CSN in lymph gland core progenitors (green) using *Tep4-Gal4, UAS-2XEGFP* driver by expressing *CSN1b^{RNAi}* (**S**) and *CSN5^{RNAi}* (**T**) does not show any significant difference in the plasmatocyte numbers marked by anti-P1 (magenta) compared to control (*Tep4-Gal4, UAS-EGFP/+*) (**R**). (**U**) Plasmatocyte index in (**R–T**): *w^{1118}* ($n = 10$), *CSN1b^{RNAi}* ($n = 17$, $P = 0.4335$), *CSN5^{RNAi}* ($n = 12$, $P = 0.8359$). (**V**) Core progenitor index in (Figs. 1IZA–ZC and EV1R–T). *w^{1118}* ($n = 21$), *CSN1b^{RNAi}* ($n = 17$, $P = 0.3448$), *CSN5^{RNAi}* ($n = 12$, $P = 0.934$). (**W–Y**) Knockdown of CSN using a differentiated zone driver *Hml$^{\Delta}$-Gal4, UAS-EGFP* by expressing *CSN1b^{RNAi}* (**X**), and *CSN5^{RNAi}* (**Y**) resulted in a drastic decrease in plasmatocyte numbers (magenta) as compared to control (**W**). (**Z**) Plasmatocyte index in (W-Y): *w^{1118}* ($n = 25$), *CSN1b^{RNAi}* ($n = 18$, $P < 0.0001$), *CSN5^{RNAi}* ($n = 19$, $P < 0.0001$). All confocal images represent maximum-intensity projections of the middle-third optical sections of the wandering third-instar anterior lymph gland lobe from at least three independent biological experiments. White dashed lines demarcate one lymph gland lobe. Scale bar, 25 μm. Error bars in graphs: mean ± S.D. n.s.: not significant ($P \geq 0.05$); ***$P < 0.001$; ****$P < 0.0001$. A one-way ANOVA with Tukey's post-hoc test was used for analysis involving (**O**, **P**, **U**, **V**, **Z**). A two-tailed, unpaired Student's *t* test was employed to compare the mean value of the two groups in (**Q**). Source data are available online for this figure.

 © The Author(s)

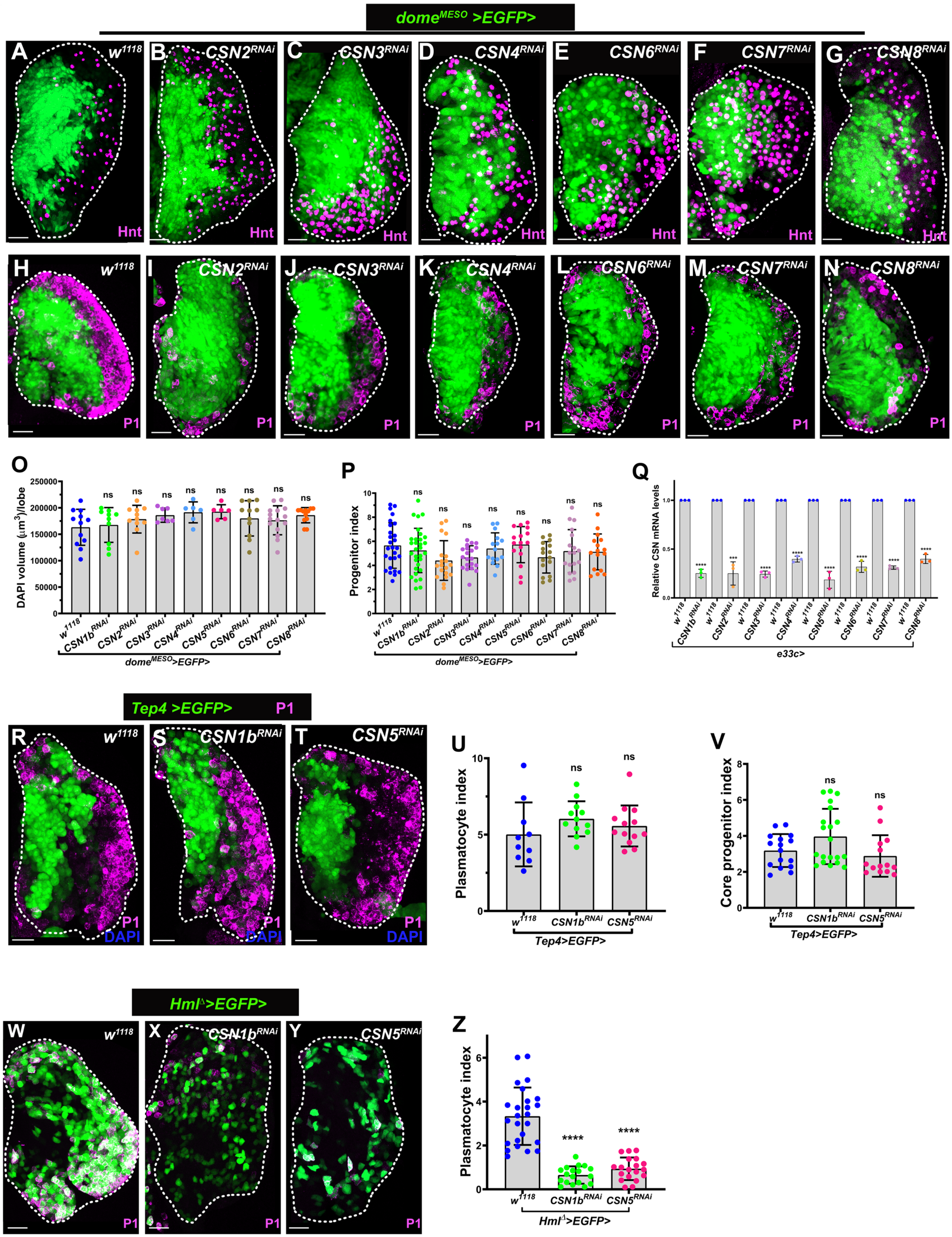
dome^MESO >EGFP>
A w1118
B CSN2RNAi
C CSN3RNAi
D CSN4RNAi
E CSN6RNAi
F CSN7RNAi
G CSN8RNAi
Hnt
H w1118
I CSN2RNAi
J CSN3RNAi
K CSN4RNAi
L CSN6RNAi
M CSN7RNAi
N CSN8RNAi
P1
O
DAPI volume (μm3)/lobe
dome^MESO>EGFP>
P
Progenitor index
Q
Relative CSN mRNA levels
e33c>
Tep4 >EGFP> P1
R w1118
S CSN1bRNAi
T CSN5RNAi
DAPI
U
Plasmatocyte index
Tep4>EGFP>
V
Core progenitor index
Hml^Δ>EGFP>
W w1118
X CSN1bRNAi
Y CSN5RNAi
Z
Plasmatocyte index

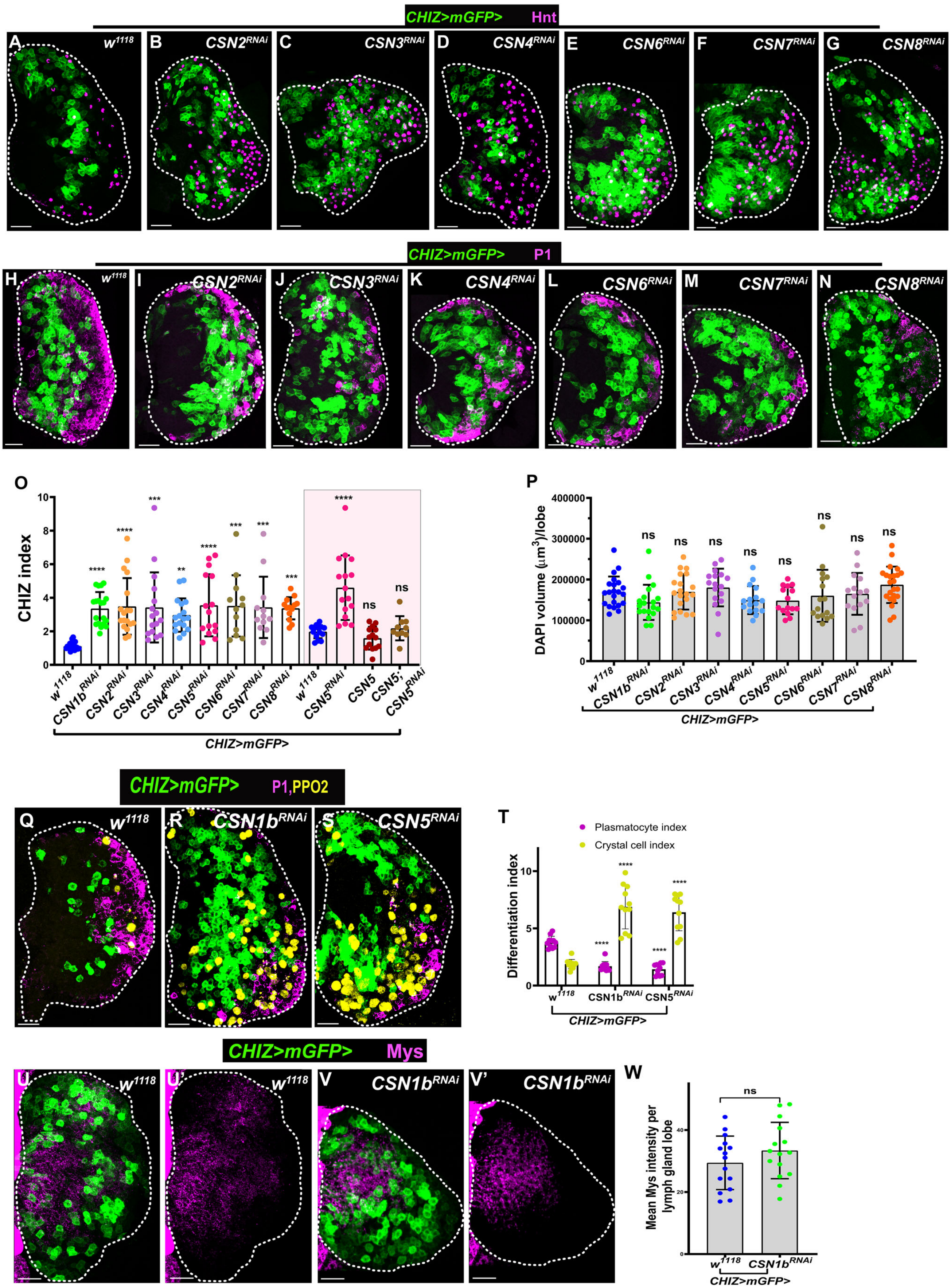

CHIZ>mGFP> Hnt
A w1118
B CSN2RNAi
C CSN3RNAi
D CSN4RNAi
E CSN6RNAi
F CSN7RNAi
G CSN8RNAi
CHIZ>mGFP> P1
H w1118
I CSN2RNAi
J CSN3RNAi
K CSN4RNAi
L CSN6RNAi
M CSN7RNAi
N CSN8RNAi
O
CHIZ index
w1118 CSN1bRNAi CSN2RNAi CSN3RNAi CSN4RNAi CSN5RNAi CSN6RNAi CSN7RNAi CSN8RNAi w1118 CSN5RNAi CSN5 CSN5; CSN5RNAi
CHIZ>mGFP>
P
DAPI volume (µm3)/lobe
w1118 CSN1bRNAi CSN2RNAi CSN3RNAi CSN4RNAi CSN5RNAi CSN6RNAi CSN7RNAi CSN8RNAi
CHIZ>mGFP>
CHIZ>mGFP> P1,PPO2
Q w1118
R CSN1bRNAi
S CSN5RNAi
T
Plasmatocyte index
Crystal cell index
Differentiation index
w1118 CSN1bRNAi CSN5RNAi
CHIZ>mGFP>
CHIZ>mGFP> Mys
U w1118
U' w1118
V CSN1bRNAi
V' CSN1bRNAi
W
Mean Mys intensity per lymph gland lobe
w1118 CSN1bRNAi
CHIZ>mGFP>

Figure EV2. CSN is required during the myeloid-type intermediate progenitor cell fate decision, related to Fig. 2.

(**A–G**) Knockdown of CSN complex in lymph gland progenitors (green) using *CHIZ-Gal4, UAS-mGFP* driver by expressing $CSN2^{RNAi}$ (**B**), $CSN3^{RNAi}$ (**C**), $CSN4^{RNAi}$ (**D**), $CSN6^{RNAi}$ (**E**), $CSN7^{RNAi}$ (**F**) and $CSN8^{RNAi}$ (**G**), increases the Hnt positive (magenta) crystal cell number compared to control w^{1118} (**A**). (**H–N**) Using *CHIZ-Gal4, UAS-mGFP* driver by expressing $CSN2^{RNAi}$ ($n = 18$, $P < 0.0001$) (**I**), $CSN3^{RNAi}$ (**J**) $CSN4^{RNAi}$ (**K**), $CSN6^{RNAi}$ (**L**), $CSN7^{RNAi}$ (**M**) and $CSN8^{RNAi}$ (**N**) caused reduced plasmatocytes number marked by P1 (magenta) compared to control lymph gland (*CHIZ-Gal4, UAS-mGFP/+*) (**H**). (**O**) Intermediate progenitors index in (**B–J**): w^{1118} ($n = 21$), $CSN1b^{RNAi}$ ($n = 19$, $P < 0.0001$), $CSN2^{RNAi}$ ($n = 18$, $P < 0.0001$), $CSN3^{RNAi}$ ($n = 16$, $P = 0.0001$), $CSN4^{RNAi}$ ($n = 17$, $P = 0.0039$), $CSN5^{RNAi}$ ($n = 15$, $P < 0.0001$), $CSN6^{RNAi}$ ($n = 12$, $P = 0.0003$), $CSN7^{RNAi}$ ($n = 12$, $P = 0.0006$) and $CSN8^{RNAi}$ ($n = 13$, $P = 0.0006$). w^{1118} ($n = 14$), $CSN5^{RNAi}$ ($n = 16$, $P < 0.0001$), *CSN5* ($n = 14$, $P > 0.812$), and $CSN5;CSN5^{RNAi}$ ($n = 10$, $P = 0.9758$). (**P**) Graph showing the lymph gland size in terms of DAPI volume (μm^3) upon intermediate progenitor-specific knockdown of CSN, shown in Figs. 2A–C,G–I and EV2A–N. The lymph gland size upon expression of $CSN1b^{RNAi}$ ($n = 19$, $P = 0.6699$), $CSN2^{RNAi}$ ($n = 20$, $P > 0.9999$), $CSN3^{RNAi}$ ($n = 17$, $P = 0.998$); $CSN4^{RNAi}$ ($n = 16$, $P = 0.9078$), $CSN5^{RNAi}$ ($n = 15$, $P = 0.8868$), $CSN6^{RNAi}$ ($n = 16$, $P = 0.9994$), $CSN7^{RNAi}$ ($n = 15$, $P > 0.9999$), and $CSN8^{RNAi}$ ($n = 23$, $P = 0.9263$) is nearly similar when compared to the wild-type lymph gland w^{1118} ($n = 22$). (**Q–S**) Knockdown of CSN complex in lymph gland progenitors (green) using *CHIZ-Gal4, UAS-mGFP* driver by expressing $CSN1b^{RNAi}$ (**R**), and $CSN5^{RNAi}$ (**S**), increases the PPO2 positive (yellow) crystal cells and decreases P1 positive (magenta) plasmatocytes compared to control w^{1118} (**Q**). (**T**) Plasmatocyte and crystal cell index in (**Q–S**). $CSN1b^{RNAi}$ ($n = 11$, $P < 0.0001$), $CSN5^{RNAi}$ ($n = 11$, $P < 0.0001$) and w^{1118} ($n = 11$). (**U–V′**) Intermediate progenitor-specific knockdown of *CSN1b* (*CHIZ-Gal4 UAS-mGFP; $CSN1b^{RNAi}$*) (**V–V′**) does not induce any lamellocyte differentiation and looks similar to the wild-type lymph gland (*CHIZ-Gal4 UAS-mGFP*) (**U–U′**). (**W**) Mean Mys (lamellocyte marker) fluorescent intensity per lymph gland lobe in (**U–V′**). $CSN1b^{RNAi}$ ($n = 14$, $P = 0.2305$) and w^{1118} ($n = 15$). All confocal images represent maximum-intensity projections of the middle-third optical sections of the wandering third-instar anterior lymph gland lobe from at least three independent biological experiments. White dashed lines demarcate one lymph gland lobe. Scale bar, 25 µm. Each data point represents the number of lymph gland lobes analyzed. Error bars in graphs: mean ± S.D. n.s.: not significant ($P \geq 0.05$); ****$P < 0.0001$. A one-way ANOVA with Tukey's post-hoc test was used for analysis involving (**O**, **P**). A two-way ANOVA with Tukey's post-hoc test was used for analysis involving (**T**). A two-tailed, unpaired Student's *t* test was employed to compare the mean value of the two groups in (**W**). Source data are available online for this figure.

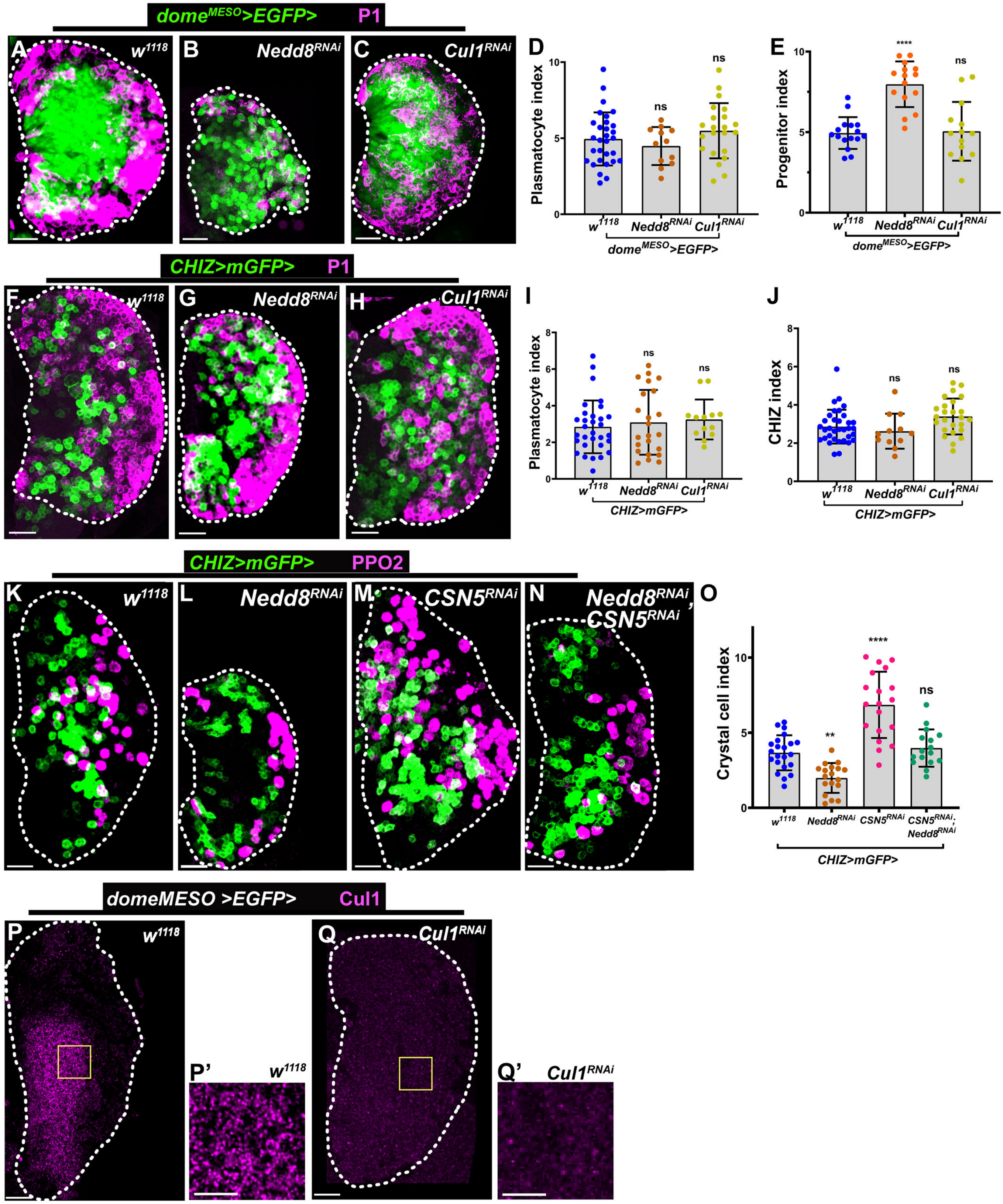

dome^{MESO}>EGFP> P1
A w1118
B Nedd8RNAi
C Cul1RNAi
D
Plasmatocyte index
ns
ns
w1118 Nedd8RNAi Cul1RNAi
domeMESO>EGFP>
E
Progenitor index
****
ns
w1118 Nedd8RNAi Cul1RNAi
domeMESO>EGFP>
CHIZ>mGFP> P1
F w1118
G Nedd8RNAi
H Cul1RNAi
I
Plasmatocyte index
ns
ns
w1118 Nedd8RNAi Cul1RNAi
CHIZ>mGFP>
J
CHIZ index
ns
ns
w1118 Nedd8RNAi Cul1RNAi
CHIZ>mGFP>
CHIZ>mGFP> PPO2
K w1118
L Nedd8RNAi
M CSN5RNAi
N Nedd8RNAi, CSN5RNAi
O
Crystal cell index
**
****
ns
w1118 Nedd8RNAi CSN5RNAi CSN5RNAi; Nedd8RNAi
CHIZ>mGFP>
domeMESO >EGFP> Cul1
P w1118
P' w1118
Q Cul1RNAi
Q' Cul1RNAi

 © The Author(s)

Figure EV3. Involvement of Cullin-1 in CSN-mediated cell fate decision in the lymph gland, related to Fig. 3.

(**A–C**) Knockdown of Nedd8 and Cullin-1 in lymph gland progenitors (green) using *dome^MESO^-Gal4, UAS-EGFP* driver by expressing *Nedd8^RNAi^* (**B**) and *Cul1^RNAi^* (**C**) shows plasmatocytes marked by P1(magenta) compared to control (*dome^MESO^-Gal4 UAS-EGFP/+*) (**A**). (**D**) Plasmatocyte index in (**A–C**). w^{1118} ($n = 31$), $Nedd8^{RNAi}$ ($n = 12$, $P = 0.6998$), and $Cul1^{RNAi}$ ($n = 22$, $P = 0.4967$). (**E**) Progenitor index in (**A–C**). w^{1118} ($n = 16$), $Nedd8^{RNAi}$ ($n = 15$, $P < 0.0001$), and $Cul1^{RNAi}$ ($n = 14$, $P = 0.9766$). (**F–H**) Knockdown of Nedd8 and Cullin-1 in lymph gland intermediate progenitors (green) using *CHIZ-Gal4, UAS-mGFP* driver by expressing $Nedd8^{RNAi}$ (**G**), and $Cul1^{RNAi}$ (**H**) resulted in a similar number of plasmatocytes marked by P1 (magenta) compared to control (*CHIZ-Gal4, UAS-mGFP/+*) (**F**). (**I**) Plasmatocyte index in (**F–H**). w^{1118} ($n = 31$), $Nedd8^{RNAi}$ ($n = 23$, $P = 0.8285$), and $Cul1^{RNAi}$ ($n = 13$, $P = 0.703$). (**J**) CHIZ index in (**F–H**): w^{1118} ($n = 35$), $Nedd8^{RNAi}$ ($n = 15$, $P = 0.7161$), and $Cul1^{RNAi}$ ($n = 25$, $P = 0.0751$). (**K–N**) Depletion of Nedd8 in the $CHIZ^+$ progenitor (green) decreased the number of crystal cells (magenta) (**L**) compared to control (**K**). Depletion of CSN5 (**M**) in the background of $Nedd8^{RNAi}$ in the intermediate progenitor rescued $CSN5^{RNAi}$-mediated increase in crystal cell number (**N**). (**O**) Crystal cell index in (**K–N**). w^{1118} ($n = 22$), $Nedd8^{RNAi}$ ($n = 19$, $P = 0.0037$), $CSN5^{RNAi}$ ($n = 19$, $P < 0.0001$), and $Nedd8^{RNAi}$; $CSN5^{RNAi}$ ($n = 16$, $P = 0.9165$). (**P–Q′**) Decreased Cul1 staining in the lymph gland (*dome^MESO^>Cul1^RNAi^*) (**Q–Q′**) compared to control (*dome^MESO^-Gal4 UAS-EGFP/+*) (**P–P′**) using anti-Cul1 immunostaining. All confocal images represent maximum-intensity projections of the middle-third optical sections of the wandering third-instar lymph gland lobe from at least three independent biological experiments. Scale bar, 25 µm, except (**P′**, **Q′**), where the scale bar is 10 µm and is a single optical section. Each data point represents the number of lymph gland lobes analyzed. Error bars in graphs: mean ± S.D. n.s.: not significant ($P \geq 0.05$); $^{**}P < 0.01$; and $^{****}P < 0.0001$. A one-way ANOVA with Tukey's post-hoc test was used for analysis involving (**D**, **E**, **I**, **J**, **O**). Source data are available online for this figure.

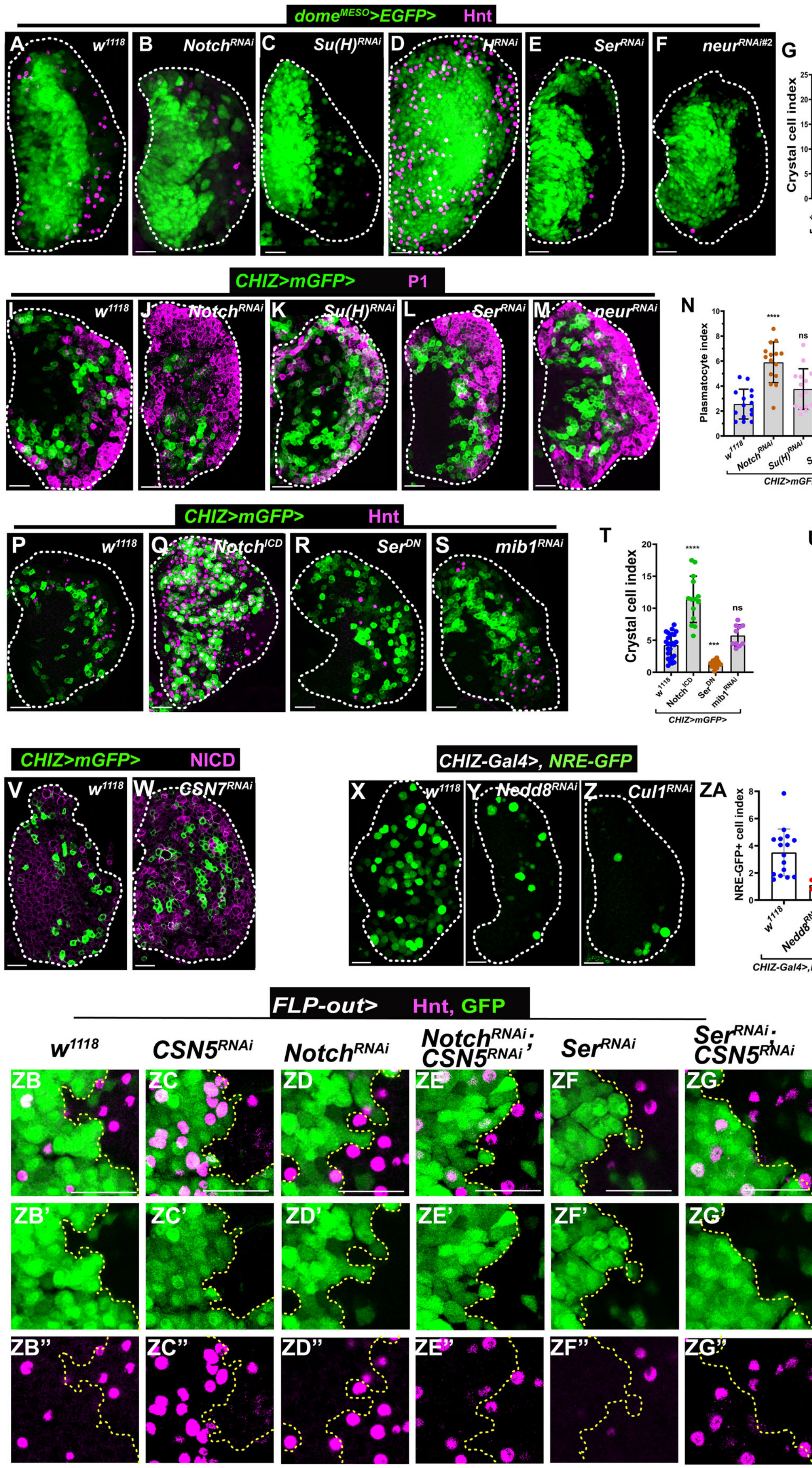
dome^MESO>EGFP> Hnt
A w1118
B Notch^RNAi
C Su(H)^RNAi
D H^RNAi
E Ser^RNAi
F neur^RNAi#2
G
Crystal cell index
H
Progenitor index
dome^MESO-Gal4>EGFP>
CHIZ>mGFP> P1
I w1118
J Notch^RNAi
K Su(H)^RNAi
L Ser^RNAi
M neur^RNAi
N
Plasmatocyte index
O
CHIZ index
CHIZ>mGFP>
CHIZ>mGFP> Hnt
P w1118
Q Notch^ICD
R Ser^DN
S mib1^RNAi
T
Crystal cell index
U
CHIZ index
CHIZ>mGFP> NICD
V w1118
W CSN7^RNAi
CHIZ-Gal4>, NRE-GFP
X w1118
Y Nedd8^RNAi
Z Cul1^RNAi
ZA
NRE-GFP+ cell index
CHIZ-Gal4>,NRE-GFP
FLP-out> Hnt, GFP
w1118
CSN5^RNAi
Notch^RNAi
Notch^RNAi; CSN5^RNAi
Ser^RNAi
Ser^RNAi; CSN5^RNAi
ZB ZC ZD ZE ZF ZG
ZB' ZC' ZD' ZE' ZF' ZG'
ZB'' ZC'' ZD'' ZE'' ZF'' ZG''

 © The Author(s)

Figure EV4. CSN modulates Neuralized/Serrate-mediated Notch activity in developing lymph gland cells, related to Fig. 4.

(**A–F**) Knockdown of Notch-Serrate signaling pathway components in lymph gland progenitors (green) using $dome^{MESO}$*-Gal4, UAS-EGFP* driver by expressing $Notch^{RNAi}$ (**B**), $Su(H)^{RNAi}$ (**C**), Ser^{RNAi} (**E**), and $neur^{RNAi\#2}$ (V108239) (**F**) resulted in very few crystal cells, while knockdown of Notch negative regulator Hairless by expressing H^{RNAi} (**D**) caused a dramatic increase in crystal cells marked by Hnt positive cells (magenta) compared to the control ($dome^{MESO}$*-Gal4, UAS-EGFP/+*) (**A**). (**G**) Crystal cell index in (**A–F**). $Notch^{RNAi}$($n = 15$, $P = 0.0057$), $Su(H)^{RNAi}$ ($n = 15$, $P = 0.0007$), H^{RNAi} ($n = 11$, $P < 0.0001$), Ser^{RNAi} ($n = 16$, $P = 0.0005$), $neur^{RNAi\#2}$($n = 24$, $P < 0.0001$) and w^{1118} ($n = 25$). (**H**) Progenitor index in (**A–F**). $Notch^{RNAi}$ ($n = 14$, $P < 0.0001$), $Su(H)^{RNAi}$ ($n = 14$, $P = 0.0004$), H^{RNAi} ($n = 15$, $P = 0.0001$), Ser^{RNAi} ($n = 15$, P = 0.0013), $neur^{RNAi\#2}$ ($n = 15$, $P = 0.0008$) and w^{1118} ($n = 15$). (**I–M**) Knockdown of Notch-Serrate signaling pathway components in lymph gland intermediate progenitors (green) using *CHIZ-Gal4, UAS-mGFP* driver by expressing $Notch^{RNAi}$ (**J**) and $neur^{RNAi}$ (**M**) resulted in higher plasmatocyte number, while $Su(H)^{RNAi}$ (**K**), Ser^{RNAi} (**L**) caused no significant change in plasmatocyte number compared to the control (*CHIZ-Gal4, UAS-mGFP /+*) (**I**). (**N**) Plasmatocyte index in (**I–M**). w^{1118} ($n = 15$), $Notch^{RNAi}$ ($n = 15$, $P < 0.0001$), $Su(H)^{RNAi}$ ($n = 15$, $P = 0.1898$), Ser^{RNAi} ($n = 16$, $P = 0.8407$), and $neur^{RNAi}$ ($n = 17$, $P < 0.0001$). (**O**) CHIZ index in (**I–M**). w^{1118} ($n = 25$), $Notch^{RNAi}$ ($n = 17$, $P = 0.3425$), $Su(H)^{RNAi}$ ($n = 17$, $P = 0.0689$), Ser^{RNAi} ($n = 23$, $P = 0.661$) and $neur^{RNAi}$ ($n = 17$, $P = 0.0869$). (**P–S**) Alteration of Notch-Serrate signaling pathway components in lymph gland intermediate progenitors (green) using *CHIZ-Gal4, UAS-mGFP* driver by expressing $Notch^{ICD}$ (**Q**) leads to increased crystal cells marked by Hnt (magenta), reduced by expressing Ser^{DN} (**R**), and no change by expressing $mib1^{RNAi}$ (**S**) as compared to wild-type lymph gland (*CHIZ-Gal4, UAS-mGFP*) (**P**). (**T**) Crystal cell index in (**P–S**). w^{1118} ($n = 25$), $Notch^{ICD}$ ($n = 14$, $P < 0.0001$), Ser^{DN} ($n = 17$, $P = 0.0003$), and $mib1^{RNAi}$ ($n = 10$, $P = 0.2454$). (**U**) CHIZ index in (**P–S**): w^{1118} ($n = 13$), $Notch^{ICD}$ ($n = 11$, $P < 0.0001$), Ser^{DN} ($n = 12$, $P = 0.9109$), and $mib1^{RNAi}$ ($n = 12$, $P = 0.4629$). (**V–W**) Knockdown of CSN5 in lymph gland intermediate progenitors using the *CHIZGal4* driver by expressing $CSN7^{RNAi}$ (**W**) resulted in increased NICD expression compared to control (**V**). (**X–Z**) Knockdown of *Nedd8* and *Cullin1* by expressing $Nedd8^{RNAi}$ (**Y**) and $Cul1^{RNAi}$ (**Z**) using the *CHIZ-Gal4* driver resulted in decreased expression of Notch activity reporters marked by *NRE-GFP* as compared to control (**X**). (**ZA**) NRE-GFP+ cell index in (**X–Z**). w^{1118} ($n = 16$), $Nedd8^{RNAi}$ ($n = 17$, $P < 0.0001$), and $Cul1^{RNAi}$ ($n = 17$, $P = 0.0003$). (**ZB–ZG″**) Mock FLP-out Gal4-expressing clones (GFP-positive cells, green) show no reduction in the crystal cell number marker by Hnt (magenta, **ZB–ZB′′**), whereas similar clones expressing $Notch^{RNAi}$ and Ser^{RNAi} show a very strong reduction in crystal cell number (**ZD–ZD′′**, **ZF–ZF″**). FLP-out Gal4-expressing clones expressing $CSN5^{RNAi}$ (**ZC–ZC′′**) showed increased crystal cell number. Coexpression of $CSN5^{RNAi}$ with either $Notch^{RNAi}$ (**ZE–ZE′′**) or Ser^{RNAi} (**ZG–ZG′′**) restored crystal cell number to levels comparable to the control (**ZB–ZB″**), showing no reduction. Mock FLP-out Gal4-expressing clones (GFP-positive cells, green) are demarcated with a yellow dashed line. FLP-out clones were made using *Hand-Gal4* Hml^{Δ}*-Gal4UAS-FLP Ay-gal4 UAS-GFP* at 29 °C. All confocal images represent maximum intensity projections of the middle third optical sections of the primary lobe of the lymph gland, which has been outlined in white dashed lines and represented with maximum intensity projections of the middle third optical sections, except (**ZB–ZG″**) images of lymph glands are the single optical section. "*n*" represents the number of lymph gland lobes analyzed. Scale bar, 25 µm. n.s.: not significant ($P \geq 0.05$); **$P < 0.01$; ***$P < 0.001$; ****$P < 0.0001$. Bars in graphs: mean with S.D. A one-way ANOVA with Tukey's post-hoc test was used for analysis involving (**G**, **H**, **N**, **O**, **T**, **U**, **ZA**). Source data are available online for this figure.

© The Author(s)

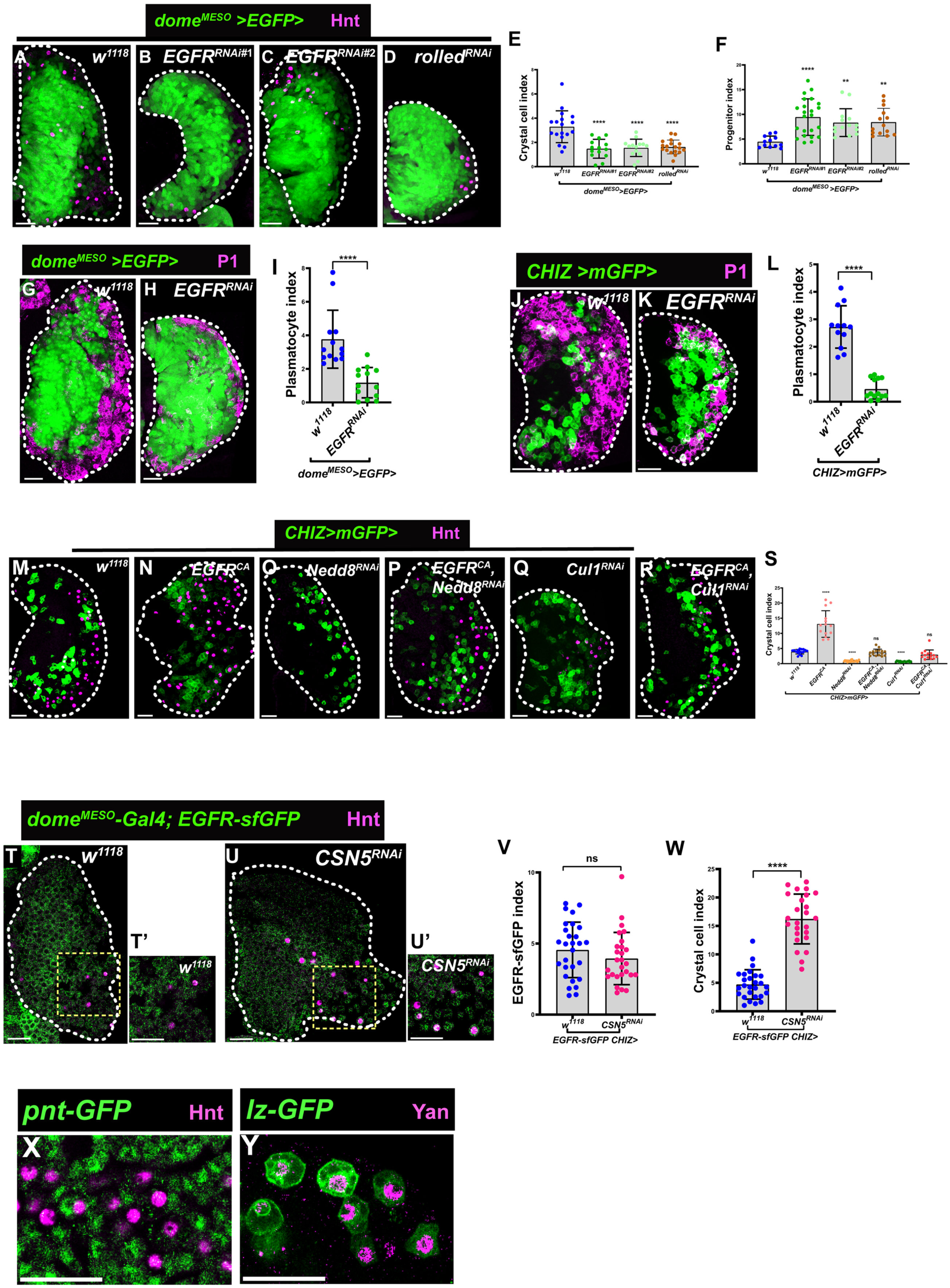
domeMESO >EGFP> Hnt
A w1118
B EGFRRNAi#1
C EGFRRNAi#2
D rolledRNAi
E
Crystal cell index
w1118 EGFRRNAi#1 EGFRRNAi#2 rolledRNAi
domeMESO>EGFP>
F
Progenitor index
w1118 EGFRRNAi#1 EGFRRNAi#2 rolledRNAi
domeMESO >EGFP>
domeMESO >EGFP> P1
G w1118
H EGFRRNAi
I
Plasmatocyte index
w1118 EGFRRNAi
domeMESO>EGFP>
CHIZ >mGFP> P1
J w1118
K EGFRRNAi
L
Plasmatocyte index
w1118 EGFRRNAi
CHIZ>mGFP>
CHIZ>mGFP> Hnt
M w1118
N EGFRCA
O Nedd8RNAi
P EGFRCA, Nedd8RNAi
Q Cul1RNAi
R EGFRCA, Cul1RNAi
S
Crystal cell index
CHIZ>mGFP>
domeMESO-Gal4; EGFR-sfGFP Hnt
T w1118
T' w1118
U CSN5RNAi
U' CSN5RNAi
V
ns
EGFR-sfGFP index
w1118 CSN5RNAi
EGFR-sfGFP CHIZ>
W
Crystal cell index
w1118 CSN5RNAi
EGFR-sfGFP CHIZ>
pnt-GFP Hnt
X
lz-GFP Yan
Y

Figure EV5. CSN is genetically associated with EGFR signaling during lymph gland cell fate decision, related to Fig. 5.

(**A–D**) Knockdown of the EGFR signaling pathway in lymph gland progenitors (green) using *dome^{MESO}-Gal4, UAS-2XEGFP* driver by expressing *EGFR$^{RNAi\#1}$*(BL31525) (**B**), *EGFR$^{RNAi\#2}$*(BL60012) (**C**), and *rolled^{RNAi}* (**D**) leads to decreased crystal cells number marked by Hnt (magenta) compared to wild-type lymph gland (*dome^{MESO}-Gal4, UAS-2XEGFP/+*) (**A**). (**E**) Crystal cell index in (**A–D**). *w^{1118}* ($n = 18$), *EGFR$^{RNAi\#1}$* ($n = 14$, $P < 0.0001$), *EGFR$^{RNAi\#2}$* ($n = 14$, $P < 0.0001$), and *rolled^{RNAi}* ($n = 18$, $P < 0.0001$). (**F**) Progenitor index in (**A–D**). *w^{1118}* ($n = 12$), *EGFR$^{RNAi\#1}$* ($n = 23$, $P < 0.0001$), *EGFR$^{RNAi\#2}$* ($n = 14$, $P = 0.0082$), and *rolled^{RNAi}* ($n = 14$, $P = 0.0065$). (**G**, **H**) Knockdown of the EGFR signaling pathway in lymph gland progenitors (green) using *dome^{MESO}-Gal4, UAS-2XEGFP* driver by expressing *EGFR$^{RNAi\#1}$* (**H**) leads to decreased plasmatocytes marked by P1 (magenta) as compared to wild-type lymph gland (*dome^{MESO}-Gal4, UAS-2XEGFP/+*) (**G**). (**I**) Plasmatocyte index in (**G**, **H**). *w^{1118}* ($n = 13$), and *EGFR$^{RNAi\#1}$* ($n = 13$, $P < 0.0001$). (**J**, **K**) Knockdown of the EGFR signaling pathway in lymph gland core progenitors (green) using *Tep4-Gal4, UAS-EGFP* driver by expressing *EGFR$^{RNAi\#1}$* (**K**) shows no significant change in crystal cell (yellow) and plasmatocytes (magenta) number as compared to wild-type lymph gland (*Tep4-Gal4, UAS-EGFP /+*) (**J**). (**L**) Plasmatocyte index in (**J**, **K**). *w^{1118}* ($n = 12$), and *EGFR^{RNAi}* ($n = 13$, $P < 0.0001$). (**M–R**) Knockdown of *Nedd8* and *Cullin1* by expressing *Nedd8^{RNAi}* (**O**) and *Cul1^{RNAi}* (**Q**) using the *CHIZ-Gal4* driver resulted in decreased crystal cells marked by Hnt (magenta) compared to control (**M**). The increase in the number of crystal cells phenotype in *EGFR^{CA}* (**N**) is rescued by downregulation of *Nedd8* and *Cullin1* in the *EGFR^{CA}* background in the intermediate progenitors by expressing (**P**), and *Cul1^{RNAi}* (**R**). (**S**) Crystal cell index in (**M–R**). *w^{1118}* ($n = 13$), *EGFR^{CA}* ($n = 14$, $P < 0.0001$), *Nedd8^{RNAi}* ($n = 15$, $P < 0.0001$), *EGFR^{CA}; Nedd8^{RNAi}* ($n = 15$, $P = 0.9988$), *Cul1^{RNAi}* ($n = 14$, $P < 0.0001$), and *EGFR^{CA}; Cul1^{RNAi}* ($n = 15$, $P = 0.6675$). (**T–U′**) Knockdown of CSN5 in lymph gland progenitors using *dome^{MESO}-Gal4* driver by expressing *CSN5^{RNAi}* (**U–U′**) leads to expression of EGFR protein (green) marked by EGFR activity reporter (*EGFR-sfGFP*) in the periphery crystal cells marked by Hnt (magenta), in comparison to control (*dome^{MESO}-Gal4; EGFR-sfGFP)* (**T–T′**). (**V**) EGFR-sfGFP index in (Fig. 6J,K). *CSN5^{RNAi}* ($n = 28$, $P = 0.2401$) with control *w^{1118}* ($n = 28$). (**W**) Crystal cell index in (Fig. 6J,K). *CSN5^{RNAi}* ($n = 25$, $P < 0.0001$) with control *w^{1118}* ($n = 27$). (**X**) Pnt expression marked by pnt-GFP is observed throughout the lymph gland except Hnt+ crystal cells. (**Y**) Anti-Yan immunostaining shows Yan expression in the lz-GFP+ crystal cells. All confocal images represent maximum intensity projections of the middle third optical sections, except for image (**T–U′**, **X**, **Y**), where the images are single optical sections of the wandering third instar lymph gland lobe from at least three independent biological experiments. White dashed lines demarcate one lymph gland lobe. Scale bar, 25 µm. "*n*" represents the number of lymph gland lobes analyzed. Error bars in graphs: mean ± S.D. n.s.: not significant ($P \geq 0.05$); **$P < 0.01$; ***$P < 0.001$; ****$P < 0.0001$. A one-way ANOVA with Tukey's post-hoc test was used for analysis involving (**E**, **F**, **S**). A two-tailed, unpaired Student's *t* test was employed to compare the mean value of the two groups in (**I**, **L**, **V**, **W**). Source data are available online for this figure.

© The Author(s)
