# Supplementary material for: COP9 signalosome regulates EGFR and Notch signaling during myeloid-type progenitor cell fate decision in Drosophila
Source: EMBO Rep. 2026 Jul 7;27(16):4751–81. doi: 10.1038/s44319-026-00862-w (PMC13503907; doi:10.1038/s44319-026-00862-w)
Supplement: Supplementary file 2 [file 44319_2026_862_MOESM1_ESM.pdf]

Appendix for

"COP9 signalosome regulates EGFR and Notch signaling during myeloid-type progenitor cell fate decision in *Drosophila*"

Table of content

Appendix Figure S1	2
Appendix Figure S2	4
Appendix Figure S3	6
Appendix Figure S4	8
Appendix Figure S5	10
Appendix Figure S6	12
Appendix Figure S7	14
Appendix Table S1	16

Appendix Figure S1

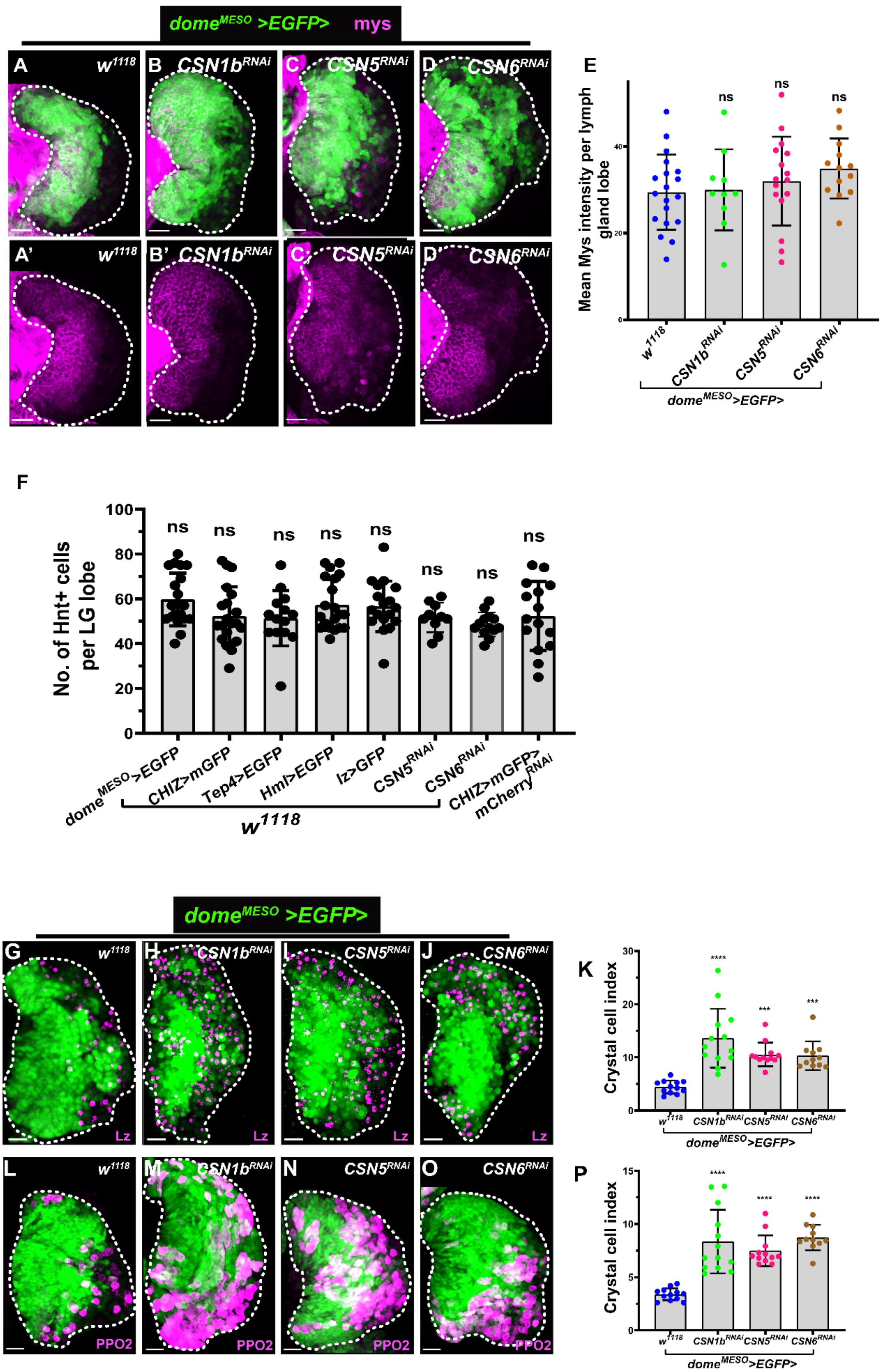

Appendix Figure S1. COP9 signalosome (CSN) regulates the lymph gland blood progenitors' cell fate

(**A-D'**) Knockdown of CSN in lymph gland progenitors (green) using *dome*^{MESO}*-Gal4, UAS-EGFP* driver by expressing *CSN1b*^{RNAi} (B-B') *CSN5*^{RNAi} (C-C') and *CSN6*^{RNAi} (D-D') does not induce lamellocyte (magenta) differentiation compared to control (A-A').

(**E**) Mean Mys (lamellocyte marker) intensity per anterior lymph gland lobe in (A-D'). *CSN1b*^{RNAi} (n=10, P=0.9987), *CSN5*^{RNAi} (n=16, P=0.8290), *CSN6*^{RNAi} (n=13, P=0.3335), and *w*^{1118} (n=19).

(**F)** The graph shows the number of crystal cells per anterior lymph gland lobe across genetic backgrounds, with each point indicating a lymph gland lobe. *dome*^{MESO}*>EGFP* (n=20), *CHIZ>mGFP* (n=20), *Tep4>EGFP* (n=14), *Hml>EGFP* (n=20), lz>GFP (n=18), *CSN5*^{RNAi} (n=10), *CSN6*^{RNAi} (n=13), *mCherry*^{RNAi} (n=15).

(**G-J**) Knockdown of CSN in progenitors (green) by expressing *CSN1b*^{RNAi} (H) *CSN5*^{RNAi} (I) and *CSN6*^{RNAi} (J) increases the early crystal cell marker Lz as compared to wild type (G).

(**K**) Crystal cell index in (G-J). *CSN1b*^{RNAi} (n=13, P<0.0001), *CSN5*^{RNAi} (n=12, P=0.0004), *CSN6*^{RNAi} (n=11, P=0.0009), and *w*^{1118} (n=13)

(**L-O**) Knockdown of CSN in progenitors (green) by expressing *CSN1b*^{RNAi} (M), *CSN5*^{RNAi} (N), and *CSN6*^{RNAi} (O) increases the mature crystal cell marker PPO2 (magenta) as compared to wild type (L).

(**P**) Crystal cell index in (L-O). *CSN1b*^{RNAi} (n=12, P<0.0001), *CSN5*^{RNAi} (n=12, P<0.0001), *CSN6*^{RNAi} (n=11, P<0.0001), and *w*^{1118} (n=12).

All confocal images represent maximum-intensity projections of the middle third optical sections, except images A-D' are the middle two optical sections of the wandering third instar anterior lymph gland lobe from at least three independent biological experiments. White dashed lines demarcate one lymph gland lobe. Scale bar, 25 µm. Error bars in graphs: mean ± S.D. n.s.: not significant (P≥0.05); ***P<0.001; ****P<0.0001. A one-way ANOVA with Tukey's post-hoc test was used for analysis involving E-F, K, and P.

Appendix Figure S2

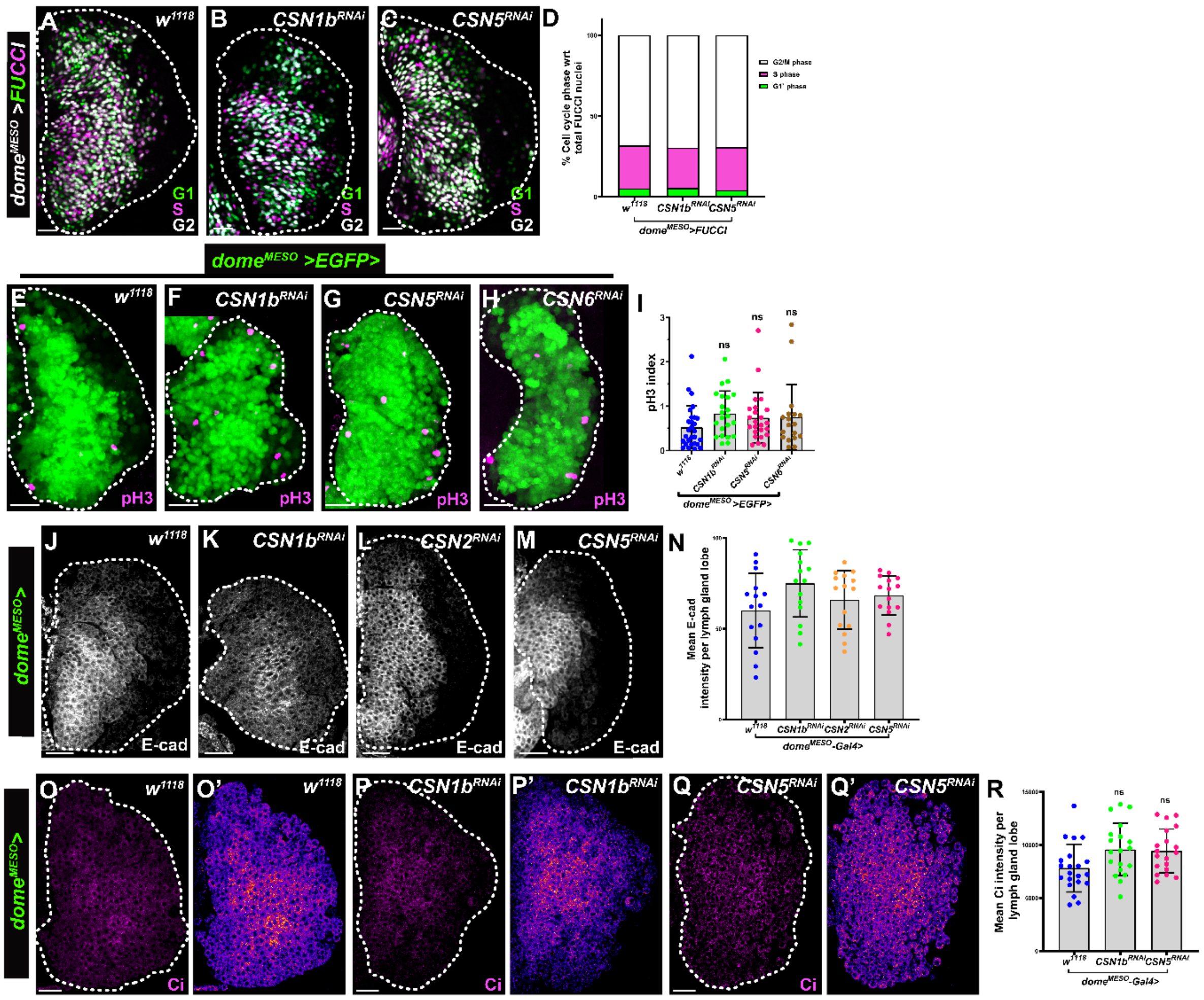

Appendix Figure S2. CSN depletion does not affect cell cycle or progenitor identity in the lymph gland

(A-C) Fluorescence Ubiquitin Cell Cycle Indicator (FUCCI) expressed transgenic fly line (PMID: 24726363) to check the G1-S-G2 cell cycle phases in the progenitors of the lymph gland (*dome^{MESO}>FUCCI*) (A). Progenitor-specific knockdown of *CSN1b* (B) and *CSN5* (C) does not alter the cell cycle phases compared to control (A). (S-phase: magenta, G1 phase: green, and G2/M phase: white).

(D) Quantifications of %G1, %S, and %G2/M cells in (A-C)**.** *CSN1b^{RNAi}* (n=11), *CSN5^{RNAi}* (n=13), and *w^{1118}* (n=21).

(E-H) Knockdown of CSN in lymph gland progenitors (green) using *dome^{MESO}-Gal4, UAS-EGFP* driver by expressing *CSN1b^{RNAi}* (F), *CSN5^{RNAi}* (G), and *CSN6^{RNAi}* (H) led to a similar number of mitotic marker pH3 positive cells (magenta) compared to the control (E).

(I) pH3 index in (E-H). *CSN1b^{RNAi}* (n=22, P=0.2382), *CSN5^{RNAi}* (n=24, P=0.5322), *CSN6^{RNAi}* (n=18, P=0.5540), and *w^{1118}* (n=28).

(J-M) Knockdown of CSN in lymph gland progenitors using *dome^{MESO}-Gal4* driver by expressing *CSN1b^{RNAi}* (K,) *CSN2^{RNAi}* (L), and *CSN5^{RNAi}* (M) resulted in similar E-cadherin (a known progenitor marker) expression (gray) compared to control (J). This result indicate that the lymph gland progenitor population is mostly unaffected in the CSN-depleted progenitor backgrounds.

(N) Mean E-cadherin immunostaining intensity per lymph gland lobe in (J-M). *CSN1b^{RNAi}* (n=15, P= 0.0847), *CSN2^{RNAi}* (n=15, P= 0.7769), *CSN5^{RNAi}* (n=15, P= 0.5357), and *w^{1118}* (n=15).

(O-Q') Knockdown of CSN in lymph gland progenitors using *dome^{MESO}-Gal4* driver by expressing *CSN1b^{RNAi}* (P-P') and *CSN5^{RNAi}* (Q-Q') resulted in similar Ci expression (magenta) as in control (O-O'). For better visibility, the heat map is also provided in O', P', and Q'.

(R) Mean Ci intensity per lymph gland lobe in (O-Q'). *CSN1b^{RNAi}* (n=17, P=0.0922), *CSN5^{RNAi}* (n=19, P=0.0599), and and *w^{1118}* (n=21).

All confocal images represent maximum intensity projections of the middle third optical sections, except images A-C**,** and J-Q' are the middle two optical sections of the wandering third instar lymph gland lobe from at least three independent biological experiments. White dashed lines demarcate one lymph gland lobe. Scale bar, 25 µm. Each data point represents the number of lymph gland lobes analyzed. Error bars in graphs: mean ± S.D. n.s.: not significant (P≥0.05). A one-way ANOVA with Tukey's post-hoc test was used for analysis involving **I, N,** and **R**.

Appendix Figure S3

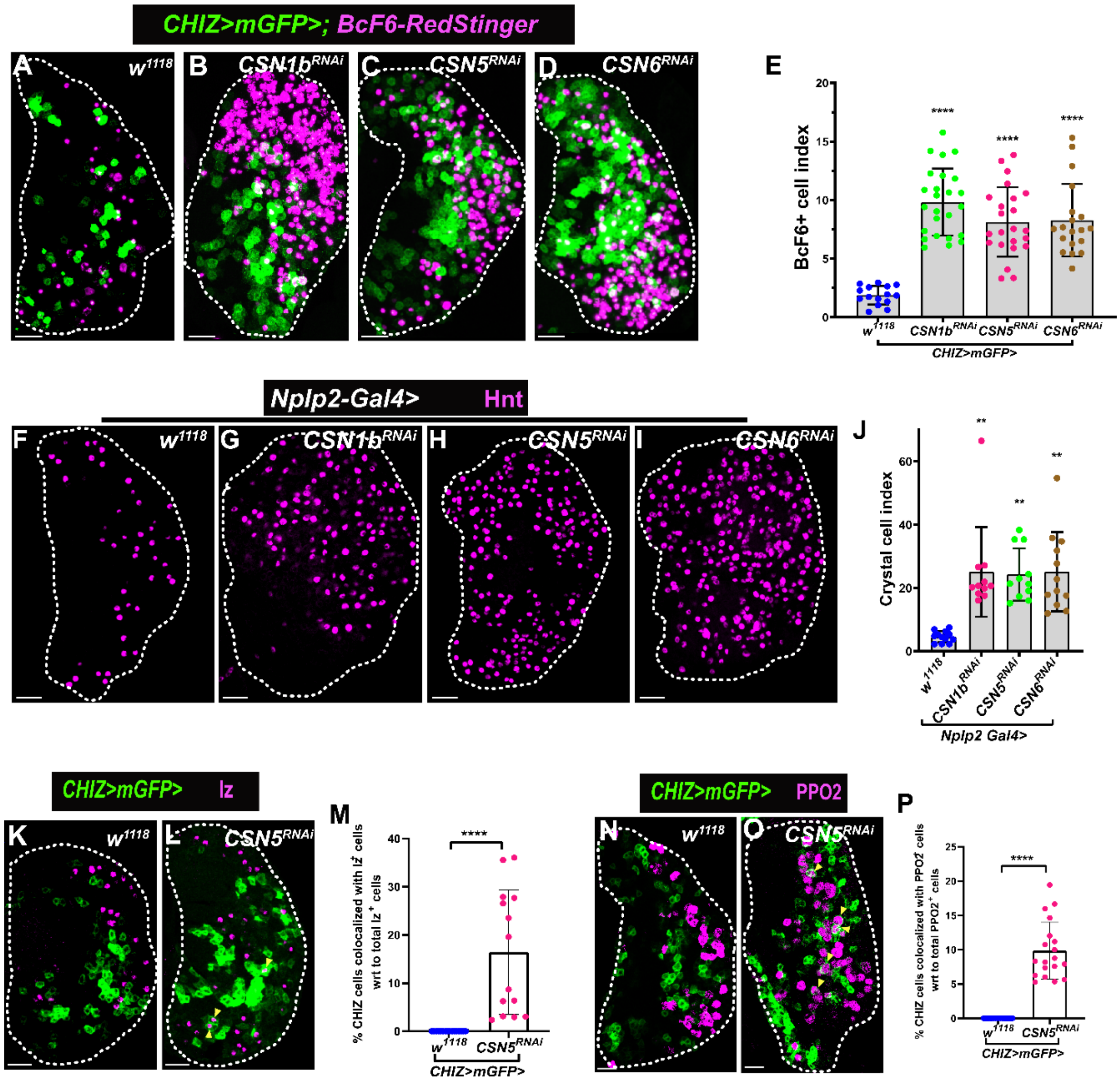

Appendix Figure S3. CSN is required during the myeloid-type intermediate progenitor cell fate decision

(A-D) Knockdown of CSN in Intermediate progenitors (green) by expressing *CSN1b*^{RNAi} (B), *CSN5*^{RNAi} (C), and *CSN6*^{RNAi} (D) increased the mature crystal cell marked *BcF6-RedStinger* (magenta) as compared to the control (A).

(E) *BcF6-RedStinger*+ cell index in (A-D). *w*^{1118} (n=15), *CSN1b*^{RNAi} (n=25. P<0.0001), *CSN5*^{RNAi} (n=23, P <0.0001) and *CSN6*^{RNAi} (n=19, P <0.0001).

(F-I) Knockdown of CSN in Intermediate progenitors using *Nplp2-Gal4* by expressing *CSN1b*^{RNAi} (G), *CSN5* ^{RNAi} (H), and *CSN6*^{RNAi} (I) increases the crystal cell number (magenta) as compared to the control (n=8) (F).

(J) Crystal cell index in (F-I). *CSN1b*^{RNAi} (n=11, P=0.0001), *CSN5* ^{RNAi} (n=11, P= 0.0002), *CSN6*^{RNAi} (n=12, P <0.0001), and *w*^{1118} (n=8).

(K-L) Intermediate progenitor-specific knockdown of *CSN5* (L) caused many lz+ cells to colocalize with CHIZ+ cells (green) compared to the control lymph gland (K).

(M) Quantification of the percentage of lz+ cells colocalizing with CHIZ+ cells with respect to total lz+ cells per lymph gland lobe in (K-L). $CSN5^{RNAi}$ (n=14, P <0.0001), and w^{1118} (n=16).

(N-O) Intermediate progenitor-specific knockdown of *CSN5* (O) caused many PPO2+ cells to colocalize with CHIZ+ cells (green) compared to the control lymph gland (N).

(P) Quantification of the percentage of PPO2+ cells colocalizing with CHIZ+ cells with respect to total PPO2+ cells per lymph gland lobe in (N-O). $CSN5^{RNAi}$ (n=19, P <0.0001), and w^{1118} (n=18).

All confocal images represent maximum-intensity projections of the middle-third optical sections, except images K-L and N-O, which are single optical sections of the wandering third-instar lymph gland lobe from at least three independent biological experiments. White dashed lines demarcate one lymph gland lobe. The yellow arrowhead indicates Lz+ and PPO2+ cells colocalizing with CHIZ+cells. Scale bar, 25 µm. Each data point represents the number of lymph gland lobes analyzed. Error bars in graphs: mean ± S.D. n.s.: not significant (P≥0.05); ***P<0.001; ****P<0.0001. A one-way ANOVA with Tukey's post-hoc test was used for analysis involving **E** and **J**. A two-tailed, unpaired Student's t-test was employed to compare the mean value of the two groups in **M** and **P.**

Appendix Figure S4

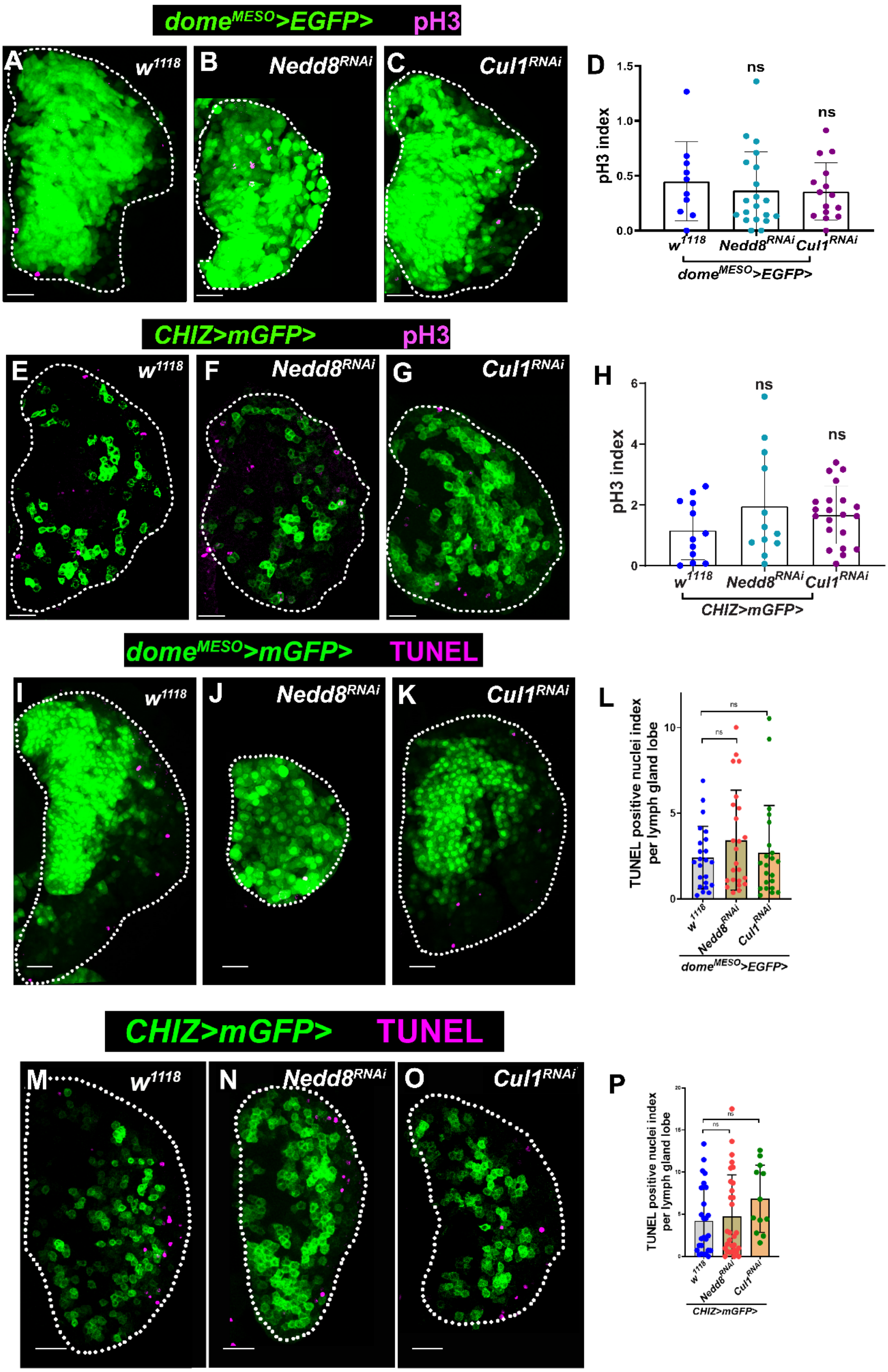

Appendix Figure S4. Nedd8 and Cullin1 depletion do not affect cell proliferation or apoptosis.

(A-C) Knockdown of *Nedd8* and *Cullin1* in lymph gland progenitors (green) using *dome*^{MESO}*-Gal4, UAS-EGFP* driver by expressing *Nedd8*^{RNAi} (B) and *Cul1*^{RNAi} (C) led to a similar number of mitotic marker pH3 positive cells (magenta) compared to the control (n=10) (A).

(D) pH3 index in (A-C). *Nedd8*^{RNAi} (n=20, P= 0.9876), *Cul1*^{RNAi} (n=15, P= 0.9847), and *w*^{1118} (n=10).

(E-G) Knockdown of *Nedd8* and *Cullin1* in lymph gland progenitors (green) using *CHIZ-Gal4, UAS-mGFP* driver by expressing *Nedd8*^{RNAi} (F) and *Cul1*^{RNAi} (G) led to a similar number of mitotic marker pH3 positive cells (magenta) compared to the control (E).

(H) pH3 index in (E-G). *Nedd8*^{RNAi} (n=12, P= 0.4059), *Cul1*^{RNAi} (n=21, P= 0.6592), and *w*^{1118} (n=12).

(I-K) Knockdown of *Nedd8* and *Cullin1* in lymph gland progenitors (green) using *dome*^{MESO}*-Gal4, UAS-EGFP* driver by expressing *Nedd8*^{RNAi} (J) and *Cul1*^{RNAi} (K) led to a similar number of TUNEL positive cells (magenta) compared to the control (I).

(L) TUNEL positive nuclei index in (I-K). *Nedd8*^{RNAi} (n=24, P=0.1605), *Cul1*^{RNAi} (n=23, P=0.6955), and *w*^{1118} (n=24).

(M-O) Knockdown of *Nedd8* and *Cullin1* in lymph gland progenitors (green) using *CHIZ-Gal4, UAS-mGFP* driver by expressing *Nedd8*^{RNAi} (N) and *Cul1*^{RNAi} (O) led to similar number of TUNEL positive cells (magenta) compared to the control (n=10) (M).

(P) TUNEL positive nuclei index in **(M-O).** *Nedd8*^{RNAi} (n=28, P=0.6552), *Cul1*^{RNAi} (n=12, P=0.07), and *w*^{1118} (n=10).

All confocal images represent maximum-intensity projections of the middle third optical sections of the wandering third instar anterior lymph gland lobe from at least three independent biological experiments. White dashed lines demarcate one lymph gland lobe. Scale bar, 25 µm. Error bars in graphs: mean ± S.D. n.s.: not significant (P≥0.05); ***P<0.001; ****P<0.0001. A one-way ANOVA with Tukey's post-hoc test was used for analysis involving **D, H, L** and **P**.

Appendix Figure S5

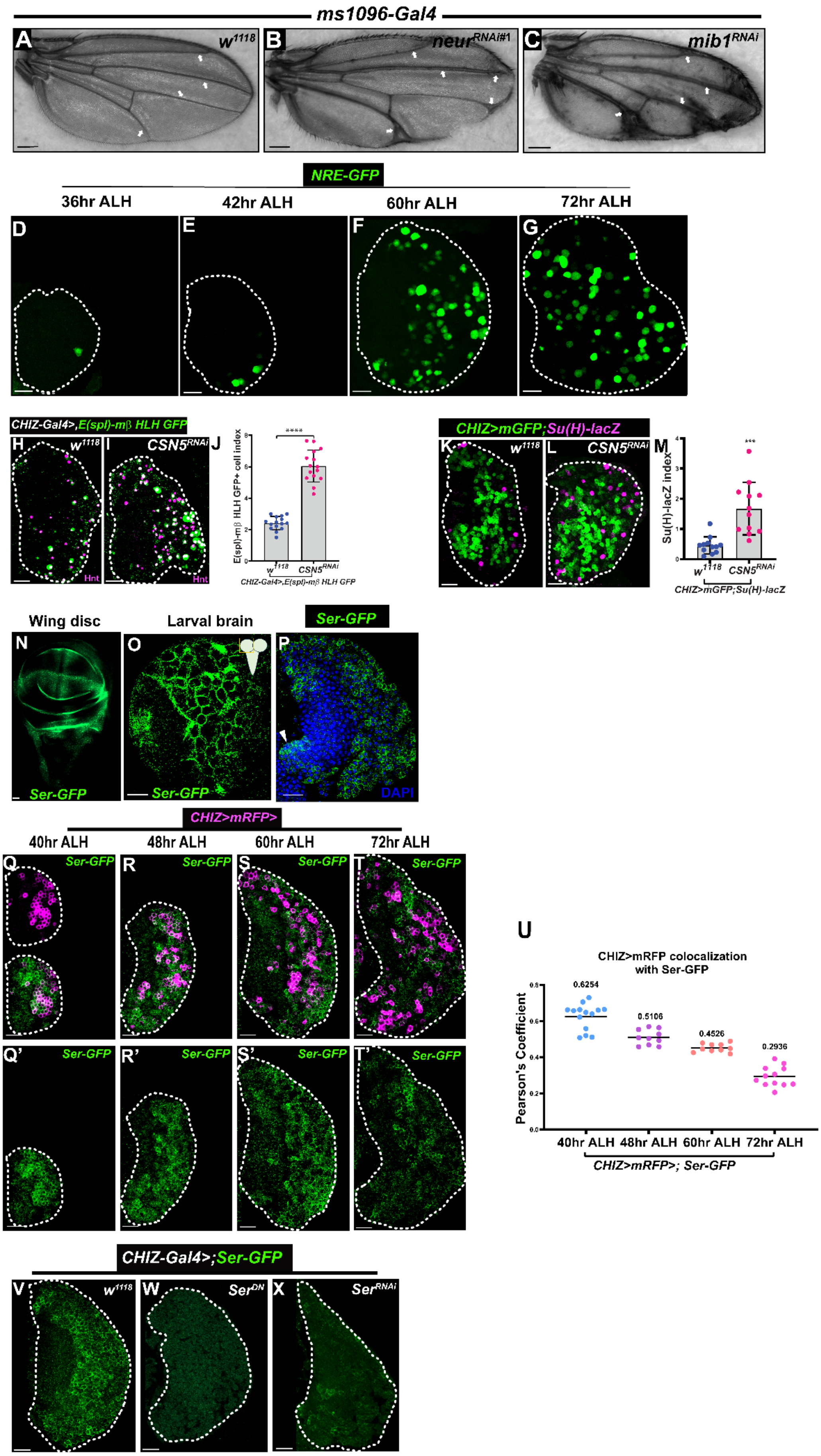

Appendix Figure S5. CSN modulates Neuralized/Serrate-mediated Notch activity in developing lymph gland cells

(A-C) Knockdown of *neuralized* and *mindbomb1* by expressing *neur*^{RNAi} (B) and *mib1*^{RNAi} (C) using the *ms1096-Gal4* driver in the wing disc resulted in a truncated wing vein marked by an arrow as compared with the control (*ms1096-Gal4/+*) (A).

(D-G) The NRE-GFP (green) starts expressing at mid-2nd instar (36hr ALH) and its number keeps increasing through the wandering third instar (72hr ALH) lymph gland.

(H, I, K, L) Knockdown of CSN5 in lymph gland intermediate progenitors using the *CHIZ Gal4* driver by expressing *CSN5RNAi* (I, L) resulted in increased expression of Notch activity reporters marked by *E(spl)-mβ HLH GFP* (H, I) and *Su(H)-lacZ* (K, L) as compared to control (H, K).

(J, M) Notch reporter activity index in (H, I, K, L). *E(spl)-mβ HLH GFP*: *w*^{1118} (n=15), *CSN5*^{RNAi} (n=29, P<0.0001) and *Su(H)-lacZ*: *w*^{1118} (n=13), *CSN5*^{RNAi} (n=13, P<0.0001).

(N, O) Ser-GFP fully recapitulates the endogenous expression in the larval wing disc (N) and optic lobe of the brain (O).

(P) Ser-GFP concentrates near the posterior signaling center of the lymph gland (arrowhead) and is limited to a shallow cortical layer, absent in the progenitor zone of the early 3rd instar lymph gland.

(Q-T') *CHIZ>mRFP* cells (magenta) colocalize with *Ser-GFP* (green) at mid-2nd and early 3rd instar (40hr ALH, 48hr ALH) lymph gland (Q-R'). Then *Ser-GFP* expression (green) begins to decrease and becomes nearly undetectable in the wandering 3rd instar lymph gland 60hr ALH and 72hr ALH, with significantly less or no colocalization between a few residual, low-serrate-expressing cells and the large number of CHIZ cells (S-T').

(U) Pearson's Coefficient of Ser-GFP co-localization with *CHIZ>mRFP* in (Q-T') at 40hr ALH (n=14), 48hr ALH (n=10), 60hr ALH (n=10) and 72hr ALH (n=12).

(V-X) Ser-GFP expression (green) is dramatically reduced in mid-3rd instar larval LG (60hALH) when *Ser*^{DN} *and Ser*^{RNAi} is driven by *CHIZ-Gal4* (W-X) when compared to control LG *(CHIZ-Gal4>; Ser-GFP)* (V)**.**

All confocal images represent maximum intensity projections of the middle third optical sections of the primary lobe of the lymph gland has been outlined in white dashed lines and represented with maximum intensity projections of the middle third optical sections, except images A-C of wings, N is wing disc, O is brain lobe, and Q- T' images of lymph glands are single optical section. All confocal images represent the wandering third instar except D-G and Q-T'. "n" represents the number of lymph gland lobes analyzed. Scale bar, 25 µm except image A-C scale bar, 500 µm. ***P<0.001; ****P<0.0001. Bars in graphs: mean with S.D. A two-tailed, unpaired Student's t-test was employed to compare the mean value of the two groups in **J** and **M.**

Appendix Figure S6

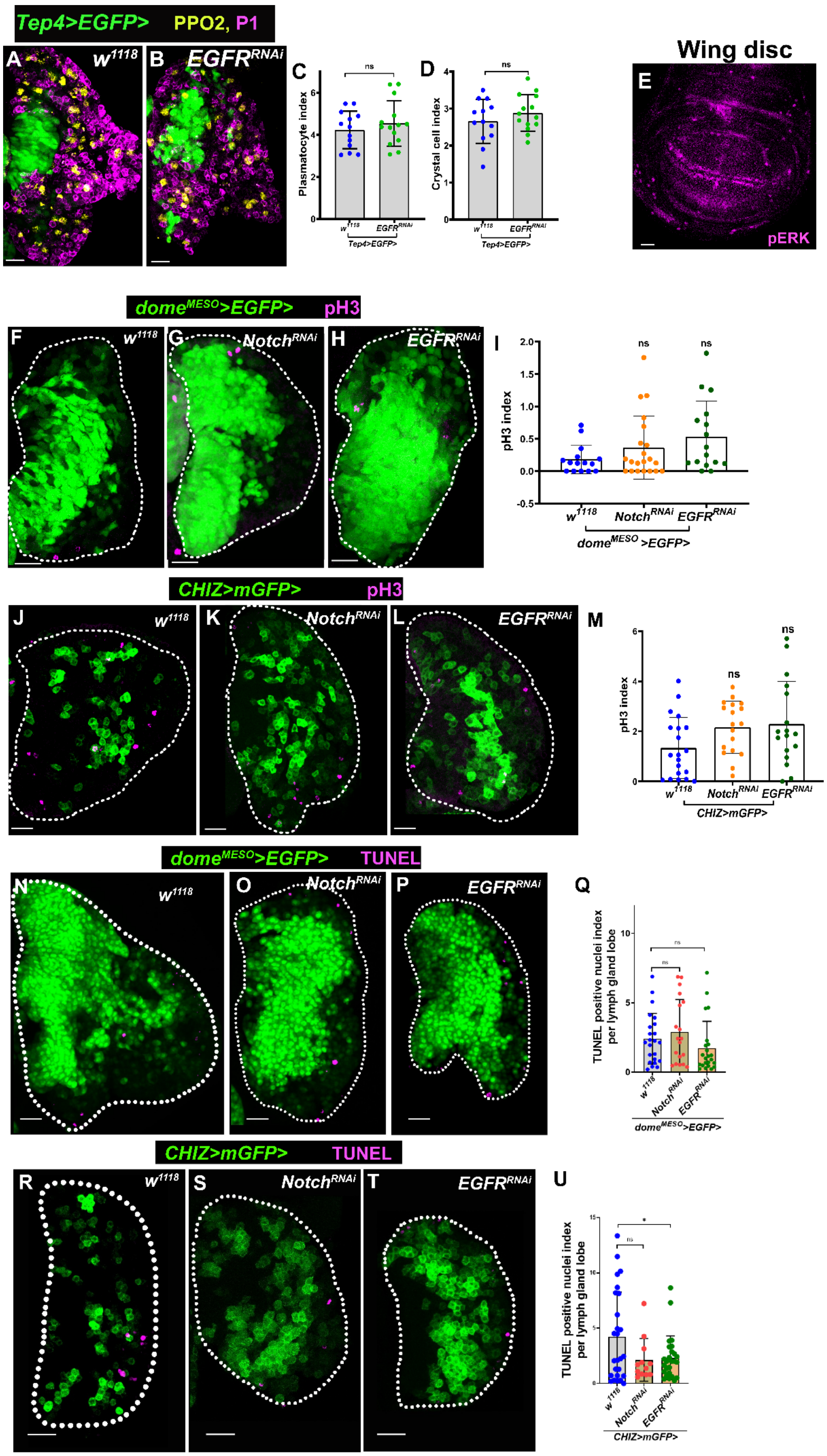

Appendix Figure S6. CSN is genetically associated with EGFR signaling during lymph gland cell fate decision

(A-B) Knockdown of EGFR signaling pathway in lymph gland core progenitors (green) using *Tep4-Gal4, UAS-EGFP* driver by expressing *EGFR*$^{RNAi\#1}$ (B) shows no significant change in crystal cell (yellow) and plasmatocytes (magenta) number as compared to wild type lymph gland (*Tep4-Gal4, UAS-EGFP /+*)(A).

(C) Plasmatocyte index in (A-B); *EGFR*$^{RNAi\#1}$ (n=13, P=0.4204), and *w*^{1118} (n=13).

(D) Crystal cell index in (A-B); *EGFR*$^{RNAi\#1}$ (n=13, P=0.2848), and *w*^{1118} (n=13).

(E) pERK fully recapitulates the endogenous expression in the wing disc.

(F-H) Knockdown of *Notch* and *EGFR* in lymph gland progenitors (green) using *dome*^{MESO}*-Gal4, UAS-EGFP* driver by expressing *Notch*^{RNAi} (G), and *EGFR*^{RNAi} (H) led to similar number of mitotic marker pH3 positive cells (magenta) compared to the control(F).

(I) pH3 index in (F-H). *Notch*^{RNAi} (n=21, P= 0.9847), *EGFR*^{RNAi} (n=16, P= 0.8486), and *w*^{1118} (n=15).

(J-L) Knockdown of *Notch* and *EGFR* in lymph gland progenitors (green) using *CHIZ-Gal4, UAS-mGFP* driver by expressing *Notch*^{RNAi} (K) and *EGFR*^{RNAi} (L) led to a similar number of mitotic marker pH3 positive cells (magenta) compared to the control (J).

(M) pH3 index in (J-L). *Notch*^{RNAi} (n=17, P= 0.1141), *EGFR*^{RNAi} (n=17, P= 0.0636), and *w*^{1118} (n=20).

(N-P) Knockdown of *Notch* and *EGFR* in lymph gland progenitors (green) using *dome*^{MESO}*-Gal4, UAS-EGFP* driver by expressing *Notch*^{RNAi} (O) and *EGFR*^{RNAi} (P) led to a similar number of TUNEL positive cells (magenta) compared to the control (N).

(Q) TUNEL positive nuclei index in (N-P). *Notch*^{RNAi} (n=21, P=0.4425), *EGFR*^{RNAi} (n=24, P=0.2310), and *w*^{1118} (n=24).

(R-T) Knockdown of *Notch* and *EGFR* in lymph gland progenitors (green) using *CHIZ-Gal4, UAS-mGFP* driver by expressing *Notch*^{RNAi} (S) and *EGFR*^{RNAi} (T) led to a similar number of TUNEL positive cells (magenta) compared to the control (R).

(U) TUNEL positive nuclei index in (R-T). *Notch*^{RNAi} (n=13, P=0.1006), *EGFR*^{RNAi} (n=25, P=0.0376), and *w*^{1118} (n=24).

All confocal images represent maximum intensity projections of the middle third optical sections, where the images are single optical sections of the wandering third instar lymph gland lobe, except image E, which is the single optical section of the wing disc from at least three independent biological experiments. White dashed lines demarcate one lymph gland lobe. Scale bar, 25 µm. “n” represents the number of lymph gland lobes analyzed. Error bars in graphs: mean ± S.D. n.s.: not significant (P≥0.05); *P<0.05; **P<0.01; ***P<0.001; ****P<0.0001. A one-way ANOVA with Tukey’s post-hoc test was used for analysis involving **I, M, Q,** and **U**. A two-tailed, unpaired Student’s t-test was employed to compare the mean value of the two groups in **C** and **D.**

Appendix Figure S7

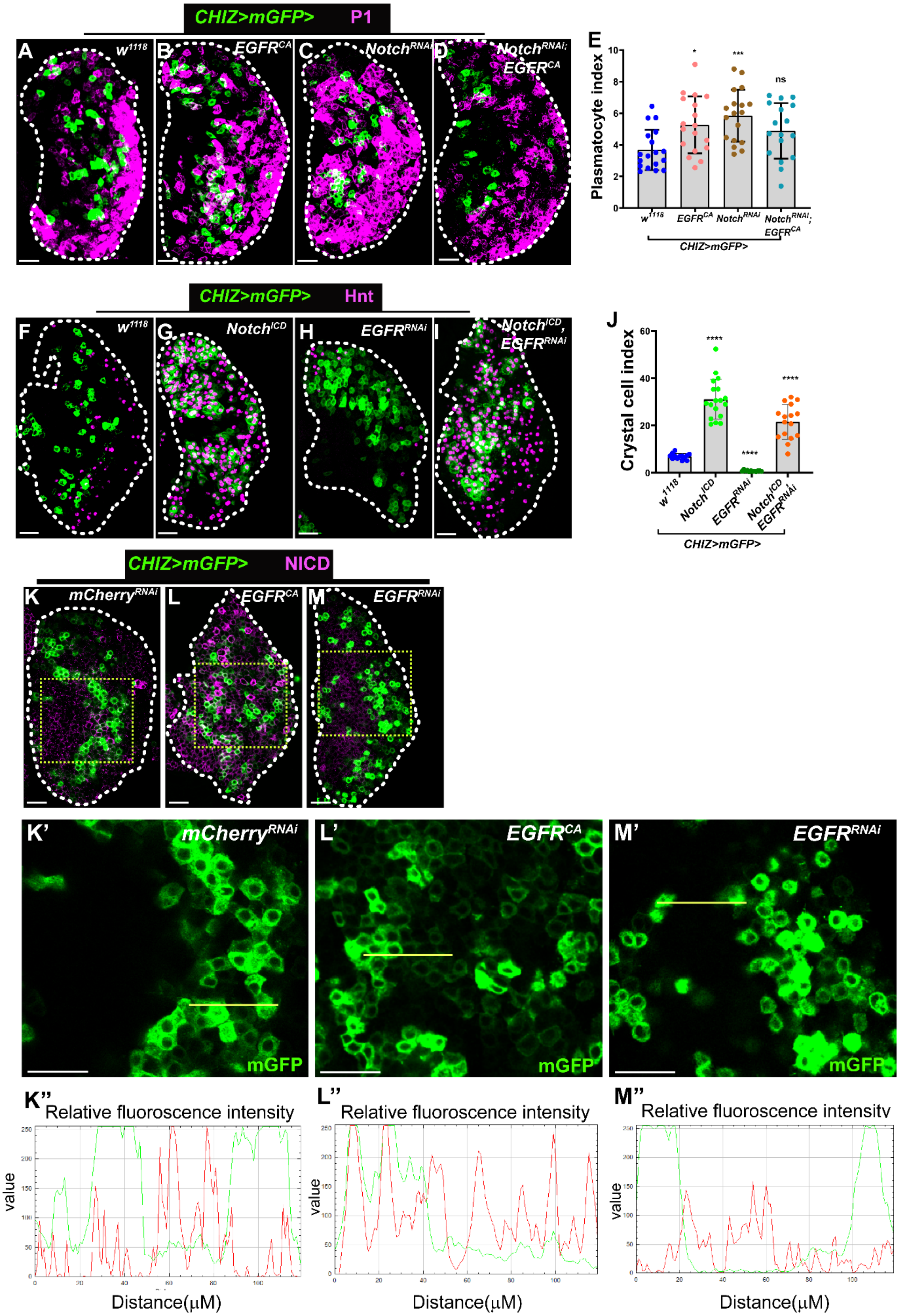

Appendix Figure S7. The combination of CSN, EGFR, and Notch signaling regulates crystal cell specification in the lymph gland

(**A-D**) A high number of plasmatocytes in constitutive active EGFR overexpression *EGFR*^{CA} (B), when compared to control (A), in intermediate progenitors are rescued (D) by depleting Notch using *Notch*^{RNAi} (C).

(E) Plasmatocyte index in (A-D). *EGFR*^{CA} (n=18, P=0.0258), *Notch*^{RNAi} (n=18, P=0.0010), *Notch*^{RNAi}*;* *EGFR*^{CA} (n=17, P=0.1418), and *w*^{1118} (n=18).

(**F-I**) A high number of crystal cells in *Notch*^{ICD} (G) when compared to control (F) in intermediate progenitors isn't rescued (I) by depleting EGFR using *EGFR*^{RNAi} (H).

(J) Crystal cell index in (F-I). *Notch*^{ICD} (n=18, P<0.0001), *EGFR*^{RNAi} (n=12, P<0.0001), *Notch*^{ICD}*,* *EGFR*^{RNAi}(n=16, P<0.0001), and *w*^{1118} (n=15).

(K-M') Alteration of EGFR signaling in lymph gland intermediate progenitors (green) using *CHIZ-Gal4, UAS-mGFP* driver by expressing *EGFR*^{CA} (L-L') increases the NICD expression, and *EGFR*^{RNAi} (M-M') reduces the NICD expression in comparison to the control (*CHIZ-Gal4, UAS-mGFP; mCherry*^{RNAi}) (K-K'). The insets in the yellow dotted box indicate NICD expression in *EGFR*^{CA} (L'), *EGFR*^{RNAi} (M'), and control *mCherry*^{RNAi}(K'). Fig. 6P-R' shows insets of the lymph gland (K-M) with NICD and CHIZ+ cells.

(K''-M'') Relative fluorescent intensity of NICD (red) and CHIZ+ cells (green) at the yellow solid line in K-K', L-L', and M-M', respectively.

All confocal images represent maximum intensity projections of the middle third optical sections, except images I-K", the single optical section of the wandering third instar lymph gland lobe from at least three independent biological experiments. White dashed lines demarcate one lymph gland lobe. Scale bar, 25 µm. Each data point represents the number of lymph gland lobes analyzed (n). Error bars in graphs: mean ± S.D. n.s.: not significant (P≥0.05); *P<0.05; ***P<0.001; ****P<0.0001. A two-tailed, unpaired Student's t-test was employed to compare the mean value of the two groups in **E** and **J.**

Appendix Table S1: Main figures' genetic background, immunostaining used and sample sizes.

Figure no.	Genotype	Marker	n
Fig. 1D	$dome^{MESO}$-Gal4 UAS-EGFP	Hnt	21
Fig. 1E	$dome^{MESO}$-Gal4 UAS-EGFP, UAS-$CSN1b^{RNAi}$	Hnt	20
Fig. 1F	$dome^{MESO}$-Gal4 UAS-EGFP, UAS-$CSN5^{RNAi}$	Hnt	19
Fig. 1G	$dome^{MESO}$-Gal4 UAS-EGFP; UAS-CSN5	Hnt	17
Fig. 1H	$dome^{MESO}$-Gal4 UAS-EGFP UAS-CSN5, UAS-$CSN5^{RNAi}$	Hnt	14
Fig. 1J	$dome^{MESO}$-Gal4 UAS-EGFP	P1	32
Fig. 1K	$dome^{MESO}$-Gal4 UAS-EGFP, UAS-$CSN1b^{RNAi}$	P1	15
Fig. 1L	$dome^{MESO}$-Gal4 UAS-EGFP, UAS-$CSN5^{RNAi}$	P1	19
Fig. 1M	$dome^{MESO}$-Gal4 UAS-EGFP; UAS-CSN5	P1	18
Fig. 1N	$dome^{MESO}$-Gal4 UAS-EGFP UAS-CSN5, UAS-$CSN5^{RNAi}$	P1	10
Fig. 1P	$dome^{MESO}$-Gal4 UAS-EGFP at 36hr ALH	Hnt	14
Fig. 1U	$dome^{MESO}$-Gal4 UAS-EGFP, UAS-$CSN1b^{RNAi}$ at 36hr ALH	Hnt	12
Fig. 1Q	$dome^{MESO}$-Gal4 UAS-EGFP at 42hr ALH	Hnt	10
Fig. 1V	$dome^{MESO}$-Gal4 UAS-EGFP, UAS-$CSN1b^{RNAi}$ at 42hr ALH	Hnt	14
Fig. 1R	$dome^{MESO}$-Gal4 UAS-EGFP at 48hr ALH	Hnt	18
Fig. 1W	$dome^{MESO}$-Gal4 UAS-EGFP, UAS-$CSN1b^{RNAi}$ at 48hr ALH	Hnt	15
Fig. 1S	$dome^{MESO}$-Gal4 UAS-EGFP at 60hr ALH	Hnt	15
Fig. 1X	$dome^{MESO}$-Gal4 UAS-EGFP, UAS-$CSN1b^{RNAi}$ at 60hr ALH	Hnt	15
Fig. 1T	$dome^{MESO}$-Gal4 UAS-EGFP at 72hr ALH	Hnt	13
Fig. 1Y	$dome^{MESO}$-Gal4 UAS-EGFP, UAS-$CSN1b^{RNAi}$ at 72hr ALH	Hnt	16
Fig. 1ZA	Tep4-Gal4 UAS-EGFP	Hnt	12
Fig. 1ZB	Tep4-Gal4 UAS-EGFP; UAS-$CSN1b^{RNAi}$	Hnt	16
Fig. 1ZC	Tep4-Gal4 UAS-EGFP; UAS-$CSN5^{RNAi}$	Hnt	14
Fig. 1ZE	Hml^{Δ}-Gal4 UAS-EGFP	Hnt	23
Fig. 1ZF	Hml^{Δ} -Gal4 UAS-EGFP; UAS-$CSN1b^{RNAi}$	Hnt	23
Fig. 1ZG	Hml^{Δ} -Gal4 UAS-EGFP; UAS-$CSN5^{RNAi}$	Hnt	15

Fig. 2A	CHIZ-Gal4, UAS-mGFP	Hnt	20
Fig. 2B	CHIZ-Gal4 UAS-mGFP; UAS-$CSN1b^{RNAi}$	Hnt	25
Fig. 2C	CHIZ-Gal4 UAS-mGFP; UAS-$CSN5^{RNAi}$	Hnt	20
Fig. 2D	CHIZ-Gal4 UAS-mGFP; $CSN5^{RNAi}$	Hnt	20
Fig. 2E	CHIZ-Gal4 UAS-mGFP, UAS-CSN5	Hnt	15
Fig. 2G	CHIZ-Gal4, UAS-mGFP	P1	32
Fig. 2H	CHIZ-Gal4 UAS-mGFP; UAS-$CSN1b^{RNAi}$	P1	16
Fig. 2I	CHIZ-Gal4 UAS-mGFP; UAS-$CSN5^{RNAi}$	P1	15
Fig. 2J	CHIZ-Gal4 UAS-mGFP, UAS-CSN5	P1	18
Fig. 2K	CHIZ-Gal4 UAS-mGFP, UAS-CSN5; UAS $CSN5^{RNAi}$	P1	14
Fig. 3M-M'	CHIZ-Gal4 UAS-mGFP	Hnt	30
Fig. 3N-N'	CHIZ-Gal4 UAS-mGFP; UAS-$CSN1b^{RNAi}$	Hnt	29

Fig. 3A	$dome^{MESO}$-Gal4 UAS-EGFP	Hnt	20
Fig. 3B	$dome^{MESO}$-Gal4 UAS-EGFP, UAS-$Nedd8^{RNAi}$	Hnt	16
Fig. 3C	$dome^{MESO}$-Gal4 UAS-EGFP, UAS-$Cul1^{RNAi}$	Hnt	22
Fig. 3E	CHIZ-Gal4 UAS-mGFP	Hnt	20
Fig. 3F	CHIZ-Gal4 UAS-mGFP; UAS-$Nedd8^{RNAi}$	Hnt	12

Fig. 3G	*CHIZ-Gal4 UAS-mGFP; UAS-Cul1* ^{RNAi}	Hnt	22
Fig. 3I	*CHIZ-Gal4 UAS-mGFP*	Hnt	13
Fig. 3J	*CHIZ-Gal4 UAS-mGFP; UAS-Cul1* ^{RNAi}	Hnt	15
Fig. 3K	*CHIZ-Gal4 UAS-mGFP; CSN5* ^{RNAi}	Hnt	14
Fig. 3L	*CHIZ-Gal4 UAS-mGFP; UAS-Cul1* ^{RNAi} *; UAS-CSN5* ^{RNAi}	Hnt	18

Fig. 4B	*CHIZ-Gal4 UAS-mGFP*	Hnt	18, 13 and 15
Fig. 4C	*CHIZ-Gal4 UAS-mGFP, UAS-Notch* ^{RNAi}	Hnt	21
Fig. 4D	*CHIZ-Gal4 UAS-mGFP, UAS-Serrate* ^{RNAi}	Hnt	25
Fig. 4E	*CHIZ-Gal4 UAS-mGFP; UAS-neur* ^{RNAi}	Hnt	29
Fig. 4F	*CHIZ-Gal4 UAS-mGFP; UAS-CSN5* ^{RNAi}	Hnt	29, 12 and 15
Fig. 4G	*CHIZ-Gal4 UAS-mGFP, UAS-Notch* ^{RNAi} *; UAS-CSN5* ^{RNAi}	Hnt	20
Fig. 4H	*CHIZ-Gal4 UAS-mGFP, UAS-Ser* ^{RNAi} *; UAS-CSN5* ^{RNAi}	Hnt	10
Fig. 4I	*CHIZ-Gal4 UAS-mGFP, UAS-neur* ^{RNAi} *; UAS-CSN5* ^{RNAi}	Hnt	14
Fig. 4L	*CHIZ-Gal4 NRE-GFP*	Hnt	15
Fig. 4M	*CHIZ-Gal4 NRE-GFP; UAS-CSN5* ^{RNAi}	Hnt	15
Fig. 4O-O''	*HHLT-Gal4 UAS-2EGFP*	NICD	18
Fig. 4P-P''	*HHLT-Gal4 UAS-2EGFP; UAS-CSN5* ^{RNAi}	NICD	21
Fig. 4Q-Q''	*HHLT-Gal4 UAS-2EGFP, UAS-Notch* ^{RNAi}	NICD	23
Fig. 4R-R''	*HHLT-Gal4 UAS-2EGFP, UAS-Notch* ^{RNAi} *; UAS-CSN5* ^{RNAi}	NICD	17
Fig. 4S-S''	*HHLT-Gal4 UAS-2EGFP, UAS-Serrate* ^{RNAi}	NICD	16
Fig. 4T-T''	*HHLT-Gal4 UAS-2EGFP, UAS-Serrate* ^{RNAi} *; UAS-CSN5* ^{RNAi}	NICD	23
Fig. 4U-U'	*CHIZ-Gal4; Ser-EGFP*	GFP, Hnt	13
Fig. 4V-V'	*CHIZ-Gal4; UAS-CSN5* ^{RNAi} *, Ser-EGFP*	GFP, Hnt	13

Fig. 5A	*CHIZ-Gal4 UAS-mGFP*	Hnt	35, 14 and 16
Fig. 5B	*CHIZ-Gal4 UAS-mGFP, UAS-EGFR* ^{RNAi}	Hnt	25
Fig. 5C	*CHIZ-Gal4 UAS-mGFP, UAS-rolled* ^{RNAi}	Hnt	18
Fig. 5D	*CHIZ-Gal4 UAS-mGFP, UAS-EGFR* ^{DN}	Hnt	18
Fig. 5E	*CHIZ-Gal4 UAS-mGFP, UAS-EGFR* ^{CA}	Hnt	16
Fig. 5F	*CHIZ-Gal4 UAS-mGFP, UAS-cic* ^{RNAi}	Hnt	16
Fig. 5G	*CHIZ-Gal4 UAS-mGFP, UAS-pnt* ^{RNAi}	Hnt	14
Fig. 5H	*CHIZ-Gal4 UAS-mGFP, UAS-pnt* ^{OE}	Hnt	12
Fig. 5I	*CHIZ-Gal4 UAS-mGFP, UAS-Yan* ^{RNAi}	Hnt	10
Fig. 5J	*CHIZ-Gal4 UAS-mGFP, UAS-Yan* ^{Act}	Hnt	15
Fig. 5K	*CHIZ-Gal4 UAS-mGFP, UAS-CSN5* ^{RNAi}	Hnt	35
Fig. 5L	*CHIZ-Gal4 UAS-mGFP, UAS-EGFR* ^{RNAi} *UAS-CSN5* ^{RNAi}	Hnt	35
Fig. 5O-O'	*CHIZ-Gal4 UAS-mGFP, EGFR::sfGFP*	Hnt, GFP	17
Fig. 5P-P'	*CHIZ-Gal4 UAS-mGFP, EGFR::sfGFP; UAS-CSN5* ^{RNAi}	Hnt, GFP	19
Fig. 5Q-Q''	*CHIZ-Gal4 UAS-mGFP*	EGFR	20
Fig. 5R-R''	*CHIZ-Gal4 UAS-mGFP, UAS-CSN7* ^{RNAi}	EGFR	15
Fig. 5S	*CHIZ-Gal4 UAS-mGFP*	pERK	12
Fig. 5T	*CHIZ-Gal4 UAS-mGFP; UAS-CSN1b* ^{RNAi}	pERK	14
Fig. 5U	*CHIZ-Gal4 UAS-mGFP; UAS-CSN5* ^{RNAi}	pERK	14
Fig. 5V	*CHIZ-Gal4 UAS-mGFP; UAS-CSN6* ^{RNAi}	pERK	8
Fig. 5X-X'	*cic-GFP*	Hnt	13

Fig. 5Y-Y'	*CHIZ-Gal4 pnt-GFP*	Yan	11
Fig. 5Z-Z'	*CHIZ-Gal4 pnt-GFP; UAS-CSN5* ^{RNAi}	Yan	11

Fig. 6A	*CHIZ-Gal4 UAS-mGFP*	Hnt	13, 11
Fig. 6B	*CHIZ-Gal4 UAS-mGFP, UAS-Notch* ^{RNAi}	Hnt	15
Fig. 6C	*CHIZ-Gal4 UAS-mGFP, UAS-Serrate* ^{DN}	Hnt	13
Fig. 6D	*CHIZ-Gal4 UAS-mGFP, UAS-EGFR* ^{CA}	Hnt	12, 13
Fig. 6E	*CHIZ-Gal4 UAS-mGFP, UAS-Notch* ^{RNAi} *; UAS-EGFR* ^{CA}	Hnt	16
Fig. 6F	*CHIZ-Gal4 UAS-mGFP, UAS-Serrate* ^{DN} *; UAS-EGFR* ^{CA}	Hnt	20
Fig. 6I	*CHIZ-Gal4; Ser-EGFP*	Hnt, GFP	11
Fig. 6J	*CHIZ-Gal4; UAS-EGFR* ^{RNAi} *, Ser-EGFP*	Hnt, GFP	11
Fig. 6K	*CHIZ-Gal4; UAS-EGFR* ^{CA} *, Ser-EGFP*	Hnt, GFP	11
Fig. 6M	*CHIZ-Gal4 E(spl)-m* β *-HLH*	Hnt	10
Fig. 6N	*CHIZ-Gal4 E(spl)-mb-HLH; UAS-EGFR* ^{CA}	Hnt	10
Fig. 6P-P'	*CHIZ-Gal4 UAS-mGFP, UAS-mCherry* ^{RNAi}	NICD	12
Fig. 6Q-Q'	*CHIZ-Gal4 UAS-mGFP, UAS-EGFR* ^{CA}	NICD	11
Fig. 6R-R'	*CHIZ-Gal4 UAS-mGFP, UAS-EGFR* ^{RNAi}	NICD	16
