# Supplementary material for: COP9 signalosome regulates EGFR and Notch signaling during myeloid-type progenitor cell fate decision in Drosophila
Source: EMBO Rep. 2026 Jul 7;27(16):4751–81. doi: 10.1038/s44319-026-00862-w (PMC13503907; doi:10.1038/s44319-026-00862-w)
Supplement: Supplementary file 3 [file 44319_2026_862_MOESM2_ESM.pdf]

EMBO reports

COP9 signalosome regulates EGFR and Notch signaling during myeloid-type progenitor cell fate decision in Drosophila

Gayatri Rai, Deepak Maurya, Debleena Mandal, and Bama Charan Mondal

Corresponding author(s): Bama Charan Mondal (bamacharan@bhu.ac.in)

Review Timeline:

Submission Date:	22nd Oct 25
Editorial Decision:	22nd Dec 25
Revision Received:	28th Mar 26
Editorial Decision:	27th May 26
Revision Received:	13th Jun 26
Accepted:	24th Jun 26

Editor: Martina Rembold

Transaction Report:

(Note: With the exception of the correction of typographical or spelling errors that could be a source of ambiguity, letters and reports are not edited. Depending on transfer agreements, referee reports obtained elsewhere may or may not be included in this compilation. Referee reports are anonymous unless the Referee chooses to sign their reports.)

1st Editorial Decision 22nd Dec 2025

Dear Dr. Mondal

Thank you for the submission of your manuscript to EMBO reports. I apologize for the delay in handling it, but we have now received the full set of referee reports as well as referee cross-comments that are all pasted below.

As you will see, the referees acknowledge that the findings are potentially interesting and consider the data overall solid. The referees however also point out that the CSN has been shown to regulate Notch and EGFR before, which in turn are known players in the lymph gland. I have discussed these novelty concerns further with the referees and both consider your study of interest to Notch biologists given that the connection between CSN and Notch has not been extensively studied (Referee #1) and for theidentification of the Drosophila lymph gland as a valid biological system to unravel the connections between the CSN complex ant the signaling pathways that regulate fate choices.

That said, it will be crucial to focus the study, streamline it and work out the most relevant points along the lines suggested by the referees.

I would thus like to invite you to revise your manuscript with the understanding that the referee concerns must be fully addressed and their suggestions taken on board. Please address all referee concerns in a complete point-by-point response. Acceptance of the manuscript will depend on a positive outcome of a second round of review. It is EMBO reports policy to allow a single round of major revision only and acceptance or rejection of the manuscript will therefore depend on the completeness of your responses included in the next, final version of the manuscript.

We realize that it is difficult to revise to a specific deadline. In the interest of protecting the conceptual advance provided by the work, we recommend a revision within 3 months (23rd Mar 2026). Please discuss the revision progress ahead of this time with the editor if you require more time to complete the revisions.

You can either publish the study as a short report or as a full article. For short reports, the revised manuscript should not exceed 27,000 characters (including spaces but excluding materials & methods and references) and 5 main plus 5 expanded view figures. The results and discussion sections must further be combined, which will help to shorten the manuscript text by eliminating some redundancy that is inevitable when discussing the same experiments twice. For a normal article there are no length limitations, but it should have more than 5 main figures and the results and discussion sections must be separate. In both cases, the entire materials and methods must be included in the main manuscript file.

IMPORTANT NOTE: we perform an initial quality control of all revised manuscripts before re-review. Your manuscript will FAIL this control and the handling will be DELAYED if the following APPLIES:
1) A data availability section providing access to data deposited in public databases is missing. If you have not deposited any data, please add a sentence to the data availability section that explains that.
2) Your manuscript contains statistics and error bars based on n=2. Please use scatter blots in these cases. No statistics should be calculated if n=2.

When submitting your revised manuscript, please review the submission guidelines (https://link.springer.com/journal/44319/submission-guidelines#cms-Revised-submissions) and carefully review the instructions that follow below. Failure to include requested items will delay the evaluation of your revision.

1) a .docx formatted version of the manuscript text (including legends for main figures, EV figures and tables). Please make sure that the changes are highlighted to be clearly visible.

2) individual production quality figure files as .eps, .tif, .jpg (one file per figure). See hhttps://media.springernature.com/original/springer-cms/rest/v1/content/27825798/data/v1 for more info on how to prepare your figures.

3) We replaced Supplementary Information with Expanded View (EV) Figures and Tables that are collapsible/expandable online. A maximum of 5 EV Figures can be typeset. EV Figures should be cited as 'Figure EV1, Figure EV2" etc... in the text and their respective legends should be included in the main text after the legends of regular figures.

- For the figures that you do NOT wish to display as Expanded View figures, they should be bundled together with their legends in a single PDF file called *Appendix*, which should start with a short Table of Content. Appendix figures should be referred to in the main text as: "Appendix Figure S1, Appendix Figure S2" etc. See 'Expanded View' section of the submission guidelines.

- Additional Tables/Datasets should be labeled and referred to as Table EV1, Dataset EV1, etc. Legends have to be provided in a separate tab in case of .xls files. Alternatively, the legend can be supplied as a separate text file (README) and zipped together with the Table/Dataset file.

4) a .docx formatted letter INCLUDING the reviewers' reports and your detailed point-by-point responses to their comments. As part of the EMBO Press transparent editorial process, the point-by-point response is part of the Review Process File (RPF), which will be published alongside your paper.

5) a complete author checklist, which you can download from our author guidelines. Please insert information in the checklist that is also reflected in the manuscript. The completed author checklist will also be part of the RPF.

6) Please note that all corresponding authors are required to supply an ORCID ID for their name upon submission of a revised manuscript (<https://orcid.org/>), which can be linked to your profile in the manuscript submission system.

7) Before submitting your revision, primary datasets produced in this study need to be deposited in an appropriate public database. Please remember to provide a reviewer password if the datasets are not yet public. The accession numbers and database should be listed in a formal "Data Availability" section placed after Methods. Please note that the Data Availability Section is restricted to new primary data that are part of this study. * Note - All links should resolve to a page where the data can be accessed. *

If your study has not produced novel datasets, please mention this fact in the Data Availability Section.

8) At EMBO Press we ask authors to provide source data for the main manuscript figures. You will receive a separate email with instructions for providing source data with your revised manuscript, including how to upload and organize the files.

9) Our journal also encourages inclusion of *data citations in the reference list* to directly cite datasets that were re-used and obtained from public databases. Data citations in the article text are distinct from normal bibliographical citations and should directly link to the database records from which the data can be accessed. In the main text, data citations are formatted as follows: "Data ref: Smith et al, 2001" or "Data ref: NCBI Sequence Read Archive PRJNA342805, 2017". In the Reference list, data citations must be labeled with "[DATASET]". A data reference must provide the database name, accession number/identifiers and a resolvable link to the landing page from which the data can be accessed at the end of the reference.

10) Regarding data quantification, the following points must be specified in each figure legend:

- the name of the statistical test used to generate error bars and P values,

- the number (n) of independent experiments (please specify technical or biological replicates) underlying each data point,

- the nature of the bars and error bars (s.d., s.e.m.),

- If the data are obtained from n {less than or equal to} 5, show the individual data points in addition to the SD or SEM.
- If the data are obtained from n {less than or equal to} 2, use scatter blots showing the individual data points.

Discussion of statistical methodology can be reported in the materials and methods section, but figure legends should contain a basic description of n, P and the test applied.

- Please also include scale bars in all microscopy images.

11) The journal requires a statement specifying whether or not authors have competing interests (defined as all potential or actual interests that could be perceived to influence the presentation or interpretation of an article). In case of competing interests, this must be specified in your disclosure statement.

12) All Materials and Methods need to be described in the main text using our 'Structured Methods' format, which is required for all research articles. According to this format, the Methods section includes a Reagents and Tools Table (listing key reagents, experimental models, software and relevant equipment and including their sources and relevant identifiers) followed by a Methods and Protocols section describing the methods using a step-by-step protocol format. The aim is to facilitate adoption of the methodologies across labs. More information on how to adhere to this format as well as a downloadable template (.docx) for the Reagents and Tools Table can be found in our author guidelines.

We would also welcome the submission of cover suggestions, or motifs to be used by our Graphics Illustrator in designing a cover.

As part of the EMBO publication's Transparent Editorial Process, EMBO reports publishes online a Review Process File (RPF) to accompany accepted manuscripts. This File will be published in conjunction with your paper and will include the referee reports, your point-by-point response and all pertinent correspondence relating to the manuscript.

You are able to opt out of this by letting the editorial office know (emboreports@embo.org). If you do opt out, the Review

Process File link will point to the following statement: "No Review Process File is available with this article, as the authors have chosen not to make the review process public in this case."

I look forward to seeing a revised form of your manuscript when it is ready.

Yours sincerely,

Martina Rembold, PhD
Senior Editor
EMBO reports

====================

Referee #1:

Rai and colleagues have done a very thorough and convincing job showing that the COP9 signalosome regulates crystal cell specification in intermediate progenitors via Notch/Serrate and EGFR signaling. I am quite impressed by how exhaustive the authors were with respect to both controls and testing multiple pathway components/alleles throughout this work. The study advances our knowledge of both CSN mechanisms and lymph gland development, and would be suitable for publication in EMBO Reports with some revisions, as described below, to further clarify some of the mechanisms.

1. Although the effect of CSN and other perturbations on crystal cells is very convincingly demonstrated here, the effect on plasmatocytes is less clear. Presenting the data as ratios (crystal cell index:plasmatocyte index) would make the plasmatocyte results easier to interpret.

2. Although the most obvious interpretation is that there is a switch between plasmatocyte and crystal cell fates regulated by CSN, the fact that certain perturbations (e.g. Nedd8.RNAi or EGFR.DN) do not cause a concomitant increase in plasmatocyte numbers along with the observed decrease in crystal cells suggests that these processes can, at least in some cases, be decoupled. Given that N.RNAi does cause increased plasmatocyte number along with decreased crystal cells, looking at the plasmatocyte index in the N.RNAi/EGFR.CA rescue experiment might shed some light on this difference.

3. For the timecourse shown in figure 2, there is already a significant increase with CSN1b.RNAi in crystal cell number at the earliest timepoint shown (48h ALH). Is there an earlier timepoint at which the numbers are equivalent to wild-type? Or, alternatively, does this timepoint represent the beginning of crystal cell differentiation? Either way, an earlier timepoint should be shown, even if it simply shows no crystal cells in both RNAi and WT tissues.

4. The observation in Figure 3N-O that a significant number of CSN-depleted Hnt+ crystal cells still express the CHIZ>mGFP marker (compared to essentially zero cells in the WT control) suggests that these cells may still retain some intermediate cell or even progenitor-like properties. Looking at early (Lz) or late (PPO1 or PPO2) crystal cell markers in this CHIZ>mGFP+ subpopulation might provide some clarity.

5. In the cases where crystal cell number decreases significantly, apoptosis and proliferation arrest should be ruled out. At minimum, this should be done for Nedd8.RNAi, Cul.RNAi, EGFR.RNAi, and N.RNAi.

6. The data clearly show that disrupting CSN affects Notch and EGFR. However, the link between Cullin/Nedd8 and these signaling pathways is not examined. The authors should look at NRE-GFP and p-ERK in a cul.RNAi and/or Nedd8.RNAi background, and ask whether these RNAis can rescue the NICD and EGFR.CA crystal cell phenotypes.

7. Does the temporally dynamic Serrate expression pattern (Figure 5) correspond to a change in Notch signaling (as visualized by NRE-GFP or other Notch reporters)?

8. Finally, all of the experiments here utilize tissue-specific Gal4 drivers to drive gene/RNAi expression in all cells of a particular type (e.g. progenitors, intermediate cells, etc). However, given the nature of Notch as a means of transmitting a signal between two functionally distinct neighboring cells, often within the same tissue, some clone experiments seem warranted here. Specifically, the model in Figure 7V proposes that CSN can act in both the Serrate (ligand) and Notch (receptor) cells. To prove this, the authors should perform the CSN5.RNAi/Ser.RNAi and CSN5.RNAi/N.RNAi rescue experiments in somatic clones and look at both NRE-GFP activation and crystal cell index in clones vs surrounding WT tissue.

Referee #2:

Review EMBOR-2025-63028V1

Summary

The manuscript "COP9 signalosome regulates EGFR and Notch signaling during myeloid-type progenitor cell fate decision in Drosophila" identifies the role of CSN in the intermediate progenitor cells of the Drosophila lymph gland and its interaction with the EGFR and Notch signaling pathways. With a wealth of data, the authors show that CSN depletion specifically in intermediate progenitor cells drive ectopic crystal cell differentiation while decreasing the number of plasmatocytic cells. The phenotype of ectopic crystal cells is recapitulated by both EGFR and Notch pathways activation, and genetic combinations suggest that CSN knockdown promotes EGFR and Notch activation. These data are supportive of the notion that CSN loss leads to excess degradation of negative regulators of both Notch and EGFR signaling in the Drosophila lymph gland, although how CSN activity regulates Notch or EGFR activation is not specifically addressed in this manuscript. They also study the interplay between EGFR and Notch pathways in crystal cell differentiation, showing that loss of Notch rescues the excess of crystal cells caused by increase of EGFR.

The main conclusion of the manuscript; CSN regulates crystal cell and plasmatocytes in intermediate progenitors, is original and strongly supported by the presented evidence. This conclusion is sustained by the use of multiple Gal4 lines with restricted expression patterns, the use of UAS-RNAi lines targeting all subunits of the CSN complex and the use of cell-type specific markers. The analyses of Notch and EGFR is also thoughtfully carried out, although somehow less original, as previous evidences already indicated a role for Notch and Ras signaling in the specification of crystal cells. The study of functional relationships between CSN and the Notch and EGFR pathways is addressed through the analysis of genetic interactions and identification of epistatic relationships. However, the results of these experiments are mostly confirmatory, as one of the molecular targets of CSN in the EGFR pathway is known (cic), and CSN was previously identified as a regulator of Notch signaling. In this manuscript the authors suggest that Yan and Hairless are additional candidate targets of CSN but they don't follow on these possibilities.

With these considerations, the manuscript has medium novelty and biological significance, but significant importance to the specific field of cell fate assignment in the Drosophila Lymph gland. The manuscript offers an abundance of genetic combinations and developmental analyses that are not easy to follow for non-specialists, reducing the possible impact of these results outside the specific fields of Drosophila Lymph gland development. In this sense, this work would immensely benefit from a better narrative and simplified figures that retain only the most relevant data in a better organized manner.

Specific comments:

1) The main figures and supplementary figures have a very high number of panels. We strongly suggest to move redundant, confirmatory and less relevant information to supplementary material. In addition, some experiments that employ the same controls can be grouped, avoiding unnecessary repetitions that can help reduce the size of the figures. We suggest the following specific modifications:

- Figure 1: The experiments described in Figure 1W 1ZG are mostly technical confirmations that might best be passed to Supplementary information, as the evidence presented in Fig. 1A-V is pretty convincing to indicate that knockdowns of any member of the COP9 signalosome increase crystal cells and reduce plasmatocytes numbers in the LG. We also suggest to remove the DAPI staining (blue channel) from L to T.

- Figure 2: Panels A to H show that the phenotype is already evident in the earliest stage monitored (A vs. B). We fail to see the relevance of this information in the broad context of the manuscript.

- Figure 3: As it is the case for Figure 1, panels P to ZE are only technical confirmations that may be best placed as Supplementary information.

A more radical suggestion is to merge Figs. 1-3 into two figures, one for crystal cells and other for plasmatocytes. One figure could illustrate CSN components (Fig. 1A), the drivers used (dome, Tep4, Hml and Chiz; Fig. 1B and S1A), representative examples of crystal cells phenotypes (2 UAS-RNAi lines with all drivers) and the quantifications of all RNAi lines. The other Figure dedicated to the plasmatocytes organized in a similar manner.

- Figure 5: Panels O-S show how Ser-GFP and Chiz colocalization is progressively reduce in older LG. This information is not very relevant to the main points of the Figure and manuscript. We suggest to move panels O-S to Supplementary Information.

- Figure 6: This figure could also be simplified, merging together first all components of the EGFR pathway (including cic, pnt and yan), plus the combination with CSN (6G) and then the expression of relevant markers of EGFR activation (dPERK and cic)

- Figure 7: combine panels of genetic interactions to avoid repetition of controls (A-B with K-L) and then the expression of Notch pathway activation.

- Fig. 7V needs to be simplified, to suggest in a cleaner manner where the authors are placing the targets of CSN/Cullin-1 on the EGFR and Notch pathways. This panel could be made an independent figure.

We believe the authors are entitled to make the paper as they wish, but in its present form the manuscript contains too many data that are not always relevant to the main argument and are organized in a narrative that hinders more than helps the understanding of their findings. This will be an added difficulty of an already difficult manuscript for non-specialist in the field.

2) The manuscript contains many statements that lack precision or that conclude beyond the observed results. For example:

a) lines 392-395 "Results showed that crystal cell numbers increased with yanRNAi and decreased with yanAct, while Pnt manipulation had no significant effect (Figures 6ZA-ZG), indicating their dynamic participation in cell fate decisions as shown during Drosophila eye development".

These sentence lacks any specific meaning. What is "dynamic participation?". The conclusion of these experiments is that Yan seems to be required and Pnt is not.

b) Lines 461-463: "Studies reported that EGFR signaling in Drosophila eye development depends on the ratio of transcription activator Pnt and repressor Yan, with Cic also negatively regulating EGFR92,93,97,98."

Cic, as well as Pnt or Yan, regulate EGFR transcriptional targets, not EGFR itself.

c) Lines 490-493: "Since both EGFR and Notch pathways are crucial for maintaining blood cell homeostasis individually, integrating them through CSN-mediated modulation is essential for determining blood cell fate for an appropriate number of plasmatocytes and crystal cells."

The activities of EGFR and Notch are independently affected by CSN, which does not mean that CSN integrates their activities. These pathways might be integrated because existing cross interactions at any other level (cross-regulation of ligands expressions, targets coregulated, sequential activation). To affirm that CSN affects the integration between these pathways requires some level of CSN regulation by Notch or EGFR.

d) Lines 497-498: "Since depleting CSN promotes crystal cell differentiation, CSN could be targeted to enhance immune responses and respiratory health."

This may well be, but the existence of Multiple pathways downstream of CSN may prevent the usefulness of this approach.

e) Lines 116-119: "Our research findings shed new light on how the dysregulation of EGFR and Notch signaling significantly contributes to the development of cancers and other diseases, thereby facilitating the development of potential CSN-targeted therapeutic interventions.

and

Lines 500-504: "Many studies have reported that the dysregulation of EGFR and Notch signaling is a significant factor in the development of numerous cancers and diseases28,105,106. Therefore, this study enhances our understanding of these dysregulations and explores potential therapeutic interventions targeting CSN, with the potential to impact the field profoundly."

The regulation of Notch and EGFR by CSN was already known, and therefore this new study doesn't contribute to the understanding of pathologies mediated by these pathways.

Minor:

-Some panels in main Figures are repeated in Supplementary Figures, identically or with different magnifications (Fig 7S-U and Fig S7E-G;) or the same channel (pERK) merged with DAPI instead of GFP (Fig 6N-Q and Fig S6V-Y). Additionally, labeling is wrong in Fig S6V-Y (said CHIZ-mGFP).

-Is the number of cells in the intermediate and differentiated regions (Hml-Gal4) affected in CSN knock downs?

-Do the protein expression levels of Hairless, Yan, Cic change after CSN knockdown?

-What is the result of activating Notch in a background of reduced EGFR?

-Lines 281-284 : Notch-depleted intermediate progenitors (CHIZ>mGFP>NotchRNAi) and progenitors (domeMESO>EGFP>NotchRNAi) caused a significantly low number of crystal cells (Figures 5B-C, J and S5A-B, G), but no

change in CHIZ+ cells and progenitor population (Figure 5K, S5H, S5O).."

However, Fig S5H shows a reduction in the progenitor index upon Notch, Su(H), Ser and neu knockdowns.

-Line 89: "myeloid-type blood cells: Plasmatocytes, the most common are phagocytic blood cells, which". There should be a comma after "Plasmatocytes"

-Line 121: "subunits of CSN depletion" should be "depletion of CSN subunits"

-Figure 1L-T: remove DAPI channel for proper visualization of P1 staining in these panels.

-Figure S1D-G: Show only the Mys staining channel for proper visualization.

-Figure S2O-Q: indicate labeling in O'-Q' panels.

-Line 256: cite the reference of the RNAi screen.

-Line 1552: "Scale bar, 25 m except image U-X scale bar, 500 m. n.s.: not significant (p>0.01);.."
Should be "except image V-X"

-Line 338: "but crystal cells were about two-fold more when constitutively active EGFR (EGFRCA) was overexpressed in progenitors (Figures 6A, E, H)."

Information is repeated in Line 346: "EGFRCA expression in intermediate progenitors dramatically increased the number of crystal cells (Figures 6A, E, H)".

-
Answers to the questions

1. Does this manuscript report a single key finding? YES/NO
If YES, please describe it in one sentence.

YES
Identification of the function of CSN in intermediate progenitors to promote the differentiation of crystal cells.

2. Is the reported work of significance (YES), or does it describe a confirmatory finding or one that has already been documented using other methods or in other organisms etc (NO)? YES/NO

YES, although the roles of Notch (more) and EGFR (less) were already identified in the lymph gland.

3. Is it of general interest to the molecular biology community? YES/NO
If YES, please say why, in a single sentence. If NO, please state which more specialized community you feel it is aimed at (or none), in a single word or phrase.

NO. It is very much suited to the community of researches studying the Drosophila lymph glands. A more detailed analyses of how CSN regulate EGFR and Notch signaling (what are their targets) in intermediate progenitors of the lymph gland would have been of broader interest.

4. Is the single major finding robustly documented using independent lines of experimental evidence (YES), or is it really just a preliminary report requiring significant further data to become convincing, and thus more suited to a longer ¬format article (NO)? YES/NO

YES

==================

Further feedback from the referees:

Referee #2
This paper deals with proteins and pathways that are pretty well characterized at the molecular-biochemical level, and mainly

shows a plausible scenario, consistent with many previous observations, in which they can work together to specify cell fates in the developing lymph gland. For this reason, the best way to increase the interest for a broader community of molecular biologist is to offer additional mechanistic insights linking CSN with the Notch and EGFR pathways. However, this analysis is probably outside the scope of this manuscript, which essentially identifies the lymph gland as a convenient tissue to identify which proteins (mainly Ubiquitin ligases and their targets) ultimately link CSN with these pathways. With these considerations, I believe it may be unfair to demand an in-depth investigation of potential new targets, considering the amount of work the authors have already put into this analysis, but it is quite important that they made the paper much more focused and cleaner in both text and Figures. They have to identify what are the broad relevant points and discard all information that does not really contribute to make their claims. To me the key contribution of this manuscript is to identify the Drosophila lymph gland as a favorable biological system to unravel the connections between the CSN complex and the signaling pathways that regulate cell fate choices in this tissue.

Referee #1
Regarding the suitability of the topic for a broad audience, I believe that this study would be of interest to Notch biologists. Although the link between CSN and EGFR is pretty well-established, the link between CSN and Notch is much less clear.
As such, I think some further mechanistic analysis of the CSN-Notch interaction would help a lot here. I do agree with my fellow reviewer that identification of new targets is probably too much to ask. However, some of the suggestions I made in my review, particularly points 2, 6, and 8, which all address issues of mechanism with respect how CSN may regulate Notch (and EGFR), would not be too burdensome and would provide some much-needed mechanistic insight.
I also do strongly agree with the other reviewer that the sheer amount of data presented in the figures may be obfuscating some of the main points of this manuscript, particularly for a non-specialist reader, and that streamlining some of this information will help a great deal.

Response to Editorial decision letter:

We sincerely appreciate your careful handling of our manuscript for review and the receipt of reviewers' feedback following the review process. The suggested modifications to streamline the manuscript, focusing on the crucial point of our study and additional experimental data, have substantially improved the quality of this paper, as we have made diligent efforts to incorporate the reviewers' suggestions.

Responses to the reviewers' comments are provided in a point-by-point manner following the editorial letter responses. The editorial letter content and instructions are given verbatim in black, and our responses are in blue.

Dear Dr. Mondal

Thank you for the submission of your manuscript to EMBO reports. I apologize for the delay in handling it, but we have now received the full set of referee reports as well as referee cross-comments that are all pasted below.

As you will see, the referees acknowledge that the findings are potentially interesting and consider the data overall solid. The referees however also point out that the CSN has been shown to regulate Notch and EGFR before, which in turn are known players in the lymph gland. I have discussed these novelty concerns further with the referees and both consider your study of interest to Notch biologists given that the connection between CSN and Notch has not been extensively studied (Referee #1) and for the identification of the Drosophila lymph gland as a valid biological system to unravel the connections between the CSN complex and the signaling pathways that regulate fate choices.

That said, it will be crucial to focus the study, streamline it and work out the most relevant points along the lines suggested by the referees.

I would thus like to invite you to revise your manuscript with the understanding that the referee concerns must be fully addressed and their suggestions taken on board**.** Please address all referee concerns in a complete point-by-point response. Acceptance of the manuscript will depend on a positive outcome of a second round of review. It is EMBO reports policy to allow a single round of major revision only and acceptance or rejection of the manuscript will therefore depend on the completeness of your responses included in the next, final version of the manuscript.

We realize that it is difficult to revise to a specific deadline. In the interest of protecting the conceptual advance provided by the work, we recommend a revision within 3 months (23rd Mar 2026). Please discuss the revision progress ahead of this time with the editor if you require more time to complete the revisions.

You can either publish the study as a short report or as a full article. For short reports, the revised manuscript should not exceed 27,000 characters (including spaces but excluding materials & methods and references) and 5 main plus 5 expanded view figures. The results and discussion sections must further be combined, which will help to shorten the manuscript text by eliminating some redundancy that is inevitable when discussing the same experiments twice. For a normal article there are no length limitations, but it should have more than 5 main figures and the results and discussion sections must be separate. In both cases, the entire materials and methods must be included in the main manuscript file.

We thank you for the opportunity to submit a revised manuscript. We have streamlined the revised version as suggested by the reviewers and added new experimental data. We followed the

instructions in the editorial letter while preparing the revised manuscript for the EMBO Reports article. Additionally, a point-by-point response to your letter is provided below.

IMPORTANT NOTE: **We perform an initial quality control of all revised manuscripts before re-review.** Your manuscript will FAIL this control, and the handling will be DELAYED if the following APPLIES:

1) A data availability section providing access to data deposited in public databases is missing. If you have not deposited any data, please add a sentence to the data availability section that explains that.

We added a sentence to the data availability section stating that we will share all data reported in this paper upon request. This study did not generate any unique datasets or code.

2) Your manuscript contains statistics and error bars based on n=2. Please use scatter blots in these cases. No statistics should be calculated if n=2.

All experiments were performed at least 3 times, and >10 samples were used in each experiment.

When submitting your revised manuscript, please review the submission guidelines (https://link.springer.com/journal/44319/submission-guidelines#cms-Revised-submissions) and carefully review the instructions that follow below. Failure to include requested items will delay the evaluation of your revision.

1) a .docx formatted version of the manuscript text (including legends for main figures, EV figures and tables). Please make sure that the changes are highlighted to be clearly visible.

We have provided a revised manuscript in .docx format, with the changes highlighted.

2) individual production quality figure files as .eps, .tif, .jpg (one file per figure). See hhttps://media.springernature.com/original/springer-cms/rest/v1/content/27825798/data/v1 for more info on how to prepare your figures.

We have provided figures as a .tif file (one file per figure).

3) We replaced Supplementary Information with Expanded View (EV) Figures and Tables that are collapsible/expandable online. A maximum of 5 EV Figures can be typeset. EV Figures should be cited as 'Figure EV1, Figure EV2" etc... in the text and their respective legends should be included in the main text after the legends of regular figures.

- For the figures that you do NOT wish to display as Expanded View figures, they should be bundled together with their legends in a single PDF file called *Appendix*, which should start with a short Table of Content. Appendix figures should be referred to in the main text as: "Appendix Figure S1, Appendix Figure S2" etc. See 'Expanded View' section of the submission guidelines.

- Additional Tables/Datasets should be labeled and referred to as Table EV1, Dataset EV1, etc. Legends have to be provided in a separate tab in case of .xls files. Alternatively, the legend can be supplied as a separate text file (README) and zipped together with the Table/Dataset file.

We have provided figures as instructed.

4) a .docx formatted letter INCLUDING the reviewers' reports and your detailed point-by-point responses to their comments. As part of the EMBO Press transparent editorial process, the point-by-point response is part of the Review Process File (RPF), which will be published alongside your paper.

A point-by-point response to reviewers' comments provided as a .docx file.

5) a complete author checklist, which you can download from our author guidelines. Please insert information in the checklist that is also reflected in the manuscript. The completed author checklist will also be part of the RPF.

A complete author checklist is provided as a PDF file in the given format.

6) Please note that all corresponding authors are required to supply an ORCID ID for their name upon submission of a revised manuscript (<https://orcid.org/>), which can be linked to your profile in the manuscript submission system.

ORCID ID is given for the corresponding author.

7) Before submitting your revision, primary datasets produced in this study need to be deposited in an appropriate public database. Please remember to provide a reviewer password if the datasets are not yet public. The accession numbers and database should be listed in a formal "Data Availability" section placed after Methods. **Please note that the Data Availability Section is restricted to new primary data that are part of this study.** * Note - All links should resolve to a page where the data can be accessed. *

If your study has not produced novel datasets, please mention this fact in the Data Availability Section.

We added a sentence to the data availability section stating that we shared all data reported in this paper through submission portal as source data in the prescribed format. This study did not generate any unique datasets or code.

8) At EMBO Press we ask authors to provide source data for the main manuscript figures. You will receive a separate email with instructions for providing source data with your revised manuscript, including how to upload and organize the files.

We have deposited the source data as instructed in the separate email received from the editor.

9) Our journal also encourages inclusion of *data citations in the reference list* to directly cite datasets that were re-used and obtained from public databases. Data citations in the article text are distinct from normal bibliographical citations and should directly link to the database records from which the data can be accessed. In the main text, data citations are formatted as follows: "Data ref: Smith et al, 2001" or "Data ref: NCBI Sequence Read Archive PRJNA342805, 2017". In the Reference list, data citations must be labeled with "[DATASET]". A data reference must provide the database name, accession number/identifiers and a resolvable link to the landing page from which the data can be accessed at the end of the reference.

We have not used any such data in the manuscript.

10) Regarding data quantification, the following points must be specified in each figure legend:
- the name of the statistical test used to generate error bars and P values,
- the number (n) of independent experiments (please specify technical or biological replicates) underlying each data point,
- the nature of the bars and error bars (s.d., s.e.m.),
- If the data are obtained from n {less than or equal to} 5, show the individual data points in addition to the SD or SEM.
- If the data are obtained from n {less than or equal to} 2, use scatter blots showing the individual data points.

Discussion of statistical methodology can be reported in the materials and methods section, but figure legends should contain a basic description of n, P and the test applied.
- Please also include scale bars in all microscopy images.

All these points have been addressed, and the revised manuscript has been prepared accordingly.

11) The journal requires a statement specifying whether or not authors have competing interests (defined as all potential or actual interests that could be perceived to influence the presentation or interpretation of an article). In case of competing interests, this must be specified in your disclosure statement.
The authors declare no competing interests.

12) All Materials and Methods need to be described in the main text using our 'Structured Methods' format, which is required for all research articles. According to this format, the Methods section includes a Reagents and Tools Table (listing key reagents, experimental models, software and relevant equipment and including their sources and relevant identifiers) followed by a Methods and Protocols section describing the methods using a step-by-step protocol format. The aim is to facilitate adoption of the methodologies across labs. More information on how to adhere to this format as well as a downloadable template (.docx) for the Reagents and Tools Table can be found in our author guidelines.

The revised manuscript has been prepared accordingly.

We would also welcome the submission of cover suggestions, or motifs to be used by our Graphics Illustrator in designing a cover.

We love to submit cover page image if our manuscript accepted.

As part of the EMBO publication's Transparent Editorial Process, EMBO reports publishes online a Review Process File (RPF) to accompany accepted manuscripts. This File will be published in conjunction with your paper and will include the referee reports, your point-by-point response and all pertinent correspondence relating to the manuscript.

You are able to opt out of this by letting the editorial office know (emboreports@embo.org). If you do opt out, the Review Process File link will point to the following statement: "No Review Process File is available with this article, as the authors have chosen not to make the review process public in this case."
We will be happy to be part of the EMBO publication's Transparent Editorial Process.

I look forward to seeing a revised form of your manuscript when it is ready.

Yours sincerely,
Martina Rembold, PhD
Senior Editor
EMBO reports
====================

Response to reviewers' comments:

We sincerely thank both reviewers for their time, insightful comments, and constructive criticism, which have greatly improved our manuscript. The suggested changes and additional experimental data have significantly enhanced the quality of this paper, as we have made every effort to incorporate the reviewers' suggestions.

Most of the additional data included in the revised manuscript is also provided here, along with our responses to the comments. This was done to help reviewers follow the responses given the new data. Changes in the revised manuscript are highlighted for clear visibility.

Point-by-point responses to reviewers' comments are given below, with reviewers' comments given verbatim in black, and our responses in blue with embedded figures:

Referee #1:

Rai and colleagues have done a very thorough and convincing job showing that the COP9 signalosome regulates crystal cell specification in intermediate progenitors via Notch/Serrate and EGFR signaling. I am quite impressed by how exhaustive the authors were with respect to both controls and testing multiple pathway components/alleles throughout this work. The study advances our knowledge of both CSN mechanisms and lymph gland development, and would be suitable for publication in EMBO Reports with some revisions, as described below, to further clarify some of the mechanisms.

We appreciate the reviewer's thoughtful evaluation of our work and the valuable suggestions for improving our study.

1. Although the effect of CSN and other perturbations on crystal cells is very convincingly demonstrated here, the effect on plasmatocytes is less clear. Presenting the data as ratios (crystal cell index:plasmatocyte index) would make the plasmatocyte results easier to interpret.

We thank the reviewer for suggesting that presenting the data as ratios (crystal cell index: plasmatocyte index) would make the plasmatocyte results easier to interpret. Most experiments were performed on a different set of samples using either the plasmatocyte marker P1 or the crystal cell marker Hnt, as both antibodies are raised in mice. However, we performed a co-staining of P1 and PPO2 (a mature crystal cell marker) in CHIZ>mGFP-driven CSN1b^{RNAi} and CSN5^{RNAi} and observed an increased number of crystal cells, with a significant reduction in plasmatocytes (**Figures EV2Q-T**). Furthermore, when we pooled all data to analyze the plasmatocyte (P1+) and crystal cell (Hnt+) populations in the CHIZ-Gal4-driven CSN1b-CSN8 RNAi backgrounds (shown in **Figure 2**), we plotted them on the same graph.

Their index in a graph showing a shift in crystal cell formation across all CSN^{RNAi} backgrounds (n=15-50, N≥3) (**Graph below**). These pooled data indicate that CSN is involved in cell fate decisions of plasmatocytes and crystal cells (see graph below).

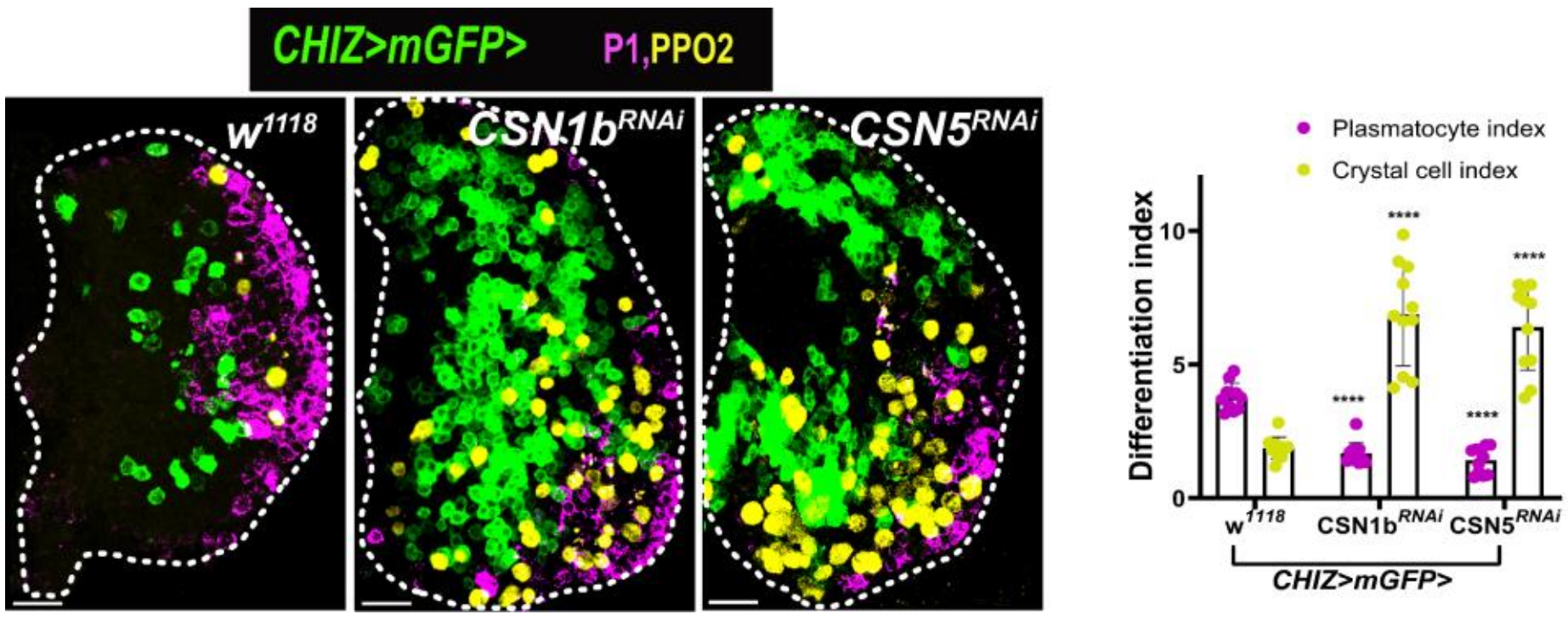

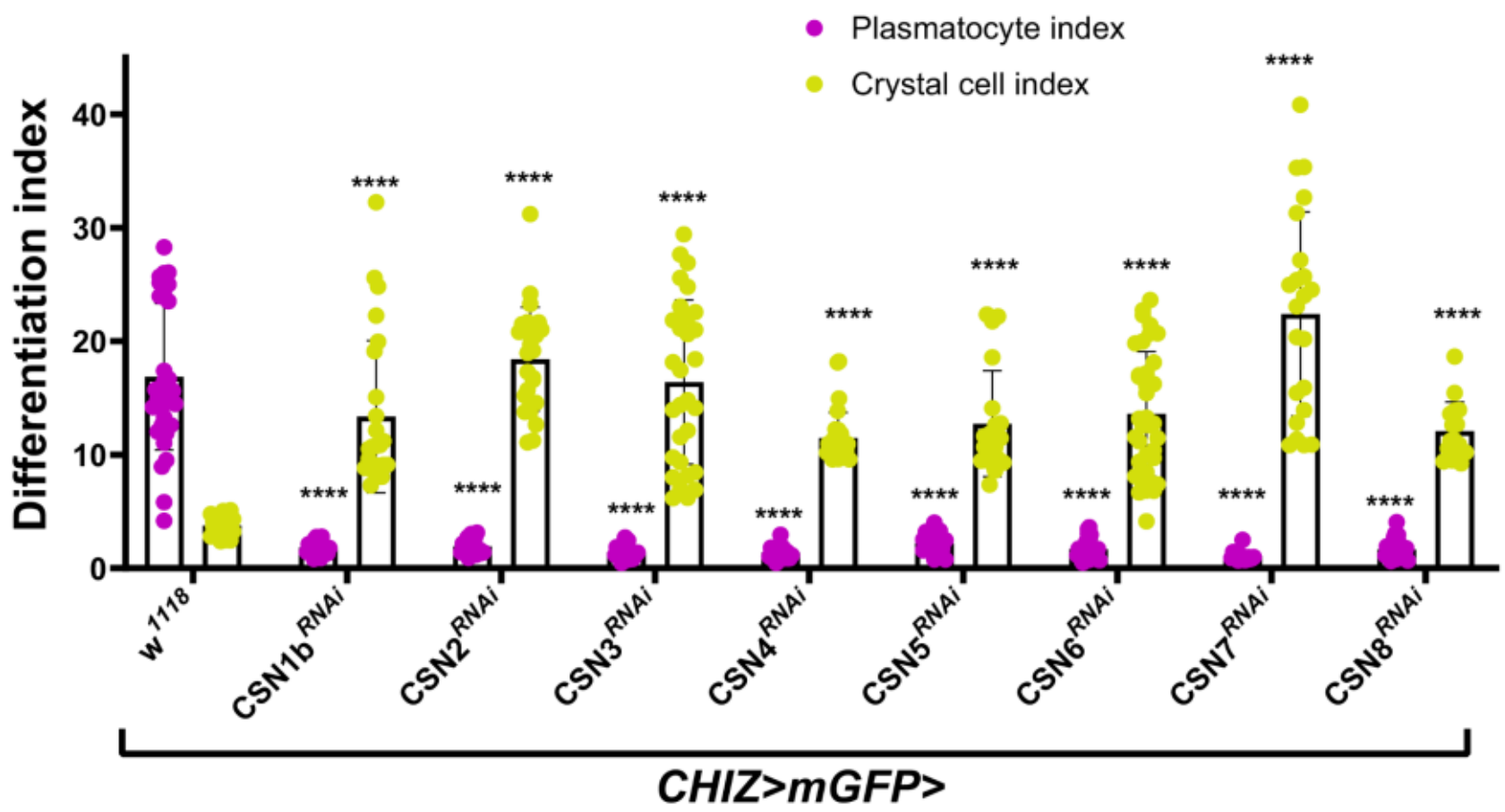

2. Although the most obvious interpretation is that there is a switch between plasmatocyte and crystal cell fates regulated by CSN, the fact that certain perturbations (e.g. Nedd8.RNAi or EGFR.DN) do not cause a concomitant increase in plasmatocyte numbers along with the observed decrease in crystal cells suggests that these processes can, at least in some cases, be decoupled. Given that N.RNAi does cause increased plasmatocyte number along with decreased crystal cells, looking at the plasmatocyte index in the N.RNAi/EGFR.CA rescue experiment might shed some light on this difference.

We thank the reviewer for this valuable suggestion and for noting that plasmatocyte numbers do not increase upon knockdown of EGFR or Nedd8. As previously reported (PMID: 38866012), depletion of EGFR results in an overall reduction in differentiated blood cells in the *Drosophila* lymph gland. However, as noted by the reviewer, we observed an increase in plasmatocyte numbers in the Notch RNAi background. To further investigate this interaction, following the reviewer's suggestion, we conducted additional experiments. A rescue experiment was performed by expressing Notch RNAi in a constitutively active EGFR background. This experiment restored plasmatocyte numbers, suggesting that EGFR is

genetically associated with Notch activation for lymph gland cell differentiation (**Appendix Figures 7A-E**).

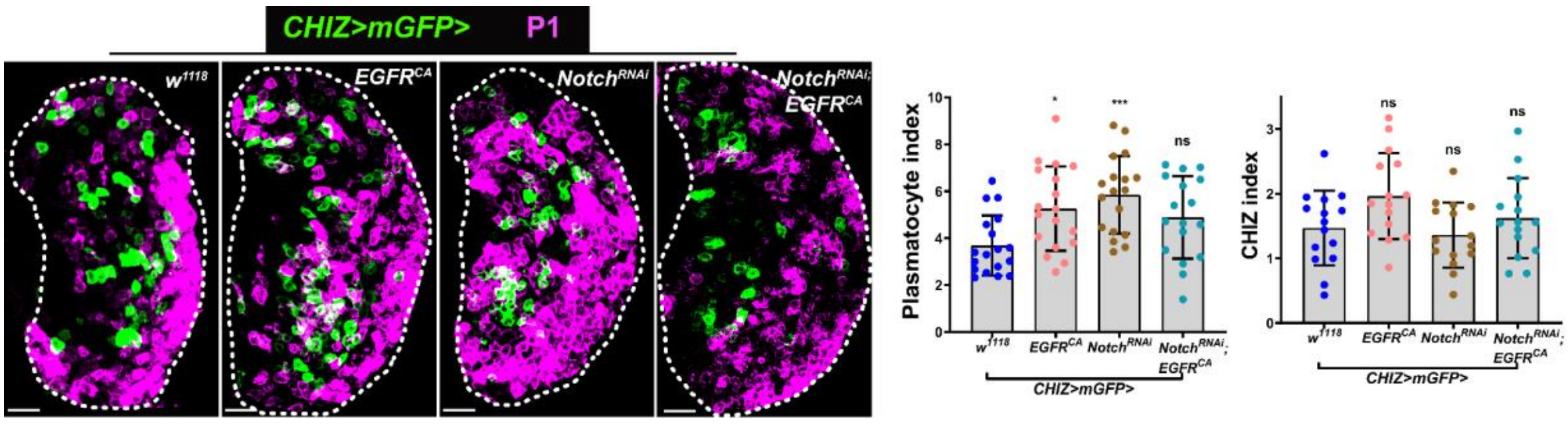

Downregulating Nedd8 decreases both crystal cells and plasmatocytes. As loss of Need8 could also affect other cullins (e.g., cullin 3, cullin 4), some of which may be necessary for cell proliferation and other functions of the lymph gland cells. Please find the six cullins screen results, along with a detailed explanation, in Reviewer 2's comment response.

3. For the timecourse shown in figure 2, there is already a significant increase with CSN1b.RNAi in crystal cell number at the earliest timepoint shown (48h ALH). Is there an earlier timepoint at which the numbers are equivalent to wild-type? Or, alternatively, does this timepoint represent the beginning of crystal cell differentiation? Either way, an earlier timepoint should be shown, even if it simply shows no crystal cells in both RNAi and WT tissues.

We thank the reviewer for pointing this out. As the reviewer noted, there is already a notable increase in crystal cell numbers at 48 hr after larval hatching **(ALH)**, which raises the question of whether this time point marks the onset of crystal cell differentiation or if there is an earlier stage where crystal cell numbers are similar to those in the wild type. To investigate this, we conducted staging experiments at 36 hr ALH and 42 hr ALH. The Hnt+ crystal cells are not detectable at 36 hr ALH, but by 42 hr ALH, they begin to differentiate, and the number of crystal cells is comparable between the control and CSN lymph gland. However, the number of crystal cells began to increase by 48 hr ALH in CSN-depleted lymph glands and remained elevated at 60 hr ALH and 72 hr ALH compared with controls (**Figures 1P-Z**).

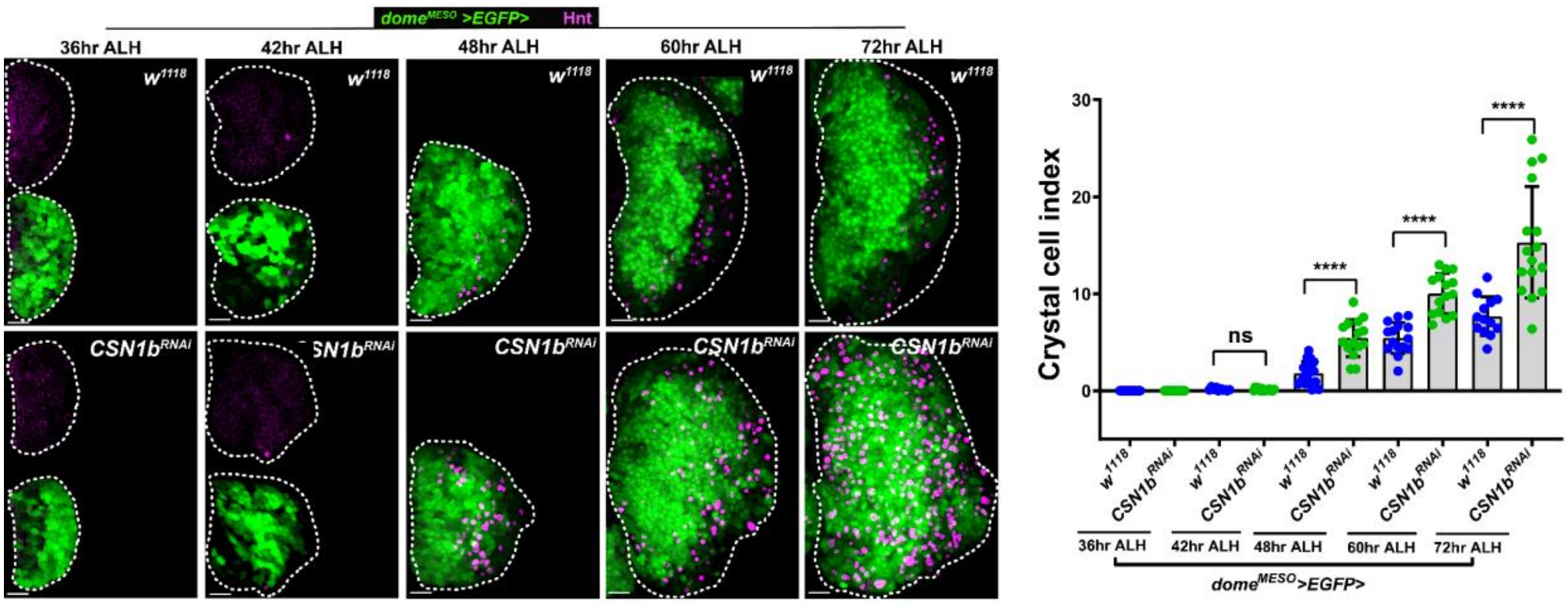

4. The observation in Figure 3N-O that a significant number of CSN-depleted Hnt+ crystal cells still express the CHIZ>mGFP marker (compared to essentially zero cells in the WT control) suggests that these cells may still retain some intermediate cell or even progenitor-like properties. Looking at early (Lz) or late (PPO1 or PPO2) crystal cell markers in this CHIZ>mGFP+ subpopulation might provide some clarity.

We thank the reviewer for raising this important question about whether these Hnt+ crystal cells retain intermediate progenitor properties. To answer this question, we would like to bring to the reviewer's attention that, under normal conditions, CHIZ cells do not express either P1 (a mature plasmatocyte marker) or Hnt (a crystal cell marker) (PMID: 34918741). However, CHIZ-Gal4-mediated knocking down CSN increases the number of Hnt+ cells and CHIZ+ cells, as well as the number of CHIZ cells expressing Hnt. This indicates that, in the CSN-depleted state, CHIZ cells (intermediate progenitors or differentiating cells) are more likely to differentiate into crystal cells. Additionally, we tested the status of the early crystal cell marker Lz and the late marker PPO2, as the reviewer suggested. We found that both Lz and PPO2 are expressed in CHIZ+ cells in a CSN-depleted background (**Appendix Figures S3K, M, N, P).** The co-expression of crystal cell markers with CHIZ>mGFP suggests that the intermediate zone cells tend to shift fate decision to crystal cells in a CSN-depleted background. This may be due to CHIZ>mGFP+ cells switching fate rapidly towards the crystal cells before the degradation of GFP protein expressed by the CHIZ-Gal4 driver.

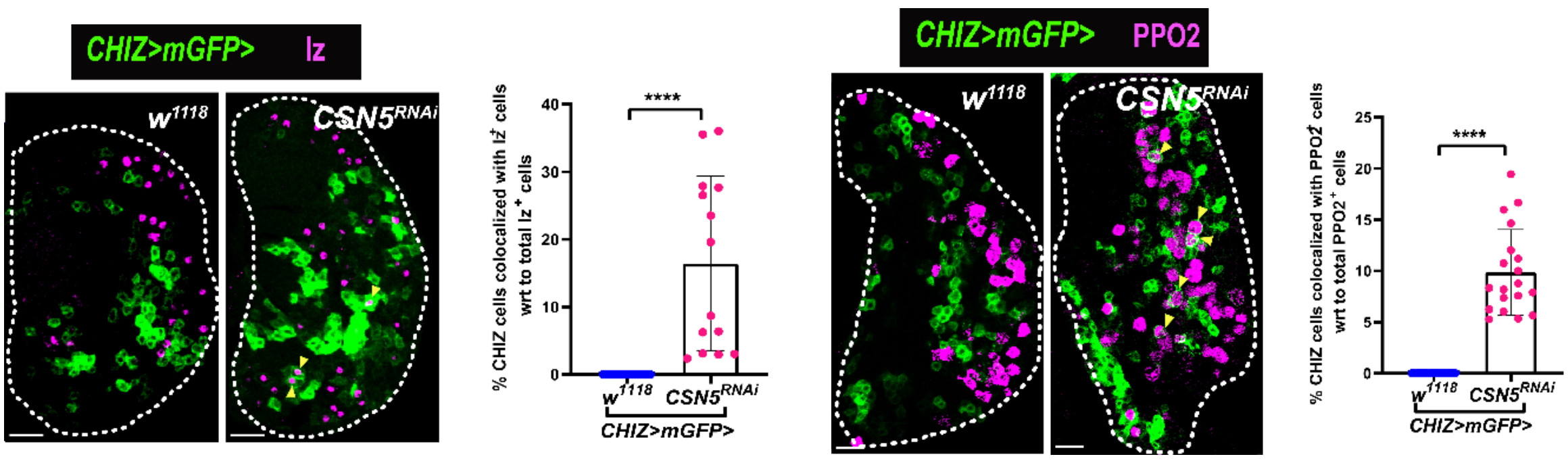

5. In the cases where crystal cell number decreases significantly, apoptosis and proliferation arrest should be ruled out. At minimum, this should be done for Nedd8.RNAi, Cul.RNAi, EGFR.RNAi, and N.RNAi.

The reviewer is concerned about whether cell proliferation and/or cell death occur in terminally differentiated crystal cells in the background of Nedd8^{RNAi}, Cul^{RNAi}, EGFR^{RNAi}, and N^{RNAi}. We performed pH3 and TUNEL staining to determine whether more cells proliferate or die (using *domeMESO> and CHIZ>* drivers) and found no significant change in the index of TUNEL-positive cells and pH3+ cells in the lymph glands of Nedd8^{RNAi}, Cul^{RNAi}, EGFR^{RNAi}, and N^{RNAi} backgrounds (**Appendix Figure S4A-P** and **S6F-U**). Therefore, none of these backgrounds alter cell proliferation or apoptosis. Our study suggests that Nedd8^{RNAi}, Cul^{RNAi}, EGFR^{RNAi}, and N^{RNAi} explicitly affect the process of cell fate decision, leading to the formation

of plasmatocytes or crystal cells, either from intermediate progenitors or later in plasmatocyte development.

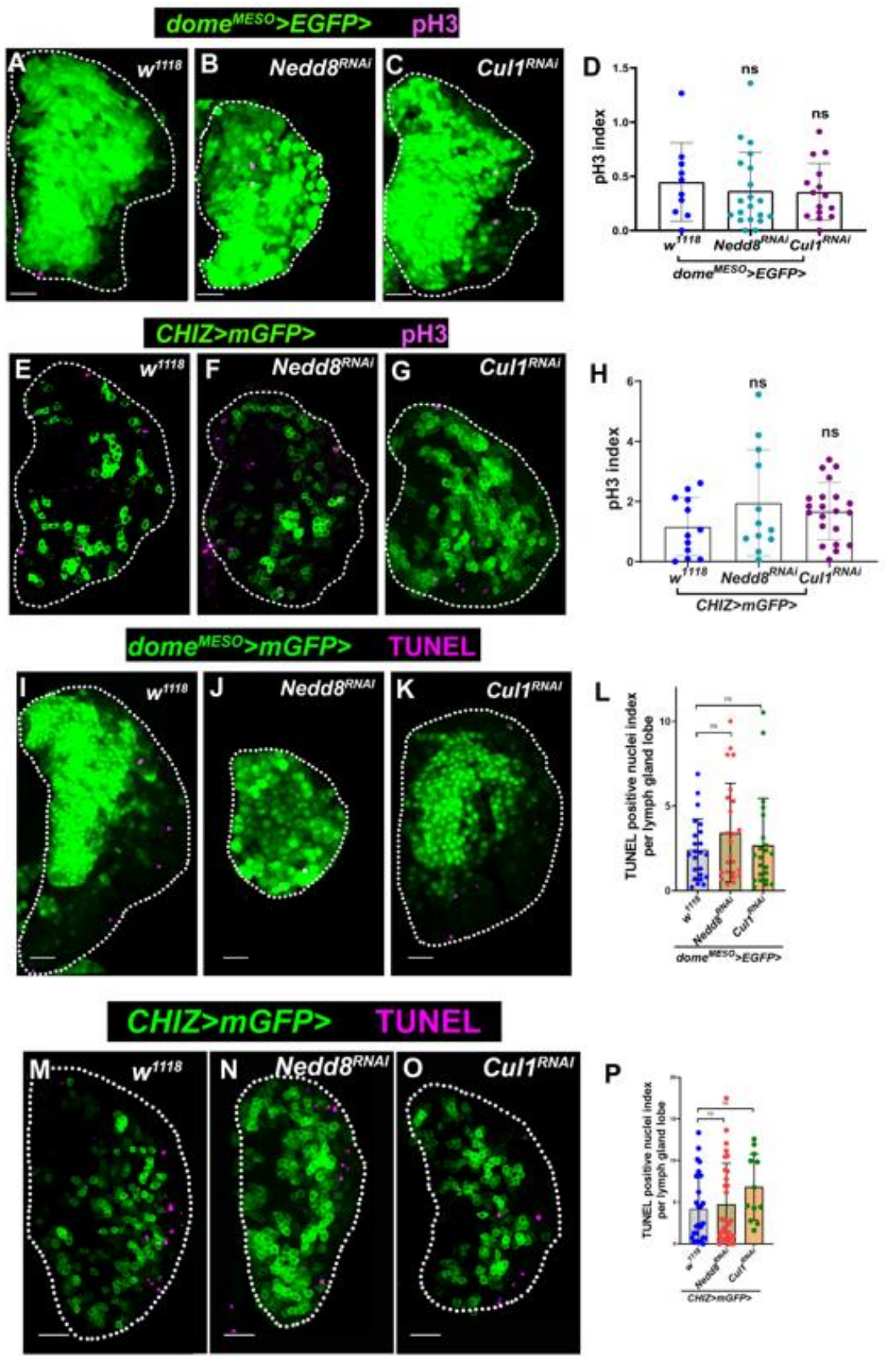

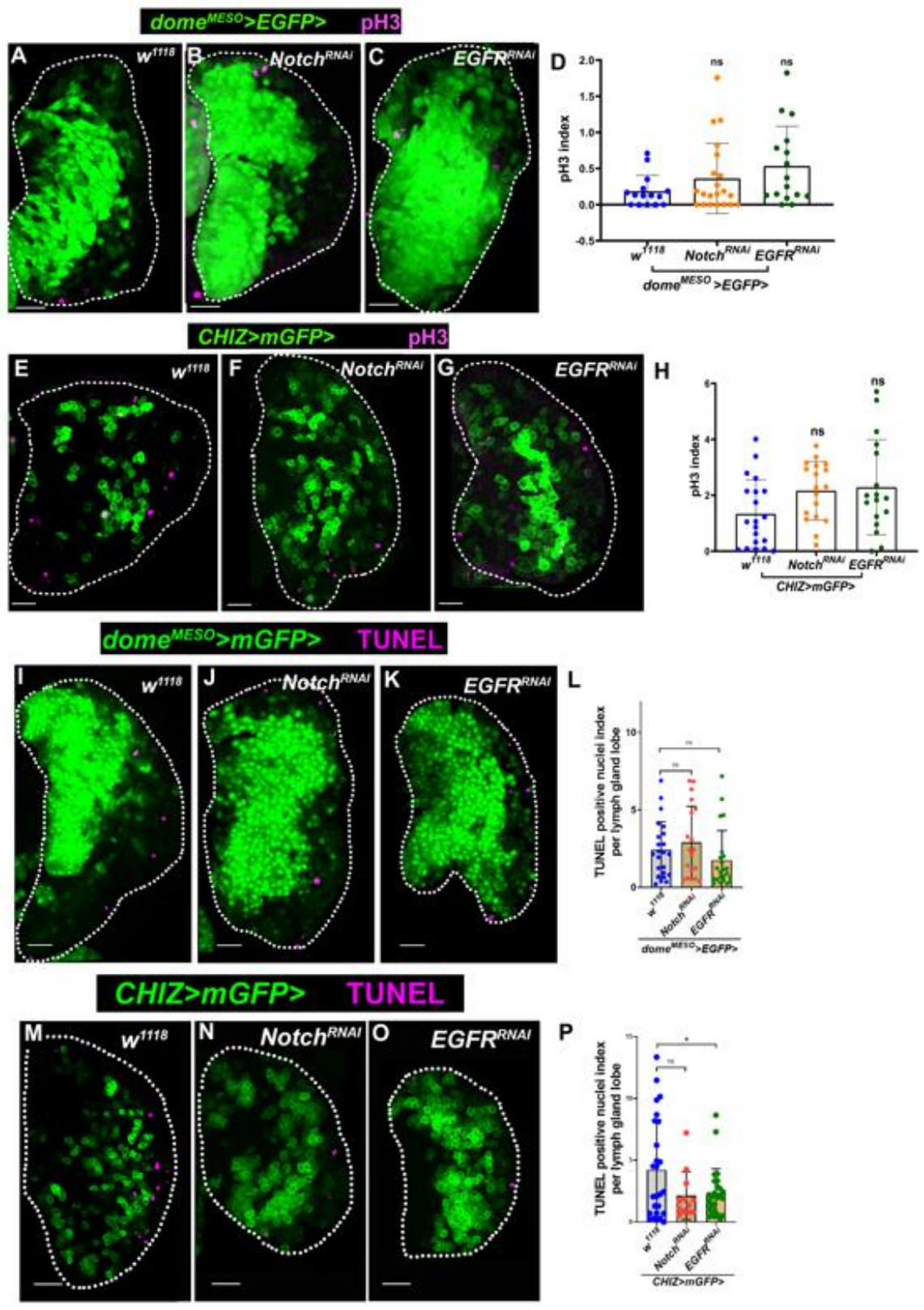

6. The data clearly show that disrupting CSN affects Notch and EGFR. However, the link between Cullin/Nedd8 and these signaling pathways is not examined. The authors should look at NRE-GFP and p-ERK in a cul.RNAi and/or Nedd8.RNAi background, and ask whether these RNAis can rescue the NICD and EGFR.CA crystal cell phenotypes.

We thank the reviewer for this suggestion to test the link between Cullin/Nedd8 and Notch/EGFR signaling pathways. We have now looked at NRE-GFP in $Cul1^{RNAi}$ and $Nedd8^{RNAi}$ backgrounds, and also tested whether these RNAi can rescue NICD and $EGFR^{CA}$ crystal cell phenotypes. Depletion of Cul1 and Nedd8 caused a decrease in NRE-GFP+ cells in the lymph gland (**Figures EV4X-ZA**). However, these RNAi lines could not rescue the increase in crystal cell number in the lymph gland caused by NICD overexpression (**Appendix Figure S7F-J**). Notch signaling may be downstream of Nedd8 and Cul1 activity during crystal differentiation in the lymph gland. However, both $Cul1^{RNAi}$ and $Nedd8^{RNAi}$ rescued a high number of crystal cell phenotypes mediated by $EGFR^{CA}$ (**Figures EV5M-S**).

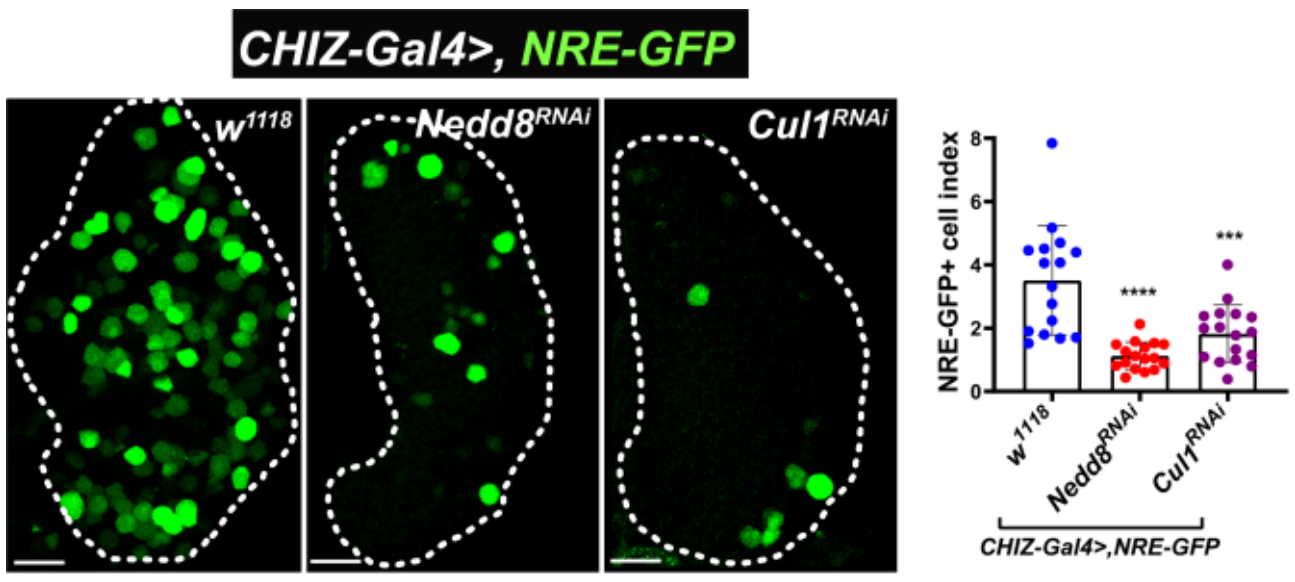

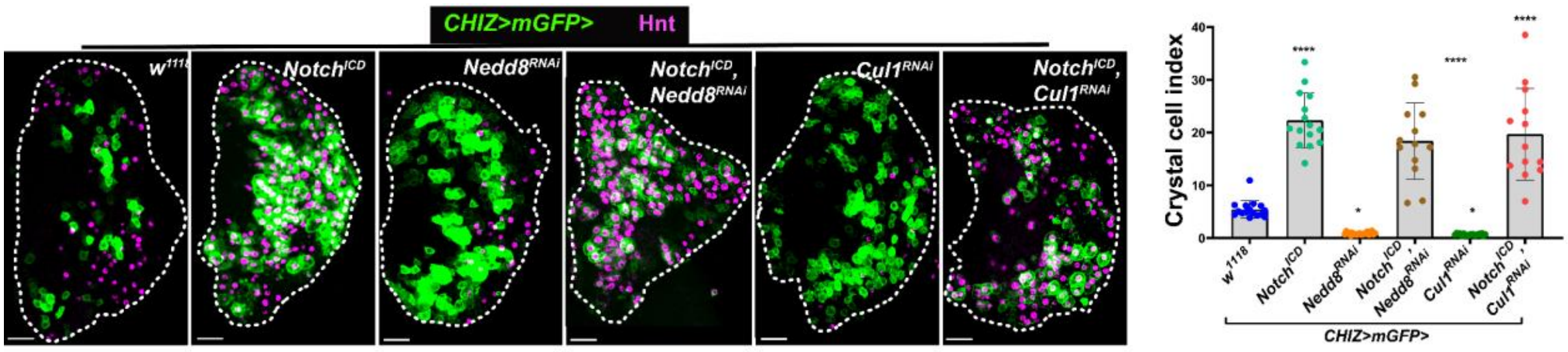

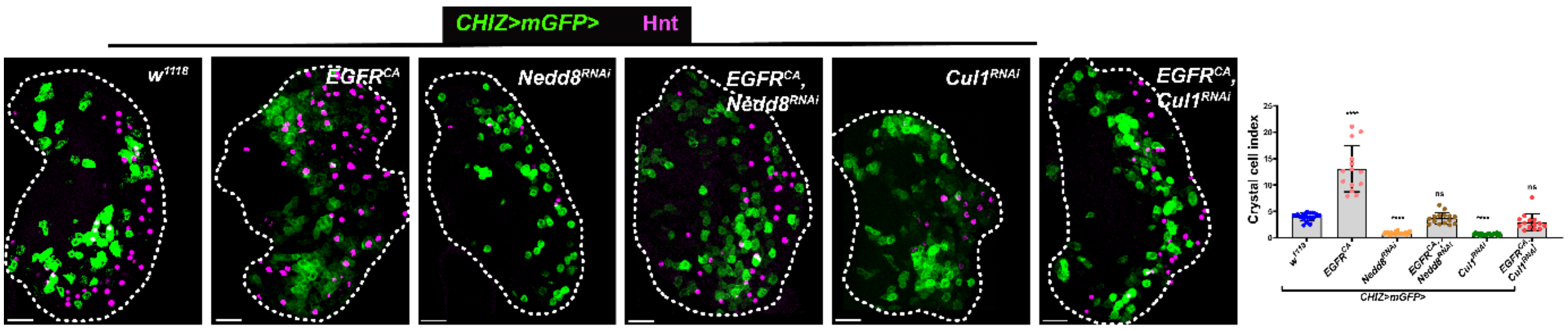

7. Does the temporally dynamic Serrate expression pattern (Figure 5) correspond to a change in Notch signaling (as visualized by NRE-GFP or other Notch reporters)?

We thank the reviewer for bringing this critical observation to our attention. Earlier studies have shown that Serrate/Notch signaling is crucial for the specification of crystal cells in the lymph gland (PMID: 21636775; 23325760). Studies also showed, using an anti-Ser antibody, that Serrate (Ser) expression begins in the lymph gland, coinciding with CHIZ>GFP+ cells (PMID: 34918741, 29992947). Since the anti-Ser antibody was unavailable, we used a Ser-GFP protein trap line that replicates the expected expression patterns in the wing disc, optic lobe, and lymph gland (**Appendix Figure S5N-P)** (PMID: 15829515, 38391176, 40075235). We found that Ser-GFP expression is dynamic, overlapping with that of intermediate progenitor cells in the mid-second instar. However, Ser-positive cells do not colocalize with crystal cells in the mid-third instar, and Ser-GFP expression drops in the wandering third instar.

Based on reviewer comments, we have now performed a time-course analysis of NRE-GFP to determine whether Serrate expression correlates with Notch activity. We found that NRE-GFP also starts to express at 36 hr ALH corresponds to Ser-GFP expression. In the later stages, the number of NRE-GFP-positive cells increases until 60 hr ALH, then remains constant and saturated because active Notch is required for crystal cell maintenance (PMID: 21636775) (**Appendix Figures S5D-G**).

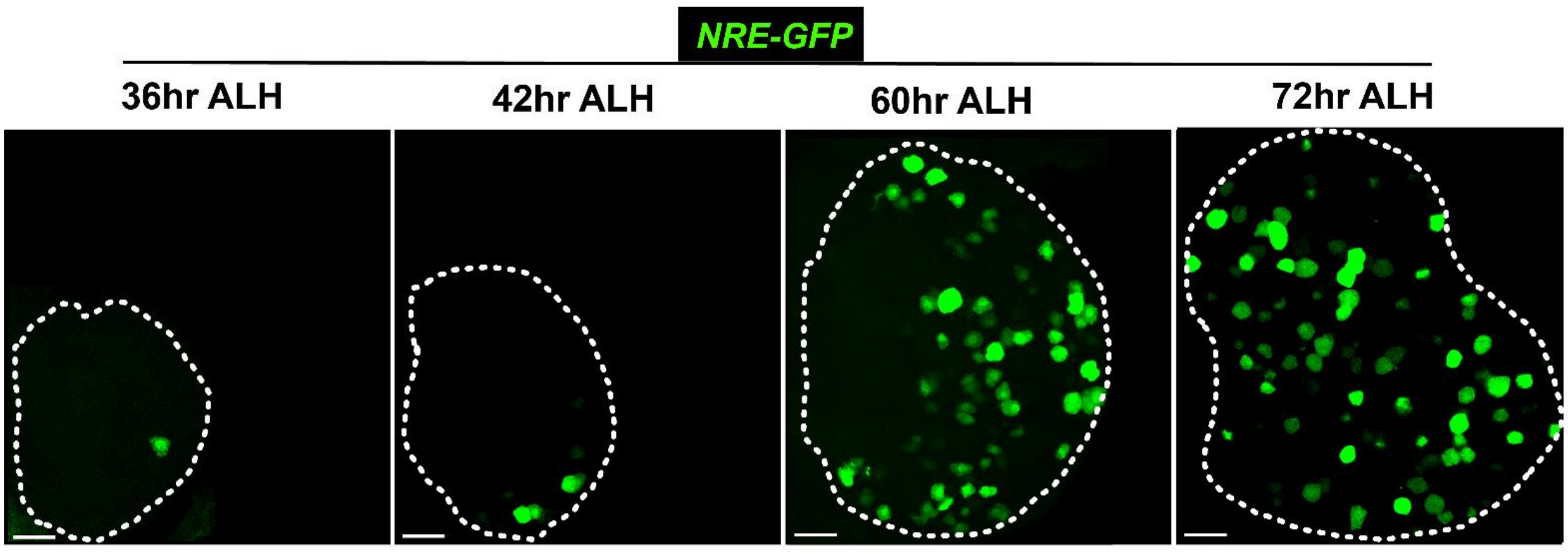

8. Finally, all of the experiments here utilize tissue-specific Gal4 drivers to drive gene/RNAi expression in all cells of a particular type (e.g. progenitors, intermediate cells, etc). However, given the nature of Notch as a means of transmitting a signal between two functionally distinct neighboring cells, often within the same tissue, some clone experiments seem warranted here. Specifically, the model in Figure 7V proposes that CSN can act in both the Serrate (ligand) and Notch (receptor) cells. To prove this, the authors should perform the CSN5.RNAi/Ser.RNAi and CSN5.RNAi/N.RNAi rescue experiments in somatic clones and look at both NRE-GFP activation and crystal cell index in clones vs surrounding WT tissue.

We thank the reviewer for studying these experiments. We want to mention that we were unable to perform a heatshock-mediated Ay-Gal4 UAS-GFP somatic clone experiment as the reviewer suggested for two reasons: first, heat shock directly affects crystal cell differentiation and survival. Second, we obtain very small somatic clones even after 45 minutes of heat shock, possibly due to the mesodermal origin of the lymph gland, which is a hematopoietic organ with multiple cell types present during *Drosophila* larval development.

However, we have tested the suggested experiments by generating a somatic clone in the developing lymph gland that spans the progenitor-to-differentiated zone using HHLT>GFP [Hand-Gal4, HmlΔ-Gal4, UAS-FLP, UAS-EGFP; Gal4-Act5C(FRT.CD2).P], a lineage trace driver for the lymph gland. Due to inefficient Flippase activity, lineage Gal4 expression is observed throughout the lymph gland, with patches (see Mondal et al. 2014; PMID: 25201876). We observed a high number of crystal cells (Hnt+) and high-intensity NICD immunostaining in the *HHLT>GFP>CSN5^{RNAi}* lymph gland cells compared to neighboring cells (**Figures 4O-P”** and **EV4ZB-ZC”**). Notably, both coexpression of *HHLT>GFP>CSN5^{RNAi};Ser^{RNAi}* and *HHLT>GFP>CSN5^{RNAi};Notch^{RNAi}* rescued crystal cell numbers and high-intensity NICD immunostaining in the *CSN5*^{RNAi}-expressing cells compared to neighboring cells in the lymph gland (**Figures 4O-T”** and **EV4ZB-ZG”**)

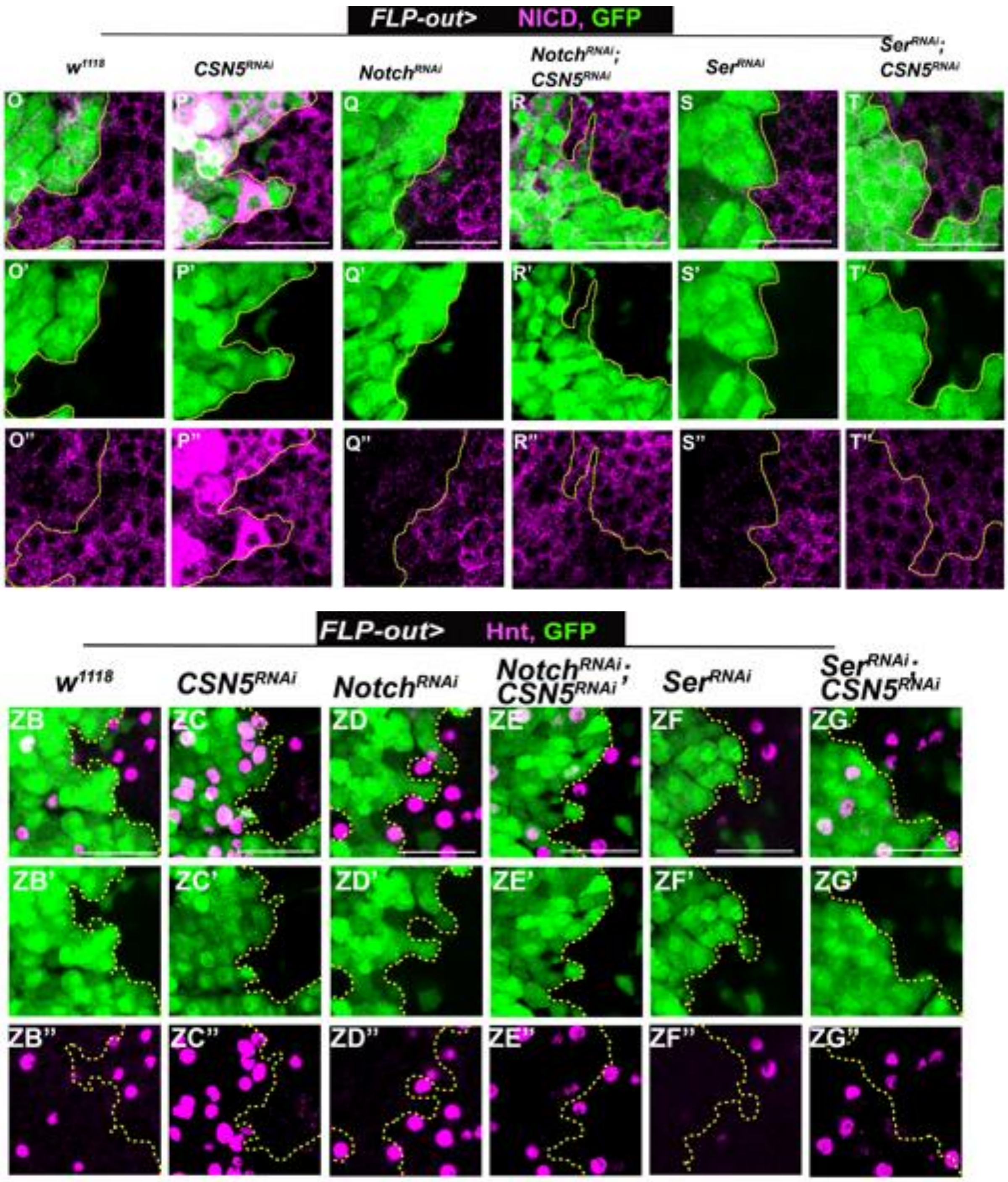
FLP-out> NICD, GFP
w^{1118}
$CSN5^{RNAi}$
$Notch^{RNAi}$
$Notch^{RNAi}$; $CSN5^{RNAi}$
Ser^{RNAi}
Ser^{RNAi}; $CSN5^{RNAi}$
O P Q R S T
O' P' Q' R' S' T'
O'' P'' Q'' R'' S'' T''
FLP-out> Hnt, GFP
w^{1118}
$CSN5^{RNAi}$
$Notch^{RNAi}$
$Notch^{RNAi}$; $CSN5^{RNAi}$
Ser^{RNAi}
Ser^{RNAi}; $CSN5^{RNAi}$
ZB ZC ZD ZE ZF ZG
ZB' ZC' ZD' ZE' ZF' ZG'
ZB'' ZC'' ZD'' ZE'' ZF'' ZG''

Referee #2:

Review EMBOR-2025-63028V1

Summary

The manuscript "COP9 signalosome regulates EGFR and Notch signaling during myeloid-type progenitor cell fate decision in Drosophila" identifies the role of CSN in the intermediate progenitor cells of the Drosophila lymph gland and its interaction with the EGFR and Notch signaling pathways. With a wealth of data, the authors show that CSN depletion specifically in intermediate progenitor cells drive ectopic crystal cell differentiation while decreasing the number of plasmatocytic cells. The phenotype of ectopic crystal cells is recapitulated by both EGFR and Notch pathways activation, and genetic combinations suggest that CSN knockdown promotes EGFR and Notch activation. These data are supportive of the notion that CSN loss leads to excess degradation of negative regulators of both Notch and EGFR signaling in the Drosophila lymph gland, although how CSN activity regulates Notch or EGFR activation is not specifically addressed in this manuscript. They also study the interplay between EGFR and Notch pathways in crystal cell differentiation, showing that loss of Notch rescues the excess of crystal cells caused by increase of EGFR.

The main conclusion of the manuscript; CSN regulates crystal cell and plasmatocytes in intermediate progenitors, is original and strongly supported by the presented evidence. This conclusion is sustained by the use of multiple Gal4 lines with restricted expression patterns, the use of UAS-RNAi lines targeting all subunits of the CSN complex and the use of cell-type specific markers. The analyses of Notch and EGFR is also thoughtfully carried out, although somehow less original, as previous evidences already indicated a role for Notch and Ras signaling in the specification of crystal cells. The study of functional relationships between CSN and the Notch and EGFR pathways is addressed through the analysis of genetic interactions and identification of epistatic relationships. However, the results of these experiments are mostly confirmatory, as one of the molecular targets of CSN in the EGFR

pathway is known (cic), and CSN was previously identified as a regulator of Notch signaling. In this manuscript the authors suggest that Yan and Hairless are additional candidate targets of CSN but they don't follow on these possibilities.

With these considerations, the manuscript has medium novelty and biological significance, but significant importance to the specific field of cell fate assignment in the Drosophila Lymph gland. The manuscript offers an abundance of genetic combinations and developmental analyses that are not easy to follow for non-specialists, reducing the possible impact of these results outside the specific fields of Drosophila Lymph gland development. In this sense, this work would immensely benefit from a better narrative and simplified figures that retain only the most relevant data in a better organized manner.

We appreciate your thorough feedback on our manuscript and value the constructive criticism, which helps us enhance its quality.

Specific comments:

1) The main figures and supplementary figures have a very high number of panels. We strongly suggest to move redundant, confirmatory and less relevant information to supplementary material. In addition, some experiments that employ the same controls can be grouped, avoiding unnecessary repetitions that can help reduce the size of the figures. We suggest the following specific modifications:

Thank you for your suggestions to streamline the manuscript by keeping relevant data in the main figures and moving confirmatory and supporting data to the supplementary material. We have now rearranged our revised manuscript according to your suggestion, with the topic focusing on how CSN regulates both Notch and EGFR signaling.

- Figure 1: The experiments described in Figure 1W 1ZG are mostly technical confirmations that might best be passed to Supplementary information, as the evidence presented in Fig. 1A-V is pretty convincing to indicate that knockdowns of any member of the COP9 signalosome increase crystal cells and reduce plasmatocytes numbers in the LG. We also suggest to remove the DAPI staining (blue channel) from L to T.

We thank the reviewer for these suggestions. We are glad that our data convinced the reviewer. We also removed the DAPI staining (blue channel) from L to T. We rearranged the figure panels by moving the technical confirmation data to supplementary information (either the Extended View or the Appendix), along with your more radical suggestion.

- Figure 2: Panels A to H show that the phenotype is already evident in the earliest stage monitored (A vs. B). We fail to see the relevance of this information in the broad context of the manuscript.

Please see below, we follow your more radical suggestion.

- Figure 3: As it is the case for Figure 1, panels P to ZE are only technical confirmations that may be best placed as Supplementary information.

Please see below, we follow your more radical suggestion.

A more radical suggestion is to merge Figs. 1-3 into two figures, one for crystal cells and other for plasmatocytes. One figure could illustrate CSN components (Fig. 1A), the drivers used (dome, Tep4, Hml and Chiz; Fig. 1B and S1A), representative examples of crystal cells phenotypes (2 UAS-RNAi lines with all drivers) and the quantifications of all RNAi lines. The other Figure dedicated to the plasmatocytes organized in a similar manner.

We thank the reviewer for this valuable suggestion to improve the revised manuscript's flow for readers. We have now rearranged the figures to merge Figs. 1-3 into two figures, as both reviewers suggested to streamline the presentation of our results. We only keep representative examples of crystal cell phenotypes (2 UAS-RNAi lines with all drivers) and the quantifications for all RNAi lines in the main figures; confirmatory or redundant data move to the Extended View or Appendix.

- Figure 5: Panels O-S show how Ser-GFP and Chiz colocalization is progressively reduce in older LG. This information is not very relevant to the main points of the Figure and manuscript. We suggest to move panels O-S to Supplementary Information.

We have moved it to **Appendix Figures S5Q-U**, as the reviewer suggested.

- Figure 6: This figure could also be simplified, merging together first all components of the EGFR pathway (including cic, pnt and yan), plus the combination with CSN (6G) and then the expression of relevant markers of EGFR activation (dPERK and cic)

We acknowledge the reviewer's suggestions and have rearranged accordingly (current **Figure 5**).

- Figure 7: combine panels of genetic interactions to avoid repetition of controls (A-B with K-L) and then the expression of Notch pathway activation.

We thank the reviewer for this valuable suggestion to combine figure panels to avoid repetition. We have rearranged accordingly (current **Figure 6**).

- Fig. 7V needs to be simplified, to suggest in a cleaner manner where the authors are placing the targets of CSN/Cullin-1 on the EGFR and Notch pathways. This panel could be made an independent figure.

We agree with the reviewer to create an independent figure featuring a cleaner schematic. We have tried our best to make a simplified model based on our genetic interaction study (current **Figure 7**).

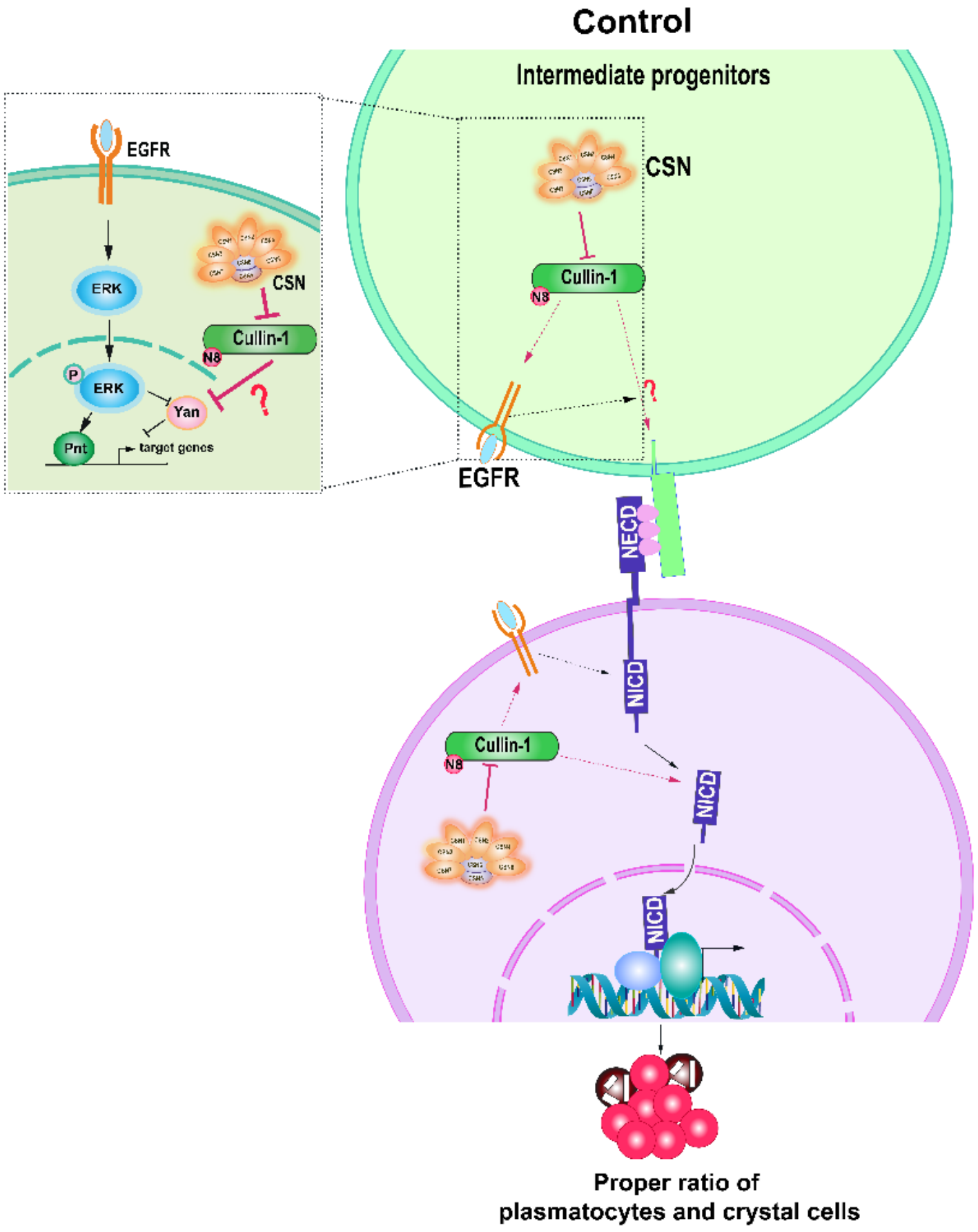

We believe the authors are entitled to make the paper as they wish, but in its present form the manuscript contains too many data that are not always relevant to the main argument and are organized in a narrative that hinders more than helps the understanding of their findings. This will be an added difficulty of an already difficult manuscript for non-specialist in the field.

We thank the reviewer for these suggestions. The revised manuscript is now streamlined by rearranging as suggested by reviewers and moving confirmatory and redundant data to either the Extended View Figures or the Appendix.

2) The manuscript contains many statements that lack precision or that conclude beyond the observed results. For example:

a) lines 392-395 "Results showed that crystal cell numbers increased with yanRNAi and decreased with yanAct, while Pnt manipulation had no significant effect (Figures 6ZA-ZG), indicating their dynamic participation in cell fate decisions as shown during Drosophila eye development".
These sentence lacks any specific meaning. What is "dynamic participation?". The conclusion of these experiments is that Yan seems to be required and Pnt is not.

We thank the reviewer for pointing this out. Many studies have shown the roles of both Yan and Pointed (Pnt) in cell fate decisions during *Drosophila* eye development. Recently,

Richard Carthew's lab (PMID: 36942737) has shown that the transcription factors Pnt and Yan regulate photoreceptor fate decisions in the *Drosophila* eye through a ratio-dependent mechanism, with dynamic participation, as inferred from genetic and immunostaining results, as well as a mathematical model. In eye progenitor cells, both proteins are co-expressed and maintain a relatively constant Pnt/Yan ratio despite dynamic fluctuations. During photoreceptor differentiation, RTK–Ras–MAPK signaling increases the Pnt/Yan ratio by activating Pnt and repressing Yan, triggering the transition to R-cell fate. Similarly, in the *Drosophila* lymph gland, Pnt and Yan show dynamic participation during blood cell differentiation. Pnt expression is variable in most lymph gland cells, with mature crystal cells showing the lowest levels (PMID:34713801). We observed Pnt-GFP expression in the lymph gland but not in Hnt^{+} crystal cells, while nuclear Yan staining was present in crystal cells (**Figures EV5X-Y**). In the CSN5-depleted intermediate progenitor background, some cells showed colocalization of Pnt-GFP and Yan (**Figures 5Y-ZA**). Functional analysis using *CHIZ-Gal4* revealed that crystal cell numbers increased with yanRNAi and decreased with activated Yan, whereas Pnt manipulation had no significant effect (**Figures 5A, G-J, M**). This parallel suggests that, as in eye development, relative activity and dynamic interaction between Pnt and Yan—rather than their absolute expression—helps regulate cell fate transitions during hematopoiesis. However, this aspect needs further investigation.

b) Lines 461-463: "Studies reported that EGFR signaling in Drosophila eye development depends on the ratio of transcription activator Pnt and repressor Yan, with Cic also negatively regulating EGFR92,93,97,98."

Cic, as well as Pnt or Yan, regulate EGFR transcriptional targets, not EGFR itself.

Thanks. We have corrected the sentence as "Studies reported that EGFR signaling in *Drosophila* eye development depends on the ratio of transcription activator Pnt and repressor Yan, with Cic also negatively regulating EGFR transcriptional targets." (Page 20, lines 515-517)

c) Lines 490-493: "Since both EGFR and Notch pathways are crucial for maintaining blood cell homeostasis individually, integrating them through CSN-mediated modulation is essential for determining blood cell fate for an appropriate number of plasmatocytes and crystal cells."

The activities of EGFR and Notch are independently affected by CSN, which does not mean that CSN integrates their activities. These pathways might be integrated because existing cross interactions at any other level (cross-regulation of ligands expressions, targets coregulated, sequential activation). To affirm that CSN affects the integration between these pathways requires some level of CSN regulation by Notch or EGFR.

We thank the reviewer for this comment. We agree with the reviewer that these pathways might be integrated due to cross-regulation at different levels, and based only on genetic analysis, it is difficult to affirm that CSN-mediated modulation of both EGFR and Notch signaling. It is also well known that EGFR and Notch signaling are cross-regulated at multiple levels of the signaling pathway in various contexts. However, how both signals are regulated

in the same cell remains unclear. Additionally, CSN is a Cullin-RING ligase-mediated proteasomal regulator that modulates various signaling pathways (Hh, EGFR, etc.) at different levels. During lymph gland development, past studies reported that EGFR signaling is important for progenitor maintenance and differentiation (PMID: 38521056, 38866012), whereas Notch/Serrate signaling specifies the crystal cell (PMID: 21636775). In this study, our genetic interaction analysis indicates CSN as a proteasomal regulator that may modulate EGFR and Notch signaling components at multiple levels, resulting in an appropriate number of plasmatocytes and crystal cells in the lymph gland. We have modified the sentence as follows: Since both the EGFR and Notch pathways are crucial for maintaining blood cell homeostasis individually, integrating them through CSN-mediated modulation at multiple cross-interactions at different levels is essential for determining blood cell fate and ensuring appropriate numbers of plasmatocytes and crystal cells." (Page 21, lines 548-551)

d) Lines 497-498: "Since depleting CSN promotes crystal cell differentiation, CSN could be targeted to enhance immune responses and respiratory health." This may well be, but the existence of Multiple pathways downstream of CSN may prevent the usefulness of this approach.

We thank the reviewer for this important comment. We agree that the CSN regulates multiple signaling pathways and cellular processes, and therefore, direct targeting of CSN could have pleiotropic effects. However, our intention was to highlight the potential biological implications of CSN-mediated regulation of crystal cell differentiation rather than propose an immediate therapeutic strategy. To address the reviewer's concern, we have revised the manuscript text to clarify that the broad regulatory role of CSN may limit its direct therapeutic targeting and that further studies will be required to determine whether specific downstream pathways can be selectively modulated.
Revised text in the manuscript:
"Since depleting CSN promotes crystal cell differentiation, CSN could be targeted to enhance immune responses and respiratory health. However, given that CSN regulates multiple signaling pathways, further studies are required to determine whether specific downstream components can be targeted without broader cellular effects." (Page 21-22, lines 557-561)

e) Lines 116-119: "Our research findings shed new light on how the dysregulation of EGFR and Notch signaling significantly contributes to the development of cancers and other diseases, thereby facilitating the development of potential CSN-targeted therapeutic interventions.
and
Lines 500-504: "Many studies have reported that the dysregulation of EGFR and Notch signaling is a significant factor in the development of numerous cancers and diseases28,105,106. Therefore, this study enhances our understanding of these dysregulations and explores potential therapeutic interventions targeting CSN, with the potential to impact the field profoundly."
The regulation of Notch and EGFR by CSN was already known, and therefore this new study doesn't contribute to the understanding of pathologies mediated by these pathways.

We thank the reviewer for this thoughtful comment. We agree that EGFR regulation by the COP9 signalosome has been reported in several biological contexts, including the *Drosophila* wing disc. Many studies also reported how EGFR and Notch signaling interact in a context-dependent manner. However, EGFR's regulation by CSN in the *Drosophila* lymph gland hematopoietic system has not been previously characterized.

We have to mention that a single genome-wide RNAi primary screen identified Notch signaling and some CSN subunits, both of which caused a similar phenotype in determining binary cell fate during *Drosophila* sensory organ development. Based on clustering algorithms (similar phenotype), the author reported that the CSN was a Notch regulator (PMID: 19363474). However, to date, no molecular mechanism or genetic interaction study has examined how CSN regulates Notch signaling. Our study provides evidence of a genetic interaction between CSN and Notch/Serrate signaling in the *Drosophila* hematopoietic context.

Our intention was to highlight that understanding how CSN regulates both EGFR and Notch signaling during hematopoietic cell fate decisions may provide broader insights into the biological regulation of these pathways, as both EGFR and Notch are misregulated in various diseases, including several cancers.

Minor:

We thank the reviewer for finding the text's errors and making suggestions. We corrected the suggested minor points and thoroughly checked any needed corrections.

-Some panels in main Figures are repeated in Supplementary Figures, identically or with different magnifications (Fig 7S-U and Fig S7E-G;) or the same channel (pERK) merged with DAPI instead of GFP (Fig 6N-Q and Fig S6V-Y). Additionally, labeling is wrong in Fig S6V-Y (said CHIZ-mGFP).

We thank the reviewer for pointing out our mistake. We have corrected the figure panels.

-Is the number of cells in the intermediate and differentiated regions (Hml-Gal4) affected in CSN knock downs?

The number of cells in the intermediate (CHIZ>mGFP+ cells) and differentiated regions (Hml>EGFP + cells) significantly increased in CSN knockdowns.

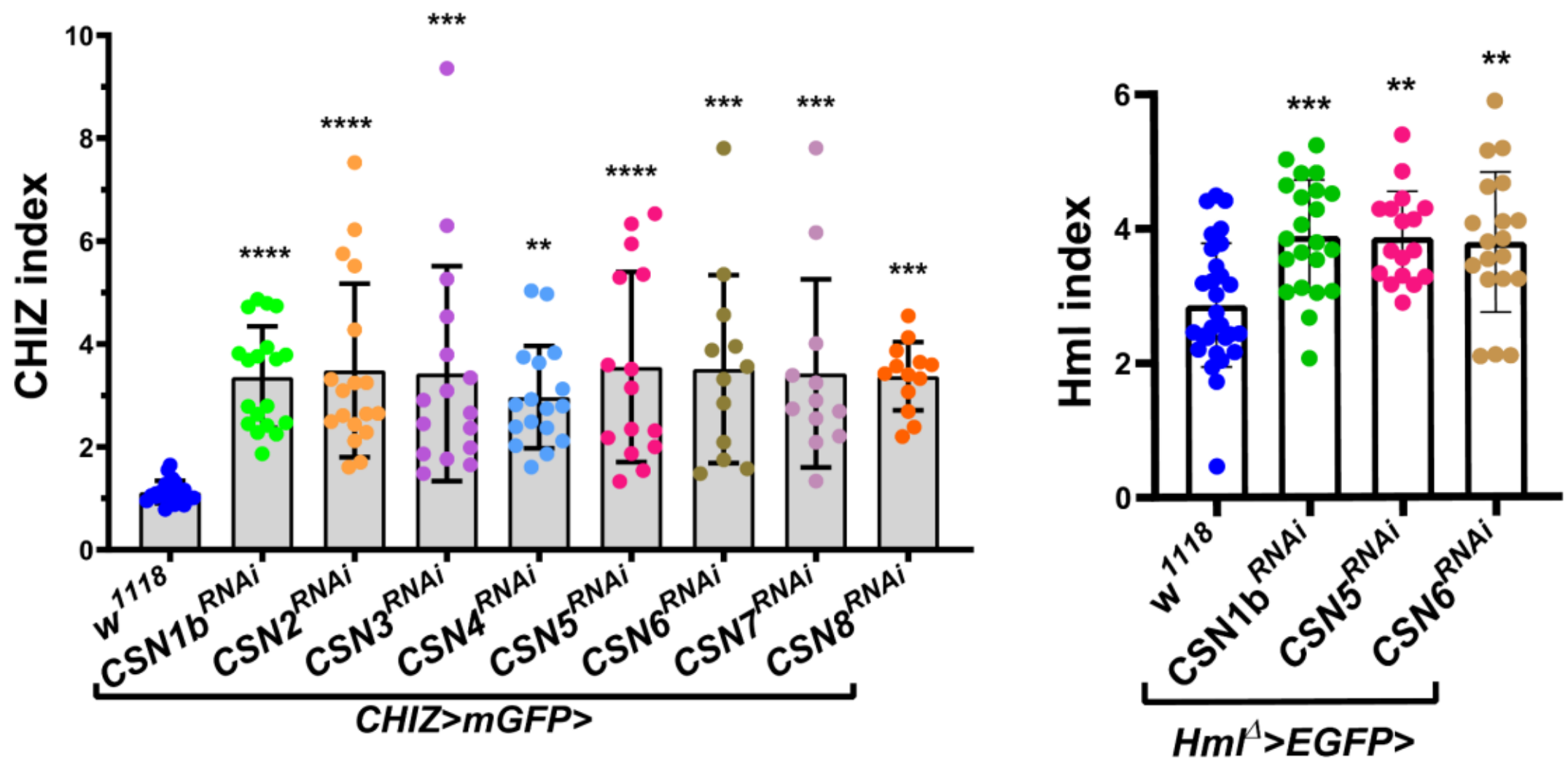

-Do the protein expression levels of Hairless, Yan, Cic change after CSN knockdown?

We have tested Yan's expression pattern in CSN knockdown, which results in increased anti-Yan immunostaining compared to controls (**Figures 5Y-ZA**). However, we were unable to test Hairless and Cic due to the unavailability of anti-Hairless and anti-Cic antibodies.

-What is the result of activating Notch in a background of reduced EGFR?

We thank the reviewer for this question. We have performed the experiment and found that EGFR RNAi could not rescue the high number of crystal cells observed upon overexpression of NICD (**Appendix Figure S7F-J**), possibly because Notch signaling is downstream of EGFR signaling in the context of crystal differentiation in the lymph gland.

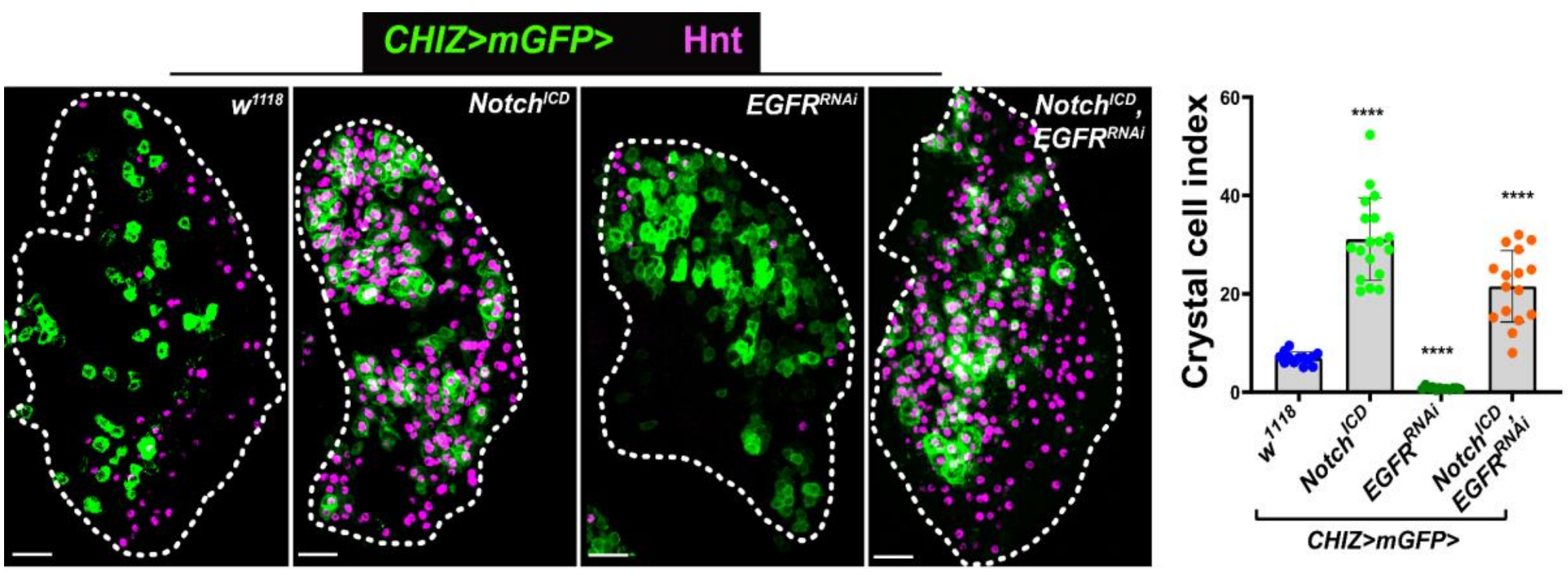

-Lines 281-284 : Notch-depleted intermediate progenitors (CHIZ>mGFP>NotchRNAi) and progenitors (domeMESO>EGFP>NotchRNAi) caused a significantly low number of crystal cells (Figures 5B-C, J and S5A-B, G), but no change in CHIZ+ cells and progenitor population (Figure 5K, S5H, S5O).."

However, Fig S5H shows a reduction in the progenitor index upon Notch, Su(H), Ser and neu knockdowns.

We thank the reviewer for pointing out the error in the text. Results slightly different, possibly due to a different Gal4 driver. We have corrected.

-Line 89: "myeloid-type blood cells: Plasmatocytes, the most common are phagocytic blood cells, which". There should be a comma after "Plasmatocytes"

This has been corrected (line: 88).

-Line 121: "subunits of CSN depletion" should be "depletion of CSN subunits"

This has been corrected.

-Figure 1L-T: remove DAPI channel for proper visualization of P1 staining in these panels.

We have removed the DAPI channel to better visualize P1 staining in these panels (current **Figures 1J-N and EV1H-N**).

-Figure S1D-G: Show only the Mys staining channel for proper visualization.

Thanks. We have shown only the Mys staging channel for proper visualization (**Appendix Figures S1A-D'**).

-Figure S2O-Q: indicate labeling in O'-Q' panels.

This has been corrected.

-Line 256: cite the reference of the RNAi screen.

We thank the reviewer for this suggestion to refer to the RNAi screen. We have screened all six cullins present in *Drosophila* to identify which one associates with the CSN-mediated lymph gland cell fate decision, particularly with the crystal cell phenotype. We found that knocking down Nedd8 does not cause the same phenotype.

Figures for referee with unpublished data and their description have been removed upon request by the authors.

Therefore, we focus only on Cul1-mediated regulation of lymph gland cells. We appreciate the reviewer for bringing up this point.

-Line 1552: "Scale bar, 25 µm except image U-X scale bar, 500 µm. n.s.: not significant (p>0.01);.."
Should be "except image V-X"

Thanks. This has been corrected.

-Line 338: "but crystal cells were about two-fold more when constitutively active EGFR (EGFRCA) was overexpressed in progenitors (Figures 6A, E, H)."

Information is repeated in Line 346: "EGFRCA expression in intermediate progenitors dramatically increased the number of crystal cells (Figures 6A, E, H)".

We thank the reviewer for pointing out an error. This has been corrected.

Response

-
Answers to the questions

1. Does this manuscript report a single key finding? YES/NO
If YES, please describe it in one sentence.

YES
Identification of the function of CSN in intermediate progenitors to promote the differentiation of crystal cells.

2. Is the reported work of significance (YES), or does it describe a confirmatory finding or one that has already been documented using other methods or in other organisms etc (NO)? YES/NO

YES, although the roles of Notch (more) and EGFR (less) were already identified in the lymph gland.

3. Is it of general interest to the molecular biology community? YES/NO
If YES, please say why, in a single sentence. If NO, please state which more specialized community you feel it is aimed at (or none), in a single word or phrase.

NO. It is very much suited to the community of researches studying the Drosophila lymph glands. A more detailed analyses of how CSN regulate EGFR and Notch signaling (what are their targets) in intermediate progenitors of the lymph gland would have been of broader interest.

4. Is the single major finding robustly documented using independent lines of experimental evidence (YES), or is it really just a preliminary report requiring significant further data to become convincing, and thus more suited to a longer ¬format article (NO)? YES/NO

YES

===================

Further feedback from the referees:

Referee #2

This paper deals with proteins and pathways that are pretty well characterized at the molecular-biochemical level, and mainly shows a plausible scenario, consistent with many previous observations, in which they can work together to specify cell fates in the developing lymph gland. For this reason, the best way to increase the interest for a broader community of molecular biologist is to offer additional mechanistic insights linking CSN with the Notch and EGFR pathways. However, this analysis is probably outside the scope of this manuscript, which essentially identifies the lymph gland as a convenient tissue to identify which proteins (mainly Ubiquitin ligases and their targets) ultimately link CSN with these pathways. With these considerations, I believe it may be unfair to demand an in-depth investigation of potential new targets, considering the amount of work the authors have already put into this analysis, but it is quite important that they made the paper much more focused and cleaner in both text and Figures. They have to identify what are the broad relevant points and discard all information that does not really contribute to make their claims. To me the key contribution of this manuscript is to identify the Drosophila lymph gland as a favorable biological system to unravel the connections between the CSN complex and the signaling pathways that regulate cell fate choices in this tissue.

We appreciate the reviewer's valuable feedback for improving this article. Based on both reviewers' comments, we addressed most questions in the revised version. This manuscript identifies the *Drosophila* lymph gland as a useful system for exploring how the CSN complex influences signaling pathways in cell fate determination. The modified version improves quality and scientific content by streamlining, as suggested by the reviewers. We hope the current draft is acceptable.

Referee #1

Regarding the suitability of the topic for a broad audience, I believe that this study would be of interest to Notch biologists. Although the link between CSN and EGFR is pretty well-established, the link between CSN and Notch is much less clear. As such, I think some further mechanistic analysis of the CSN-Notch interaction would help a lot here. I do agree with my fellow reviewer that identification of new targets is probably too much to ask. However, some of the suggestions I made in my review, particularly points 2, 6, and 8, which all address issues of mechanism with respect how CSN may regulate Notch (and EGFR), would not be too burdensome and would provide some much-needed mechanistic insight. I also do strongly agree with the other reviewer that the sheer amount of data presented in the figures may be obfuscating some of the main points of this manuscript, particularly for a non-specialist reader, and that streamlining some of this information will help a great deal.

We thank the reviewer for appreciating the work and for their constructive critique. The comments have been beneficial in drafting the revised version, and we have made every possible attempt to address every concern raised. With these new changes, either through the introduction of new data or through the reshuffling of data, as suggested by the reviewers, the revised version is much improved in overall quality and scientific content. The current draft will be acceptable.

Dear Dr. Mondal

Thank you for the submission of your revised manuscript to EMBO reports. We have meanwhile received the full set of referee reports that is copied below.

As you will see, both referees are very positive about the study and support publication pending further changes and editing of the text. Referee #2 provided a marked-up PDF with suggestions and corrections, which I attach for your information.

Browsing through the manuscript myself, I noticed a few editorial things that we need before we can proceed with the official acceptance of your study.

- The manuscript sections should be in the following order: Title page - Abstract - Introduction - Results - Discussion - Methods - Data Availability - Acknowledgments - Disclosure and Competing Interests Statement - References - Figure Legends - (Main Tables with legends if applicable) - Expanded View Figure Legends.

- Please remove the paragraph called "Materials Availability" and keep only "Data and Code Availability" but note that it needs to be renamed as 'Data Availability'. The Data Availibility section should only contain text that refers to deposition of data in public repositories. Therefore please remove the sentence "We shared all data reported in this paper through submission portal as source data in the prescribed format."

- Please upload the Author Checklist as .xls file.

- Author Checklist "Experimental study design and statistics" indicates that randomization procedures, blinding, and data inclusion/exclusion criteria were defined in the methods section. I might have missed it, but could not find information on these procedures or definitions in the methods section. Please check.

- The competing interests statements should be called "Disclosure and Competing Interests Statement".

- Regarding the Author Contributions, we use CRediT to specify the contributions of each author in the journal submission system. CRediT replaces the author contribution section, which therefore needs to be removed from the manuscript text. You can use the free text box in our system if you wish to provide more detailed descriptions. See also guide to authors https://www.embopress.org/page/journal/14693178/authorguide#authorshipguidelines.

- The information about funding listed in the manuscript text must be congruent with that in the online submission system. In this respect we noted that the grant labeling of Institute of Eminence Scheme-Seed Grant, BHU to BCM - need to be the same in manuscript and system.

- Please remove the Reagents and Tools table from the manuscript text and upload it as separate .docx file. Please use our template for this table, which you can download from our website - For authors - submission guidelines.

- Appendix file: the nomenclature when listing figures and their legends needs to be updated to Appendix Figure S1, etc.; Appendix Table S1. Please add a legend for the table.

- During our routine figure check we noted the following points that need your attention. Please carefully check the issues and please clarify by providing a point-by-point response:

A) The lymph gland shown in Figure 1L looks identical to the one shown in Figure EV1M. Fig. 1L shows CSN5-RNAi but Figure EV1M shows CSN7-RNAi. A comparison with the source data you supplied showed that the source data for Figure 1L does not match the panel. Please check these figure panels and the related source data.

B) Figure 1G (CSN5) appears identical to Figure EV5A (w1118) and 1M (CNS5) to EV5G (w1118). This applies to the panel and the corresponding source data.

C) Figure 6 P,Q,R and Figure S7 K-M' appear identical.

D) Figure EV1 H and Figure EV3 A appear identical.

- During our routine analysis of .xls source data files we detected a number of potential duplications. I attach these as color-coded files. You will see, e.g., that for Fig. 4J the numbers for w1118, line 12-15 are reproduced for neurRNAi, line 7 -10. W1118 line 19-22 matches neurRNAi line 2-5. Similar blocks of duplications are seen in the source data for Fig. 4K. Compare NotchRNAi; CSN5RNAi to SerRNAi; CSN5RNAi.
For Fig. 5M the numbers for yanCA appear to be repeated and similar patterns were obvious for EV1P, EV3E, EV4H, EV4N,

and EV5W. Please check and clarify.

- The resolution of the Appendix file is too low for microscopy images. This reduction in resolution is commonly caused by converting original 16-bit TIFF files to RGB format for publication. While this is not inherently problematic, it can raise concerns about image integrity for critical readers.

To avoid any misunderstanding and to meet EMBO Press standards, we kindly ask that you resubmit the Appendix file at the captured original data resolution.

- Figure 1P, Q, U, V: is it correct that you show the two channels for one lymph gland staining in the same image, magenta on top and green at the bottom? This needs to be indicated in the figure legend, because from 48hr ALH onwards you show only the overlay of the two channels for one (now larger) lymph gland. Please separate the top and bottom part of the images shown for 36hr and 42 hr ALH by a white space, so that it is clear that we are looking at the two distinct channels recorded for one gland.

- Please address the following points in the figure legends and mark the changes:
* Please note that the exact p values are not provided in the legends of figures 1I, O, Z, ZH, 3D, H, M; 4J, K, N, W; 5M, N, W; 6G, H, L, O; EV1 Q, EV2 O, T; EV3 E, O; EV4 G, H, N, T, U, ZA; EV5 E, F, I, L, S. You can either specify the exact p-values in the figure legend or in the figure panel itself. In case p<0.0001 it is not required to specify the exact value.
* Please note that the yellow dotted borders are not defined in the legend of figures O-T'; EV4 ZB-ZG'. This needs to be rectified.
* Please show the individual datapoints in Fig. EV1Q in additon to mean and error bars.

- Finally, EMBO Reports papers are accompanied online by
A) a short (1-2 sentences) summary of the findings and their significance,
B) 2-3 bullet points highlighting key results and
C) a schematic summary figure that provides a sketch of the major findings (not a data image).
Please provide the summary figure as a separate file in PNG or JPG format at a size of 550x300 pixels (width x height). Please note that the size is rather small and that text needs to be readable at the final size. Please send us this information along with the revised manuscript.

We look forward to seeing a final version of your manuscript as soon as possible.

With kind regards,

Martina Rembold, PhD
Senior Editor
EMBO reports

================

Referee #1:

The authors have dealt with all of the concerns outlined in my previous review in a satisfactory method. I would therefore recommend publication of this work in EMBO Reports, as I believe the science is sound and the findings of importance to the field.

However, if possible, I would recommend that the authors perform an additional round of editing of the text for grammar and clarity prior to publication. At minimum, grammatical errors need to be fixed.

In general, I found the text to be very dense and at points extremely difficult to understand, which I think does a disservice to the (excellent!) science in the paper. Many paragraphs are exhaustively long, and would probably be better split into two or more paragraphs. I also noted grammatical errors such as incomplete sentences at multiple points throughout the text. Some specific examples are listed below, although they are not exhaustive:

-- lines 302-309: The text is very hard to understand as well as being grammatically incorrect. I also believe that the mention of EGFR in this section is a mistake (possibly they meant to say Notch?)

--line 325, 1078, 1295: "plasmatocytes numbers" should be "plasmatocyte numbers"

--lines 333-334: This is grammatically incorrect.

--lines 393-396: This is not a complete sentence.

--lines 480-481: should say "crystal cell differentiation", not "crystal differentiation"

Minor point: in Figure 7, Serrate should be labeled in the diagram.

Referee #2:

The authors have made a great effort to comply with both reviewers suggestion, which has involved considerable reorganization of the manuscript and the generation of additional experimental data. The current manuscript is now more focussed, but some level of language editing should be necessary to improve the writing. Some specific suggestions are sent to the editor in case she consider appropriated sending it to the authors.

Responses to editorial and reviewers' comments are provided in a point-by-point manner. The editorial letter content and instructions are given verbatim in black, and our responses are in blue.

Dear Dr. Mondal
Thank you for the submission of your revised manuscript to EMBO reports. We have meanwhile received the full set of referee reports that is copied below.
As you will see, both referees are very positive about the study and support publication pending further changes and editing of the text. Referee #2 provided a marked-up PDF with suggestions and corrections, which I attach for your information.
Browsing through the manuscript myself, I noticed a few editorial things that we need before we can proceed with the official acceptance of your study.

We appreciate your time and effort in identifying all these errors in the manuscript we submitted, as well as your suggestions for clarification and correction.

- The manuscript sections should be in the following order: Title page - Abstract - Introduction - Results - Discussion - Methods - Data Availability - Acknowledgments - Disclosure and Competing Interests Statement - References - Figure Legends - (Main Tables with legends if applicable) - Expanded View Figure Legends.

We made the revised manuscript in the above-mentioned order.

- Please remove the paragraph called "Materials Availability" and keep only "Data and Code Availability" but note that it needs to be renamed as 'Data Availability'. The Data Availibility section should only contain text that refers to deposition of data in public repositories. Therefore please remove the sentence "We shared all data reported in this paper through submission portal as source data in the prescribed format."

We have corrected the Data Availability section in the revised manuscript as you suggested.

- Please upload the Author Checklist as .xls file.

We have uploaded the Author Checklist as an .xls file.

- Author Checklist "Experimental study design and statistics" indicates that randomization procedures, blinding, and data inclusion/exclusion criteria were defined in the methods section. I might have missed it, but could not find information on these procedures or definitions in the methods section. Please check.

We thank the editor for bringing this to our attention. In the new version of the manuscript, we have added a heading to the methods section describing the randomization procedure, blinding, and data inclusion and exclusion criteria.

"Statement on the experimental design

All experiments represent pooled data conducted using at least three independent crosses. No blinding procedure was applied in any of our experiments. Both male and female flies (adult/larvae) were included in each experiment."

- The competing interests statements should be called "Disclosure and Competing Interests Statement".

Thanks, we have changed it as suggested.

- Regarding the Author Contributions, we use CRediT to specify the contributions of each author in the journal submission system. CRediT replaces the author contribution section, which therefore needs to be removed from the manuscript text. You can use the free text box in our system if you wish to provide more detailed descriptions. See also guide to authors https://www.embopress.org/page/journal/14693178/authorguide#authorshipguidelines.

Thanks, we have removed the Author Contributions section from the manuscript text.

- The information about funding listed in the manuscript text must be congruent with that in the online submission system. In this respect we noted that the grant labeling of Institute of Eminence Scheme-Seed Grant, BHU to BCM - need to be the same in manuscript and system.

We have corrected it in the manuscript.

- Please remove the Reagents and Tools table from the manuscript text and upload it as separate .docx file. Please use our template for this table, which you can download from our website - For authors - submission guidelines.

We have removed the Reagents and Tools table from the revised manuscript text and uploaded it as a separate .docx file in the format downloaded from the EMBO Reports website.

- Appendix file: the nomenclature when listing figures and their legends needs to be updated to Appendix Figure S1, etc.; Appendix Table S1. Please add a legend for the table.

We have corrected.

- During our routine figure check we noted the following points that need your attention. Please carefully check the issues and please clarify by providing a point-by-point response:
A) The lymph gland shown in Figure 1L looks identical to the one shown in Figure EV1M. Fig. 1L shows CSN5-RNAi but Figure EV1M shows CSN7-RNAi. A comparison with the source data you supplied showed that the source data for Figure 1L does not match the panel. Please check these figure panels and the related source data.

We thank the editor for flagging this issue. The image shown in Figure 1L (CSN5 RNAi) was mistakenly duplicated from Figure EV1M (CSN7 RNAi). This was an inadvertent error during figure assembly. We have now replaced Figure 1L with the correct image for CSN5 RNAi, in accordance with the provided source data.

B) Figure 1G (CSN5) appears identical to Figure EV5A (w1118) and 1M (CNS5) to EV5G (w1118). This applies to the panel and the corresponding source data.

We apologize for this oversight. The identical appearances of these panels resulted from an error during figure preparation, in which the wrong images were assigned to the respective panels. We have carefully reviewed all panels and the corresponding source data, and the correct images have been placed in their respective figures.

C) Figure 6 P,Q,R and Figure S7 K-M' appear identical.

We thank the editor for this observation. We would like to clarify that Figure 6P, Q, R, and Appendix Figure S7 K-M' are indeed identical. Figure 6 P, Q, R are the insets (yellow dotted box) of Appendix Figure S7 K, L, M, respectively. We apologize for not mentioning it anywhere in the manuscript. We have now mentioned in the figure legend of the current version of the manuscript.

D) Figure EV1 H and Figure EV3 A appear identical.

We apologize for this error. The same image was mistakenly used for both Figure EV1H and Figure EV3A during figure assembly. The correct image for Figure EV3A has now been inserted, and the source data has been verified to match.

We sincerely regret these errors and have thoroughly re-examined all figure panels and their corresponding source data to ensure accuracy. We appreciate your diligence and patience in this matter.

- During our routine analysis of .xls source data files we detected a number of potential duplications. I attach these as color-coded files. You will see, e.g., that for Fig. 4J the numbers for w1118, line 12-15 are reproduced for neurRNAi, line 7 -10. W1118 line 19-22 matches neurRNAi line 2-5. Similar blocks of duplications are seen in the source data for Fig. 4K. Compare NotchRNAi; CSN5RNAi to SerRNAi; CSN5RNAi.
For Fig. 5M the numbers for yanCA appear to be repeated and similar patterns were obvious for EV1P, EV3E, EV4H, EV4N, and EV5W. Please check and clarify.

We sincerely thank the editor for the careful scrutiny of our source data. Upon thorough investigation, we confirm that the duplicated values identified across and within the same genotype columns in Figure 4J, 4K, 5M, EV1P, EV3E, EV4H, EV4N, and EV5W were the result of an inadvertent copy-paste error during the assembly of the source data file. Specifically, values from one genotype column were accidentally copied into another, resulting in apparent duplication across genotypes.

We want to emphasize that this was an error in preparing the submitted source data file and does not reflect an error in the underlying experimental data. We have gone back to our original raw data and replaced the erroneous entries with the correct values. The corrected source data file is attached for your review. To facilitate full transparency and verification, we have attached an Excel file containing two sheets for Figures 4J, 4K, and EV1P. **Sheet 1** contains the corrected crystal cell index (Figure 4J), CHIZ index (Figure 4K), and progenitor index (Figure EV1P) values for all genotypes across different experimental groups, along with the results of Tukey's multiple comparisons test. **Sheet 2** contains the complete raw data underlying the crystal cell index, CHIZ index, or progenitor index calculations, including the individual crystal cell counts, CHIZ volume, progenitor volume, and DAPI volumes for each sample, from which the index value was derived: Crystal index = [(crystal cell number ÷ DAPI volume) x 10000], CHIZ index = [(CHIZ volume ÷ DAPI volume) x 10], and progenitor index = [(progenitor volume ÷ DAPI volume) x 10]. Importantly, re-analysis of the data using the correct values does not alter the statistical outcomes or the conclusions drawn from these figures. The key findings reported in the manuscript remain fully supported by the corrected dataset. We deeply regret this error and apologize for any confusion it may have caused. We have carefully reviewed all other source data files to ensure no similar errors are present elsewhere in the manuscript.

- The resolution of the Appendix file is too low for microscopy images. This reduction in resolution is commonly caused by converting original 16-bit TIFF files to RGB format for publication. While this is not inherently problematic, it can raise concerns about image integrity for critical readers.
To avoid any misunderstanding and to meet EMBO Press standards, we kindly ask that you resubmit the Appendix file at the captured original data resolution.

We have uploaded an updated Appendix file (within 10 MB) with higher-resolution images.

- Figure 1P, Q, U, V: is it correct that you show the two channels for one lymph gland staining in the same image, magenta on top and green at the bottom? This needs to be indicated in the figure legend, because from 48hr ALH onwards you show only the overlay of the two channels for one (now larger) lymph gland. Please separate the top and bottom part of the images shown for 36hr and 42 hr ALH by a white space, so that it is clear that we are looking at the two distinct channels recorded for one gland.

Yes, we indeed wanted to show different channels for the same lymph gland in 36 and 42hr ALH to clearly show the Hnt positive crystal cells (magenta) because when magenta and GFP were overlaid together in these stages, the status of the crystal cells (magenta) was not clear, as you can see in the merged image 1P', Q', U' and V'. As suggested by the editor, we have included it in the figure legend (line number: 1079-1080) and separated the two channels with white space. We thank the editor for this useful suggestion.

- Please address the following points in the figure legends and mark the changes:
* Please note that the exact p values are not provided in the legends of figures 1I, O, Z, ZH, 3D, H, M; 4J, K, N, W; 5M, N, W; 6G, H, L, O; EV1 Q, EV2 O, T; EV3 E, O; EV4 G, H, N, T, U, ZA; EV5 E, F, I, L, S. You can either specify the exact p-values in the figure legend or in the figure panel itself. In case $p<0.0001$ it is not required to specify the exact value.

We have provided the exact P-value for each of the graphs mentioned above in their respective figure legends.

* Please note that the yellow dotted borders are not defined in the legend of figures O-T'; EV4 ZB-ZG'. This needs to be rectified.

Thank you. We have rectified this in the current manuscript and have mentioned in the figure legend (line no. 1196, 1502)

* Please show the individual datapoints in Fig. EV1Q in additon to mean and error bars.
We have shown the individual data points, along with the mean and error bars, in Fig. EV1Q.

- Finally, EMBO Reports papers are accompanied online by
A) a short (1-2 sentences) summary of the findings and their significance,
B) 2-3 bullet points highlighting key results and
C) a schematic summary figure that provides a sketch of the major findings (not a data image).
Please provide the summary figure as a separate file in PNG or JPG format at a size of 550x300 pixels (width x height). Please note that the size is rather small and that text needs to be readable at the final size. Please send us this information along with the revised manuscript.
We have uploaded a short summary with highlighted key results and a schematic summary figure.

We look forward to seeing a final version of your manuscript as soon as possible.

With kind regards,

Martina Rembold, PhD
Senior Editor
EMBO reports

The reviewers kindly offered helpful suggestions and comments on the revised version. We've thoughtfully addressed each comment and included a detailed, point-by-point response below. We truly appreciate the valuable advice we've received, which has improved the quality of our manuscript.

Referee #1:
The authors have dealt with all of the concerns outlined in my previous review in a satisfactory method. I would therefore recommend publication of this work in EMBO Reports, as I believe the science is sound and the findings of importance to the field.
We express our gratitude to the reviewer for acknowledging that our revised manuscript has been substantially improved and for the recommendation.

However, if possible, I would recommend that the authors perform an additional round of editing of the text for grammar and clarity prior to publication. At minimum, grammatical errors need to be fixed.
In general, I found the text to be very dense and at points extremely difficult to understand, which I think does a disservice to the (excellent!) science in the paper. Many paragraphs are exhaustively long, and would probably be better split into two or more paragraphs. I also noted grammatical errors such as incomplete sentences at multiple points throughout the text. Some specific examples are listed below, although they are not exhaustive:

-- lines 302-309: The text is very hard to understand as well as being grammatically incorrect. I also believe that the mention of EGFR in this section is a mistake (possibly they meant to say Notch?)

We thank the reviewer for pointing this out and acknowledge that the text here is indeed very difficult to understand. The reviewer is also correct that the mention of EGFR here is a mistake. We have corrected the text as follows in the manuscript:

"Notch-depleted intermediate progenitors (*CHIZ>mGFP>Notch*^{RNAi}) and progenitors (*dome*^{MESO}*>EGFP>Notch*^{RNAi}) caused a significantly low number of crystal cells (Figures 4B-C, J and EV4A-B, G) and an increased number of plasmatocytes (Figures EV4I, J, N), but no change in CHIZ+ cells (Figures 4K and EV4O), though altering the progenitor population (Figure EV4H). Also, we did not detect a significant change in the indices of TUNEL-positive cells and pH3+ cells in the lymph glands of the *NotchRNAi* background (Appendix Figures S6F-U), indicating that the loss of Notch activity does not alter cell proliferation or cell death in the lymph glands."

--line 325, 1078, 1295: "plasmatocytes numbers" should be "plasmatocyte numbers"

We have corrected it in the main text as suggested by the reviewer.

--lines 333-334: This is grammatically incorrect.

We have corrected.

--lines 393-396: This is not a complete sentence.

We thank the reviewer for pointing this out. We have corrected the sentence as follows:

"However, no significant change in the index of TUNEL-positive cells or pH3+ cells was observed in the lymph glands of *EGFR*^{RNAi} background (**Appendix Figures S6F-U**), indicating no alteration in cell proliferation or cell death in the lymph gland upon EGFR depletion."

--lines 480-481: should say "crystal cell differentiation", not "crystal differentiation"

We have corrected it in the main text.

Minor point: in Figure 7, Serrate should be labeled in the diagram.
We thank the reviewer. We have labeled Serrate in Figure 7.

Referee #2:
The authors have made a great effort to comply with both reviewers suggestion, which has involved considerable reorganization of the manuscript and the generation of additional experimental data. The current manuscript is now more focussed, but some level of language editing should be necessary to improve the writing. Some specific suggestions are sent to the editor in case she consider appropriated sending it to the authors.

We are grateful to the reviewer for language-editing suggestions, which have greatly enhanced the quality of our manuscript. We have incorporated all the recommended changes and sincerely appreciate the reviewer's valuable time and effort in helping us improve the text.

Dr. Bama Charan Mondal
Banaras Hindu University
Department of Zoology
Varanasi, Uttar Pradesh 221005
India

Dear Dr. Mondal,

I am very pleased to accept your manuscript for publication in the next available issue of EMBO reports. Thank you for your contribution to our journal.

Your manuscript will be processed for publication by EMBO Press. It will be copy edited and you will receive page proofs prior to publication. Please note that you will be contacted by Springer Nature Author Services to complete licensing and payment information.

You may qualify for financial assistance for your publication charges - either via a Springer Nature fully open access agreement or an EMBO initiative. Check your eligibility: https://link.springer.com/journal/44319/how-to-publish-with-us

Should you be planning a Press Release on your article, please notify the production team via this form in order to coordinate publication and release dates: https://forms.cloud.microsoft/e/eH4A8JVP6g

If you have any questions, please do not hesitate to contact the Editorial Office. Thank you for your contribution to EMBO Reports.

Yours sincerely,

Martina Rembold, PhD
Senior Editor
EMBO reports

>>> Please note that it is EMBO Reports policy for the transcript of the editorial process (containing referee reports and your response letter) to be published as an online supplement to each paper. If you do NOT want this, you will need to inform the Editorial Office via email immediately. More information is available here: https://link.springer.com/partners/embo-press/editorial-policies#Peer%20review

EMBO Press Author Checklist

Corresponding Author Name: Bama Charan Mondal	
Journal Submitted to: EMBO Reports	
Manuscript Number: EMBOR-2025-63028V2	

USEFUL LINKS FOR COMPLETING THIS FORM
The EMBO Journal - Author Guidelines
EMBO Reports - Author Guidelines
Molecular Systems Biology - Author Guidelines
EMBO Molecular Medicine - Author Guidelines

Reporting Checklist for Life Science Articles (updated January
This checklist is adapted from Materials Design Analysis Reporting (MDAR) Checklist for Authors. MDAR establishes a minimum set of requirements in transparent reporting in the life sciences (see Statement of Task: 10.31222/osf.io/9sm4x). Please follow the journal's guidelines in preparing your
Please note that a copy of this checklist will be published alongside your article.

Abridged guidelines for figures
1. Data
The data shown in figures should satisfy the following conditions:
- the data were obtained and processed according to the field's best practice and are presented to reflect the results of the experiments in an accurate and unbiased manner.
- ideally, figure panels should include only measurements that are directly comparable to each other and obtained with the same assay.
- plots include clearly labeled error bars for independent experiments and sample sizes. Unless justified, error bars should not be shown for technical
- if n<5, the individual data points from each experiment should be plotted. Any statistical test employed should be justified.
- Source Data should be included to report the data underlying figures according to the guidelines set out in the authorship guidelines on Data

2. Captions
Each figure caption should contain the following information, for each panel where they are relevant:
- a specification of the experimental system investigated (eg cell line, species name).
- the assay(s) and method(s) used to carry out the reported observations and measurements.
- an explicit mention of the biological and chemical entity(ies) that are being measured.
- an explicit mention of the biological and chemical entity(ies) that are altered/varied/perturbed in a controlled manner.
- the exact sample size (n) for each experimental group/condition, given as a number, not a range;
- a description of the sample collection allowing the reader to understand whether the samples represent technical or biological replicates (including how many animals, litters, cultures, etc.).
- a statement of how many times the experiment shown was independently replicated in the laboratory.
- definitions of statistical methods and measures:
  - common tests, such as t-test (please specify whether paired vs. unpaired), simple χ2 tests, Wilcoxon and Mann-Whitney tests, can be unambiguously identified by name only, but more complex techniques should be described in the methods section;
  - are tests one-sided or two-sided?
  - are there adjustments for multiple comparisons?
  - exact statistical test results, e.g., P values = x but not P values < x;
  - definition of 'center values' as median or average;
  - definition of error bars as s.d. or s.e.m.

Please complete ALL of the questions below.
Select "Not Applicable" only when the requested information is not relevant for your study.

Materials

Newly Created Materials	Information included in the manuscript?	In which section is the information available? (Reagents and Tools Table, Materials and Methods, Figures, Data Availability Section)
New materials and reagents need to be available; do any restrictions apply?	Not Applicable	

Antibodies	Information included in the manuscript?	In which section is the information available? (Reagents and Tools Table, Materials and Methods, Figures, Data Availability Section)
For **antibodies** provide the following information:<br>- Commercial antibodies: RRID (if possible) or supplier name, catalogue number and or/clone number<br>- Non-commercial: RRID or citation	Yes	Reagents and Tools Table, Methods Section

DNA and RNA sequences	Information included in the manuscript?	In which section is the information available? (Reagents and Tools Table, Materials and Methods, Figures, Data Availability Section)
**Short novel DNA or RNA including primers, probes**: provide the sequences.	Yes	Reagents and Tools Table, Methods Section

Cell materials	Information included in the manuscript?	In which section is the information available? (Reagents and Tools Table, Materials and Methods, Figures, Data Availability Section)
**Cell lines:** Provide species information, strain. Provide accession number in repository **OR** supplier name, catalog number, clone number, and/**OR** RRID.	Not Applicable	
**Primary cultures:** Provide species, strain, sex of origin, genetic modification status.	Not Applicable	
Report if the cell lines were recently **authenticated** (e.g., by STR profiling) and tested for mycoplasma contamination.	Not Applicable	

Experimental animals	Information included in the manuscript?	In which section is the information available? (Reagents and Tools Table, Materials and Methods, Figures, Data Availability Section)
**Laboratory animals or Model organisms:** Provide species, strain, sex, age, genetic modification status. Provide accession number in repository **OR** supplier name, catalog number, clone number, **OR** RRID.	Yes	Reagents and Tools Table, Methods Section
**Animal observed in or captured from the field:** Provide species, sex, and age where possible.	Not Applicable	
Please detail **housing and husbandry conditions**.	Yes	Methods Section

Plants and microbes	Information included in the manuscript?	In which section is the information available? (Reagents and Tools Table, Materials and Methods, Figures, Data Availability Section)
**Plants:** provide species and strain, ecotype and cultivar where relevant, unique accession number if available, and source (including location for collected wild specimens).	Not Applicable	
**Microbes:** provide species and strain, unique accession number if available, and source.	Not Applicable	

Human research participants	Information included in the manuscript?	In which section is the information available? (Reagents and Tools Table, Materials and Methods, Figures, Data Availability Section)
If collected and within the bounds of privacy constraints report on age, sex and gender or ethnicity for all study participants.	Not Applicable	

Core facilities	Information included in the manuscript?	In which section is the information available? (Reagents and Tools Table, Materials and Methods, Figures, Data Availability Section)
If your work benefited from core facilities, was their service mentioned in the acknowledgments section?	Not Applicable	

Design

Study protocol	Information included in the manuscript?	In which section is the information available? (Reagents and Tools Table, Materials and Methods, Figures, Data Availability Section)
If study protocol has been **pre-registered, provide DOI in the manuscript**. For clinical trials, provide the trial registration number **OR** cite DOI.	Not Applicable	
Report the **clinical trial registration number** (at ClinicalTrials.gov or equivalent), where applicable.	Not Applicable	

Laboratory protocol	Information included in the manuscript?	In which section is the information available? (Reagents and Tools Table, Materials and Methods, Figures, Data Availability Section)
Provide DOI OR other citation details if **external detailed step-by-step protocols** are available.	Not Applicable	

Experimental study design and statistics	Information included in the manuscript?	In which section is the information available? (Reagents and Tools Table, Materials and Methods, Figures, Data Availability Section)
Include a statement about **sample size** estimate even if no statistical methods were used.	Yes	Methods, Figures Section
Were any steps taken to minimize the effects of subjective bias when allocating animals/samples to treatment (e.g. **randomization procedure**)? If yes, have they been described?	Yes	Methods Section
Include a statement about **blinding** even if no blinding was done.	Yes	Methods Section
Describe **inclusion/exclusion criteria** if samples or animals were excluded from the analysis. Were the criteria pre-established?<br><br>If sample or data points were omitted from analysis, report if this was due to attrition or intentional exclusion and provide justification.	Yes	Methods Section
For every figure, are **statistical tests** justified as appropriate? Do the data meet the assumptions of the tests (e.g., normal distribution)? Describe any methods used to assess it. Is there an estimate of variation within each group of data? Is the variance similar between the groups that are being statistically compared?	Yes	Methods, Figures Section

Sample definition and in-laboratory replication	Information included in the manuscript?	In which section is the information available? (Reagents and Tools Table, Materials and Methods, Figures, Data Availability Section)
In the figure legends: state number of times the experiment was **replicated** in laboratory.	Yes	Figures Section
In the figure legends: define whether data describe **technical or biological replicates**.	Yes	Figures Section

Ethics

Ethics	Information included in the manuscript?	In which section is the information available? (Reagents and Tools Table, Materials and Methods, Figures, Data Availability Section)
Studies involving **human participants**: State details of **authority granting ethics approval** (IRB or equivalent committee(s), provide reference number for approval.	Not Applicable	
Studies involving **human participants**: Include a statement confirming that **informed consent** was obtained from all subjects and that the experiments conformed to the principles set out in the WMA Declaration of Helsinki and the Department of Health and Human Services Belmont Report.	Not Applicable	
Studies involving **human participants:** For publication of **patient photos**, include a statement confirming that consent to publish was obtained.	Not Applicable	
Studies involving experimental **animals**: State details of **authority granting ethics approval** (IRB or equivalent committee(s), provide reference number for approval. Include a statement of compliance with ethical regulations.	Not Applicable	
Studies involving **specimen and field samples:** State if relevant **permits** obtained, provide details of authority approving study; if none were required, explain why.	Not Applicable	

Dual Use Research of Concern (DURC)	Information included in the manuscript?	In which section is the information available? (Reagents and Tools Table, Materials and Methods, Figures, Data Availability Section)
Could your study fall under dual use research restrictions? Please check biosecurity documents and list of **select agents and toxins** (CDC): https://www.selectagents.gov/sat/list.htm	Not Applicable	
If you used a select agent, is the security level of the lab appropriate and reported in the manuscript?	Not Applicable	
If a study is subject to dual use research of concern regulations, is the name of the **authority granting approval and reference number** for the regulatory approval provided in the manuscript?	Not Applicable	

Reporting

The MDAR framework recommends adoption of discipline-specific guidelines, established and endorsed through community initiatives. Journals have their own policy about requiring specific guidelines and recommendations to complement MDAR.

Adherence to community standards	Information included in the manuscript?	In which section is the information available? (Reagents and Tools Table, Materials and Methods, Figures, Data Availability Section)
State if relevant guidelines or checklists (e.g., **ICMJE, MIBBI, ARRIVE, PRISMA**) have been followed or provided.	Not Applicable	
For **tumor marker prognostic studies,** we recommend that you follow the **REMARK** reporting guidelines (see link list at top right). See author guidelines, under 'Reporting Guidelines'. Please confirm you have followed these guidelines.	Not Applicable	
For **phase II and III randomized controlled trials**, please refer to the **CONSORT** flow diagram (see link list at top right) and submit the CONSORT checklist (see link list at top right) with your submission. See author guidelines, under 'Reporting Guidelines'. Please confirm you have submitted this list.	Not Applicable	

Data Availability

Data availability	Information included in the manuscript?	In which section is the information available? (Reagents and Tools Table, Materials and Methods, Figures, Data Availability Section)
Have **primary datasets** been deposited according to the journal's guidelines (see 'Data Deposition' section) and the respective accession numbers provided in the Data Availability Section?	Not Applicable	
Were **human clinical and genomic datasets** deposited in a public access-controlled repository in accordance to ethical obligations to the patients and to the applicable consent agreement?	Not Applicable	
Are **computational models** that are central and integral to a study available without restrictions in a machine-readable form? Were the relevant accession numbers or links provided?	Not Applicable	
If publicly available data were reused, provide the respective **data citations in the reference list**.	Not Applicable	
